# The bowfin genome illuminates the developmental evolution of ray-finned fishes

**DOI:** 10.1038/s41588-021-00914-y

**Published:** 2021-08-30

**Authors:** Andrew W. Thompson, M. Brent Hawkins, Elise Parey, Dustin J. Wcisel, Tatsuya Ota, Kazuhiko Kawasaki, Emily Funk, Mauricio Losilla, Olivia E. Fitch, Qiaowei Pan, Romain Feron, Alexandra Louis, Jérôme Montfort, Marine Milhes, Brett L. Racicot, Kevin L. Childs, Quenton Fontenot, Allyse Ferrara, Solomon R. David, Amy R. McCune, Alex Dornburg, Jeffrey A. Yoder, Yann Guiguen, Hugues Roest Crollius, Camille Berthelot, Matthew P. Harris, Ingo Braasch

**Affiliations:** 1 Department of Integrative Biology, Michigan State University, East Lansing, MI USA; 2 Ecology, Evolution & Behavior Program, Michigan State University, East Lansing, MI USA; 3 Department of Genetics, Harvard Medical School, Boston, MA USA; 4 Department of Orthopedic Research, Boston Children’s Hospital, Boston, MA USA; 5 Department of Organismic and Evolutionary Biology, Harvard University, Cambridge, MA USA; 6 Museum of Comparative Zoology, Harvard University, Cambridge, MA USA; 7 Institut de Biologie de l’ENS (IBENS), Département de Biologie, École Normale Supérieure, CNRS, INSERM, Université PSL, Paris, France; 8 Department of Molecular Biomedical Sciences, NC State University, Raleigh, NC USA; 9 Department of Evolutionary Studies of Biosystems, SOKENDAI (the Graduate University for Advanced Studies), Hayama, Japan; 10 Department of Anthropology, Pennsylvania State University, University Park, PA USA; 11 Department of Ecology and Evolutionary Biology, Cornell University, Ithaca, NY USA; 12 Department of Ecology and Evolution, University of Lausanne, Lausanne, Switzerland; 13 Swiss Institute of Bioinformatics, Lausanne, Switzerland; 14 INRAE, LPGP, Rennes, France; 15 GeT-PlaGe, INRAE, Genotoul, Castanet-Tolosan, France; 16 Department of Plant Biology, Michigan State University, East Lansing, MI USA; 17 Department of Biological Sciences, Nicholls State University, Thibodaux, LA USA; 18 Department of Bioinformatics and Genomics, University of North Carolina at Charlotte, Charlotte, NC USA; 19 Comparative Medicine Institute, NC State University, Raleigh, NC USA; 20 Center for Human Health and the Environment, NC State University, Raleigh, NC USA; 21 Present Address: Animal Science Department, University of California Davis, Davis, CA USA

**Keywords:** Genomics, Developmental biology, Zoology

## Abstract

The bowfin (*Amia calva*) is a ray-finned fish that possesses a unique suite of ancestral and derived phenotypes, which are key to understanding vertebrate evolution. The phylogenetic position of bowfin as a representative of neopterygian fishes, its archetypical body plan and its unduplicated and slowly evolving genome make bowfin a central species for the genomic exploration of ray-finned fishes. Here we present a chromosome-level genome assembly for bowfin that enables gene-order analyses, settling long-debated neopterygian phylogenetic relationships. We examine chromatin accessibility and gene expression through bowfin development to investigate the evolution of immune, scale, respiratory and fin skeletal systems and identify hundreds of gene-regulatory loci conserved across vertebrates. These resources connect developmental evolution among bony fishes, further highlighting the bowfin’s importance for illuminating vertebrate biology and diversity in the genomic era.

## Abstract (web summary)

Analysis of a chromosome-level bowfin genome assembly sheds light into neopterygian fish evolution. Chromatin accessibility and gene expression profiling provides insight into bowfin embryonic development.

## Main

The monotypic bowfin, *A. calva* (Linnaeus, 1776), is a textbook example in comparative anatomy for its prototypical fish body plan and key phylogenetic position^1,2^. Bowfin biology thus sheds light on the evolution and development of ray-finned fishes and bony vertebrates in general. Ray-finned fishes constitute the most diverse vertebrate lineage with >30,000 living species, of which >96% belong to the teleost fishes (Teleostei)^3^. The bowfin (Amiiformes) and seven gar species (Lepisosteiformes) represent the extant Holostei, the sister lineage of teleost fishes, together comprising the Neopterygii^4–8^. These eight holosteans, however, capture just a minor fraction of this once speciose lineage. The fossil record shows that the biodiversity of holosteans is highly underappreciated, as they were much more abundant in the past and as species rich as stem teleosts^9^.

With a multitude of teleost and a few non-teleost species, including spotted gar (*Lepisosteus oculatus*), sequenced^7,10–13^, the bowfin represents the last major neopterygian fish lineage remaining for detailed genomic and developmental exploration. Comparing bowfin and gar covers the maximum holostean genomic diversity available to study, and, due to their early divergence during holostean evolution >250 million years ago^8^, also spans the vast majority of holosteans to ever have existed^9^.

The bowfin is an important outgroup to investigate teleost evolution and development. The common ancestor of extant teleosts underwent the teleost whole-genome duplication (TGD), resulting in a sizeable fraction of often functionally divergent gene duplicates in living teleosts^14,15^. Genomic comparisons of teleosts with tetrapods such as humans are thus challenging, for example, in biomedical studies leveraging teleost models such as zebrafish, medaka, killifish or cavefish^10^. Bowfin and gar diverged from teleosts before the TGD and thus maintained more one-to-one gene relations with tetrapods^7^ and also have slower rates of molecular sequence evolution than teleosts^7,16^. Thus, the genome of the gar has been used as an intermediate steppingstone or ‘bridge’ for identifying hidden orthologies of genetic elements between teleosts and tetrapods^7^. By adding the bowfin genome, the ‘holostean bridge’ will capture more genomic diversity across the ray-finned tree of life and resolve critical questions about vertebrate evolution for which analyzing only one holostean representative is insufficient.

Importantly, within Neopterygii, the relations of bowfin, gars and teleosts have been matter of a long-standing, controversial debate (for example, refs. ^17–19^) with two main proposed alternative scenarios. Bowfin has been grouped with gars in the Holostei clade with strong phylogenomic support (for example, refs. ^4–8,12^), but, due to similarities in morphology, bowfin has also been historically grouped together with teleosts as Halecostomi (for example, refs. ^17–20^). This Halecostomi scenario could further reconcile similarities of bowfin and teleost karyotypes^21^. A chromosomal bowfin genome assembly is thus essential to test these two alternative scenarios and to develop a comprehensive evolutionary framework for understanding phenotypic evolution among ray-finned fishes.

Bowfin’s unique developmental, morphological, immunological and behavioral phenotypes, including pronounced sexual dimorphism^2^, an evolutionarily informative immune system^22^, a derived type of dermal scales^23^, air breathing with a respiratory gas bladder^24,25^, and a largely ancestral fin skeleton^1,20^, are ripe for study with genomic sequence information. Here, we report a chromosome-level genome assembly that allows us to finally settle bowfin’s phylogenetic position and to investigate chromosomal evolution, key gene families and gene-regulatory regions, as well as developmental processes in bowfin. We increase the utility of holostean genomic resources by characterizing chromatin accessibility and gene expression through bowfin development, enabling comparisons of non-coding regulatory regions across model and non-model fishes and tetrapods. The inclusion of the bowfin genomic landscape permits translation of genetic and genomic changes underlying vertebrate evolution and its regulation.

## Results

### The bowfin genome

**Extended Data Fig. 1:**
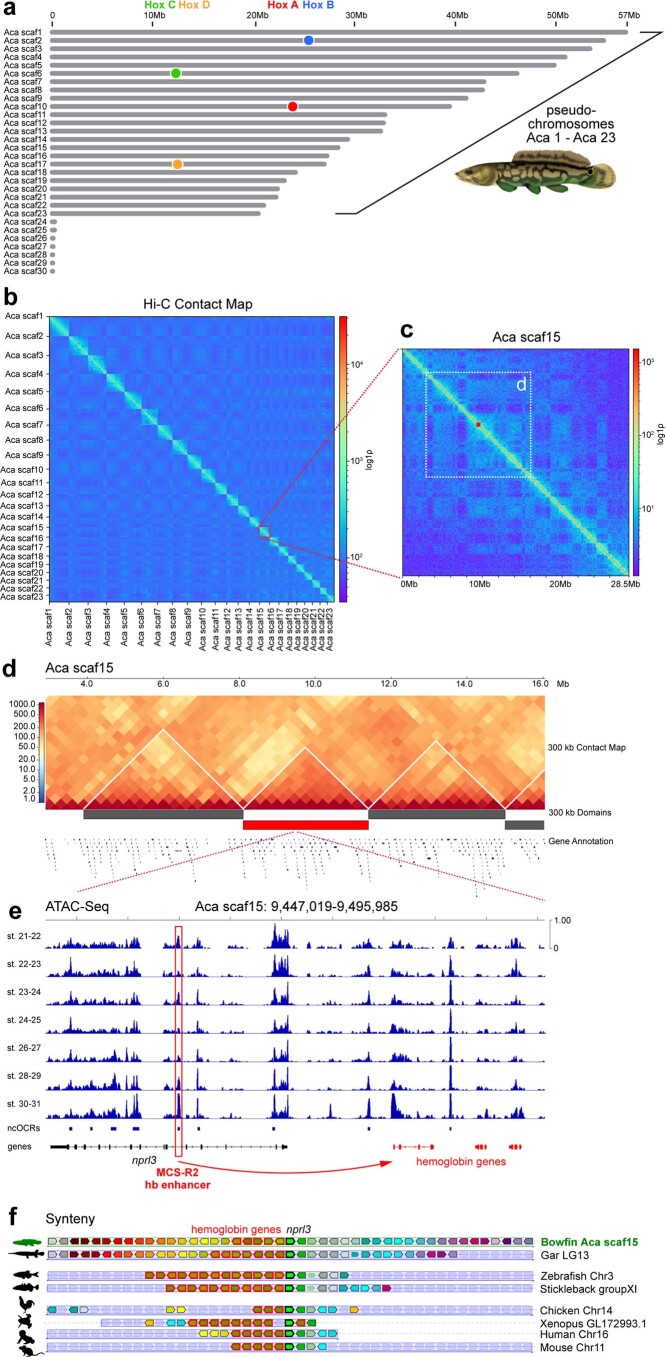
The bowfin genome. (**a**) Size distribution of the 30 largest scaffolds in the AmiCal1 assembly. Aca scaf1 to Aca scaf23 match the number of chromosomes in the bowfin genome^21,29^ and are thus considered pseudochromosomes. Locations of the four bowfin Hox gene clusters Hox A-D are indicated by colored circles. (**b**) Hi-C contact map for the 23 pseudochromosomes, generated from blood sample. Resolution: 500-kb bins. The red dashed box indicates Aca scaf15. (**c**) Hi-C contact map for Aca scaf15. Resolution: 50-kb bins. (**d**) Topologically Associating Domains (TADs) called using a resolution of 300-kb bin size in the region of Aca scaf15 indicated by the white dashed box in (**c**). (**e**) ATAC-Seq profile through bowfin development within the central TAD in (**d**). ATAC-Seq peaks and ncOCRs are observed for a putative bowfin ortholog of the MCS-R2 hb enhancer in intron 5 of the *nprl3* gene known to regulate flanking hemoglobin genes^62^ (red). (**f**) Conserved synteny of the region containing hemoglobin genes (red highlighted arrows) on bowfin Aca scaf15 to gar, teleosts, and tetrapods, centered on the *nprl3* gene (central green arrow) that contains the putative MCS-R2 intronic hb enhancer. Source data

The bowfin genome was sequenced from a single adult phenotypic male. A de novo genome assembly was constructed with Meraculous^26^ and further scaffolded with Chicago^27^ and Hi-C approaches^28^ using the HiRise software pipeline^27^ (Supplementary Figs. 1 and 2). The final assembly (*N*_50_ = 41.2 Mb) consists of 1,958 scaffolds including 23 pseudochromosomes that contain 99% of the assembly (Extended Data Fig. 1 and Supplementary Table 1) and match bowfin’s chromosome number^21,29^, consistent with a chromosome-level genome assembly. The final assembly (AmiCal1, National Center for Biotechnology Information (NCBI) accession PESF00000000; Supplementary Tables 1 and 2) is largely complete as reported by multiple core eukaryotic genes mapping approach (CEGMA) and benchmarking universal single-copy orthologs (BUSCO) scores (Supplementary Table 3).

The bowfin genome consists of 22.1% repeats, very similar to that of spotted gar (22.8%). However, there are clear differences between bowfin and gar in the distribution of individual transposable element (TE) types (Supplementary Table 4) and the evolutionary history of repeat amplification. Bowfin shows a single peak of TE activity (Kimura distance of 4), while spotted gar has two older TE bursts (Kimura distances of 7–8 and 25 (ref. ^7^)) (Supplementary Fig. 3).

Using transcriptomic evidence from ten adult tissues^7,30^, we generated a MAKER^31^ genome annotation reporting 21,948 protein-coding genes, very similar to that for spotted gar (21,443 genes)^7^. OrthoFinder^32^ predicted orthologies for 86.6% of these genes to 11 other vertebrates (Supplementary Tables 5 and 6). See Supplementary Notes 1 and 2 for genome assembly and annotation details.

**Fig. 1:**
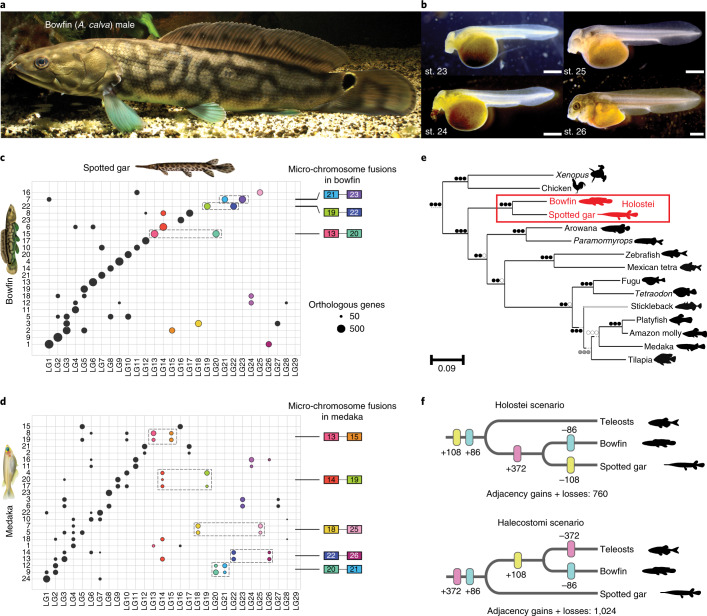
Bowfin and the evolution of neopterygian genome organization. **a**, Adult male bowfin. **b**, Bowfin stages (st.) 23–26 (ref. ^53^), covering critical phases of pectoral fin and gas bladder development (representative of *n* = 20 individuals per stage). Scale bar, 1 mm. **c**, Orthologies between bowfin and gar chromosomes and inferred bowfin micro-chromosome fusions for bowfin. **d**, Orthologies between medaka and gar chromosomes and inferred medaka micro-chromosome fusions. Circles, number of orthologous genes shared by bowfin and gar and/or medaka chromosomes (if in excess compared to random expectations). Ancestral micro-chromosomes are highlighted with colors; micro-chromosome fusions are indicated by dotted boxes. Colored boxes refer to the gar chromosome number. Micro-chromosome fusions differ between **c** and **d**: for example, medaka 9 and 12 result from a pre-TGD fusion of ancestral chromosomes orthologous to gar micro-chromosomes LG20 and LG21 and macro-chromosome LG2, followed by TGD duplication of the fusion chromosome (**d**). Bowfin 15 is a fusion of ancestral micro-chromosomes orthologous to gar LG13 and LG20; bowfin 7 is a fusion of ancestral chromosomes orthologous to gar LG1 and micro-chromosomes LG21 and LG23 (**c**). **e**, NJ phylogeny based on gene-order divergence built using a normalized breakpoint distance. Circles, bootstrap support for NJ, FastME and UPGMA analyses; black, 100%; gray ≥70%; white <70%. The Holostei clade is strongly supported (red box). Note that the location of the stickleback branch (gray) is in disagreement with the consensus phylogeny^4,8^ (but see ref. ^85^). **f**, Dollo parsimony applied to gains or losses of local gene adjacencies in Holostei (top) and Halecostomi (bottom) scenarios. Adjacencies shared by bowfin and gar only are in pink, those shared by bowfin and teleosts only are in yellow, and those shared by gar and teleosts only are in blue.

Despite pronounced sexual dimorphism in adult behavior and color patterning in bowfin (Fig. 1a, Supplementary Fig. 4a,b and Supplementary Note 3), its karyotype does not show any obvious cytogenetic differentiation of sex chromosomes^21,29^. Here, pool-sequencing (Pool-seq) strategies that contrast 30 mature phenotypic males with 30 mature phenotypic females using both reference genome-based and genome-free approaches did not reveal any genomic region exhibiting sex differentiation (Supplementary Fig. 4c–f, Supplementary Tables 7 and 8 and Supplementary Note 3), similar to previous findings for spotted gar^33^. Sex chromosomes and genetic sex-determination mechanisms thus remain elusive in holosteans if they exist.

### Genome organization and gene order support the monophyly of holostean fishes

**Extended Data Fig. 2:**
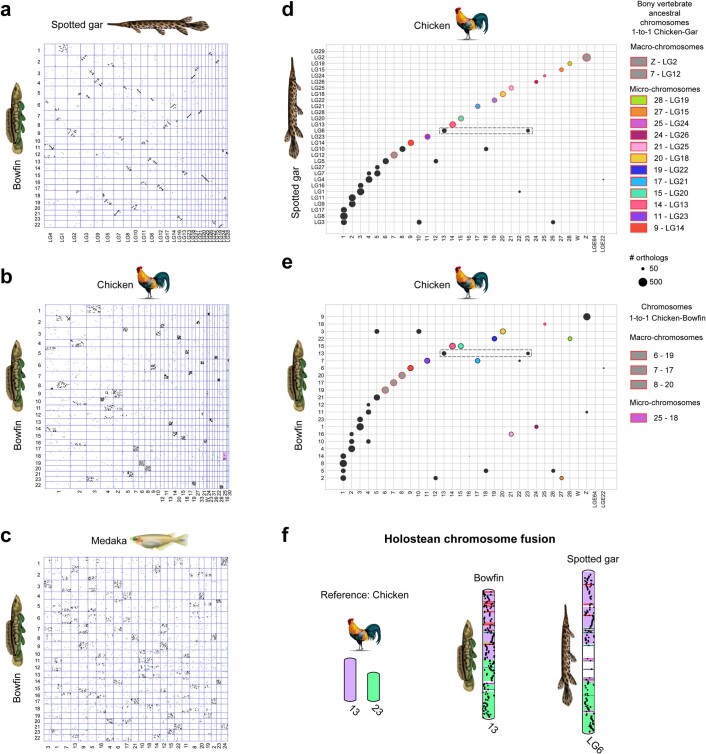
Conserved synteny analyses. Oxford grid comparisons of bowfin vs. gar (**a**), chicken (**b**), and medaka (**c**) genomes. (**d**) Orthologies between the bowfin and chicken chromosomes. (**e**) Orthologies between the gar and chicken chromosomes. Circles represent the number of orthologous genes shared between pairs of chromosomes (if in excess compared to random expectations). One-to-one orthologous chromosome pairs are circled in red and summarized on the right. The dotted rectangle highlights a potential shared fusion in bowfin and gar. (**f**) Putative holostean-specific chromosome fusion. Bowfin chromosome 13 and spotted gar LG6 are painted according to chicken chromosomes 13 and 23.

Cytogenetic comparisons suggest that the bowfin karyotype is more similar to those of teleosts than those of gars^21^. While gar and chicken retained micro-chromosomes from the bony vertebrate ancestor^7,34,35^, karyotype analyses provide no evidence for micro-chromosomes in bowfin^21,29^. To the best of our knowledge, true micro-chromosomes have also not been discovered in teleosts. This shared absence of micro-chromosomes could imply a closer relationship of bowfin and teleosts (Halecostomi) opposed to the monophyly of bowfin and gars (Holostei). We previously showed that the teleost ancestor experienced micro-chromosome fusions after its divergence from gar but before the TGD^7^. We thus investigated whether these teleost micro-chromosome fusions were shared with bowfin (Fig. 1 and Extended Data Fig. 2a–c). By orthology to gar micro-chromosomes conserved from the bony vertebrate ancestor^7^, we identified bowfin and teleost chromosomes corresponding to fused ancestral micro-chromosomes. We found three such micro-chromosome fusions for bowfin and five for the teleost medaka, with none shared between them (Fig. 1c,d). These fusions were also not shared with micro-chromosome fusions in the derived genome of bichir (Supplementary Fig. 5), representing the most basally diverging extant ray-finned fish lineage^12^. We conclude that bowfin, teleosts and bichir independently fused different sets of micro-chromosomes. The karyotypic similarities of bowfin and teleosts are the result of convergent evolution rather than due to a common origin and thus do not support the Halecostomi scenario.

We identified four chromosome pairs with one-to-one orthology between bowfin and chicken, while there are 14 one-to-one pairs between gar and chicken^7^ (Extended Data Fig. 2d,e). Thus, the bowfin karyotype is more derived than that of gar. However, in support of holostean monophyly, chicken chromosomes 13 and 23 correspond to a fusion chromosome in both bowfin and gar (Extended Data Fig. 2f), a rearrangement not found in teleosts or bichir (Supplementary Fig. 5).

We investigated gene-order evolution as a new line of evidence to illuminate bowfin’s phylogenetic position. Genomic rearrangements (inversions, transpositions, translocations) introduce gene-order differences across species over evolutionary time but are much rarer than nucleotide substitutions and therefore useful for reconstructing phylogenies^36^. Using rearrangement distances, we reconstructed a phylogeny of bowfin, gar and ten teleosts, with the addition of two tetrapod outgroups, and leveraged all 3,223 marker genes that are present in exactly one copy in non-teleost genomes and in one or two copies in TGD-derived teleost genomes. We then estimated pairwise evolutionary distances between species using a normalized breakpoint distance (Supplementary Fig. 6) and constructed rearrangement-based neighbor-joining (NJ), minimum evolution and unweighted pair group method with arithmetic mean (UPGMA) species trees that all support holostean monophyly with high confidence (Fig. 1e). Focusing on all gene adjacencies acquired since the neopterygian ancestor (that is, gene adjacencies found in gar, bowfin and/or teleosts but not in outgroups), we positioned gains and losses of these adjacencies on the two alternative phylogenies using the Dollo algorithm, assuming that a gene adjacency is gained only once but can be lost numerous times independently^37^. The Holostei scenario required a significantly lower number of evolutionary changes (760 versus 1,024; Kishino-Hasegawa (KH) test, *P* value = 0.028), rejecting the Halecostomi scenario (Fig. 1f).

Phylogenomic analyses of OrthoFinder^32^-generated protein alignments from 2,079 single-copy genes in 12 vertebrate species using maximum-likelihood and Bayesian approaches, as well a species tree generated from 7,532 OrthoFinder gene trees using STAG^38^, provide additional, strong evidence for holostean monophyly (Supplementary Note 4 and Supplementary Fig. 7). Thus, gene order, in agreement with our sequence-based analyses and published phylogenomic studies using coding and non-coding markers^4–8,12^, strongly supports holostean monophyly regardless of methodology.

While the bowfin karyotype is more derived than that of gar at a gross chromosomal level, this is not reflected in local gene order, for which bowfin and gar present similar levels of species-specific rearrangements rates (Supplementary Note 5). In agreement with the slow rate of genomic sequence evolution in holosteans^7,16^, gene-order rearrangement rates in bowfin are significantly lower than those in teleosts (Supplementary Note 5).

### Characterizing the holostean immunogenome

Our previous analyses of spotted gar immune genes revealed shared characteristics with those of both teleosts and tetrapods but left important aspects of the ray-finned fish immunogenome unresolved^7,39^. For a comprehensive understanding of holostean immune genes, we surveyed the bowfin genome and a newly generated bowfin immune tissue transcriptome (Supplementary Note 6).

**Fig. 2:**
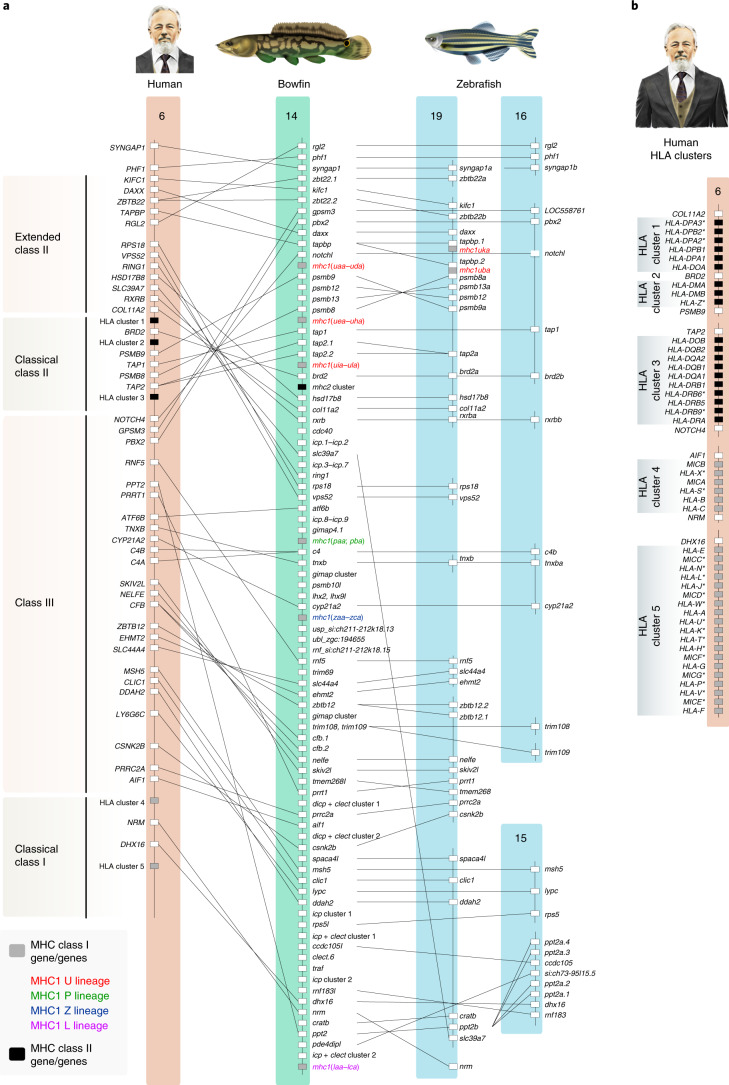
The bowfin MHC. **a**, The bowfin MHC on pseudochromosome 14 contains class I, class II and class III genes with orthologous relationships to the human MHC on chromosome 6 and zebrafish chromosomes 19, 16 and 15. Boxes represent genes or gene clusters of related sequences; gene placement is not to scale. Only those genes with bowfin orthologs are shown in human and zebrafish. Pseudogenes of non-MHC genes and RNA-coding genes are not included. **b**, Detailed lists of genes within each human leukocyte antigen (HLA) gene cluster in human (for example, William W. Ballard) indicated in **a** are provided. Asterisks indicate MHC or MHC-related pseudogenes. See also Supplementary Table 9.

The human major histocompatibility complex (MHC) is a cluster of >200 genes, broadly characterized as class I, II and III genes, the products of which play various roles in antigen processing, antigen presentation and inflammation^40^. Class I and class II genes are tightly linked on one chromosome in cartilaginous fishes and tetrapods (for example, on human chromosome 6, Fig. 2)^41^. By contrast, teleost class I and class II genes are not linked, and class III genes are scattered throughout teleost genomes^42^. The ancestral organization of the ray-finned MHC has remained unresolved because the gar MHC is highly fragmented in the reference assembly^7^. The bowfin, by contrast, has a cluster on pseudochromosome 14 that contains the majority of class I, II and III genes (Fig. 2, Supplementary Figs. 8 and 9, Supplementary Tables 9–11 and Supplementary Note 6) and that is not the result of a recent chromosome fusion (Fig. 1c and Supplementary Fig. 8). Thus, the overall organization of MHC regions in holostean and ray-finned fish ancestors was similar to that in tetrapods and cartilaginous fishes. Furthermore, the loss of linkage of teleost class I, II and III genes therefore occurred after divergence from holosteans and is associated with differential gene loss from TGD-duplicated chromosomes, exemplified by MHC regions on zebrafish chromosomes 19 and 16 (Fig. 2 and Supplementary Fig. 8).

**Extended Data Fig. 3:**
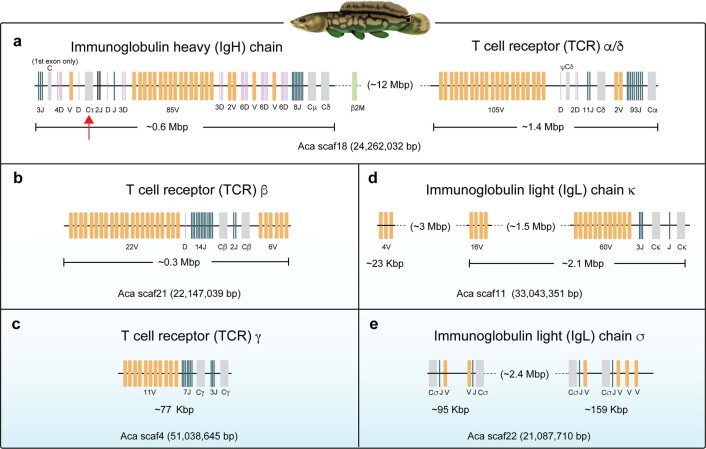
Genomic organization of bowfin immunoglobulin and TCR genes. (**a**) The immunoglobulin heavy (IgH) chain and T cell receptor (TCR) α/δ loci are separated by 12 Mb on pseudochromosome 18 (Aca scaf18). The IgH locus encodes 3 classes of Ig constant domains, Cτ, Cμ and Cδ. The red arrow indicates Cτ, which was previously thought to be teleost-specific. Note that the IgL sigma-2 locus is present in the middle of V gene segment cluster of the IgH locus (not shown). (**b**) The TCRβ locus is encoded on Aca scaf21. (**c**) The TCRγ locus is located on Aca scaf4. (**d**) The Ig light (IgL) chain kappa locus is present on Aca scaf11. (**e**) The IgL chain sigma loci are encoded on Aca scaf22. Multiple genes (including pseudogenes) other than Ig and TCR are present in these pseudochromosomes, but not shown. V domains are shaded orange, D segments purple, J segments blue, and constant (C) domains gray. Source data

Antigen-recognition receptors of the adaptive immune system, that is, immunoglobulin (Ig) and T cell receptor (TCR), have been identified in all jawed vertebrate lineages but can differ in their genomic structure and organization^43^. The bowfin genome contains all three canonical TCR loci and, remarkably, encodes not only antibody classes IgM and IgD but also IgT (IgZ in zebrafish) (Extended Data Fig. 3, Supplementary Figs. 10–12, Supplementary Tables 12–14 and Supplementary Note 6), which has been considered teleost specific^44^. We then used the bowfin gene encoding IgT, which is confirmed by transcriptomic analyses^45^, to identify a previously unknown^7^ IgT ortholog in spotted gar. Thus, IgT-like antibodies date back to the neopterygian ancestor (Supplementary Note 6)^45^.

Toll-like receptors (TLRs) provide initial immune responses to infection^46^. The bowfin possesses 20 TLR genes, twice the number of human TLR genes^47^, more than the 16 functional spotted gar TLR genes^7,39^ and almost on par with the 20+ teleost TLR genes^48^ (Supplementary Fig. 13, Supplementary Table 15 and Supplementary Note 6). TLR complexity is therefore a more general pattern among neopterygians, and the TGD is not the sole evolutionary mechanism leading to large TLR gene numbers in ray-finned fishes. In summary, the holostean immunogenome shows an overall complexity and diversity comparable to those of teleosts, with a chromosomal organization resembling tetrapods and more distant vertebrate lineages.

### Biomineralization genes and the formation of ray-finned fish scales

While bowfin and gar both possess enamel-covered teeth, they prominently differ in scale biomineralization. Gars have ganoid scales, representing an ancestral actinopterygian scale type covered with a thick layer of hypermineralized ganoin. The bowfin, by contrast, has thin, flexible elasmoid scales that secondarily lost the thick bony plate and ganoin^17,23^. Teleosts have neither tooth enamel nor scale ganoin. It is thus expected that genes involved in scale formation differ among neopterygians.

**Fig. 3:**
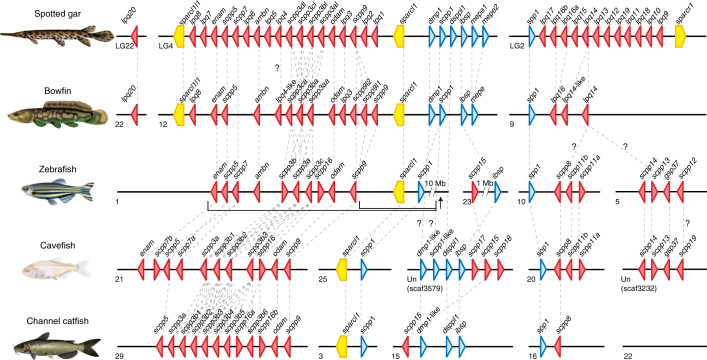
Organization of biomineralization genes in neopterygian fishes. Genomic arrangements of SCPP genes and secreted protein acidic and rich in cysteine (SPARC)-family genes in the genomes of spotted gar, bowfin and the teleosts zebrafish, Mexican tetra (cavefish) and channel catfish. The direction of triangles and pentagons indicates the transcriptional direction of each gene, encoding P/Q-rich SCPPs (red), acidic SCPPs (blue) and SPARC-family proteins (yellow). Orthologs are connected by dashed lines; unresolved orthologies are indicated with a ‘?’. Intervening non-SCPP genes are not shown. In the zebrafish genome, a cluster of *sparcl1* and *scpp1* is separated by ~10 Mb from the other genes encoding P/Q-rich SCPPs (arrow). Two cavefish SCPP gene clusters are located on unplaced scaffolds. See Supplementary Data 1 and Source Data for SCPP gene predictions, locations and gene IDs. Source data

The secretory calcium-binding phosphoprotein (SCPP) gene family, generated by complex successive gene duplications during bony vertebrate evolution, encodes proteins involved in biomineralization^49^. We identified 22 SCPP genes in bowfin, 21 of which form two large genomic clusters arranged similarly to those in gar (Fig. 3, Supplementary Data 1 and Supplementary Note 7). Similar to gar^7,50,51^, the bowfin possesses ganoin-forming SCPP genes *enamelin* (*enam*), *ameloblastin* (*ambn*) and *scpp5* (Fig. 3). Their involvement in the formation of both tooth enamel and scale ganoin in gar supports the idea that these two mineralized structures evolved from a common genetic program^7,50,51^. As bowfin lacks ganoin, this suggests that gene-regulatory shifts in *enam*, *ambn* and *scpp5* have occurred in the bowfin lineage that silenced their expression during scale development but not dental enamel formation.

The total number of bowfin SCPP genes (22) is considerably smaller than that of gar, which has the largest known SCPP gene repertoire among vertebrates (38)^7,49^. All 22 bowfin SCPP genes have a gar ortholog. By contrast, orthologs of 16 gar SCPP genes could not be found in bowfin, nine of which are located in a cluster on gar chromosome LG2 syntenic to a reduced gene cluster on bowfin pseudochromosome 9 (Fig. 3). Expression of most gar LG2 SCPP genes was weak or undetectable in tooth germs but strong in scale-forming skin^7^. This implies that the SCPP clusters on gar LG2 and bowfin pseudochromosome 9 are particularly involved in scale formation, a hypothesis further supported by teleost SCPP genes (Supplementary Fig. 14 and Supplementary Note 7). The orthologous zebrafish SCPP genes are located on TGD-duplicated clusters on chromosomes 5 and 10 (ref. ^7^) and are expressed during zebrafish skin development (Supplementary Table 16 and Supplementary Note 7). Furthermore, these clusters have been reduced to only one gene (*scpp8*) in the scaleless channel catfish (Fig. 3). They are also highly reduced in scale-reduced sturgeon and paddlefish^13^ (Supplementary Note 7). These multiple lines of evidence suggest that SCPP genes with major roles in scale formation are clustered in a genomic region that is expanded in the ganoid gar on LG2 and likely secondarily reduced in bowfin on pseudochromosome 9, corresponding to the formation of their modified elasmoid scales. We thus hypothesize that reduced biomineralization of the bowfin scale is attributed to both changes in gene regulation and the loss of specific SCPP genes.

### Chromatin profiling through bowfin development connects gene regulation across vertebrate morphologies

**Fig. 4:**
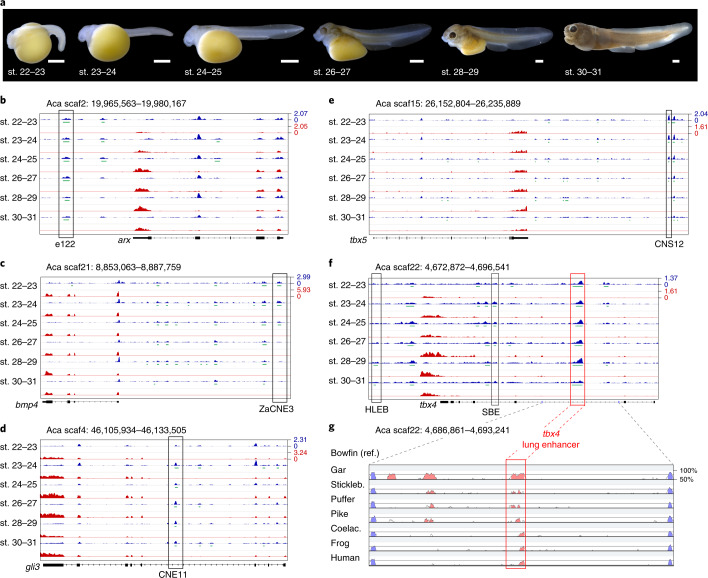
Chromatin and gene expression profiles during bowfin development. **a**, Developmental-stage series of bowfin^53^ (representative of *n* = 5 individuals per stage). Scale bars, 1 mm. **b**–**f**, ncOCRs overlapping with key developmental enhancers are indicated by black boxes. ATAC-seq profiles are in blue, MACS2-called OCRs are in green, mRNA-seq profiles are in red, and gene annotations are in black. ATAC-seq and RNA-seq data ranges are indicated for each panel. **b**, Forebrain enhancer e122 (ref. ^56^) downstream of *arx* is consistently open. Aca, *A. calva*. **c**, Pan-cardiac heart enhancer ZaCNE3, conserved from tetrapods to fish^57^ and upstream of *bmp4*, transitions from open to closed chromatin as development progresses. **d**, Intragenic *gli3* limb enhancer CNE11 (ref. ^58^), connected by sequence orthology from tetrapods to teleosts through gar^7^ and bowfin, opens at stage 23 when pectoral fins are first visible^53^ and remains open during pectoral fin development. **e**, Pectoral fin *tbx5* enhancer CNS12 (ref. ^59^) is open during stages 22–27 when pectoral fins develop. **f**, The *tbx4* hindlimb enhancer HLEB^60^ is an ncOCR at stages 28–30, shortly after the onset of pelvic fin development in stage 27 (ref. ^53^). Another ncOCR is located on the *tbx4* ‘lung enhancer’, critical for lung development in mouse^60,61^, that is open throughout bowfin gas bladder budding and outgrowth stages (stages 24–27) when *tbx4* is expressed in the bowfin gas bladder^24^. A putative swim bladder enhancer (SBE)^86^, conserved with teleosts (Extended Data Fig. 4), is also marked by open chromatin. **g**, VISTA-SLAGAN^87,88^ conservation plot with bowfin as reference (ref.) shows sequence conservation of the *tbx4* lung enhancer from tetrapods to lobe-finned fish (coelacanth (coelac.)) and teleosts (stickleback (stickleb.), pufferfish, pike), demonstrating the power of the ‘holostean bridge’ to reveal deep homology of gene regulation of key vertebrate morphologies (Extended Data Fig. 4 and Supplementary Note 9).

To increase the power and utility of the holostean genome for comparative studies on vertebrate gene regulation, we used the assay for transposase-accessible chromatin with sequencing (ATAC-seq)^52^ to generate an atlas of open chromatin regions (OCRs) from seven developmental stages of wild-caught bowfin embryos and larvae, from before the conserved phylotypic stage to the end of the described larval development^53^ (Supplementary Fig. 15 and Supplementary Note 8). We identified a total of 172,276 OCRs, of which 81.8% (140,902) were non-coding OCRs (ncOCRs) and annotated their genomic feature location and nearest gene with HOMER^54^ (Supplementary Tables 17–19). About 70% of OCRs and ncOCRs were identified in at least two stages; 33,239 OCRs and 21,636 ncOCRs were found in all seven stages (Supplementary Tables 17 and 18). Using whole-genome alignments (WGAs) generated with Progressive Cactus^55^, we showed that more than 50% of bowfin ncOCRs were conserved in gar, and 3,844 core bowfin ncOCRs were conserved in gar, zebrafish, mouse and human (Supplementary Fig. 16 and Supplementary Table 20). Adding RNA-seq transcriptomes from the same stages, our dataset provides a rich resource for exploring the correlation between chromatin accessibility of putative gene-regulatory elements and gene expression. This is exemplified by bowfin ncOCRs overlapping key enhancers active during the development of a variety of vertebrate systems such as the brain^56^, heart^57^, anterior and posterior fin or limb^58–60^ and lungs^60,61^ (Fig. 4). Using Hi-C contact maps from bowfin blood cells, we further identify a bowfin ncOCR at the location of an evolutionary conserved hemoglobin gene enhancer^62^, embedded within a topologically associating domain (TAD) that shows conserved synteny (Extended Data Fig. 1c–f) with tetrapod hemoglobin-region TADs^63,64^.

Evolutionary conserved elements such as conserved non-coding elements (CNEs) and ultraconserved elements (UCEs) (as defined in the Methods) are used to identify candidate enhancers and genetic loci for phylogenetic inference^6,7^. With our WGAs, we found that 31.9% of both gar-centric vertebrate CNEs^7^ (21,127 of 66,182) and 27.5% of bowfin UCEs^6^ (100 of 364) intersected with bowfin ncOCRs (Supplementary Table 21); thus, many CNEs or UCEs are likely active gene-regulatory elements during bowfin development, some of which are specific to different fish clades (Supplementary Table 21 and Supplementary Note 8). Overlap with bowfin ncOCR increases with CNE age, suggesting that ancestry of sequence conservation is a predictor of chromatin accessibility during bowfin development (Supplementary Table 21 and Supplementary Note 8). To further investigate the comparative efficacy of our ATAC-seq dataset, we used our WGAs to identify bowfin orthologs of experimentally confirmed mammalian VISTA enhancers^56^ (Supplementary Tables 22 and 23 and Supplementary Note 8). Orthologs of 60.2% of human VISTA enhancers (600 of 996) were found in bowfin, and over half (56.3%, 338 of 600) overlapped with bowfin ncOCRs. By contrast, orthology for only 44.0% (449 of 996) of human enhancers could be established in the zebrafish genome (Supplementary Table 22), illustrating the usefulness of the slowly evolving and ‘unduplicated’ bowfin genome to connect accessible, non-coding, regulatory regions from human to fish. Overlap with 2,261 ncOCRs from a mouse developmental single-nucleus ATAC-seq atlas^65^ (22.5% of 10,035 mouse ncOCRs with bowfin orthology) established bowfin ncOCRs that likely function in a cell and/or tissue type-specific manner, for example, in the central nervous system, mesoderm, neural crest and many other cell types and tissues (Supplementary Table 24).

Currently, beyond zebrafish and medaka (for example, refs. ^66,67^), there are limited gene-regulatory resources for fishes, rendering comparative analyses across species or whole-genome duplications difficult. Our bowfin ATAC-seq atlas provides a key platform to further connect gene-regulatory homology across vertebrates while strengthening the utility of non-teleost fishes for examining genome function in the context of long-standing questions about vertebrate evolution and development.

### Deep homology of vertebrate air-filled organs at the gene-regulatory level

**Extended Data Fig. 4:**
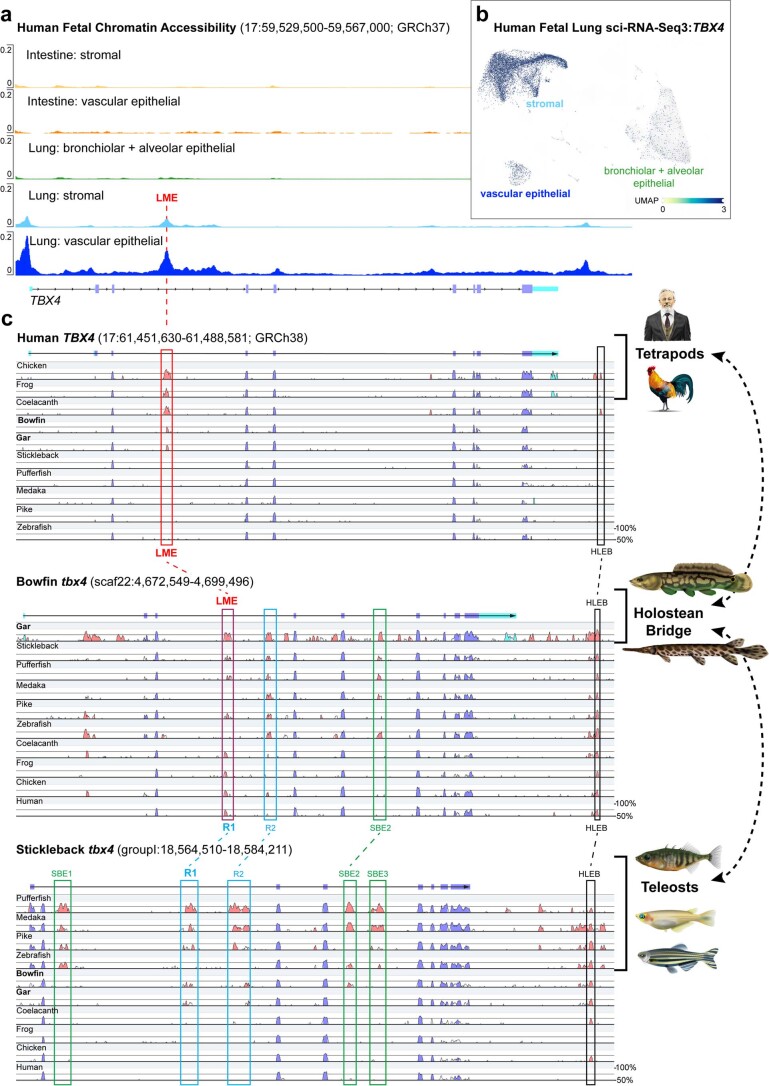
The holostean bridge connects vertebrate *Tbx4* gene enhancers. (**a**) Human fetal *TBX4* chromatin accessibility shows a peak at the ‘lung mesenchyme enhancer’ (LME) in lung cells. (**b**) Human *TBX4* single cell expression in fetal lung. See Supplementary Note 9 for details. (**c**) VISTA SLAGAN alignments with human (top), bowfin (middle), and stickleback (bottom) as reference. *Top:* Human *TBX4* intron 3 ‘lung mesenchyme enhancer’ (LME, red box), driving expression in the developing mammalian lung bud^60,61^, shows conservation with other tetrapods, coelacanth, bowfin, and gar (and also with bichir^12,126^ and some sharks^127^, not shown), but no conservation with teleosts. HLEB: *TBX4* hind limb enhancer B^60^ (black box). *Bottom:* The stickleback *tbx4* gene contains three putative ‘swimbladder enhancers’^86^ (SBE1-3, green boxes). SBE2 is conserved with bowfin but not lobe-finned vertebrates. Intron 3 regions R1 and R2 (blue boxes) appear as conserved among neopterygians (teleosts, bowfin, gar), but not with coelacanth or tetrapods. HLEB (black box) is also present. *Middle:* The bowfin *tbx4* gene identifies different conserved regions defined in the human- and stickleback-centric alignments (top, bottom): SBE2 (green box), R2, and HLEB. Lobe-finned LME and neopterygian R1 elements overlap within the bowfin genome, and are thus orthologous conserved non-coding elements, connected via the holosteans bridge (right). This bowfin LME/R1 region is also a bowfin developmental ncOCR (main text Fig. 4f, where it is shown on the reverse strand).

Ever since Owen and Darwin, the homology of vertebrate air-filled organs has been debated^68^ and is supported by adult gene expression similarities among various fish air bladders and tetrapod terrestrial lungs (for example, refs. ^12,69,70^). Our previous genetic developmental investigations^24,25^ in bowfin identified several key developmental genes for respiratory gas bladder development including the *tbx4* transcription factor gene. Using our ATAC-seq atlas, we identified an intronic ncOCR within the bowfin *tbx4* gene (Fig. 4f) that is orthologous to a *tbx4* ‘lung enhancer’ important for embryonic lung budding in mammals^60,61^ (Extended Data Fig. 4a,b and Supplementary Note 9). Furthermore, we showed sequence conservation with a previously uncharacterized, conserved intronic region in teleost fishes (Fig. 4g). Thus, the ‘holostean bridge’ establishes orthology of this important developmental enhancer from tetrapods to teleosts (Fig. 4g, Extended Data Fig. 4c and Supplementary Note 9). Importantly, the chromatin accessibility of the ‘lung enhancer’ activity in bowfin correlates with critical timing of *tbx4* expression during gas bladder development^24^ (Fig. 4f and Supplementary Note 8). These results support deep homology among bony vertebrate air-filled organs, that is, terrestrial lungs, holostean respiratory gas bladders and buoyancy-controlling swim bladders in teleosts (Supplementary Note 9) at the developmental, gene-regulatory level.

### Evolutionary stasis of holostean Hox clusters and the enigmatic *hoxD14* gene

**Fig. 5:**
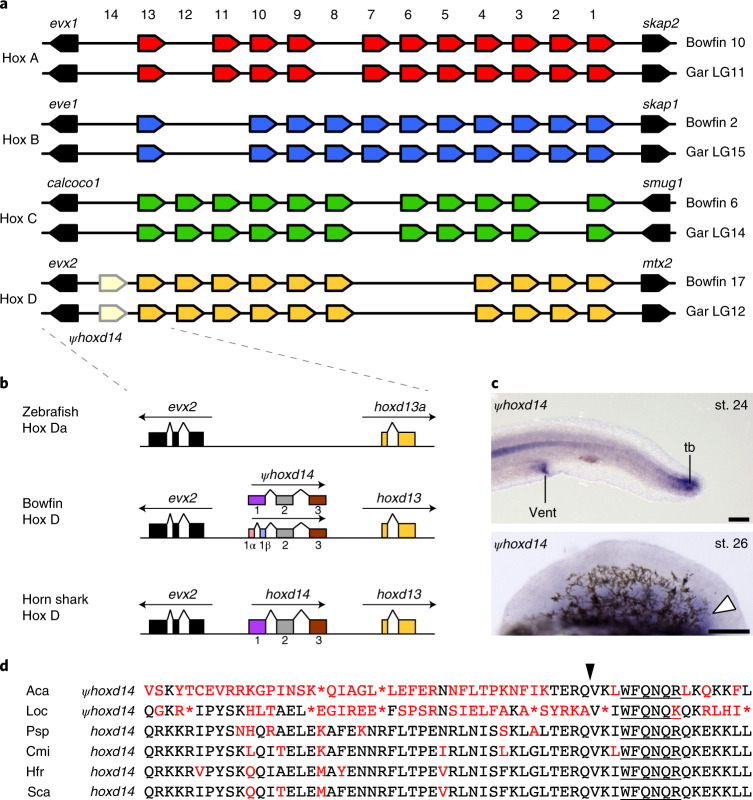
Holostean Hox gene clusters and the bowfin *hoxd14* pseudogene. **a**, Hox cluster organization in bowfin and spotted gar genomes. **b**, Relative position and orientation of genes encoding Hox14 paralogs in the Hox D cluster between *evx2* and *hoxd13* in zebrafish, bowfin and horn shark. We identified two alternative splice variants in bowfin. **c**, Whole-mount RNA in situ hybridization of *hoxd14* pseudogene expression in the tailbud (tb) and vent at stage 24 and in the posterior pectoral fin mesenchyme at stage 26 (white arrowhead), observed consistently across *n* = 10 samples. Scale bars are 125 µm. **d**, Multiple-sequence alignment of the HoxD14 homeodomain across species including bowfin (Aca), spotted gar (Loc), paddlefish (Psp, *Polyodon spathula*), elephant shark (Cmi, *Callorhinchus milii*), horn shark (Hfr, *Heterodontus francisci*) and small-spotted catshark (Sca, *Scyliorhinus canicula*). The black arrowhead indicates the conserved intra-domain splicing position, and underlining indicates the conserved WFQNQR motif.

Hox cluster genes play essential roles in patterning the primary body axis during animal development as well the proximo–distal and anterior–posterior axes of vertebrate appendages. As a result of the two early rounds of vertebrate genome duplication, non-teleost ray-finned fishes without additional genome duplications such as gar contain four Hox clusters, Hox A–D^7^. Highlighting the evolutionary stasis of the holostean lineage, the bowfin genome harbors the same repertoire of 43 bona fide Hox cluster genes as spotted gar (Fig. 5a, Supplementary Data 2 and Supplementary Note 10), with only one gene lost since the bony vertebrate ancestor and none lost since the ray-finned and holostean fish ancestors^7,11^.

Evolution of the vertebrate Hox14 paralog group exhibits unique patterns of conservation, loss and pseudogenization. Members of this paralogy group are absent from tetrapods and teleosts but present in lamprey, cartilaginous fishes, paddlefish, sturgeon and gar^7,11,71–73^. Gar *hoxd14* is a pseudogene without evidence for transcription^7^ (Supplementary Note 10). We found sequence conservation of gar *hoxd14* with the bowfin genome within the intergenic region between *evx2* and *hoxd13*. A large number of bowfin pectoral fin bud RNA-seq reads (see below) aligned to this region, which also showed open chromatin status (Supplementary Fig. 17). We cloned two different *hoxd14* transcripts from bowfin pectoral fin bud cDNA (Fig. 5b). RNA in situ hybridization revealed that bowfin *hoxd14* is expressed in stage 26 pectoral fin buds in a posterior mesenchymal domain (Fig. 5c), similar to expression in paddlefish^73^. Expression was also observed in the vent, similar to that in shark and lamprey^71^, and in the tailbud (Fig. 5c). Bowfin *hoxd14* is likely a pseudogene, with numerous stop codons and nonsynonymous changes throughout the coding region and homeodomain (Fig. 5d). The hallmark Hox14 WFQNQR motif is preserved, however, as is the split homeodomain junction^72^.

Initially, *hox14* genes were thought not to be subject to ancestral regulatory mechanisms that control *hox1*–*hox13* (ref. ^71^). However, paddlefish fin buds express *hoxd14* posteriorly, consistent with spatially collinear Hox regulation^73^. The bowfin now supports temporal collinearity of *hoxd14* expression (see below), and expression in its tailbud reveals another domain exhibiting global collinear regulation. The bowfin *hoxd14* pseudogene is still transcribed at high levels, spliced and expressed in highly specific, ancestral patterns. This discovery raises the possibility that, even though bowfin *hoxd14* likely no longer codes for a functional protein, it may have some function in the regulation of *hox* expression and fin patterning and/or other to-be-identified processes.

### Unexpected gene expression dynamics during holostean pectoral fin development

**Extended Data Fig. 5:**
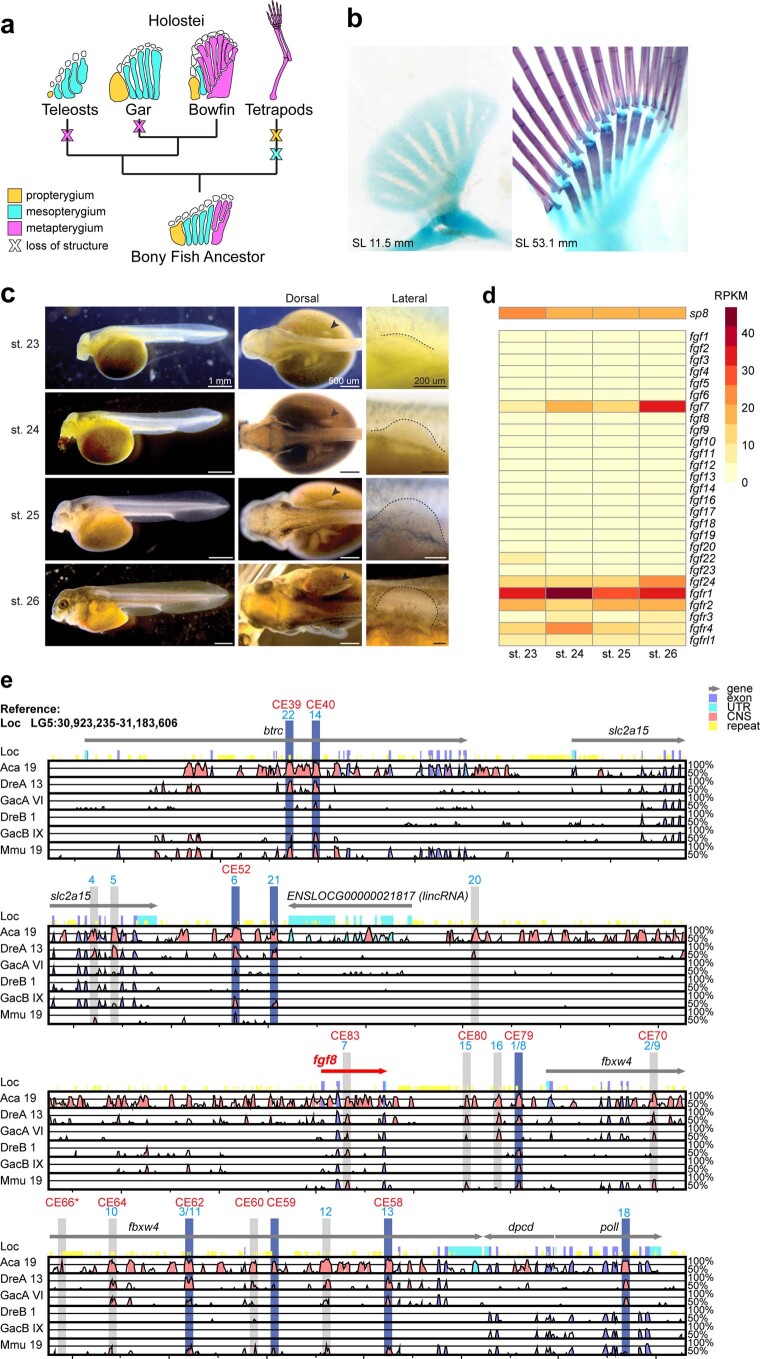
Bowfin pectoral fin Evo-Devo. (**a**) Evolution of the pectoral endoskeleton in bony fishes. Elements are colored based on which portion of the fin they comprise. From the tribasal ancestral condition, teleosts and gars independently lost the metapterygium, while tetrapods lost the propterygium and mesopterygium. Bowfin is the closest living relative of the teleosts that retains the metapterygium. (**b**) Representative bowfin pectoral fins at two different developmental stages cleared and stained with alcian blue for cartilage and alizarin red for bone. Anterior to left, distal to top in all panels. Sample sizes: *n* = 10 juveniles 10-13 mm (left); *n* = 1 juvenile 51.3 mm (right). (**c**) Bowfin developmental stages^53^ sampled for RNA-seq transcriptomics (representative of *n* = 20 individuals per stage). Arrow in dorsal view (middle column) indicates developing pectoral fin, dotted line in lateral view (right column) indicates outline of the developing pectoral fin bud. (**d**) Pectoral fin RNA-Seq of *sp8* and Fgf signaling genes. Expression levels in Reads Per Kilobase Million (RPKM). (**e**) Genomic alignment of bony vertebrate *fgf8* regions. mVISTA plot showing sequence conservation of bowfin (Aca), zebrafish (Dre; *fgf8a* and *fgf8b* paralogons), stickleback (Gac; *fgf8a* and *fgf8b* paralogons), and mouse (Mmu) vs. spotted gar (Loc) as reference. Shaded areas highlight known regulatory elements for zebrafish (blue numbers)^128^ and mouse (red CE numbers)^129^; blue shade indicates bowfin ncOCRs (Supplementary Table 26). All relevant elements, including those important for expression in the apical ectodermal ridge of the mouse limb^130^, that is, CE58, CE59, CE66, CE80, except for CE61 (also absent from zebrafish), are found in gar and bowfin. CE66 (asterisk) was identified by sequence similarity to human. Source data

Given their predominance among neopterygians, teleosts have long been used as models for paired fin development and as outgroups for tetrapod limb development. However, the teleost pectoral fin endoskeleton has undergone severe reduction since the ancestral bony vertebrate, having lost the metapterygium and proximal–distal long bone articulation. Bowfin is the closest living teleost relative that retains the metapterygium and proximal–distal elaboration of the fin endoskeleton (Extended Data Fig. 5a,b) and thus serves as an essential node connecting appendicular skeletal diversity of ray-finned fishes and those of lobe-finned vertebrates, including tetrapods.

**Fig. 6:**
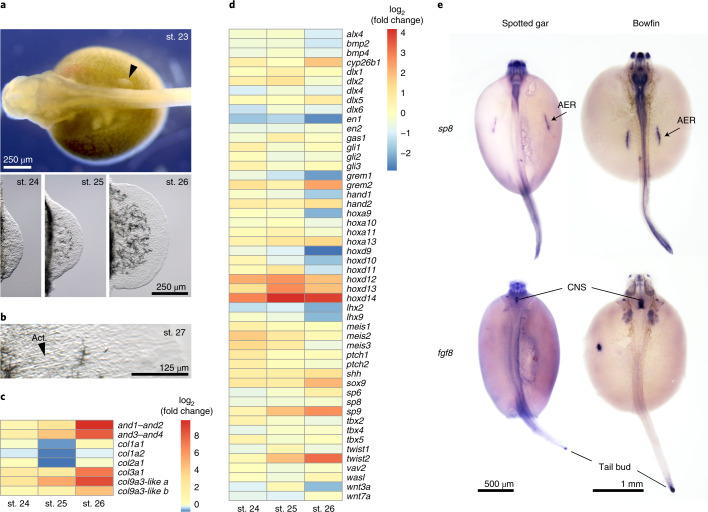
Gene expression analyses of developing pectoral fins in bowfin. **a**, Bowfin developmental stages^53^ used for RNA-seq transcriptome analysis. The fin bud is first visible at stage 23 (black arrowhead, anterior to left). Isolated fin buds showing increasing size from stages 24 to 25 to 26 (anterior to top, distal to right). **b**, Actinotrichia (Act.) fibrils first appear at stage 27. Pictures in **a**,**b** are representative of *n* = 5 individuals per stage. **c**, Transcriptional log_2_ (fold change) in fin fold structural genes relative to that at stage 23. Between stages 23 and 26, *and1*–*and2* and *and3*–*and4* show 862- and 285-fold increases, respectively; *col3a1*, *col9a3-like a* and *col9a3-like b* exhibit 133-, 417- and 19-fold increases. Stable expression across stages is observed for *col1a1*, *col1a2* and *col2a1*, each showing a fold-change range between 0.5 and 1.6. **d**, Transcriptional log_2_ (fold change) in expression of fin or limb patterning genes relative to that of stage 23 fin buds. Indicative of their temporal collinearity, expression of *hoxa9*, *hoxa10*, *hoxd9* and *hoxd10* peaks at stage 23, while expression of the more posterior *hoxa11*, *hoxa13*, *hoxd11*, *hoxd12* and *hoxd13* peaks at stage 25. Expression of *hoxa13* is highest at stage 26. The highest expression level of *hoxd14* is at stages 25 and 26. **e**, The transcription factor gene *sp8* is expressed in the AER of gar (stage 26) and bowfin (stage 23), while *fgf8* expression is not detected in the AER of either holostean (*n* = 10 samples per gene and species). CNS, central nervous system. Source data

We performed RNA-seq across four early patterning stages of bowfin pectoral fin bud development (Figs. 1b and 6a, Extended Data Fig. 5c, Supplementary Table 25 and Supplementary Note 11), revealing dynamic expression of many known fin or limb patterning genes. The most dramatic expression change in bowfin was in the transcription of structural genes of actinodin and collagen encoding components of the actinotrichia, elastinoid fibrils supporting the early fin fold^74^ (Fig. 6b). The expression of actinodin genes spikes at stage 26, as does that of type III and type IX collagen components (Fig. 6c). By contrast, *col1a1*, *col1a2* and *col2a1* exhibit relatively stable expression across the bowfin stages examined (Fig. 6c) but exhibit dynamic expression and comprise the main structural component of actinotrichia during zebrafish fin development^75^. Genes encoding type III collagens, absent from teleosts genomes^75^, have not been previously implicated in actinotrichia development and may underlie differences between bowfin and teleost fin morphology.

Given the distinct variation in patterning of the endochondral component between holosteans and teleosts, we focused on changes observed in early patterning genes during bowfin development. Temporal and spatial collinearity of Hox cluster gene expression plays critical roles in the patterning of fin and limb skeletons. We found collinearity in bowfin *hox* gene expression levels across early fin bud outgrowth. Peak expression of posterior *hox* genes tracked with their position within the Hox cluster, including that of *hoxd14* with highest expression levels at stages 25 and 26 (Fig. 6d). Other gene expression patterns were consistent with mechanisms described in limbs. Termination of tetrapod limb bud outgrowth is caused by the transcription factor Twist2, which negatively regulates *grem1* expression^76^. Similarly, bowfin *twist2* expression peaked at stage 26, while that of *grem1* peaked at stage 23 and was then downregulated approximately threefold by stage 26 (Fig. 6d). Other correlated expression profiles in bowfin are consistent with known genetic interactions, such as negative regulation of *meis* genes by cytochrome Cyp26b1 (ref. ^77^) (Fig. 6d).

Surprisingly, we noticed a lack of expression of the critical limb-development signaling ligand gene *fgf8* from the bowfin pectoral fin transcriptome at all stages. By contrast, other fin or limb apical ectodermal ridge (AER) markers are highly expressed across all stages, such as *sp8* (Extended Data Fig. 5d), which encodes a transcription factor that directly positively regulates *fgf8* expression in the AER^78^. Using RNA in situ hybridization, we found expected *sp8* and *fgf8* expression in the central nervous system and tailbud in both bowfin and gar embryos (Fig. 6e). In both holosteans, *sp8* was highly expressed in the AER throughout early fin bud development, while *fgf8* expression was not detected in the AER (Fig. 6e). These findings are surprising, as *fgf8* is expressed in every fin and limb bud in which it has been assessed across gnathostome lineages (for example, refs. ^79–82^) and is critical for the outgrowth and patterning of these appendages. Even in species that lack a morphological AER, *fgf8* is still expressed (for example, refs. ^83,84^). Thus, the lack of *fgf8* expression in the holostean fin bud is entirely unexpected. Given that *sp8* was expressed in the AER at high levels (Extended Data Fig. 5d), we hypothesize that changes have occurred in the regulation of *fgf8* that remove Sp8 responsiveness in the fin bud, a unique holostean feature. Genomic comparisons (Supplementary Note 11) showed that holosteans possess a complex set of known *fgf8* enhancers^82^, some of which are marked as ncOCRs by ATAC-seq (Extended Data Fig. 5e and Supplementary Table 26). We did not detect an obvious enhancer near the *fgf8* locus not used or deleted in the bowfin (Extended Data Fig. 5e). As epistasis in regulation of *fgf8* expression is likely to be complex, and Fgf8 signaling at large still likely plays critical roles in development apart from the pectoral fin (Fig. 6e), shared enhancer use may be common. Our results reveal an unexpected drift in the genetic control of holostean fin development, potentially to some other fibroblast growth factor (Fgf) signaling genes, and represent a unique case of robustness of appendage development in the absence of early *fgf8* regulation (Extended Data Fig. 5d and Supplementary Note 11). As these changes are defining for holosteans and not observed in other fishes or tetrapods, these findings demonstrate the importance of holostean genomic information that challenges long-standing assumptions of core developmental mechanisms and informs new models of appendage patterning.

## Discussion

Bowfin’s phylogenetic position and unique suite of ancestral and derived phenotypes make it an important component for understanding vertebrate evolution^1,20^. Our analyses using a chromosome-level genome assembly for this ‘living fossil’ (ref. ^20^) show that, despite its derived karyotype with superficially convergent similarities to those of teleosts, its chromosomal organization nevertheless indicates a closer phylogenetic relationship to gar in the holostean clade. The slow-evolving, ancestrally unduplicated genome and the developmental epigenome of the bowfin will be a critical resource for comparative vertebrate genomics and evolutionary developmental biology. After genome sequencing, many challenges remain in determining and functionally assaying non-coding gene-regulatory loci in both model and non-model species. Here, we have identified putative enhancers for this non-model species and illustrate that our ATAC-seq dataset can be easily connected to orthologous enhancers in fish and more distant vertebrate lineages. The bowfin not only informs genome evolution in holostean fishes but also offers valuable insights into the genomic basis of its archetypical anatomy and the many developmental and physiological phenotypes in ray-finned fishes. While this species represents a once-large taxonomic group that is now mostly extinct and thus lost for studying genomic diversity, the bowfin genome adds a major sequenced branch for genetic and developmental exploration to the fish tree of life.

Bowfin and gars have persisted for millions of years. Fortunately, the negative perception of holosteans as ‘trash fishes’ is changing to include greater appreciation for their evolutionary, ecological and cultural importance and, as shown here, their relevance for understanding vertebrate genome biology. As the *Amia* genus may not be monotypic^2^, our genome assembly will be of utmost importance to evaluate bowfin’s species status and conservation.

## Methods

### Animal work

Bowfin work was performed in compliance with ethical guidelines and approved under Institutional Animal Care and Use Committee protocols from Nicholls State University (IA046/IA053), Michigan State University (10/16-179-00) and Cornell University (2006-0013). Bowfins for genome sequencing, sex-determination analysis and immune transcriptomics were sampled from the Louisiana (USA) population as detailed below. Bowfin embryos and larvae (unsexed) for developmental and fin bud transcriptomics, ATAC-seq and RNA in situ hybridization were collected from nests of the Oneida Lake (New York, USA) population and then raised in the laboratory as previously described^25^ (Supplementary Note 11) until sampling at the desired developmental stages^53^. Spotted gar embryos (unsexed) were obtained from the Louisiana population, raised and fixed as previously described^89^, and protocols were approved by the Nicholls State University (IA053) and the Michigan State University (10/16-179-00) institutional animal care and use committees.

### Genome sequencing and assembly

DNA was extracted from blood of a single, adult, wild male (phenotypic sex was confirmed by gonadal observation) collected from the Atchafalaya River Basin in Louisiana, USA (Supplementary Note 1). A de novo assembly was constructed using a paired-end sequencing library (mean assembly-based insert size, ~410 bp) with Meraculous version 2.2.2.5 (ref. ^26^) (*k*-mer size 55; minimum *k*-mer frequency 55; diploid nonredundant haplotigs). Input data consisted of 433.5 million read pairs sequenced from the paired-end library, totaling 122 Gb after trimming for quality, sequencing adaptors and mate pair adaptors using Trimmomatic version 0.38 (ref. ^90^) (parameters: ‘PE ILLUMINACLIP LEADING:20 TRAILING:20 SLIDINGWINDOW:13:20 MINLEN:23’).

A Chicago library was prepared as previously described^27^. Briefly, ~500 ng HMW gDNA (mean fragment length 50 kb) was reconstituted into chromatin in vitro and fixed in 1% formaldehyde for 15 min at room temperature. Fixed chromatin was digested with DpnII, 5′ overhangs were filled in with biotinylated nucleotides, and free blunt ends were ligated. After ligation, cross-links were reversed, and DNA was purified from protein. Purified DNA was treated to remove biotin that was not internal to ligated fragments. DNA was then sheared to a mean fragment size of ~350 bp, and sequencing libraries were generated using NEBNext Ultra enzymes and Illumina-compatible adaptors. Biotin-containing fragments were isolated using streptavidin beads before PCR enrichment of each library. Libraries were sequenced on an Illumina HiSeq platform to produce 177 million 2 × 101-bp paired-end reads, which provided 89.9× physical coverage of the *k*-mer-based estimated genome size of 0.91 Gb (1–50-kb pairs).

A Hi-C library was prepared as previously described^28^. For each library, chromatin was fixed in place in the nucleus in 1% formaldehyde for 10 min at room temperature and then extracted. Fixed chromatin was digested with DpnII, 5′ overhangs were filled in with biotinylated nucleotides, and then free blunt ends were ligated. After ligation, cross-links were reversed, and DNA was purified from protein. Purified DNA was treated to remove biotin that was not internal to ligated fragments. DNA was sheared to a mean fragment size of ~350 bp, and sequencing libraries were generated using NEBNext Ultra enzymes and Illumina-compatible adaptors. Biotin-containing fragments were isolated using streptavidin beads before PCR enrichment of each library. Libraries were sequenced on an Illumina HiSeq platform to produce 527 million 2 × 151-bp paired-end reads, which provided 22,887× physical coverage of the *k*-mer-based estimated genome size (1–50-kb pairs).

Input de novo assembly, shotgun, Chicago library and Hi-C library reads were used as input data for HiRise^27^ to scaffold genome assemblies with an iterative analysis. First, shotgun and Chicago library sequences were aligned to the draft input assembly using a modified SNAP read mapper (http://snap.cs.berkeley.edu). Separations of Chicago read pairs mapped within draft scaffolds were analyzed by HiRise to produce a likelihood model for genomic distance between read pairs. The model was used to identify and break putative misjoins, to score prospective joins and to make joins above a likelihood threshold. After aligning and scaffolding Chicago data, Dovetail Hi-C library sequences were aligned and scaffolded following the same method. After scaffolding, shotgun sequences were used to close gaps between contigs to generate the final genome assembly (AmiCal1).

### Hi-C contact mapping

Raw Hi-C reads were trimmed with Trimmomatic version 0.38 (ref. ^90^) (parameters: ‘PE, ILLUMINACLIP:adapter_contamination_sequences.txt:1:30:7 MINLEN:25’). BWA version 0.7.17 (ref. ^91^) was used to map forward and reverse reads to the final genome assembly independently with the following parameters: ‘bwa mem -A1 -B4 -E50 -L0’. Downstream analyses were performed with the HiCExplorer version 3.6 toolkit^92^. A Hi-C matrix was constructed from mapped reads at 10-kb resolution with the hicBuildMatrix tool. The hicMergeMatrixBins tool was used to further bin the matrix into 50-kb, 100-kb, 200-kb, 300-kb, 400-kb and 500-kb bin size matrices. All matrices were corrected with hicCorrectMatrix with the filter threshold parameter set to ‘-1.5 5’. TADs were called with hicFindTADs using the following parameters: ‘–correctForMultipleTesting fdr–thresholdComparisons 0.05–delta 0.01’. Hi-C contact maps were plotted using the hicPlotMatrix tool and pyGenomeTracks version 3.6 (ref. ^93^).

### Repeat analysis

A custom bowfin repeat database was constructed using Repeat Modeler version 1.0.8 (ref. ^94^) with default parameters and combined with custom repeat libraries used for gar^7^ and all repeats from the Vertebrata Repbase^95^ (downloaded 15 November 2017). These repeat elements were input to RepeatMasker^96^ within MAKER version 2.31 (ref. ^31^) during gene annotation. Repeat proteins were identified by searching against a library packaged with MAKER via RepeatRunner^97^. The new combined repeat library was used to characterize repeats in both bowfin and spotted gar. RepeatMasker functions ‘calcDivergenceFromAlign.pl’ and ‘createRepeatLandscape.pl’ (ref. ^96^) generated repeat landscapes for both species.

### Gene annotation

Protein-coding genes were annotated with a bowfin reference transcriptome from multiple adult tissues^7,30^ as evidence. The EST2genome function in MAKER version 2.31 (ref. ^31^) was used identify putative bowfin genes based on BLAST^98^ and Exonerate^99^ transcript alignments. Best-scoring genes with an annotation edit distance of 0.2 or less were used to train hidden Markov models with SNAP^100^ and AUGUSTUS^101^ as previously described^102^. Ensembl and RefSeq protein sequences from other vertebrate species were used as additional evidence: gar (LepOcu1), coelacanth (LatCha1), mouse (GRCm38.p5), chicken (Gallus_gallus-5.0), human (GRCh38.p10), *Xenopus* (JGI 4.2), anole lizard (AnoCar2.0), zebrafish (GRCz10), medaka (HdrR), arowana (GCA_001624265.1, ASM162426v1) and elephant shark (GCA_000165045.2, Callorhinchus_milii-6.1.3). Proteins were aligned to the genome using BLAST and Exonerate with default options, guiding gene predictions by HMMs (from SNAP and AUGUSTUS) in the MAKER workflow. MAKER output all predicted genes with and without transcript and protein evidence (MAKER-Max). Pfam protein domains^103^ were identified within the MAKER-Max gene set using HMMER version 3.0b3 (hmmrscan *E* value < 1 × 10^−5^)^104^. MAKER-Max genes with transcript or protein or Pfam domain support were retained as the final MAKER-Standard gene set^105^.

We ran OrthoFinder version 1.1.3 (ref. ^32^) (fastME distance method parameters: ‘-t 20 -M msa -A mafft -T FastTree’) to identify orthologous genes between bowfin and other vertebrates using protein sequences of the longest isoforms from the same species set used for MAKER annotation.

### Bowfin sex-determination analyses

Reference genome-based and genome-free Pool-seq approaches to compare 30 adult bowfin males with 30 adult females (phenotypic sex was confirmed by gonadal observation) from the Louisiana population are described in Supplementary Note 3.

### Genome structure and gene-order analyses

Orthologous genes were extracted from reconciled gene trees built with the Ensembl Compara pipeline^106^. The reconciled gene trees contain 78 species, including 55 fish, 18 other vertebrates and five non-vertebrate outgroups. Briefly, starting with the set of predicted coding sequences, we performed an all-against-all BLAST^98^, followed by clustering with hcluster_sg^107^ to define gene families, multiple-alignment inference using M-Coffee^108^ and phylogenetic tree construction with TreeBeST^106^. From these trees, gene families were defined as groups of genes that are derived from the same ancestral gene. Depending on the set of species used, we chose different ancestral species to define the families, each time taking the most recent common ancestor (see below).

We identified pairs of chromosomes between species sharing significantly more orthologs than expected after random gene shuffling (*P* < 0.05, *χ*^2^ test; Yates correction for small sample size, Bonferroni correction for multiple testing). Karyotype comparisons with spotted gar were based on all orthologous genes inherited from the Neopterygii ancestor (*n* = 16,398 orthologous genes between bowfin and gar; *n* = 14,374 orthologous genes between medaka and gar). Comparisons with chicken were based on all orthologous genes inherited from the Euteleostomi ancestor (*n* = 8,219 orthologous genes between bowfin and chicken; *n* = 7,835 orthologous genes between gar and chicken).

We used a distance-based method to reconstruct the phylogeny of 14 species using gene-order data from high-confidence orthologs inherited from the common Euteleostomi ancestor, reducing all studied genomes to 3,223 marker genes: 1,527 were singletons in all species; 1,614 were present in one copy in bowfin, gar, chicken and *Xenopus* and one or two copies in post-TGD teleosts; 82 were present in one copy in all non-teleosts and two copies in all teleosts. Intervals between these marker genes cover >60% of the most fragmented genomes and >80% of all other genomes and are arranged into 16,064 gene adjacencies that exist in at least one study species. Differences in genome assembly qualities and gene duplications resulted in varying numbers of adjacent gene markers, ranging from 3,028 (Amazon molly) to 3,800 (arowana).

Normalized breakpoint distances between pairs of genomes were computed as the number of gene adjacencies that exist in G1 but not in G2, normalized to the number of adjacencies in G1, where G1 is the genome with the smallest number of adjacencies, in line with previously reported adjustments^109,110^. Species trees were reconstructed on the resulting distance matrix using NJ, minimum evolution (FastME) and UPGMA approaches, with bootstrap replicates generated by resampling 100 times with replacement from the 16,064 gene adjacencies. Bootstrapped NJ trees were used to evaluate the 95% confidence interval of the branch-length difference of bowfin versus gar and bowfin versus teleosts (see Supplementary Note 5 for details). To address the possibility that adjacency losses in teleosts result from loss of TGD-duplicated genes instead of rearrangements, we explicitly ignored any adjacency loss for marker genes on alternative TGD-derived regions, which did not affect the results.

Comparing Holostei versus Halecostomi scenarios within a maximum parsimony framework, we treated each of the 566 Neopterygian-specific gene adjacencies as an independent character, either absent or present in each Neopterygian genome, and placed adjacency gains and losses on the two alternative phylogenies according to the assumptions of Dollo’s parsimony^111^, as chances are extremely low that distinct rearrangements would (re)create the same gene adjacency. To assess whether one scenario was significantly more parsimonious than the other, we used the KH test (Phylip package^112^, dollop program).

### Gene-family analyses

Immune, SCPP and Hox genes were annotated and analyzed as detailed in Supplementary Notes 6, 7 and 10, respectively. Generally, spotted gar and/or other vertebrate sequences were used in BLAST searches^98^ against the bowfin genome assembly to generate manually curated annotations further supported by diverse bowfin transcriptomic evidence (described below and published^7,30^). Orthologies were assigned based on phylogenetic and synteny evidence as described in the respective Supplementary Notes.

### Developmental transcriptomics

Total RNA was extracted (Qiagen RNeasy Mini Plus) from RNAlater-preserved (Ambion), unsexed embryos or larvae from the Oneida Lake population at Ballard stages 22–23, 23–24, 24–25, 26–27, 28–29 and 30–31 (ref. ^53^). Stage 22–23 was defined as the bowfin phylotypic stage by similarity to the phylotypic stages of zebrafish and medaka^66^. Five embryos were pooled for stages 22–23 and 24–25; one embryo or larva was used for stages 23–24, 26–27, 28–29 and 30–31. Libraries were generated with the Illumina TruSeq Stranded mRNA Library Preparation Kit with IDT for Illumina Unique Dual Index and sequenced on an Illumina NextSeq version 2.5 (Mid Output flow cell, 2 × 75-bp paired-end format, 150-cycle version 2.5 NextSeq 500 reagent cartridge). After base calling with Illumina Real Time Analysis (RTA) version 2.4.11, sequences were demultiplexed and converted to FastQ format with Illumina bcl2fastq version 2.19.1. Read pairs were trimmed for quality and adaptor sequences with Trimmomatic version 0.38 (ref. ^90^) using the same parameters as described above for Hi-C reads. Remaining paired-end reads were mapped to the repeat-masked bowfin genome with Bowtie 2 (ref. ^113^) (‘very-sensitive’ option, other parameters set to default). Reads mapping to the mitochondrial genome were removed with ‘removeChrom.py’ (https://github.com/harvardinformatics/ATAC-seq/blob/master/atacseq/removeChrom.py), reads from PCR duplicates were removed with Picard Tools version 2.18.1 (‘mark duplicates’ function) (https://broadinstitute.github.io/picard/), and SAMtools view (‘-b -q 10’) was used to remove poorly mapped reads^114^. Reads were visualized in IGV^115^ after normalization with BPM (bin size, 25) with the deepTools version 3.1.1 (ref. ^116^) bamCoverage tool.

### Immune tissue transcriptomics

Immune tissues (gill, spleen, intestine) were dissected from a single adult female bowfin from Bayou Chevreuil, Louisiana. RNA was extracted from each individual tissue (Qiagen RNeasy kit), quantified (Thermo Fisher NanoDrop and Agilent Bioanalyzer) and pooled in equal amounts before library barcoding. Sequencing reads from the NovaSeq 6000 were quality trimmed using Trimmomatic version 0.38 (ref. ^90^) and assembled into transcripts using genome-guided Trinity version 2.8.5 (ref. ^117^) (Supplementary Note 6).

### Fin bud expression analyses

Whole, unsexed bowfin embryos from the Oneida Lake population were incubated overnight in RNAlater at 4 °C, followed by dissection of pectoral fin buds using tungsten needles with three replicates of six to seven animals collected at the desired stages. Tissues were dissolved by incubation in RLT Plus. RNA was isolated from both whole animals (for cDNA cloning) and dissected fin buds using the RNeasy Plus Micro kit (Qiagen). For standard cloning, cDNA libraries were produced from RNA extracted from stage 23 and stage 25 single whole embryos and from stage 26 fin buds using the SuperScript IV Reverse Transcriptase System (Invitrogen). Following polyA selection, stranded mRNA-seq libraries were produced using the PrepX RNA-seq Library Kit for Illumina (IntegenX) from stage 23, 24, 25 and 26 fin bud RNA. For each developmental stage, two replicate libraries were sequenced with 75-bp single-end reads, and a third was sequenced with 150-bp paired-end reads on an Illumina NextSeq machine. Reads were filtered using Trimmomatic version 0.38 (ref. ^90^), and ribosomal RNA reads were removed using SortMeRNA^118^. Filtered non-rRNA reads were aligned to the bowfin nuclear transcriptome using CLC Genomics Workbench (Qiagen). The transcriptome toolkit in CLC Genomics Workbench was used to calculate RPKM values, fold change and analysis of differential gene expression. Heatmaps to visualize gene expression dynamics were created using the Pretty Heatmaps package in R (https://rdrr.io/cran/pheatmap/).

### Assay for transposase-accessible chromatin with sequencing analysis

ATAC-seq library preparation and amplification with barcoding was performed using a modification of published protocols^52,119^. Samples were taken from the same unsexed clutches (Oneida Lake population) and stages as for the developmental transcriptome series, covering stages 21–31 (see above). One whole embryo per stage was dissociated into a cell solution with 0.125% collagenase (Sigma, C9891) at 37 °C until completely dissociated and then strained (100-µM filter) and counted on an improved Neubauer chamber. In total, 100,000 cells were collected and lysed per sample for DNA transposition and PCR amplification. Agencourt AMPure XP beads were used to clean up PCR products. ATAC-seq libraries were individually barcoded, pooled and sequenced with Illumina NextSeq version 2 (High Output flow cell, 2 × 75-bp paired-end format, 150-cycle version 2 NextSeq 500 reagent cartridge). Base calling, demultiplexing, FastQ format conversion, read trimming, mapping to the bowfin genome, alignment filtering and data visualization of ATAC-seq reads in IGV^115^ were performed as described above for developmental transcriptome RNA-seq data.

To identify OCRs, ATAC-seq peaks were called with MACS2 version 2.2.6 (ref. ^120^) (parameters: ‘callpeak -f BAMPE -g 767196669 -B -q 0.05 -s 75–call-summits’). BEDTools^121^ ‘subtract -A’ was used to subtract exon coordinates (UTRs and coding regions) from OCR coordinates in each library to generate ncOCRs, defined as OCRs with no overlap (0 bp) with any MAKER-annotated exon. BEDTools^121^ merge was used to merge OCRs and ncOCRs within libraries and stages (across replicates) as well as across stages for a global picture of open chromatin through development. OCRs merged within stage were annotated with HOMER^54^ ‘annotatePeaks.pl’ (‘-size ‘given’ -annStats’). Transcriptional start sites were defined from −1 kb to +100 bp; transcriptional termination sites were defined from −100 bp to +1 kb. OCR overlap and uniqueness between developmental stages were quantified with HOMER^54^ ‘mergePeaks’ (parameters: ‘-d given -venn’). Pairwise Jaccard distances were calculated between stages and replicates with the BEDTools Jaccard option and visualized with a heatmap created with Heatmapper^122^ and a PCA plot created with ggplot2 (ref. ^123^). Read pileup and merged OCRs from MACS2 were visualized in IGV^115^ after normalization with BPM (bin size, 25) with the deepTools version 3.1.1 (ref. ^116^) bamCoverage tool.

### Whole-genome alignments and OCR overlap

We used Progressive Cactus version 1.2.3 (ref. ^55^) to align bowfin, gar, zebrafish (GRCz11), human (hg38) and mouse (GRCm38) genomes given the phylogeny (((gar, bowfin), zebrafish), (human, mouse)) using default parameters. After completion of alignment, we used the ‘halLiftover’ tool^55^ to identify homologous elements between species as follows: ‘halLiftover–noDupes vertebrates.hal <reference species> reference_sp_coordinates.bed <target species> target_sp_coordinates.bed’. We used this approach to lift bowfin OCRs to all other species as well as lift mouse OCRs^65^, human and mouse Vista enhancers^56^ and our previously generated gar-centric CNEs^7^ to the bowfin genome. Gar-centric CNEs (sensu^7^; 156,087 total elements) were defined as ≥50-bp phastCons elements from a 13-way MultiZ vertebrate WGA, filtered for genic and repeat regions to obtain CNEs^7^. We counted all ≥50-bp lifted elements as ‘conserved’ between the reference and target species and summarized overlap between the conserved elements from other species with bowfin OCRs using BEDTools (parameters: ‘intersect -wa -f 0.33’). We counted all conserved elements that have at least 33% of their length overlapping a bowfin OCR. We applied this method to all bowfin OCRs and ncOCRs and separately to those represented in at least two developmental stages (defined by HOMER mergePeaks). Locations of published ray-finned fish UCEs^6^ (366 total elements) in the bowfin genome were established through BLASTN (*E* value < 1 × 10^−5^). Ray-finned fish UCEs (sensu^6^) have been defined as ultraconserved, ≥120-bp single-copy nuclear DNA elements with ≥80% sequence identity across a five-species teleost WGA, covering both coding and non-coding sequence space^6^.

### Local genomic sequence-conservation analyses

Repeat-masked genomic regions surrounding *tbx4* and *fgf8* in bowfin and other vertebrates were aligned with mVISTA^87^ using SLAGAN^88^ as detailed in Supplementary Notes 9 and 11, respectively.

### RNA in situ hybridization

For RNA in situ hybridization and skeletal staining, unsexed embryos of bowfin (Oneida Lake population) and gar (Louisiana population) were fixed overnight at 4 °C in 4% paraformaldehyde in PBS, washed twice for 5 min in PBS with 0.1% Tween, washed once in 100% methanol for 5 min and stored in 100% methanol at −20 °C. Amplified bowfin and gar cDNA (see Supplementary Note 11 for primer sets) was cloned into pGEM-T Easy (Promega). Antisense probes were synthesized as previously described^124^, and whole-mount in situ hybridization was performed using a standard embryo protocol^125^ (*n* = 10 per gene and species).

### Reporting Summary

Further information on research design is available in the Nature Research Reporting Summary linked to this article.

## Online content

Any methods, additional references, Nature Research reporting summaries, source data, extended data, supplementary information, acknowledgements, peer review information; details of author contributions and competing interests; and statements of data and code availability are available at 10.1038/s41588-021-00914-y.

## Supplementary information

Supplementary Information Supplementary Notes 1–11, Figs. 1–18 and Tables 1–6, 8, 16–22, 24 and 26

Reporting Summary

Peer Review Information

Supplementary Tables Supplementary Tables 7, 9–15, 23 and 25. Supplementary Table 7. Bowfin sex-biased CDSs and gene annotations. Supplementary Table 9. MHC region genes in bowfin, human and zebrafish. Supplementary Table 10. Spotted gar MHC genes. Supplementary Table 11. MHC sequence accession identifiers. Supplementary Table 12. Immunoglobulin heavy chain (IgH) sequence accession identifiers. Supplementary Table 13. Immunoglobulin light chain (IgL) sequence accession identifiers. Supplementary Table 14. TCR sequence accession identifiers. Supplementary Table 15. Bowfin TLRs. Supplementary Table 23. Location of human VISTA enhancers in bowfin OCRs. Supplementary Table 25. Developmental patterning genes used for fin transcriptome PCA.

Supplementary Data 1 Bowfin SCPP gene predictions.

Supplementary Data 2 Bowfin Hox gene transcripts.

## Data Availability

The bowfin reference genome assembly (AmiCal1) is available at GenBank under the accession number PESF00000000; raw reads are available at the Sequence Read Archive under accession numbers SRR14766073–SRR14766075. Transcriptomic and ATAC-seq reads are available under accession numbers SRP281665 and SRP252716; assembled transcripts are available under accession number GIOP00000000. The MAKER gene annotation is available at https://github.com/AndrewWT/AmiaGenomics. Data for synteny analyses and the gene-order phylogeny are available at https://github.com/DyogenIBENS/BowfinGOPhylogeny. Source data are provided with this paper.

## Source data

Source Data Fig. 3 Genomic locations and gene IDs of SCPP genes in gar, zebrafish, cavefish and channel catfish. Bowfin SCPP genes are provided in Supplementary Data 1.
Source Data Fig. 6 Expression values (in RPKM) for the genes shown in heatmaps in Fig. 6c,d and in Extended Data Fig. 5d.
Source Data Extended Data Fig. 1 Locations of TADs in the bowfin genome (300-kb domains) shown in Extended Data Fig. 1d. Note that, as indicated in Data availability, other source data for Extended Data Fig. 1 are available from the NCBI (accessions PESF00000000 for Extended Data Fig. 1a, SRR14766074 for Extended Data Fig. 1b,c, SRP281665 for Extended Data Fig. 1e), https://github.com/AndrewWT/AmiaGenomics (gene annotations in Extended Data Fig. 1d,e) and https://github.com/DyogenIBENS/BowfinGOPhylogeny/tree/main/data (synteny data in Extended Data Fig. 1f).
Source Data Extended Data Fig. 3 Genomic locations of peptide-coding regions of IgH, IgL κ, IgL σ, TCR α and δ, TCR β, TCR γ and B2M genes in the bowfin genome assembly.
Source Data Extended Data Fig. 5 Expression values (in RPKM) for the genes shown in the heatmap in Extended Data Fig. 5d.

## Code availability

Custom scripts and workflows for the Pool-seq sex-determination analysis are available at https://github.com/RomainFeron/paper-sexdetermination-bowfin and for the gene-order phylogeny at https://github.com/DyogenIBENS/BowfinGOPhylogeny. All other software was used as described in detail in the Methods and the Nature Research Reporting Summary.
