# Supplementary material for: The bowfin genome illuminates the developmental evolution of ray-finned fishes
Source: Nat Genet. 2021 Aug 30;53(9):1373–84. doi: 10.1038/s41588-021-00914-y (PMC8423624; doi:10.1038/s41588-021-00914-y)
Supplement: Supplementary file 1 — Supplementary Notes 1–11, Figs. 1–18 and Tables 1–6, 8, 16–22, 24 and 26 [file 41588_2021_914_MOESM1_ESM.pdf]

nature portfolio

https://doi.org/10.1038/s41588-021-00914-y

Supplementary information

The bowfin genome illuminates the developmental evolution of ray-finned fishes

In the format provided by the authors and unedited

SUPPLEMENTARY INFORMATION

The genome of the bowfin (*Amia calva*) illuminates the developmental evolution of ray-finned fishes

Andrew W. Thompson, M. Brent Hawkins, Elise Parey, Dustin J. Wcisel, Tatsuya Ota, Kazuhiko Kawasaki, Emily Funk, Mauricio Losilla, Olivia E. Fitch, Qiaowei Pan, Romain Feron, Alexandra Louis, Jérôme Montfort, Marine Milhes, Brett L. Racicot, Kevin L. Childs, Quenton Fontenot, Allyse Ferrara, Solomon R. David, Amy R. McCune, Alex Dornburg, Jeffrey A. Yoder, Yann Guiguen, Hugues Roest Crollius, Camille Berthelot, Matthew P. Harris, and Ingo Braasch

* Corresponding author: Ingo Braasch

Email: braasch@msu.edu

Supplementary Notes 1-11

Suppl. Note 1. Genome sequencing and assembly
Suppl. Note 2. Genome annotation
Suppl. Note 3. Sex determination analysis
Suppl. Note 4. Phylogenomic analyses
Suppl. Note 5. Gene order rearrangement rate analyses
Suppl. Note 6. The bowfin immune gene repertoire
Suppl. Note 7. SCPP genes and scale formation
Suppl. Note 8. ATAC-Seq chromatin profiling of bowfin development
Suppl. Note 9. Bowfin informs the evolution of the tbx4 'lung' enhancer
Suppl. Note 10. Hox gene cluster analyses
Suppl. Note 11. Fin development analyses

Supplementary Figures 1-18

Suppl. Fig. 1. Chicago library
Suppl. Fig. 2. Hi-C scaffolding of the bowfin genome assembly
Suppl. Fig. 3. Repeat landscapes of holostean genomes
Suppl. Fig. 4. Bowfin sex determination analysis
Suppl. Fig. 5. Holostean genomes compared to the bichir genome
Suppl. Fig. 6. Gene order rearrangement distance matrix
Suppl. Fig. 7. Phylogenetic analyses support holostean monophyly
Suppl. Fig. 8. Synteny comparison of bowfin vs. zebrafish MHC
Suppl. Fig. 9. MHC cladograms
Suppl. Fig. 10. IgH cladograms
Suppl. Fig. 11. IgL cladograms
Suppl. Fig. 12. T cell receptor (TCR) cladograms
Suppl. Fig. 13. Toll-like receptor phylogeny
Suppl. Fig. 14. Teleost Scpp5 and Scpp1 proteins
Suppl. Fig. 15. ATAC-Seq sampling through bowfin development
Suppl. Fig. 16. Conservation of bowfin non-coding open chromatin regions
Suppl. Fig. 17. Bowfin *hoxd14* pseudogene
Suppl. Fig. 18. Principle Component Analysis of the fin transcriptome

Supplementary Tables 1-26

Suppl. Tab. 1. Assembly statistics for bowfin genome AmiCal1
Suppl. Tab. 2. Red mapping statistics
Suppl. Tab. 3. BUSCO and CEGMA scores for bowfin genome assembly and annotation
Suppl. Tab. 4. Repeat content in bowfin in comparison to spotted gar
Suppl. Tab. 5. OrthoFinder results summary
Suppl. Tab. 6. Orthogroup statistics for bowfin and 11 other vertebrates
Suppl. Tab. 7. Bowfin sex-biased CDSs and gene annotations*
Suppl. Tab. 8. Reference-free k-mer analysis of bowfin sex
Suppl. Tab. 9. MHC region genes in bowfin, human, and zebrafish*
Suppl. Tab. 10. Spotted gar MHC genes*
Suppl. Tab. 11. MHC Sequence accession identifiers*
Suppl. Tab. 12. Immunoglobulin heavy chain (IgH) sequence accession identifiers*
Suppl. Tab. 13. Immunoglobulin light chain (IgL) sequence accession identifiers*
Suppl. Tab. 14. T cell receptor (TCR) sequence accession identifiers*
Suppl. Tab. 15. Bowfin Toll-like Receptor (TLRs)*
Suppl. Tab. 16. SCPP gene expression in zebrafish skin at three different ages
Suppl. Tab. 17. Open Chromatin Regions found for each bowfin developmental stage
Suppl. Tab. 18. Bowfin OCRs in N developmental stages
Suppl. Tab. 19. HOMER annotation of OCRs in each developmental stage

Suppl. Tab. 20. Conservation of bowfin OCRs/ncOCRs in other vertebrates
Suppl. Tab. 21. Overlap of bowfin OCRs with gar-centric CNEs and bowfin UCEs
Suppl. Tab. 22. Number of VISTA enhancers detected in bowfin and zebrafish genomes
Suppl. Tab. 23. Location of human VISTA enhancers in bowfin OCRs*
Suppl. Tab. 24. Mouse OCRs found in bowfin
Suppl. Tab. 25. Developmental patterning genes used for fin transcriptome PCA*
Suppl. Tab. 26. ATAC-Seq profile of putative *fgf8* gene regulatory regions in bowfin

Supplementary Data 1-2

Suppl. Data 1. Bowfin SCPP gene predictions*
Suppl. Data 2. Bowfin hox gene transcripts*

Supplementary References

* provided in a separate file

Supplementary Note 1. Genome sequencing and assembly

1.1 Genome specimen. The bowfin genome assembly is based on DNA extracted from blood drawn of a single, wild, adult male individual ("Calvin", sample ID Aca15.3; phenotypic sex confirmed by gonadal observation), collected from the Atchafalaya Basin population near Stephensville, Louisiana, USA (coordinates 29.812569 N 91.220803 W), and sacrificed in the laboratory of Allyse Ferrara (Nicholls State University, Thibodaux, LA). Information on the genome specimen is filed under NCBI BioProject PRJNA417081, BioSample SAMN07977036.

1.2 Genome assembly. The bowfin genome was sequenced and assembled in partnership with Dovetail Genomics [https://dovetailgenomics.com/]. The Meraculous[1] *de novo* genome assembly consisted of 433.5 million read pairs (NCBI accession: SRR14766075) assembled into 64,769 1 kb+ scaffolds with a total length of 768.2 Mb. The Chicago[2] assembly consisted of 177M read pairs (NCBI accession: SRR14766073) and resulted in 62,981 joins reducing the number of 1Kb+ scaffolds to 1,846 and increasing total assembly length to 775.8 Mb. The Hi-C library consisted of 527M read pairs (NCBI accession: SRR14766074) and the final assembly (Chicago + Hi-C scaffolded with HiRise[2]) resulted in 1,958 1 kb+ scaffolds with a total length of 831.0 Mb after additional 251 additional joins (Supplementary Table 1). 94% of the *de novo* genomic reads mapped to the final assembly (Supplementary Table 2). The k-mer-based estimated genome size of bowfin is 0.908 Gb. The bowfin genome size was previously estimated to be ~1.1 Gb based on Feulgen staining[3]. Scaffolds of the genome were ordered from longest to shortest and labeled as Aca scaf1 to Aca scaf1957. The bowfin karyotype is n=23[4,5]. The 23 largest assembly scaffolds range from 57.1 Mb and 20.5 Mb in size, with a clear drop in length to 319 kb for the 24th largest scaffold (Extended Data Fig. 1a). The assembly is thus at the chromosome-level with 99% of the assembly being represented by the 23 largest pseudochromosomes. The mitochondrial genome was identified as a single scaffold (Aca scaf1958) based on sequence comparison to the published *A. calva* mitogenome (NCBI RefSeq: NC_004742.1). High BUSCO[6] and CEGMA[7] scores, e.g. 95% Vertebrata BUSCOs and 98% eukaryotic CEGs, respectively (Supplementary Table 3), further support the high quality and completeness of the final assembly AmiCal1 (NCBI accession: PESF00000000).

Supplementary Note 2. Genome annotation

2.1 Repeat analysis. Both bowfin (AmiCal1) and spotted gar (LepOcu1) genome assemblies were annotated with the same set of repeat libraries to ensure maximal comparability. 22.1% of the bowfin genome is repetitive, very similar to the 22.8% repeat content of spotted gar, although there are clear differences in the distribution among repeat element types between the two species (Supplementary Table 4). Bowfin has a higher content of DNA transposons than spotted gar (7.1% vs. 4.2% of the genome assembly), a lower LINE content (3.9% vs. 6.3%), a higher level of satellite/simple repeat elements (2.1% vs. 0.6%), and a lower SINE content (0.3% vs. 3.1%).

Importantly, Kimura substitution level-based repeat landscapes show very different timing of repeat activities between bowfin and gar (Supplementary Fig. 3). Spotted gar shows bursts of transposable element (TE) activity at Kimura distances 25 and 7-8 (Supplementary Fig. 3b) as described previously[8]. Bowfin in contrast does not show any 'older' peak of TE activity, but a more pronounced and more 'recent' one at Kimura distance 4 (Supplementary Fig. 3a) that is not seen in spotted gar (Supplementary Fig. 3b). These non-overlapping peaks in bowfin vs. spotted gar are also based on amplifications of different TE classes. Despite their overall similar total repeat content, these two holostean lineages thus have undergone pronouncedly different TE activity trajectories. Neither of the two genomes shows a very strong recent TE activity at Kimura distance 0-1 as seen in mammals and teleosts[8].

2.2 Gene annotation and orthology prediction. MAKER[9] annotated 49,283 putative gene models based on evidence and model predictions (MAKER-Max). After screening for homology with Pfam protein domains[10], we retained 21,948 genes with transcriptional and/or Pfam domain evidence as the final MAKER-Standard gene set (available at https://github.com/AndrewWT/AmiaGenomics). This number is a realistic expectation when compared to spotted gar, which has 21,443 MAKER annotated genes[8]. High BUSCO scores for the predicted transcripts and proteins show that the MAKER-Standard gene annotation is close to complete (Supplementary Table 3). OrthoFinder[11] placed 86.8% of bowfin genes into orthogroups and identified 7,532 orthogroups present in all included species (gar, coelacanth, mouse, chicken, human, western clawed frog, anole lizard, zebrafish, medaka, arowana, and elephant shark). OrthoFinder output is summarized in Supplementary Tables 5-6.

Supplementary Note 3. Sex determination analysis

Background. Unlike all other living non-teleost ray-finned fishes, bowfin shows prominent sexual dimorphism with male-specific color patterns (Fig. 1a, Supplementary Fig. 4a-b) and complex reproductive behavior of paternal care with nest building and guarding of larvae[12]. Such phenotypic differences among sexes could be based on differentiated sex chromosomes (e.g.[13]), but cytogenetic sex differences have not been reported for bowfin[4,5], and its sex determination system remains unknown.

Pool-sequencing (Pool-Seq) analyses have been shown to be effective in revealing sex determination systems in many species (e.g.[14-17]). Thus, we used a Pool-Seq strategy contrasting 30 mature, adult phenotypic males vs. 30 mature, adult phenotypic females using reference genome-based and genome-free approaches.

Methods. Fin clips were sampled from 30 adult males and 30 adult females caught near Thibodaux, Louisiana. All these animals were mature adult bowfins, and their phenotypic sex was confirmed by macroscopical examination of the gonads. Individual genomic DNAs were extracted (Qiagen DNeasy Blood & Tissue kit), quantified (NanoDrop spectrophotometer), and mixed in equimolar quantities to produce a male pool and a female pool. Pool-Sequencing (Pool-Seq) libraries were prepared with the Illumina TruSeq Nano DNA HT Library Prep Kit, quality assessed (Advanced Analytical Fragment Analyzer), and quantified by QPCR using (Kapa Library Quantification Kit). Pool-Seq libraries (mean insert size: 419 bp female pool, 412 bp male pool) were sequenced on an Illumina NovaSeq6000 S4 lane (paired-end read length of 2x150 bp), resulting in 283.86 and 284.28 million reads for the female and male pool, respectively.

Pool-Seq analyses were carried out using a snakemake workflow [https://github.com/SexGenomicsToolkit/PSASS-workflow] and figures were generated with the SexGenomicsToolkit R package, *sgtr* v1.1.1 [10.5281/zenodo.3773063]. Reads were aligned to the reference genome with BWA mem v0.7.17[18], and PCR duplicates were removed using samtools rmdup v1.10[19]. A file containing nucleotide counts for each genomic position was generated with the PSASS pileup command v3.1.0 [10.5281/zenodo.4442702] and used to compute the Fixation index (F_{ST}), a measure of genetic differentiation between populations, here between males and females; the numbers of male- and female-specific SNPs; and male and female read depth in a sliding window along the entire genome using the PSASS analyze command. Analyses were run using two sets of parameters: stringent (--window-size 50000, --output-resolution: 1000, --freq-het 0.5, --range-het 0.05, --freq-hom 1, --range-hom 0, and --group-snps set as true) and default (--window-size 50000, --output-resolution: 1000, --freq-het 0.5, --range-het 0.10, --freq-hom 1, --range-hom 0.05, and --group-snps set as true). There were no qualitative differences between parameter sets, so we only report results for the default parameter set. The number of sex-specific SNPs and average depth for each pool were computed for all genes and CDS in the bowfin genome annotation using the "--gff-file" setting from PSASS. Sex-biased genes and CDS were filtered to retain only genes with a corrected male or female depth > 10 reads, and with either a

difference between male and female sex-specific SNPs > 5 or a male/female corrected depth ratio >= 1.5 or <= 0.66. We used our set of reconciled gene trees, built with the Ensembl Compara pipeline (see Methods for ‘Genome structure and gene order analyses’), to annotate these candidate sex-biased genes. In a first pass, we extracted spotted gar genes orthologous to bowfin candidate genes. The corresponding spotted gar gene names and descriptions, as provided in Ensembl Compara v95, were then used to annotate bowfin genes. To increase the number of annotated genes, we similarly extracted zebrafish orthologs of remaining candidates.

As a reference-free approach, we searched for sex-biased k-mers in the Pool-Seq data to overcome potential failure to identify sex-specific regions due to a genome reference from the homogametic sex. First, 31-mers were identified and counted in the male and female Pool-Seq reads using the Jellyfish v2.2.10[20] (“count” command, option “-C” counting only canonical 31-mers and retaining only k-mers with occurrences of 5-50,000). For each sex, 31-mer count tables were obtained with Jellyfish “dump”. 786,071,887 and 786,272,592 31-mers were identified in the female and male reads, respectively. 31-mer count tables were merged and then filtered with Kpool [https://github.com/SexGenomicsToolkit/kpool].

Results and Discussion. We searched for regions showing male versus female differences on the bowfin genome assembly using sex-specific Pool-Seq reads from 30 males and 30 females. We computed F_{ST}, number of sex-specific SNPs, and ratio of read depth between males and females in a sliding window along the genome and found no large region showing a clear difference between sexes (Supplementary Fig. 4c-f). In this Pool-Seq analysis, only very small genomic regions were found to have a sex-biased signal, but these regions were scattered across the genome with no consistent trend (i.e., consistently male- or female-biased). Such a pattern does not fit with the expectation of a single sex locus under the hypothesis of a simple monofactorial genetic sex determining system. To complement this analysis, we also searched for coding sequences (CDS) displaying a male- or female sex-biased signal (see main text Methods). We identified 104 CDS (Supplementary Table 7) exhibiting a sex-biased pattern (either sex-biased coverage or sex-biased SNPs). These 104 genes were scattered on 22 of the 23 bowfin pseudochromosomes and on 3 short, unplaced scaffolds (Aca scaf31, Aca scaf55, and Aca scaf68), without any meaningful enrichment (i.e., more than a single gene in a given genomic region with the same bias) in a restricted region that could indicate a potential sex determining region on a sex chromosome. Our annotation did not reveal any orthologs of known vertebrate sex determination genes (Supplementary Table 7) among the 104 genes exhibiting a sex-biased pattern.

Without any clear indication of a bowfin sex determination system, we cannot rule out the hypothesis that our bowfin reference assembly from a phenotypic male represents the homogametic sex, and thus would not allow the detection of potential female-specific regions. Consequently, we complemented our reference-based analyses with a reference-free approach searching for enrichment of sex-specific k-mers, which has been used as an indication for sex determination systems in other species[16,17,21]. This k-mer analysis yielded a high number of both male- and female-biased k-mers with only a slight excess of male-biased k-mers compared to female-biased k-mers (Supplementary Table 8). These results suggest that if bowfin has a genetic sex determination system, it is either more complex than a simple monofactorial heterogametic system (XX/XY or ZZ/ZW), or its sex locus is too small to be detected with confidence using a Pool-Seq approach. Complex polyfactorial systems have been described in vertebrates[22], and pure environmental sex determination or combinations of environmental effects and genetic sex determination are common among bony fishes[23]. These complex cases cannot be easily identified by contrasting pools of male and female reads. More powerful experimental designs would be needed to characterize such cases, for instance the combination of genome-wide association studies with classical high resolution linkage mapping that was used to successfully identify a very small sex determination region in the pufferfish *Takifugu rubripes*[24].

In summary, our results strongly suggests that sex determination in bowfin is either polyfactorial, too small to be detected using Pool-Seq, and/or based on environmental effects. It is worth noting that,

consistent with bowfin, no genomic sex differentiation loci have been found using similar approaches in spotted gar[25]. Holostean genetic sex determination mechanisms thus remain elusive if they exist at all.

Supplementary Note 4. Phylogenomic analyses

Methods. From the OrthoFinder analysis, we extracted and concatenated the protein alignments of 2,079 1:1 orthologs (single copy in all 12 species) into a single protein matrix. This alignment of 1,518,339 amino acid positions was used as input for phylogenetic tree reconstructions on the CIPRES portal[26] using Maximum Likelihood and Bayesian approaches via RAXML[27] and BEAST2[28], respectively. RAXML parameters were as follows: raxmlHPC-HYBRID-AVX -T 4 -f a -N autoMRE -p 12345 -m PROTGAMMADAYHOFF -x 12345 -o elephant shark. The Rapid BS search stopped after 50 replicates with the MRE-based Bootstopping criterion. BEAST v2.6.3 parameters were as follows: Dayhoff protein matrix with a gamma site model and 4 rate categories; MCMC chain Length=10000000, storing every 5000; a birth-death model with default prior settings; and a relaxed clock with lognormal distribution with default prior settings. In addition, we used STAG[29] to infer the species tree from a set of 7,532 OrthoFinder-generated gene trees with all 12 species present.

Results and Discussion. The Maximum Likelihood tree shown in Supplementary Fig. 7a supports all nodes with 100% bootstrap support, including the monophyletic holostean clade. All nodes are also supported by 1.0 posterior probability in the Bayesian analysis. The species tree generated by OrthoFinder via STAG[29] also supports holostean monophyly (Supplementary Fig. 7b). These sequence-based phylogenies are consistent with both the phylogenomic literature (e.g.[8,30-36]) as well as our gene order-based phylogenetic reconstruction and parsimony analyses presented in main Fig. 1e,f.

Supplementary Note 5. Gene order rearrangement rate analyses

Methods. Using the bootstrapped Neighbor-Joining trees based on the gene order distance matrix (Supplementary Fig. 6; see also main text Methods), we evaluated the 95% confidence interval (95CI) of the branch length difference of bowfin vs. gar and bowfin vs. three main teleost lineages since their respective Last Common Ancestor (LCA).

Results. While branch length differences are non-significant between bowfin and gar, the gene order rearrangement rate is significantly lower in bowfin compared to representatives of three main teleost lineages, i.e., Ostariophysii (zebrafish), Neoteleostei (fugu), and Osteoglossomorpha (arowana). Note that this is not simply an effect of gene adjacency losses due to TGD gene duplicate losses, as these have been ignored in our analysis (see main text Methods). Our results further illustrate that holosteans have comparatively slow rates of evolution.

- **Bowfin vs. Gar** (bowfin branch - gar branch since LCA) 95CI:
  [-0.009, 0.006] ***NON-SIGNIFICANT***
- **Bowfin vs. Zebrafish** (bowfin branch - zebrafish branch since LCA) 95CI:
  [-0.22703016917, -0.196453759465] ***SIGNIFICANT***
- **Bowfin vs. Fugu** (bowfin branch - fugu branch since LCA) 95CI:
  [-0.22406387510500003,-0.198233844745] ***SIGNIFICANT***
- **Bowfin vs. Arowana** (bowfin branch - arowana branch since LCA) 95CI:
  [-0.06424480677000002,-0.04047119256000001] ***SIGNIFICANT***

Supplementary Note 6. The bowfin immune gene repertoire

6.1 Immune tissue transcriptome. A NovaSeq6000 sequencing run from immune tissues (gill, spleen, and intestine) generated 7.6 Gb of reads that were mapped to the reference genome and assembled into 91,498 transcripts with Trinity[49,50] (N50 = 3,054 bp). More than 93% of cleaned reads mapped to the genome-guided transcriptome assembly, suggesting a high-quality assembly. BUSCO[6] analysis confirmed 72.9% (3,630/4,584) complete Actinopterygii (orthoDB9) ultraconserved orthologs are present in the transcriptome. NCBI accession numbers: raw sequencing reads SRR11303972 (SRA); assembled transcripts GIOP00000000 (TSA).

6.2 Major Histocompatibility Complex (MHC) sequences

6.2.1 MHC Background. MHC molecules present antigenic (mostly foreign) peptides processed in host cells to T-cells, thereby enabling immunological cells to recognize foreign antigens and eliminate infected host cells upon binding of cell surface T-Cell Receptors (TCRs) with MHC-antigen complexes. Two different types of MHC molecules are present in vertebrates: class I and class II. Classical class I molecules consist of MHC class I alpha and beta-2-microglobulin (B2M) heterodimers and classical class II molecules consist of MHC class II alpha and beta heterodimers. Class I alpha proteins consist of three extracellular domains: alpha1, alpha2, and alpha3 domains, that play roles in displaying peptide antigens (alpha1 and alpha2) and dimerize with B2M (alpha3). In humans, MHC class III genes are present between classical class I and class II genes and, although not directly involved in antigen presentation, many class III genes play important roles in inflammation and cell signaling[37,38]. In the genomes of tetrapods (including humans) and cartilaginous fish, the MHC class I and class II genes are linked on a single chromosome[39]. However, in teleosts, class I and class II genes are typically present on different chromosomes[40,41]. Earlier efforts to determine the genomic organization of MHC genes in the non-teleost spotted gar were hampered by its MHC genes being located on a large number of small scaffolds[8].

6.2.2 MHC Methods. BLAST searches[42] of the bowfin reference genome using spotted gar MHC sequences as queries identified bowfin pseudochromosomes containing MHC class I and class II loci. Portions of these pseudochromosomes (encoding MHC-related sequences) were used as queries in subsequent BLASTN and TBLASTN searches of the bowfin genome and identified additional genes encoding MHC class I and MHC class II loci. BLASTX analyses were used to confirm the identity of MHC sequences.

The majority of MHC class I and all class II genes were identified in a single pseudochromosome, Aca scaf14. This pseudochromosome was examined closely to identify MHC-related as well as non-MHC genes. Repeat masked pseudochromosome sequences were used as queries for BLASTX searches to identify coding segments within the pseudochromosome. Coding sequences were then used as queries in BLASTN searches of bowfin RNA-seq transcriptomes to identify expressed genes. If alternatively spliced transcripts were identified, the longest isoforms were selected and used to identify exons within Aca scaf14. Exon/intron boundaries for each gene were predicted manually. If only partial bowfin transcripts were identified, homologous genes and transcripts from other vertebrates (mostly fishes) were collected and used to predict missing peptide sequences. When conserved peptide sequences for a specific gene were absent from bowfin transcriptomes, TBLASTN searches were conducted to identify corresponding fragments in the genomic region. In general, BLASTN searches were used with identified nucleotide sequences to obtain exact genomic positions of exons as well as any homologous genes/exons present in the bowfin genome.

The immune transcriptome and exon sequences on Aca scaf14 were used to infer the protein sequences encoded by these genes. Using the identified nucleotide and/or protein sequences encoded on Aca scaf14 as queries for BLASTN or BLASTP analyses, homologs were identified in human, zebrafish and spotted gar. Human and zebrafish genes with the lowest and/or near the lowest e-values from BLAST searches were located on human chromosome 6 and zebrafish chromosomes 15, 16, and

19, and, in many cases, they were considered as orthologs. When some ambiguity remained (e.g., human genes identified with the lowest or near the lowest e-values were located on Hsa1, Hsa9, or Hsa19 which contain the MHC paralogous groups), sequences were analyzed by inferring gene trees to determine if the identified bowfin gene was a paralog or ortholog of human/zebrafish genes. MHC class I and class II sequences were aligned with ClustalW[43] and their evolutionary relationships were inferred using maximum likelihood in MEGAX[44] with the complete deletion option, 200 bootstrap replicates, and the best fit substitution model.

6.2.3 MHC Results and Discussion. In bowfin, we identified numerous linked MHC class I, class II, and class III genes on a single pseudochromosome, Aca scaf14, that shares conserved synteny with the human MHC and multiple zebrafish loci (main text Fig. 2; Supplementary Figs. 8-9, Supplementary Table 9). This observation suggests that the class I and class II genes of other non-teleost ray finned fishes are linked, and that the "unlinking" of class I and class II genes in teleosts may have resulted from the teleost genome duplication and subsequent events (e.g., recombination, gene gain/loss, pseudogenization, etc.).

Although all identified bowfin MHC class II (alpha and beta) genes are present in a single cluster on Aca scaf14 (main text Fig. 2; Supplementary Table 10), several class I (alpha) genes are present on other pseudochromosomes (Aca scaf2: 38,351,553-38,358,157; Aca scaf5: 32,640,311-32,642,730; Aca scaf6: 26,898,782-26,902,075; Aca scaf8: 39,209,049-39,217,024; Aca scaf12: 4,105,156-4,123,775, 9,159,909-9,785,271; Aca scaf18: 1,532,678-1,533,916, 13,231,672-13,233,334; Aca scaf49: 30,866-36,567), and B2M is encoded on pseudochromosome Aca scaf18 (Extended Data Fig. 3a). These observations illustrate that duplicated MHC class I genes have been translocated to other genomic regions in bowfin and suggest that similar variations in MHC organization may be present across holosteans. Tight linkage of MHC class I and class II genes is characteristic of tetrapod MHC genes, while dispersed genomic localization of MHC class I genes is typical for teleosts. As such, the bowfin reference genome provides important clues on the evolutionary origin and transition to the teleost type of MHC gene organization.

Teleosts encode up to six different lineages of MHC class I sequences (U, Z, S, L, P and H). All have been described in spotted gar, though some ambiguity remains for the S lineage[8,45,46] (Supplementary Table 10). The U lineage has been identified in spotted gar and all studied teleosts (except a few sexually parasitic anglerfishes[47]) and is the only one with genes that display classical type polymorphisms, although it also includes monomorphic non-classical class I genes. The Z lineage also has been described in all studied teleosts and spotted gar. Z lineage proteins share some characteristics with classical MHC class I genes (e.g., may bind peptide antigens) but display more conservation in their alpha1 and alpha2 domains suggesting a limited capacity for antigen display. In contrast, the nonclassical lineages, S, L, P and H have been identified in some, but not all teleost lineages[45,46].

Here we identify MHC class I sequences of the U, Z, S, P, L, and H lineages encoded within the bowfin genome (Supplementary Fig. 9, Supplementary Table 11). Phylogenetic analyses of MHC class I genes indicate that MHC class I genes of the U lineage as well as the P, Z, and L lineages are present on Aca scaf14, even though many of them are likely pseudogenes because of in-frame stop codons, frameshift mutations, deletions or insertions, and some missing exons. MHC genes/pseudogenes of the P lineage are also located on Aca scaf12, Aca scaf2, and Aca scaf18, while an MHC pseudogene of the L lineage is located on Aca scaf18. MHC genes/pseudogenes of the S lineage are found on Aca scaf5, Aca scaf8, Aca scaf12, and Aca scaf49. Additionally, the recently annotated MHC gene of the H lineage[46] is detected on Aca scaf6. Among MHC class I molecules identified, most are composed of alpha1, alpha2, and alpha3 domains with transmembrane domains and cytoplasmic tails. A few exceptions nonetheless exist: *mhc1uaa*, *mhc1uea*, *mhc1pda*, and *mhc1pfa* seem to contain multiple exons coding alpha1, alpha2, and/or alpha3 domains, but it remains to be tested if these genes encode functional proteins.

In contrast to MHC class I genes, diversified lineages of both alpha and beta MHC II genes are located in a cluster on Aca scaf14 and are not identifiable in other genomic regions (main text Fig. 2;

Supplementary Table 9). A pair of alpha and beta loci are juxtaposed with the same transcriptional orientation for most of the bowfin MHC class II genes, although this organization needs to be confirmed. Until now, MHC class II genes of teleost fishes have been classified into three lineages (DA, DB, and DE) and MHC class II genes of tetrapods have been classified into two lineages (DM and tetrapod classical)[48]. Most of bowfin and spotted gar MHC class II genes identified are distinct from teleost MHC class II genes except those of the DE lineage. Additional bowfin and spotted gar lineages identified here were named as DG, DH, DI, DJ, DK, DL, and DN. Although the DG lineage sequences may be orthologous to teleost DA/DB lineages, it was named here as DG, because they are somewhat different from those of teleost DA/DB lineages (see also[48]). It is nonetheless apparent that holostean fishes possess distinct and diverse lineages of MHC class II genes that are not present in teleosts.

The analyses of the genes identified from one end of Aca scaf14 to the MHC class I genes of the L lineage (main text Fig. 2; Supplementary Table 9) revealed that synteny is well conserved between bowfin and humans, emphasizing the utility of the bowfin genome to infer the ancestral states of early bony vertebrate genomes. Synteny with zebrafish was observed to lesser extent and restricted to regions of chromosomes where one-to-two orthologs corresponded to some genes though one-to-one orthologous correspondences were most commonly observed (Supplementary Fig. 8). Note that the zebrafish classical MHC class I U lineage genes on chromosome 19 display gene content variation[49,50], but only one haplotype is presented in main text Fig. 2.

Due to the teleost genome duplication, it is possible that two co-orthologs exist in teleosts for some bowfin and human genes, but a more common one-to-one mapping in zebrafish suggests that frequently only one of two TGD duplicates has been retained. Comparison of the bowfin and human genomes supports the hypothesis that the ancestral genome of vertebrates exhibited tight linkage among MHC class I, II and III genes. Such a condition was dissolved in teleosts by reciprocal loss of TGD paralogs from the initial two teleost MHC I/II/III paralogons and further chromosomal rearrangements, leading to the apparent dissolution of MHC class I, II and III linkage in teleosts. For example, zebrafish classical MHC class I U lineage genes are encoded on chromosomes 19 and 22, MHC class I Z lineage genes are present on zebrafish chromosomes 1 and 3, and classical MHC class II genes are encoded on zebrafish chromosomes 4, 8, and 18[50,51]. It is also worth noting that numerous orthologs from the human and bowfin MHC regions are present on Dre19 and Dre16 (main text Fig. 2, Supplementary Fig. 8) which are TGD-paralogous chromosomes carrying for example the *hoxAa* and *hoxAb* gene clusters, respectively.

6.3 Immunoglobulin (Ig) and T Cell Receptor (TCR) genes

6.3.1 Ig and TCR Background. Antigen recognition receptors of the adaptive immune system, i.e. immunoglobulin (Ig) genes and T-cell receptor (TCR) genes that undergo V(D)J recombination, have been identified in all lineages of jawed vertebrates. However, their structure and genomic organization can differ between major lineages[52,53]. Most teleosts encode three classes of antibodies [IgM, IgD and IgT (a.k.a. IgZ in zebrafish)] and canonical TCR loci (TCRα, TCRβ, TCRγ, and TCRδ). We previously reported a single, tightly linked TCRα/δ locus in spotted gar[8].

6.3.2 Ig and TCR Methods. BLAST searches[42] using immunoglobulin (Ig) and T cell receptor (TCR) constant gene segments of spotted gar and other vertebrates as queries identified bowfin pseudochromosomes encoding various types of antigen recognition molecules of the adaptive immune system using the strategy described in 6.2.2. The immune transcriptome was utilized to clarify gene structures. In addition, a custom program was used to locate potential recombination-signal sequence (RSS) neighboring V, D, and J gene segments of immunoglobulin (Ig) and T-cell receptor (TCR) genes by identifying heptamer/nanomer motifs with 12/13 or 22/23 bp spacers. Deduced amino acid sequences of Ig and TCR were aligned using ClustalW[43] and analyzed to infer their phylogenetic relationships in MEGAX[44]. The best fitting amino acid substitution models (see figure legends) were used to infer phylogenetic relationships in a maximum likelihood framework with complete deletion options and 200 bootstrap replicates.

6.3.3 Ig and TCR Results and Discussion. Our investigation of the bowfin genome revealed a single TCRα/δ locus, a single TCRβ locus, and a single TCRγ locus (Extended Data Fig. 3a-c). We identified a single Ig heavy (IgH) chain locus (Extended Data Fig. 3a) and a few Ig light (IgL) chain loci including an Igκ locus (Extended Data Fig. 3d) and Igσ loci (Extended Data Fig. 3e). The IgH chain locus and the TCRα/δ locus are linked on a single pseudochromosome (Aca scaf18), separated by approximately 12 Mb (Extended Data Fig. 3a). Except for a few IgL chain loci, the Ig loci (including the Igκ locus), and TCR loci exhibit translocon type gene organization, i.e., tandem duplications of V, D, J gene segments and C exons represented by Vn-(Dn-)-Jn-C (Extended Data Fig. 3).

Immunoglobulin heavy chain (IgH) genes: One notable feature of the IgH locus on Aca scaf18 is the presence of three different types of constant gene segments. The two constant gene segments located closer to the TCRα/δ locus are of a Cμ and Cδ type, while another constant gene segment is present at the other end (Extended Data Fig. 3a). This other constant region consists of four Ig domains (C1 - C4). Maximum likelihood inference of the phylogenetic relationships of this sequence with other vertebrate Ig constant regions with four Ig domains (Supplementary Fig. 10a**,** Supplementary Table 12) indicated a closer affinity of this novel bowfin C region to the Cτ (Cζ in zebrafish) constant region of teleost fish. Likewise, separate phylogenetic analyses of the C1 and C4 domains with C1 domains and with the carboxyl-terminal constant Ig domains (respectively) from a range of immunoglobulins and species suggest an affinity of the bowfin C4 gene segment to the Cτ/Cζ of teleosts at the carboxyl-terminal domain (Supplementary Fig. 10c, Supplementary Table 12). On the other hand, they suggest an affinity to Cω (of the chondrichthyan sandbar shark) at the C1 domain (Supplementary Fig. 10b, Supplementary Table 12), though with weak support (BSS =33%). Our results also indicate that teleost fish Cτ have an affinity with teleost C1, suggesting past genetic exchange (i.e., exon shuffling, gene conversion, etc.) between these domains in teleosts. We further determined that an ortholog of this Cτ constant gene segment found in teleosts and bowfin is in fact present in the spotted gar genome on an unplaced scaffold JH591552.1 (Supplementary Fig. 10), despite being considered absent in earlier analyses[8]. As this C gene segment found in both bowfin and spotted gar likely represents an ancestral homolog of the teleost Cτ, we consider it a Cτ constant gene segment that dates back at least to the common ancestor of crown neopterygians. Our conclusion based on bowfin genomic analyses is consistent with a recent investigation of bowfin transcriptome data[54]. These observations thus indicate that IgT-like antibodies arose prior to the origin of teleosts and raise the question about the function of IgT. Further studies in bowfin, gars, and other non-teleosts will be necessary to clarify if IgT mucosal host defense[55-57] is a derived feature of teleosts or a more universal role.

Our results raise the question of whether the mu, delta, or tau type of IgH utilize the entire repertoire of V gene segment of the IgH locus. The presence of non-IgH genes (e.g., Ig superfamily proteins, C-type lectin domain family 4, phosphatidylinositol-4,5-bisphosphate 4-phosphatase 1, and immunoglobulin light chain) in the middle of V gene segment cluster of IgH locus may interfere with the utilization of distantly located V gene segments and allow only the utilization of proximal V gene segments for each isotype.

Immunoglobulin light chain (IgL) genes: Using sequences from multiple vertebrate lineages, including the genome of the spotted gar and transcriptomes from saddled bichir (*Polypterus endlicheri*) and ropefish (*Erpetoichthys calabaricus*), five ancient vertebrate IgL isotypes have been described: kappa (κ), lambda (λ), lambda-2 (λ-2), sigma (σ), and sigma-2 (σ-2; previously sigma-cart)[58]. Spotted gar was reported as the only known species to possess all five isotypes (although the spotted gar IgL lambda sequence was reported as a pseudogene). Accordingly, we identified evidence for all five isotypes of bowfin immunoglobulin light chain loci on different pseudochromosomes (Supplementary Fig. 11, Supplementary Table 13). Among them, an IgL kappa locus (Aca scaf11: 2,079,706 -2,103,176, 5,091,825-7,188,393) contains large and extensive numbers of V gene segments under a translocon type gene organization, likely representing the primary immunoglobulin light chain of the species, together with IgL sigma loci (Aca scaf22: 14,393,451-14,401,326, 16,948,268-16,963,957) (Extended Data Fig. 3d,e).

Bowfin IgL sigma-2 sequences can be identified in the middle of the IgH locus (Aca scaf18) and shares homology with sigma-2 sequences in shark and spotted gar. Although bowfin sequences similar to IgL lambda (Aca scaf15: 25,075,577-25,090,458) and lambda-2 (Aca scaf1: 30,303,148-30,306,638) can be identified, the presence of in-frame stop codons (in both genome and transcriptome sequences) in the single J gene segment in the lambda locus and in the single V gene segment in the lambda-2 locus indicates that, unless these sequences are properly mutated at the somatic level, functional protein products would not be generated. Further investigation is required to determine the functionality of all five IgL lineages in bowfin and gar.

T cell receptor (TCR) genes: Three bowfin loci encoding four different types of TCRs were identified: TCRα/δ (Aca scaf18: 22,416,394-23,841,556), TCRβ (Aca scaf21: 27,399-316,845), and TCRγ (Aca scaf4: 38,763,058-38,840,380). Schematic representations of TCR loci with translocon type gene organization are shown in Extended Data Fig. 3. This mirrors TCR lineages in other vertebrates (e.g. see human, frog, and catfish TCR lineages in Supplementary Fig. 12, Supplementary Table 14). In bowfin, extensive diversity is observed for the V and J gene segments of the TCR Vα/δ gene segments and the Jα gene segments, as well as moderate diversity for Vβ and Vγ gene segments. Our finding of four different types of TCRs are consistent with expectations from the hypothesized early evolutionary origin of distinct sets of TCR subunits and their functional importance among vertebrate species[59].

6.4. Toll-like receptors (TLRs)

6.4.1 TLR Background. Toll-like Receptors (TLRs) are present in both vertebrates and outside vertebrates and provide one of the initial immune responses to infection through recognition of a variety of pathogen-associated molecular patterns (PAMPs)[60,61]. Six major families of TLRs have been described (TLR1, TLR3, TLR4, TLR5, TLR7, and TLR 11) with different species possessing varying numbers of genes within each family. Human and mouse encode ten and twelve TLR genes respectively. However, teleost genomes can encode more than 20 TLR genes[50,60,62]. Although the larger number of teleost TLRs could be the result of the teleost genome duplication, a total of 16 TLR genes plus one pseudogene were reported from spotted gar representing all six TLR families[8,63]. Therefore, determining the extent of TLR diversity in bowfin is critical to further polarizing the changes in TLR diversity following the teleost genome duplication.

6.4.2 TLR Methods. Bowfin Toll-like receptors (TLRs) were identified via TBLASTN searches using the amino acid sequences of well-established TIR domains[63] and annotated based on clustering within the phylogenetic treeas. TIR domains were aligned via Clustal Omega[64] with priority given to TIR domains identified from the MAKER genome annotation, followed by sequences from the PhyloFish database[65], the immune tissue transcriptome, and significant hits in the genome. The evolutionary relationships among TLR receptors were analyzed using the maximum likelihood approach available in IQ-TREE 2[66]. Analyses were conditioned on the best-fit model of protein evolution identified by IQ-TREE from a candidate pool of models that spanned all common amino-acid exchange models to protein mixture models. We conducted 1,000 ultrafast phylogenetic bootstraps [67,68] to assess support for evolutionary relationships.

For genomic loci in which BLAST identified a TIR domain but lacked a gene model, we scanned upstream sequences for the presence of leucine-rich-repeats (LRR). 50 kb upstream of each TIR domain was translated (3-frame) into amino acid sequences and searched for leucine-rich repeats (pfam clan CL0022) using HMMER3[69]. In all cases at least one LRR was found, supporting that these “orphan” TIR domains belong to unannotated TLR genes and not to other genes that utilize TIRs (e.g., *myd88*).

6.4.3 TLR Results and Discussion. Here we report 20 bowfin TLRs annotated based on phylogenetic clustering with defined TLRs from other species (Supplementary Fig. 13). TLR4, TLR9, TLR20, TLR21 and TLR25 appear to be duplicated in bowfin. In four of these instances (TLR4, TLR9, TLR20, and TLR21), the duplications are found in close proximity on the same pseudochromosome suggesting

tandem gene duplications (Supplementary Table 15). TLR25a and TLR25b are almost 10 Mb apart on Aca scaf14 suggesting a different mechanism of duplication.

In spotted gar, TLR1, TLR2, and TLR3 are found on the same chromosome (LG4). Bowfin TLR2 and TLR3 are found on the same pseudochromosome Aca scaf12, and bowfin TLR1 is isolated on pseudochromosome Aca scaf11. The duplication of TLR8 observed in spotted gar was not detected in bowfin, but bowfin TLR7 and TLR8 are found in close genomic proximity as observed in spotted gar and other vertebrate species[63]. Bowfin appears to encode six members of the TLR11 subfamily, while only one was previously reported in spotted gar, further showing the diversity of TLR repertoires even among holosteans (Supplementary Fig. 13, Supplementary Table 15). Bowfin has an ortholog to a previously identified and highly diverged gar TLR which was originally named TLR19 or TLR19-like[8,63]. Due to the identification of an authentic TLR19 in bowfin (Supplementary Fig. 13), the uncertainty as to which TLR subfamily this previously identified sequence belongs, and the apparent restriction of this sequence to holosteans, we refer to this gene as TLR-HS (holostean-specific), providing another line of evidence in support of holostean monophyly.

Supplementary Note 7. SCPP genes and scale formation

7.1 Methods. We investigated differences in the neopterygian repertoire of SCPP genes in bowfin, gar, and selected teleosts to reveal SCPP genes that are important for scale formation in Holostei and other actinopterygians. We searched for the bowfin genomic regions syntenic to the two spotted gar SCPP gene clusters and gar *scpplpq20* using TBLASTN[42] with gar SCPP amino acid sequences[8,70] as queries. All intron-spanning reads in these syntenic regions and adjacent regions (within pseudochromosomes 9, 12, and 22) identified using RNA-seq data were investigated to identify SCPP genes that possess a characteristic exon-intron structure, including the presence of entirely untranslated exon 1, all phase-0 introns, and the entire signal-peptide encoding region in exon 2[71]. For these bowfin genomic regions, we also used GENSCAN [http://hollywood.mit.edu/GENSCAN.html] to detect exons directly from the genomic sequence.

To analyze SCPP gene expression during zebrafish skin development, relative expression levels were estimated using Galaxy[72] for published skin RNA-Seq datasets[73], trimmed using Trimmomatic[74], and aligned to the zebrafish genome sequence (danRer11) using STAR[75]. Three different sets for each age from NCBI SRA (5 months: SRR850591, SRR850594, SRR850597; 24 months: SRR850598, SRR850601, SRR850603; 42 months: SRR850604, SRR850607, SRR850608) were merged. Genomic coordinates of SCPP genes were determined using Splign[76]. FPKM values were calculated using Cufflinks[77] based on BAM files obtained from STAR. Galaxy analyses used default conditions except additional options in Trimmomatic (ILLUMINACLIP and MINLEN=50) and Cufflinks (multi-read correction).

7.2 Results and Discussion. In the bowfin genome, we identified 22 SCPP genes (Supplementary File 1), of which 21 genes form two large clusters. The arrangement of these bowfin SCPP genes is similar to that of gar SCPP genes (main text Figure 3). One cluster on bowfin 12/gar LG4 consists of two subclusters of SCPP genes; genes in one subcluster encode Pro and/or Gln (P/Q)-rich SCPPs, and genes in the other subcluster encode acidic SCPPs. These two subclusters are separated by *sparcl1*, which is evolutionarily related to SCPP genes[70]. Genes in the other cluster on bowfin 9/gar LG2 encode one acidic SCPP (Spp1) and three or more P/Q-rich SCPPs (main text Figure 3). The only exception is *scpplpq20* that is isolated from these two SCPP gene clusters on bowfin 22/gar LG22.

The total number of SCPP genes identified in the bowfin genome is considerably smaller than that found in the gar genome, in which we detected 38 SCPP genes, the largest number of SCPP genes found to date[8,70]. All 22 bowfin SCPP genes have orthologs in the gar genome, but the orthologs of 16 gar SCPP genes were not detected in the bowfin genome (main text Figure 3). This result implies that various

SCPP genes were secondarily lost in the bowfin lineage and/or newly duplicated in the gar lineage. Among the 16 gar SCPP genes for which no bowfin ortholog was identified, nine gar genes (*scpplpq9*, *scpplpq10*, *scpplpq18*, *scpplpq11*, *scpplpq19*, *scpplpq12*, *scpplpq13*, *scpplpq15*, and *scpplpq17*) are clustered on LG2 (syntenic to the SCPP gene cluster on bowfin 9; main text Figure 3). Notably, our previous RT-PCR study showed that expression of all these gar SCPP genes except for *scpplpq14* and *scpplpq16a* was weak or undetectable in tooth germs but strong in the skin that includes scale-forming cells[8]. This result suggests that many of these SCPP genes clustering on gar LG2 are involved in scale formation, but not in tooth formation. To test this hypothesis, we examined P/Q-rich SCPP genes of three phylogenetically close teleost species, zebrafish, Mexican tetra (cavefish), and channel catfish, of which zebrafish and cavefish have scales but channel catfish secondarily lost scales[78].

It was previously reported that *scpp5*, among P/Q-rich SCPP genes, is involved in scale formation in teleosts, because *scpp5* is absent in the scaleless three-spine stickleback and became a pseudogene in the scaleless channel catfish but is present in two scaled catfish species[78]. However, we identified apparently functional *scpp5* genes in both three-spine stickleback, channel catfish, and the scaleless electric eel (Supplementary Fig. 14a). Moreover, our transcriptome analysis revealed no significant expression of *scpp5* in the skin of various ages of zebrafish (Supplementary Table 16). For these reasons, we concluded that *scpp5* is not essential for scale formation in teleosts.

Unlike in teleosts, however, *scpp5* encodes a ganoin matrix protein and is expressed during ganoid scale formation in gar and bichir[79]. In addition, neither *scpp5* nor *enam* is detected in the sterlet and paddlefish genomes[80] which is consistent with their reduced scales and teeth[81,82]. Further studies, however, are needed to identify tissues composing scales and teeth of sterlet and paddlefish.

Liu et al. (2016) further argued that *scpp1* is also critical to scale formation, because of its presence in the common pleco genome, a scaled catfish, and its absence from genomes of the scaleless channel catfish and the scaleless electric eel[78]. Upon our re-examination, we also identified *scpp1* in the channel catfish and electric eel genomes (Supplementary Fig. 14b), and *scpp1* genes are present in the genomes of sterlet sturgeon and paddlefish[80] that have reduced scales. Thus, our findings do not support the conclusion by Liu et al. (2016) that loss of *scpp5*, *scpp1*, or both would cause the scaleless phenotype across actinopterygians.

Instead, the presence and absence of SCPP genes in scaled zebrafish and cavefish and in scaleless channel catfish suggest that *scpp11a*, *scpp11b*, *scpp13*, *scpp14*, *gsp37*, and *scpp12* clustered on zebrafish chromosomes 5 and 10, previously shown to be TGD duplicate clusters of the SCPP gene cluster on gar LG2[8], are potentially important for scale formation. This is consistent with our transcriptome analysis that confirmed expression of all these genes in zebrafish skin (Supplementary Table 16). Furthermore, *gsp37* is known to encode a scale matrix protein in goldfish[83]. The evolutionary rate of all these genes is extremely high[70]. Thus, it is difficult to determine orthologies even between the relatively closely related zebrafish and cavefish (*Astyanax*) and not possible to clearly identify orthologies of these SCPP genes between holosteans and teleosts. However, the presence of twelve gar genes encoding P/Q-rich SCPPs, clustered on LG2, and nine missing orthologs of these twelve gar SCPP genes on bowfin 9 agree with the hypothesis that genes encoding P/Q-rich SCPPs involved in scale formation are clustered on LG2, and that these genes were secondarily lost from the bowfin genome, along with modifications of scale formation in bowfin. This hypothesis is consistent with the highly reduced repertoire of P/Q-rich SCPP genes in the genomic regions of scale-reduced sterlet and paddlefish[80] that are syntenic to the SCPP gene clusters on gar LG2 and bowfin pseudochromosome 9.

In summary, our comparative analysis of holostean SCPP genes reveals the gene cluster on gar LG2 and bowfin pseudochromosome 9 as a putative hot-spot for scale phenotype variation across ray-finned fishes and generates testable hypotheses for future functional investigations.

Supplementary Note 8. ATAC-Seq chromatin profiling of bowfin development

Results and Discussion. ATAC-Seq data are available in NCBI under SRA accession SRP281665. The inferred fragment size distribution (Supplementary Fig. 15a) confirm high library quality and illustrates nucleosome periodicity with peaks occurring around 200, 400, and 500 bp representing mononucleosome, dinucleosome, and trinucleosome sizes, respectively[84]. We find a total of 172,276 open chromatin regions (OCRs) when merging bed files of all stages with 1bp+ overlap, 140,902 of which (81.8%) were retained as non-coding OCRs (ncOCRs) that are candidates for gene regulatory regions through development. 70.8% (122,055/172,276) of OCRs and 69.4% (97,847/140,902) of ncOCRs are found in at least 2 developmental stages (Supplementary Table 17). OCR similarity between stages quantified with Jaccard indices show that OCR and ncOCR profiles for each stage are most similar to those in the adjacent developmental stage and biological replicate (Supplementary Fig. 15b,c). This illustrates gradual changes in chromatin accessibility through development as well as the consistency and robustness of our dataset. OCR and ncOCR results are summarized in Supplementary Tables 17-18. See Supplementary Table 19 for HOMER[85] annotation results of bowfin OCRs.

To explore the conservation of bowfin OCRs and to establish overlap of bowfin OCRs with genetic element annotations from other vertebrate species' studies and databases, we generated a Progressive Cactus[86] whole genome alignment including bowfin, gar, zebrafish, human, and mouse and established orthologies of genetic elements among these species. Conservation across species and overlap between bowfin OCRs and these elements extracted with the halLiftover tool[86] and BedTools[87] are summarized in Supplementary Tables 20-24. Results described in the main text and here are reported for conserved reference elements (50bp+ in length after liftover) with 33%+ of the reference element overlapping with the target element.

Bowfin ncOCR conservation across bony vertebrate species lets us define a set of 3,844 core vertebrate ncOCR loci present in holosteans (gar), teleosts (zebrafish), and mammals (human, mouse). 59,003 ncOCRs are conserved only with gar (putative holostean-specific elements), 6,245 conserved only with gar and zebrafish (putative neopterygian-specific elements), and 2,604 elements are conserved in all species except zebrafish, suggesting secondary loss or divergence beyond recognition within the teleost lineage leading to zebrafish (Supplementary Fig. 16).

We used conserved non-coding elements (CNEs) established for the gar genome (as defined in ref.[8] see also Methods), bowfin ultraconserved elements (UCEs) (as defined in ref.[33]; see also Methods), experimentally confirmed enhancer elements from human and mouse available from the Vista Enhancer Browser[88], as well as mouse embryonic single nucleus OCRs[89] to establish overlap of bowfin OCRs/ncOCRs with established, well-characterized genetic elements from other vertebrate species (see Supplementary Tables 21-24).

Using the gar CNE ancestry categorization from Braasch et al. (2016)[8], we find that CNEs conserved from the gnathostome ancestor (GCNEs) more often intersect a bowfin ncOCR (38.8%) than those that emerged later in the bony vertebrate ancestor (BCNEs; 29.2%) or in the ray-finned fish lineages (RCNEs, 25.2%) ($P < 0.00001$, chi-square test; Supplementary Table 21). This suggests that ancestry of sequence conservation is predictive of developmental chromatin accessibility in bowfin. Reanalyzing the phylogenetic distribution of vertebrate CNEs from Braasch et al. (2016)[8], we further identified 8,384 "fish-specific" CNEs (FCNEs) that have been lost secondarily in the lineage leading to tetrapods. 35.0% of these FCNEs overlap bowfin ncOCRs (Supplementary Table 21), suggesting that they harbor important gene regulatory elements with fish-specific developmental functions, but are putatively dispensable or substantially changed for terrestrial life.

For both mouse VISTA enhancers (Supplementary Table 22) and mouse embryonic single nucleus OCRs (Supplementary Table 24), we find a low level of sequence connectivity to the bowfin genome. This is likely due to the particularly high rate of molecular sequence evolution in the mouse lineage[90], which would also explain the difference in conservation in bowfin of mouse VISTA enhancers (15.9%) when

compared to human (60.2%) (Supplementary Table 22). Furthermore, since the published genome-wide mouse OCRs were defined by ATAC-Seq without taking sequence conservation into account[89], we expect a large fraction of them to be mouse-lineage specific. Nevertheless, we located the orthologous position for a total of 10,035 murine ncOCR elements in the bowfin genome, of which 2,261 (22.5%) reside in bowfin ncOCRs (Supplementary Table 24). These bowfin ncOCRs are thus putatively cell/tissue type-specific, attributable to the development of e.g., spinal cord (244), forebrain (151), mid/hindbrain (151), neuro-mesodermal progenitor cells (128), somatic mesoderm (70), somatic mesoderm (57), neural crest (50), etc. (Supplementary Table 24). Together with the 338 human and 70 mouse VISTA enhancers overlapping bowfin ncOCRs (Supplementary Table 22), these elements are prime candidates for experimental validation with reporter assays in fish and tetrapods.

Supplementary Note 9. Bowfin informs the evolution of the *tbx4* 'lung' enhancer

9.1 Background. The evolutionary origin and homology of bony vertebrate air-filled organs (AOs) in form of e.g., tetrapod lungs, respiratory gas bladders as in bowfin and gar, and teleost swim bladders has been debated since the days of Owen and Darwin[91,92]. For example, it was proposed that an ancestral lung with a dual function as both a respiratory and a hydrostatic organ evolved before the rise of bony vertebrates (but AOs are not found in cartilaginous fishes or cyclostomes). While the respiratory function was intensified in tetrapods in form of the lung, the hydrostatic function became more elaborate in teleosts in form of the swim bladder for buoyancy control[93]. Lungs in tetrapods and lungfishes as well as in the basal ray-finned lineage of polypterids (bichirs, reedfish) develop on the ventral side of the gut, while teleost swim bladders as well as the gas bladders of gars and bowfin develop on the gut's dorsal side. Therefore, it has also been suggested that these organs could have originated independently from a respiratory posterior pharynx in the bony vertebrate ancestor that then evolved into a dorsal respiratory gas bladder in ray-finned fishes and into ventral lungs in tetrapods. The respiratory function of the dorsal gas bladder would later be lost in many teleosts after divergence from holosteans[94].

Support for the homology of tetrapod lungs and teleost swim bladders comes from molecular and developmental studies that recognized similarities between transcriptional profiles of the adult zebrafish swim bladders and adult mammalian lungs[95], co-expression of genes during early zebrafish swim bladder development that also interact during tetrapod lung formation[92], and expression similarities of adult ray- and lobe-finned fish AOs with terrestrial lungs[35,96,97]. We (EF and ARM) have recently analyzed the developmental morphogenesis of the bowfin gas bladder using candidate gene approaches, differential RNA-Seq, and nano-CT scanning[98-100]. We found that bowfin gas bladder development starts at Ballard stage 25, budding from the dorsal foregut[100]. Our gene expression analyses have shown that key genes of the Fgf, Bmp, and Tbx signaling pathways known to be expressed during ventral lung development in tetrapods are also expressed during dorsal gas bladder development in bowfin[98,99]. This suggests a ventral-to-dorsal inversion of the AO gene regulatory network in an ancestor of neopterygians fishes after divergence from the more basally branching polypterid lineage which expresses these genes during the formation of their ventral lungs[98,99,101].

T-box genes *tbx4* and *tbx5* encode key transcription factors with a ventral-to-dorsal expression inversion in neopterygians compared to earlier branching lineages[98,99]. In mouse, an enhancer region located in the third intron of the *Tbx4* gene drives *Tbx4* expression into the early lung bud, where it controls lung bud formation[102]. This 'lung mesenchyme enhancer' (LME) region shows conservation among tetrapods, but is also conserved in coelacanth[103,104], which possesses a fat-filled lung-like organ[105], as well as in bichir, where LME likely functions as a lung enhancer[101]. We and others have shown that the LME is also conserved in holosteans[8,35,101], but no conservation has previously been demonstrated with teleosts that possess swim bladders[35,101,103,104,106,107]. Other non-coding elements in the *tbx4* gene region that are conserved across teleosts but not with lobe-finned vertebrates, in contrast, were previously hypothesized

to represent teleost-specific 'swim bladder enhancers'[103]. Here, we analyzed chromatin accessibility of the *TBX4* gene region in the human fetal lung to compare to our bowfin OCRs and examined conservation of *Tbx4* non-coding regions across bony vertebrates.

9.2 Methods. Atlases of human fetal single cell RNA-seq[108] and single cell chromatin accessibility[109] were surveyed at https://descartes.brotmanbaty.org/ to investigate *TBX4* expression and OCRs in different cell populations of the developing human lung compared to other tissues and organs.

The *tbx4* gene regions of bowfin (AmiCal1; Aca_scaf_22), gar (LepOcu1; LG22), stickleback (BROADS1; groupI), Japanese puffer (GCA_901000725.2; chr1), medaka (HdrR ASM223467v1; chr13), pike (Eluc_v4; LG01), zebrafish (GRCz11; chr15), coelacanth (LatCha1; JH126567), western clawed frog (Xenopus_tropicalis_v9.1; chr2), chicken (GRCg6a; chr19) and human (GRCh38; chr17) were aligned with mVISTA[110] [http://genome.lbl.gov/vista/mvista/submit.shtml] using Shuffle-Lagan[111] (calculation window: 75bp, minimum conservation width: 75bp; conservation identity: 65%). Repeat-masked sequences and gene annotations were downloaded from Ensembl [http://ensembl.org; Release 100].

9.3 Results and Discussion. As shown in Extended Data Fig. 4a, in the developing lung of the human fetus, an OCR is found at the location of the LME lung enhancer, specifically in vascular epithelial and stromal cells which also express *TBX4* (Extended Data Fig. 4b). In contrast, no LME OCR is seen in other lung cell types such as the bronchiolar and alveolar epithelial cells (Extended Data Fig. 4a) nor in the vascular epithelial or the stromal cells in other organs (e.g., intestine, Extended Data Fig. 4a). This suggests that the LME has a cell type- and organ-specific function during mammalian lung development.

To connect these human LME data to bowfin, gar, and teleosts, we generated a series of VISTA sequence conservation analyses (Extended Data Fig. 4c). In a human-centric VISTA plot (Extended Data Fig. 4c, top), we find that conservation of the 'lung enhancer' extends to holosteans because a conservation peak is found within the third intron of bowfin and gar *tbx4*, providing evidence for the presence of the LME in the common ancestor of bony vertebrates.

Based on a stickleback-centric VISTA plot (Extended Data Fig. 4c, bottom), we further find evidence for the presence of one of the three previously described putative 'swim bladder' enhancers[103] (SBE2) in bowfin. This region is found in stickleback *tbx4* intron 3 in addition to two other regions conserved among neopterygians, but that appear to be not conserved with tetrapods. We name these two other apparently ray-finned-specific regions 'R1' and 'R2'.

In the bowfin-centric VISTA plot (Extended Data Fig. 4c, middle; main text Fig. 4f), however, it becomes apparent that in intron 3 of bowfin *tbx4*, the LME lung enhancer in the human-centric alignment (Extended Data Fig. 4c, top) and the region R1 defined by the stickleback-centric alignment (Extended Data Fig. 4c, bottom) in fact overlap in the bowfin genome. Hence, here for the first time, we show that the LME and R1 elements must be considered orthologous between tetrapods and teleosts. The hidden orthology of LME from tetrapods to teleosts thus only becomes apparent by using the 'Holostean Bridge' principle (Extended Data Fig. 4c, right).

As we have shown previously, bowfin *tbx4* is expressed in the developing gas bladder, starting with strong expression in the dorsal and ventral mesoderm during the budding phase (stage 25) and then increasing dorsal expression during the outgrowth phase (stage 27), generating a dorso-ventral gradient[98]. Importantly, our ATAC-Seq profile of *tbx4* here shows that the LME region is indeed accessible during that time (main text Fig. 4g) suggesting a regulatory role for *tbx4* expression in the developing bowfin gas bladder. Of note, putative swim bladder enhancer region SBE2[103] is characterized by open chromatin in bowfin as well (main text Fig. 4g).

The example of the *tbx4* LME highlights the power of the holostean bridge to connect cryptically orthologous non-coding elements from tetrapods to teleosts (Extended Data Fig. 4c, right). It further supports deep homology of *tbx4* gene regulation during bony vertebrate AO development, leading us to postulate that *cis*-regulatory elements embedded in the third intron of *tbx4* provide further molecular

evidence for the homology of tetrapod ventral lungs, holostean dorsal respiratory gas bladders, as well as teleost dorsal swim bladders. This hypothesis awaits further functional examination of neopterygian intron 3 sequences. Intriguingly, LME is also conserved in some shark species[106,107]. Cartilaginous fishes may have vestigial AOs[94], which could suggest that the LME function even dates back to the gnathostome ancestor[107].

Supplementary Note 10. Hox gene cluster analyses

10.1 Methods. Bowfin Hox cluster genes were manually annotated using spotted gar *hox* gene orthologs, including the *hoxd14* pseudogene[8]. Manual curations were performed in Geneious 9.1.8, supported by AUGUSTUS[112] *ab initio* gene predictions on the Hox cluster regions, and following annotation strategies used for other fish Hox gene clusters[113,114]. For the annotation of the *hoxd14* gene structure, we also leveraged information from aligned fin transcriptome RNA-seq reads and cloned and sequenced *hoxd14* transcripts.

10.2 Results. Predicted bowfin *hox* gene transcripts are provided in Supplementary File 2. Except *hoxd14*, all holostean *hox* genes are complete including start/stop codon; canonical splicing sites; two coding exons, one intron; and homeodomain sequence, including three alpha helixes in the second coding exon (main text Fig. 5).

Bowfin *hoxD14*: The presence of the bowfin *hoxd14* pseudogene is supported by fin bud RNA-seq and whole embryo ATAC-Seq data (Supplementary Fig. 17). Bowfin *hoxD14* was amplified from stage 26 fin bud RNA using the following primer set:

AcHoxD14_F1: 5'-CCTTCGTCTCTGACCTCTGG-3'
AcHoxD14_R1: 5'-AAGAGGCAATTGGCACATTC-3"

➔ AcHoxD14_F1 + AcHoxD14_R1 = 545 bp product (for ISH)

We cloned two different partial splice variants of bowfin *hoxd14* as shown below (alternating color boxes refer to exons in main text Fig. 5b; grey shaded sequence indicates difference between the long and short variant).

>Aca_HoxD14_long

CCTTCGTCTCTGACCTCTGGATGTCTGAAAACCGCTGCGGACAAGAGGTTTGGGGATTTTCGTGATGTTTTA
GCGTATATGGTTACTCGAAAAAGGAAGTGGATGCATCTCTGCCCCAGGCTCCTACAAACTTTAAAACATATG
CGTGCTGCAGACAGTACTATATATCTTACAGTAATATATCTTCTTTATCTAGACCAAACACCTATAATCGGAG
CGAATACCCTCCACGTCCTTCCATATATCATCAGTGGGCGTTTGAACACCCTCGGGGGTCTTCTGCCGTCTC
AGACCAG CTTTAGGATCCCCTCGATATGGGTCTCAAAGTACACCTGCGAAGTGAGAAGAAAAGGACCCATT
AATAGCAAATAGCAGATCGCTGGATTGTAACTGGAATTTGAGAGGAATAATTTTCTCACTCCCAAAAATTTCA
TTAAAACAGAAAGACAG GTTAAACTGTGGTTTCAGAACCAGAGACTGAAACAGAAAAAGTTTCTTCGAGTTC
CACTCACAAAACCCAGTTTAATTTGAGCTGCACTTATGTTTTTCAAGAAATGCTTTTAGGCGGTACTATTTTCC
CAGTGTATAAATGCGTCTTCGAAATTGATAAAGTTCGTATGTGTCGCGATTAACATGTTTTAAATGAGAAACA
TCTGCATGTAAGCGTACATTTGAATGTGCCAATTGCCTCTT

>Aca_HoxD14_short
CCTTCGTCTCTGACCTCTGGATGTCTGAAAACCGCTGCGGACAAGAG ACCAAACACCTATAATCGGAGCG
AATACCCTCCACGTCCTTCCATATATCATCAGTGGGCGTTTGAACACCCTCGGGGGTCTTCTGCCGTCTCAG
ACCAG CTTTAGGATCCCCTCGATATGGGTCTCAAAGTACACCTGCGAAGTGAGAAGAAAAGGACCCATTAA
TAGCAAATAGCAGATCGCTGGATTGTAACTGGAATTTGAGAGGAATAATTTTCTCACTCCCAAAAATTTCATT
AAAACAGAAAGACAG GTTAAACTGTGGTTTCAGAACCAGAGACTGAAACAGAAAAAGTTTCTTCGAGTTCC
ACTCACAAAACCCAGTTTAATTTGAGCTGCACTTATGTTTTTCAAGAAATGCTTTTAGGCGGTACTATTTTCCC
AGTGTATAAATGCGTCTTCGAAATTGATAAAGTTCGTATGTGTCGCGATTAACATGTTTTAAATGAGAAACAT
CTGCATGTAAGCGTACATTTGAATGTGCCAATTGCCTCTT

Spotted gar *hoxD14*: In an attempt to clone *hoxd14* cDNA from spotted gar, RNA was extracted from Long & Ballard[115] gar stages 27, 28, 29, and 30 whole embryos, and from pectoral and caudal fin tissue dissected from stage 30 animals, using the RNeasy Plus Micro Kit (Qiagen). Template cDNA libraries were produced using the SuperScript IV Reverse Transcriptase System (Invitrogen). PCR primers were designed based on the bowfin *hoxD14* transcripts. The three left and three right primers below were used in all possible left-right pairwise combinations for PCR amplification. No primary reaction amplified a *hoxD14* cDNA, so secondary reactions were performed with nested primers using the primary PCRs as templates. All possible nested reactions also failed to amplify a *hoxD14* cDNA. However, such nested PCR approach was used to successfully amplify *sp8* from spotted gar (see below). Failure to clone *hoxd14* cDNA is consistent with the absence *hoxd14* transcripts in gar RNA-seq data[8].

LoHoxD14_L1: 5'-TTGGATCCAGTTTCTCCACA-3'
LoHoxD14_L2: 5'-GGAGTTCCTTTGTCGTCACC-3'
LoHoxD14_L3: 5'-TGATATTTTGCGGTGTGGAA-3'
LoHoxD14_R1: 5'-GGCTATTTTCTGCTGCGTTT-3'
LoHoxD14_R2: 5'-CAGACACATGCAAACCCAAC-3'
LoHoxD14_R3: 5'-AGCCATAGGCCTGCATACAA-3'

Supplementary Note 11. Fin development analyses

11.1 Collection of bowfin embryos. Bowfin embryos (unsexed) were collected from nests in Oneida Lake, New York. Eggs attached to nest material were collected in lake water and methylene blue was added to abate fungal growth. Once in the lab, eggs were separated from nesting material by hand and placed in fresh lake water. Eggs and embryos were raised in static containers of lake water and moved to fresh water every other day. Embryos and larvae were sampled at the relevant stages following Ballard's bowfin staging series[116] (Extended Data Figure 5c).

11.2 Fin transcriptomics quality control. One of the two stage 23, 75 bp single-end samples had very low complexity compared to the other libraries likely due to low RNA input. We removed this sample from our analysis, and results for the stage 23 fin bud transcriptome are based on the two remaining samples (1x 150 bp paired-end, 1x 75 bp single-end sample).

To examine possible batch effects, we performed PCA analysis using the prcomp() function in R. In analysis of the full nuclear transcriptome dataset, clear groupings based on developmental stage or sequencing strategy were not resolved by PC1 or PC2 (Supplementary Fig. 18a). Given that these samples come from wild-caught embryos, the noise in the full data set may reflect real differences in gene

expression due to factors outside of our control, such as lake environment, immune status, and parentage.

We then restricted our PCA analysis to 100 developmental patterning genes involved in core processes of vertebrate appendage development (Supplementary Table 25). This list was manually curated based on extensive analysis of the fin and limb development literature. Criteria for inclusion in this list are genes and gene families that (1) are known to affect fin and limb patterning, and/or (2) have been implicated in the fin-to-limb transition. These include genes that have roles in the proximal-distal patterning, anterior-posterior patterning, appendage bud initiation, outgrowth, and the apical ectodermal ridge to apical ectodermal fold transition in fins and limbs. Analysis of this subset revealed clear groupings by developmental stage (Supplementary Fig. 18b). PC1 accounted for 71.2% of the variance and separated stage 26 samples from younger timepoints. Younger timepoints themselves were mostly separated from one another by PC2, which accounted for 13.7% of variation. Grouping based on sequencing approach was not detected in PC1 or PC2.

In addition, we performed PCA analyses on sets of 100 randomly selected genes (Supplementary Fig. 17c) and sets of 1,000 randomly selected genes (Supplementary Fig. 18d). These random subsamples reflect the noise seen in the full data set (Supplementary Fig. 18a) as they fail to group samples by sequencing approach or developmental stage.

Based on these results, we conclude that insights from the fin bud transcriptome data set is most valid when restricted to developmental patterning genes. Batch effects due to sequencing approach may exist for individual genes, but this effect is overshadowed by the general noise in the data set.

11.3 cDNA cloning. Nested PCR was used to amplify cDNAs of target genes. *fgf8* and *sp8* were amplified from a cDNA library pooled from whole-embryo stage 23 and stage 25 libraries. Primers and target sizes are listed below (Ac: *Amia calva*; Lo: *Lepisosteus oculatus*).

AcFgf8_F1: 5'-ACCATTCAGTCCTCGCCTAA-3'
AcFgf8_R1: 5'-GGGCAGACGCTTCATAAAAT-3'

➔ AcFgf8_F1 + AcFgf8_R1 = 465 bp product (for ISH)

LoFgf8_F1: 5'-ACCATTCAGTCCTCGCCTAA-3'
LoFgf8_R1: 5'-TTGTTCAAAAGGTGAAATTCCT-3'
LoFgf8_R2: 5'-GGCAATTCTCATGGACGTTT-3'

➔ LoFgf8_F1 + LoFgf8_R1 = 414 bp product
➔ LoFgf8_F1 + LoFgf8_R2 = 350 bp product (for ISH)

AcSp8_F1: 5'-GGAAGAGCCGAGGTTAGGAT-3'
AcSp8_R1: 5'-GCTGAGGAGGTGTGGAGAAG-3'
AcSp8_R2: 5'-GAGTGACCCAAACCGGAGTA-3'

➔ AcSp8_F1 + AcSp8_R1 = 697 bp product
➔ AcSp8_F1 + AcSp8_R2 = 660 bp product in Bowfin, 330 bp product in Gar (for ISH)

11.4 Genomic comparison of the *fgf8* gene region. The *fgf8* gene regions of bowfin (AmiCal1; Aca scaf19), zebrafish (GRCz11; *fgf8a* chr13, *fgf8b* chr1), stickleback (BROADS1; *fgf8a* groupVI, *fgf8b* group IX), and mouse (GRCm38; chr19) were aligned against the spotted gar *fgf8* region (LepOcu1; LG5), spanning from *btrc* upstream and *poll* downstream of *fgf8*. Repeat-masked sequences and gene annotations were downloaded from Ensembl [Release 100] and aligned with mVISTA[110] using Shuffle-Lagan[111] (calculation window: 100bp, minimum conservation width: 100bp; conservation identity: 70%). Conserved non-coding regions in the VISTA plot (Extended Data Figure 5e) were compared against known *fgf8* region gene regulatory elements previously identified in zebrafish and mouse[117-120]. Genomic

locations of these elements in bowfin and gar along with information on overlap with open chromatin regions in bowfin embryo stages are given in Supplementary Table 26.

11.5 Fgf signaling during bowfin pectoral fin development. Like the absence of *fgf8* expression during holostean pectoral fin development (main Fig. 6e), we also do not see expression for the other classic AER Fgf signaling genes *fgf4*, *fgf9*, *fgf17*[121-123] or most other Fgf genes. In contrast, there is substantial expression of ligand genes *fgf7* and *fgf24* as well as the Fgf receptor gene *fgfr1* (but no other receptor genes), suggesting that Fgf signaling in the holostean pectoral fin relies on these ligand-receptor interactions. Fgf7 is known to play a role in the development of the tetrapod limb[124] and skate pectoral fins[125], while knock-out of *fgf24* leads to the loss of pectoral fins in zebrafish[126]. Interestingly, holosteans genomes kept *fgf24*, which is an 'ohnolog gone missing' in tetrapods[127], as well as *fgf9*, which is an 'ohnolog gone missing' in teleosts[128]. Hence, while retaining a more complete ancestral repertoire of Fgf genes compared to the derived teleosts and tetrapods, holosteans evolved a unique way of appendage regulation within bony vertebrates by downregulation of *fgf8*, *fgf9*, and other classic fin/limb Fgf signaling molecules.

Supplementary Figures

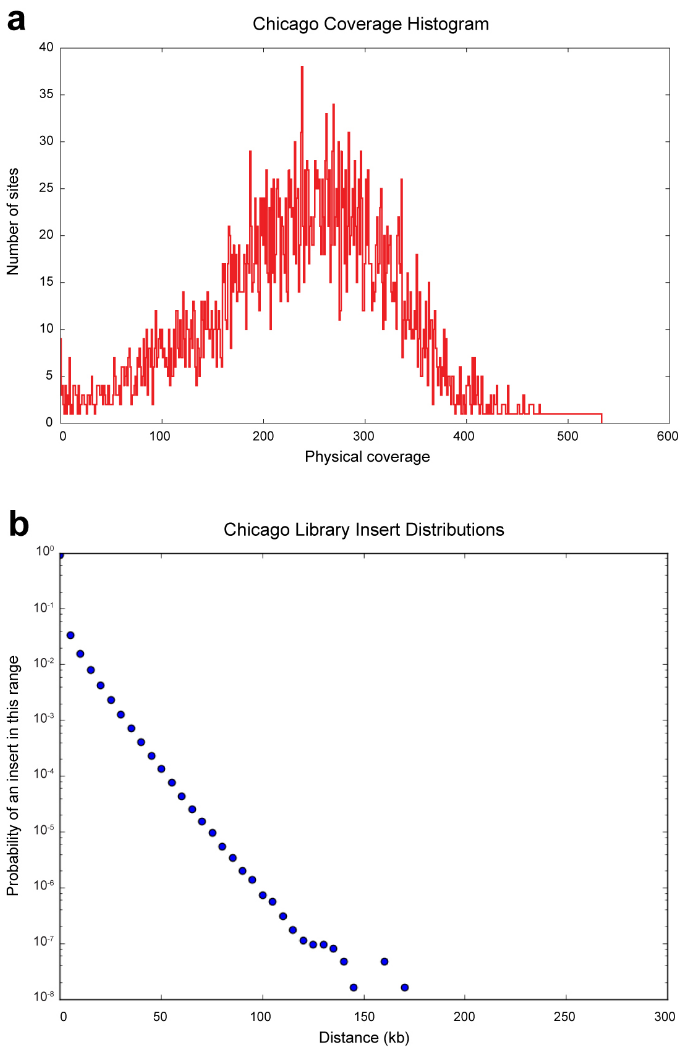

Supplementary Fig. 1. Chicago library. (**a**) Histogram of Chicago coverage over 5,000 randomly sampled sites. Coverage values are calculated as the number of Chicago read pairs with inserts between 1 and 100 kb spanning the sampled site. (**b**) Distribution of insert sizes between forward and reverse reads in the Chicago library.

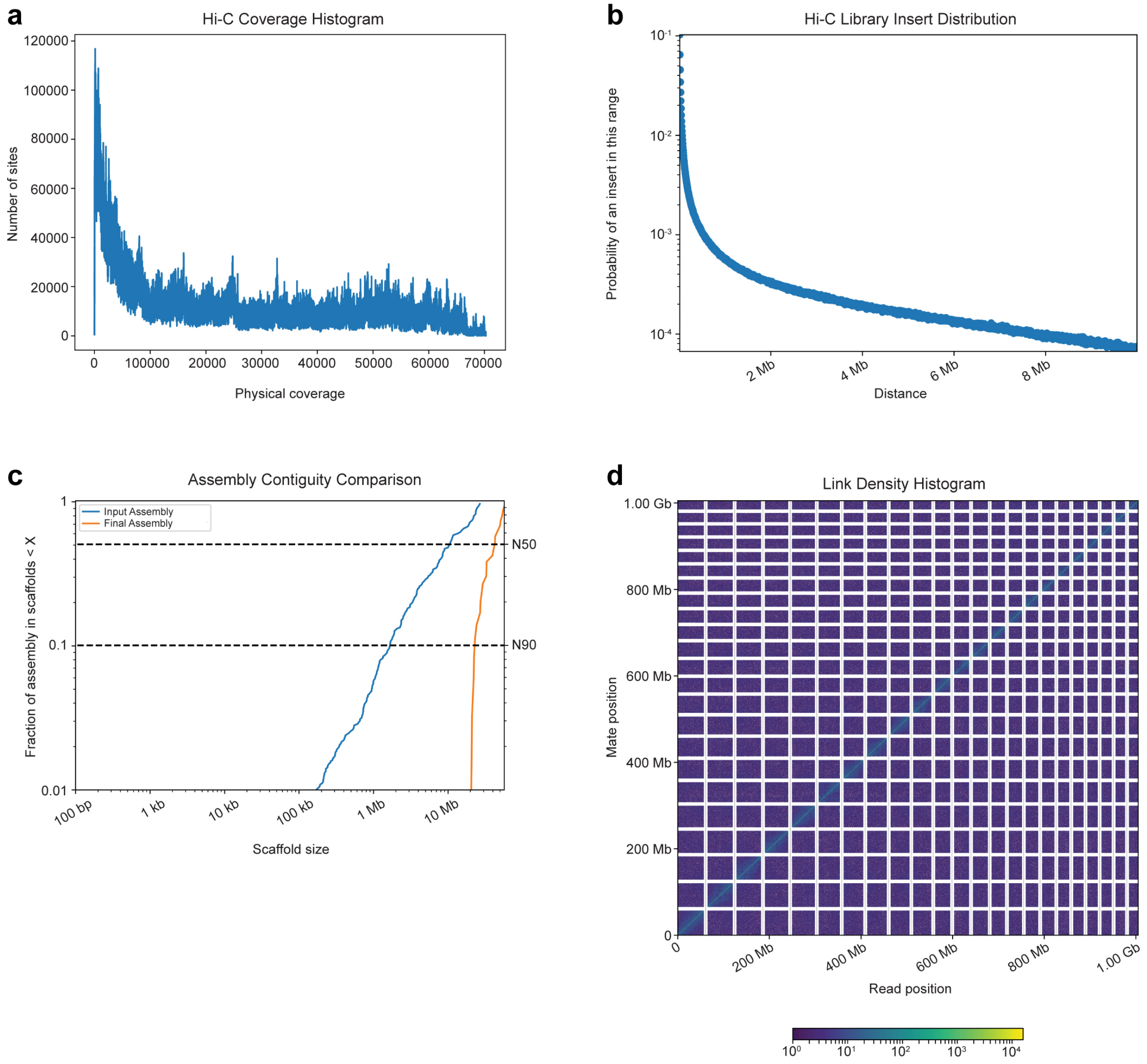

Supplementary Fig. 2. Hi-C scaffolding of the bowfin genome assembly. (**a**) Histogram of Hi-C physical coverage over input assembly. Coverage values were calculated as the number of read pairs with inserts between 10 and 10,000 kb spanning each position in the input assembly. (**b**) Distribution of insert sizes between forward and reverse reads in the Hi-C library. (**c**) Comparison of the contiguity of the input assembly and the final Hi-C-based HiRise scaffolds. Each curve shows the fraction of the total length of the assembly present in scaffolds of a given length or smaller. Dashed lines mark the N50 and N90 lengths of each assembly. Scaffolds less than 1 kb are excluded. (**d**) Hi-C link density histogram. The x and y axes give the mapping positions of the first and second read in the Hi-C read pair respectively, grouped into bins. The color of each square gives the number of read pairs within that bin. White vertical and black horizontal lines have been added to show the borders between 23 scaffolds that represent the bowfin pseudochromosomes. Scaffolds less than 1 Mb are excluded.

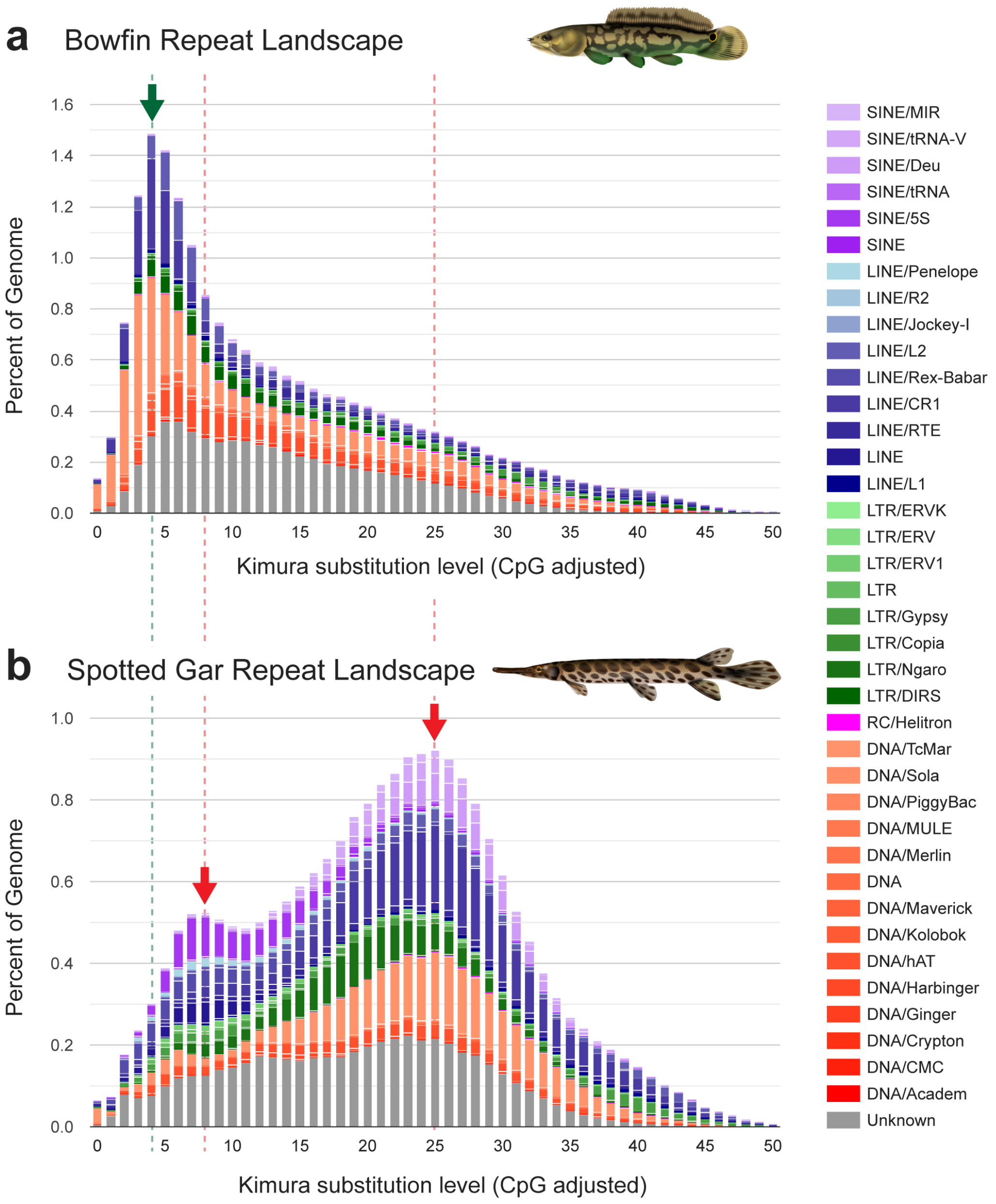

Supplementary Fig. 3. Repeat landscapes of holostean genomes. The age profiles show a single peak at Kimura distance 4 in bowfin (**a**) and two different peaks at Kimura distance 8 and 25 in spotted gar (**b**).

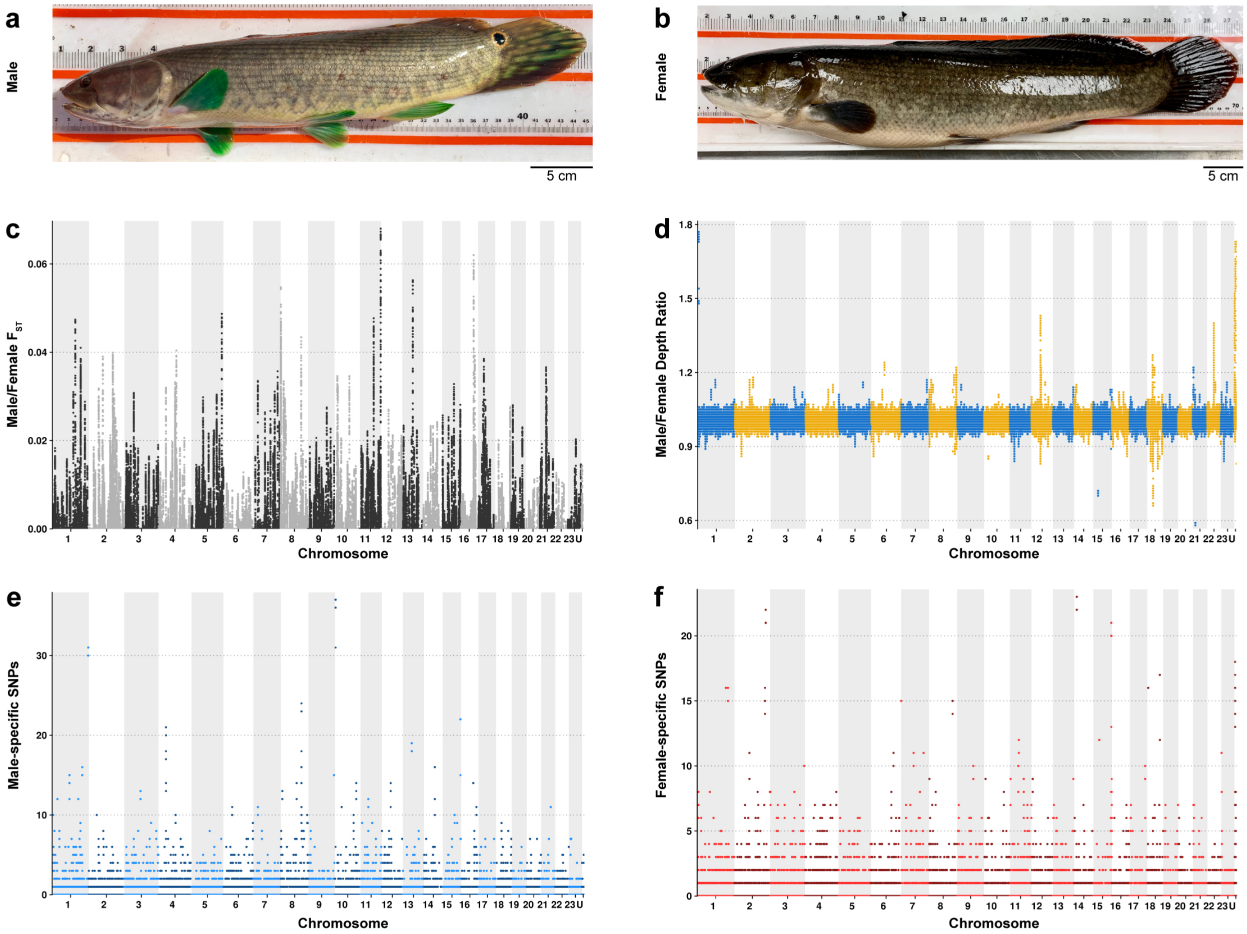

Supplementary Fig. 4. Bowfin sex determination analysis. (**a**) Representative adult, mature male from the Louisiana population in breeding condition with conspicuous caudal ocellus and green fins. (**b**) Representative adult, mature female from the same population. (**c-f**) Pool-sequencing of 30 male vs. 30 female bowfins showing (**c**) Male/Female F_{ST}; (**d**) Male/Female sequencing depth ratio; Manhattan plots showing male-specific SNPs (**e**) and female-specific SNPs (**f**) along bowfin chromosomes. Taken together, these results do not point to any clear sex-specific region in bowfin.

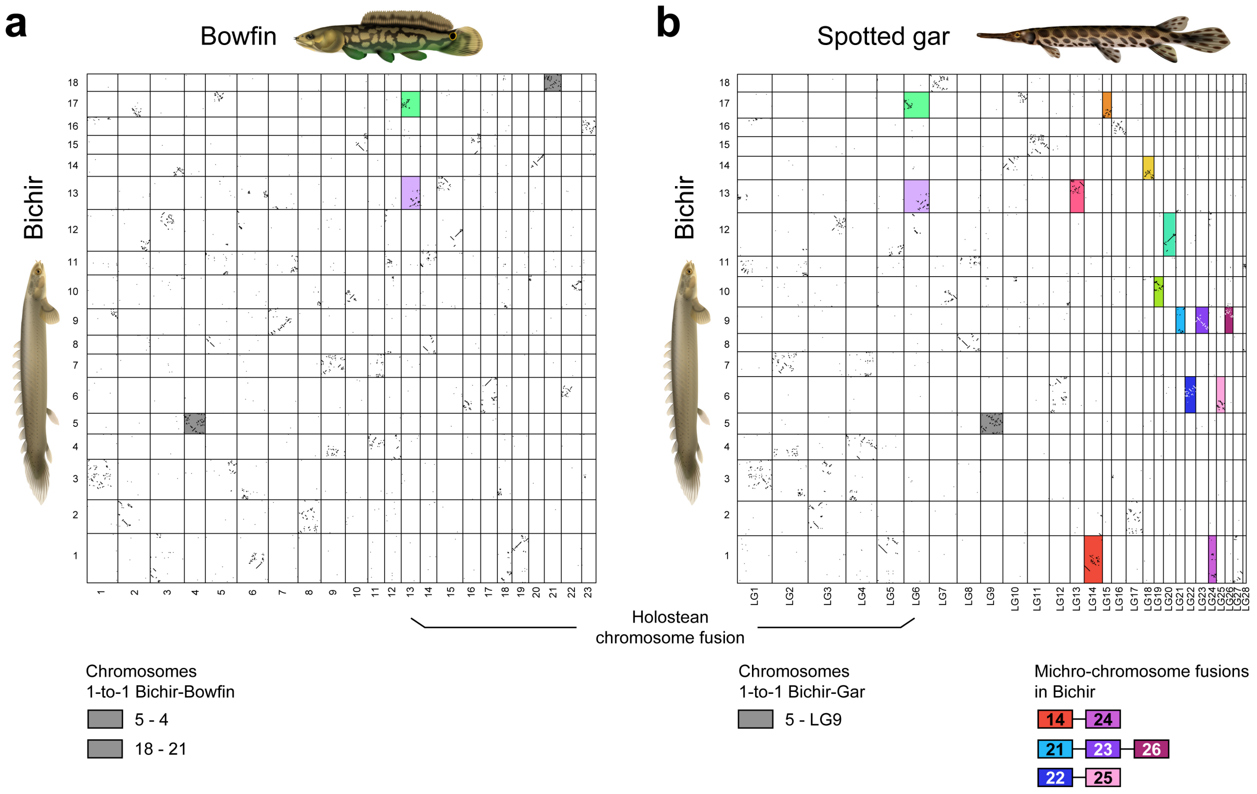

Supplementary Fig. 5: Holostean genomes compared to the bichir genome. Oxford grid comparisons of bowfin (**a**) and spotted gar (**b**) vs. the basally diverging ray-finned fish bichir (*Polypterus senegalus*; NCBI accession ASM1683550v1[35]). Orthologies were established through reciprocal best blast hits. The bichir genome contains no micro-chromosomes, and all assembled chromosomes are >99 Mb in length[35]. Both bowfin and bichir represent karyotypes that are substantially derived compared to the ancestral bony vertebrate genome. Only two chromosomes between bichir and bowfin (**a**) and one chromosome between bichir and gar (**b**) show 1-to-1 conserved synteny. Three fusions of ancestral micro-chromosomes are observed in bichir (**b**), which are different from those in bowfin (Fig. 1c) and in teleosts (Fig. 1d). Orthologs of bowfin 13 (**a**) and gar LG6 (**b**) are on separate chromosomes in bichir, teleosts, and chicken (Extended Data Fig. 2), thus representing a holostean-specific chromosome fusion. Chromosomal color code is the same as in Extended Data Fig. 2. See also https://github.com/DyogenIBENS/BowfinGOPhylogeny.

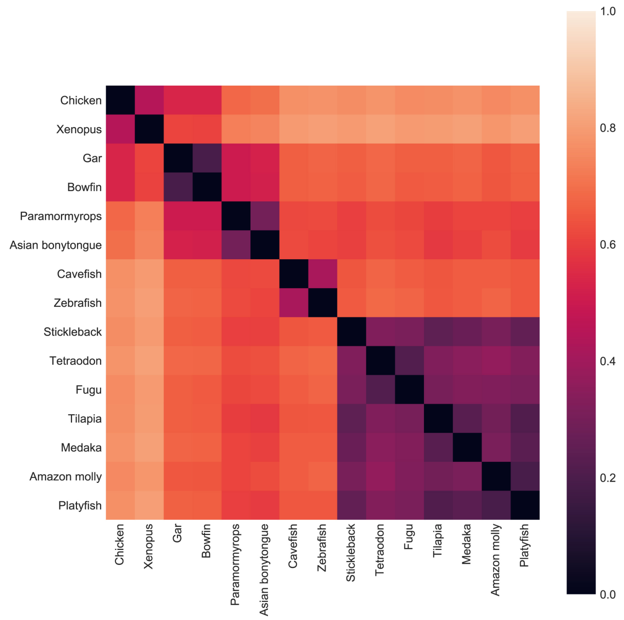

Supplementary Fig. 6. Gene order rearrangement distance matrix. Gene order rearrangement distances were used to generate the Neighbour joining tree shown in main Fig. 1e. See also https://github.com/DyogenIBENS/BowfinGOPhylogeny.

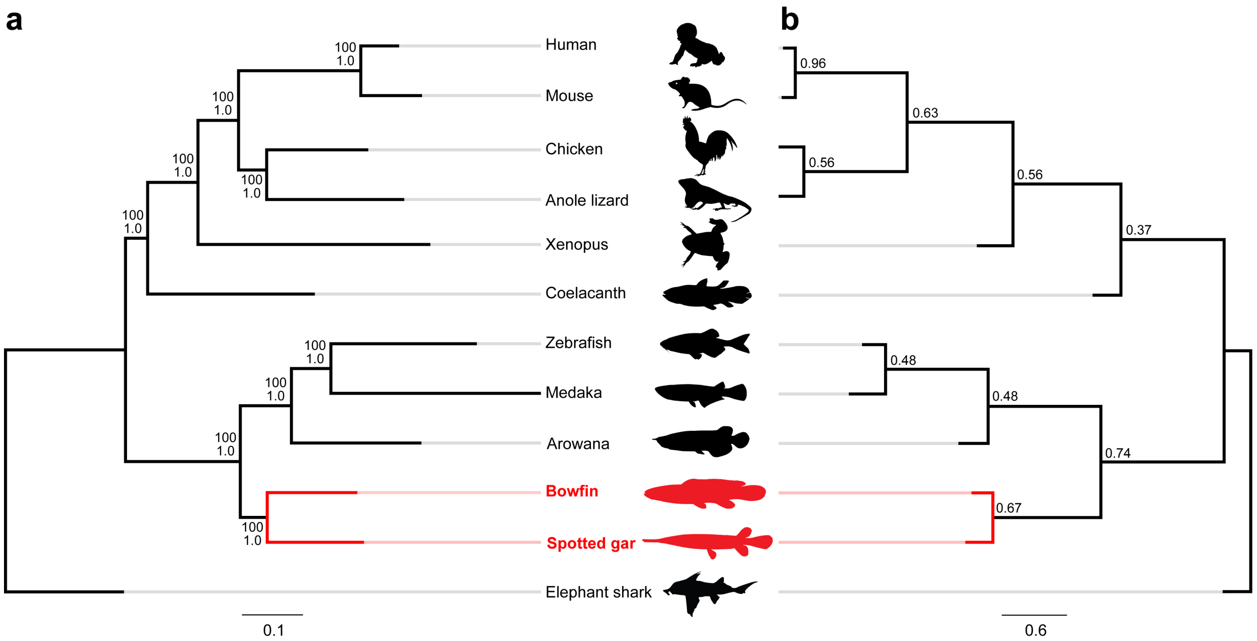

Supplementary Fig. 7. Phylogenetic analyses support holostean monophyly. (**a**) Maximum Likelihood (ML) phylogeny of 1,518,339 amino acid positions from 2,079 1:1 orthologs. Node support is indicated as % ML bootstrap support values (top) and Bayesian posterior probabilities (bottom). (**b**) OrthoFinder species tree. Node support values are the proportion of individual estimates of the species tree that contain that bipartition. Branch lengths are the average lengths for each bipartition in the individual estimates of the species tree. Both trees strongly support bowfin and spotted gar as sister taxa and thus holostean monophyly (red). Species silhouettes are from PhyloPic.org.

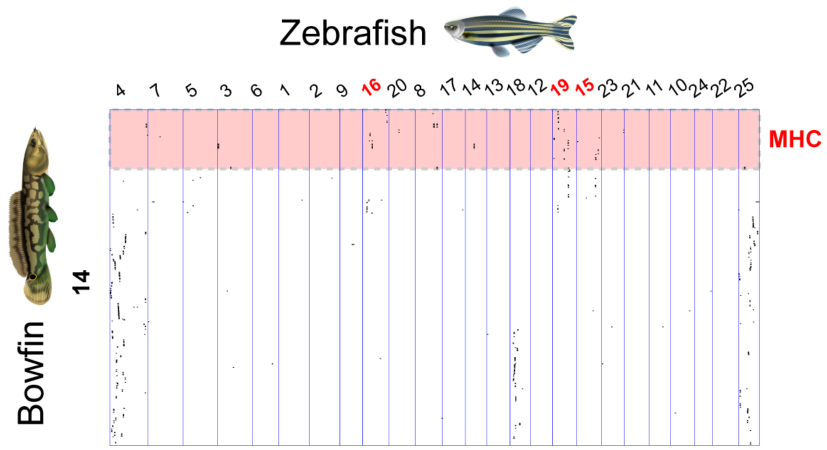

Supplementary Fig. 8. Synteny comparison of bowfin vs. zebrafish MHC. Oxford grid comparison of bowfin pseudochromosome 14 against the zebrafish genome shows that the MHC cluster region (red box, spanning from *rgl2* to *mhc1lca*; see also Supplementary Table 9) is distributed over chromosomes 16, 19, and 15 in zebrafish. Individual genes are indicated as black dots.

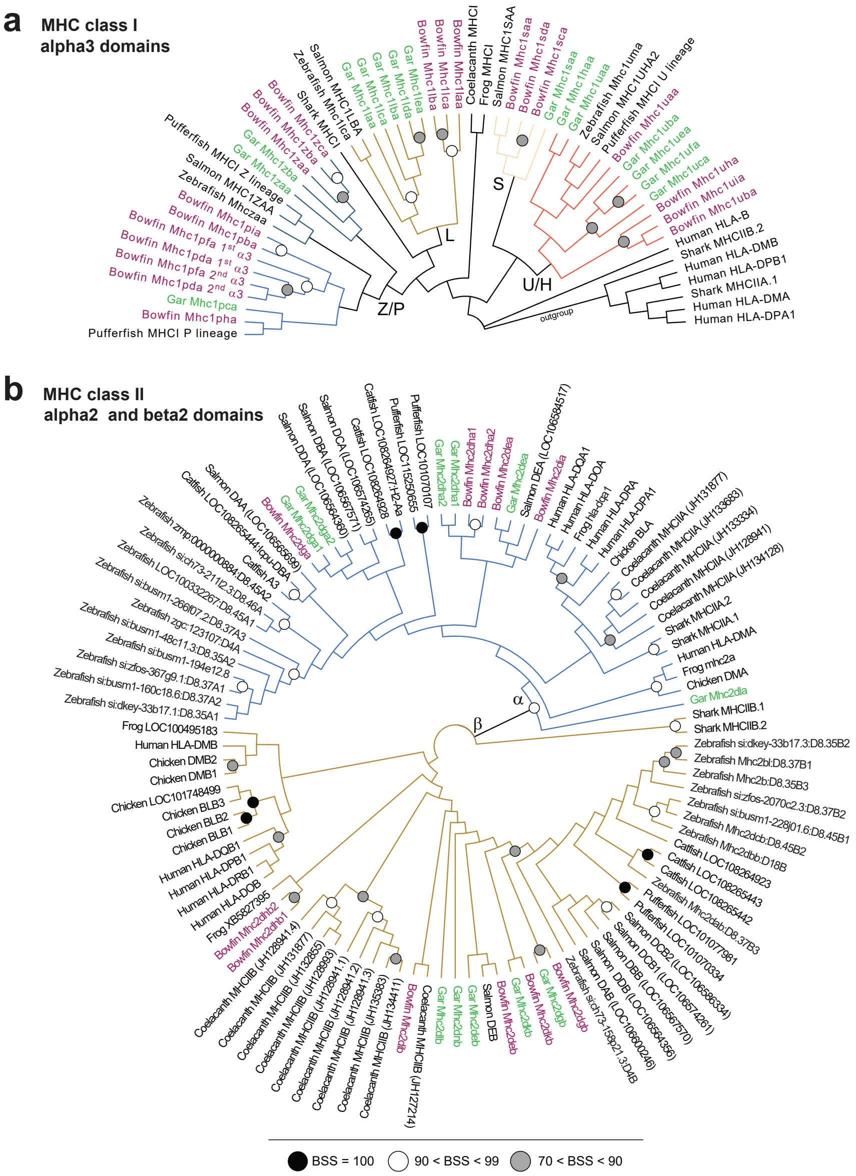

Supplementary Fig. 9. Cladograms depicting the evolutionary relationships among bowfin and representative MHC Class I and Class II sequences from other vertebrates. (**a**) Bowfin MHC class I alpha 3 domains were compared to those of other species. Class I lineages are color coded: U/H = red, S = yellow, Z = navy blue, P = blue, L = gold. Select class II sequences are included as an outgroup. Note that of all H lineages evaluated, only the spotted gar possesses an alpha3 domain and could be included in this analysis. A bowfin *mhc1pda* transcript encodes two alpha 3 domains which are indicated as Mhc1pda.1 and Mhc1pda.2. Two bowfin Mhc1pfa alpha 3 domains (Aca scaf12:9,769,801-9,769,529 and Aca scaf12:9,764,651-9,764,379) are also included although they may represent a pseudogene. (**b**) Bowfin MHC class II alpha 2 and beta 2 domains were compared to those of other species. Class IIa and IIb sequences are indicated by blue and gold branches, respectively. The holostean DG lineage may be orthologous to teleost DA/DB lineages which include salmon DA/DB/DC/DE/DF genes. Bowfin and gar sequences are indicated with purple and green text, respectively. Sequence and species identifiers are listed in Supplementary Tables 9-11. BSS = % bootstrap support. Best-fit substitution model: (**a**) WAG+G and (**b**) JTT+G.

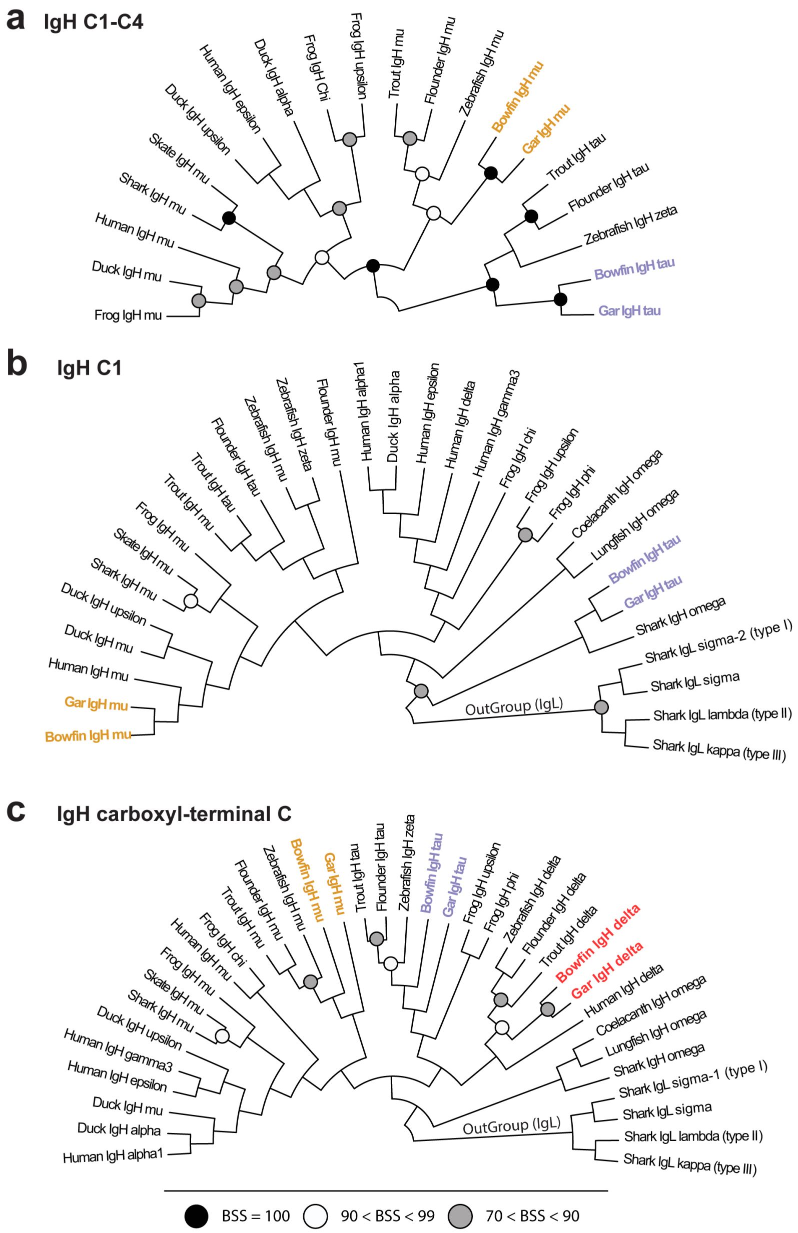

Supplementary Fig. 10. Cladograms depicting the evolutionary relationships among bowfin and diverse IgH sequences. (**a**) IgH sequences that include four constant Ig domains (C1-C2-C3-C4) were compared. IgH sequences not consisting of four constant Ig domains, such as IgHδ (delta), were excluded from this analysis due to complications associated with domain duplications. (**b**) The sequences of the first constant domain (C1) of IgH which forms disulfide bonds with IgL chain were compared. Since the IgD heavy chain of teleosts (and most likely of holosteans) utilize the first domain of IgHμ (mu) for the first domain of IgHδ (delta) peptides, the IgD sequences from teleosts and holostei were not included in this analysis. (**c**) The carboxyl-terminal constant domain of IgH sequences were compared. Sequence and species identifiers are listed in Supplementary Table 12. Holostean IgHμ (mu), IgHτ (tau) and IgHδ (delta) sequences are in yellow, purple, and red text, respectively. Note that shark IgHμ (mu) and IgL (outgroup) sequences are from horn shark, whereas the shark Igω (omega) sequence is from sandbar shark. BSS = % bootstrap support (200 replicates). Best fit substitution models: WAG+G+I model for (**a**) and WAG+G model for (**b**,**c**).

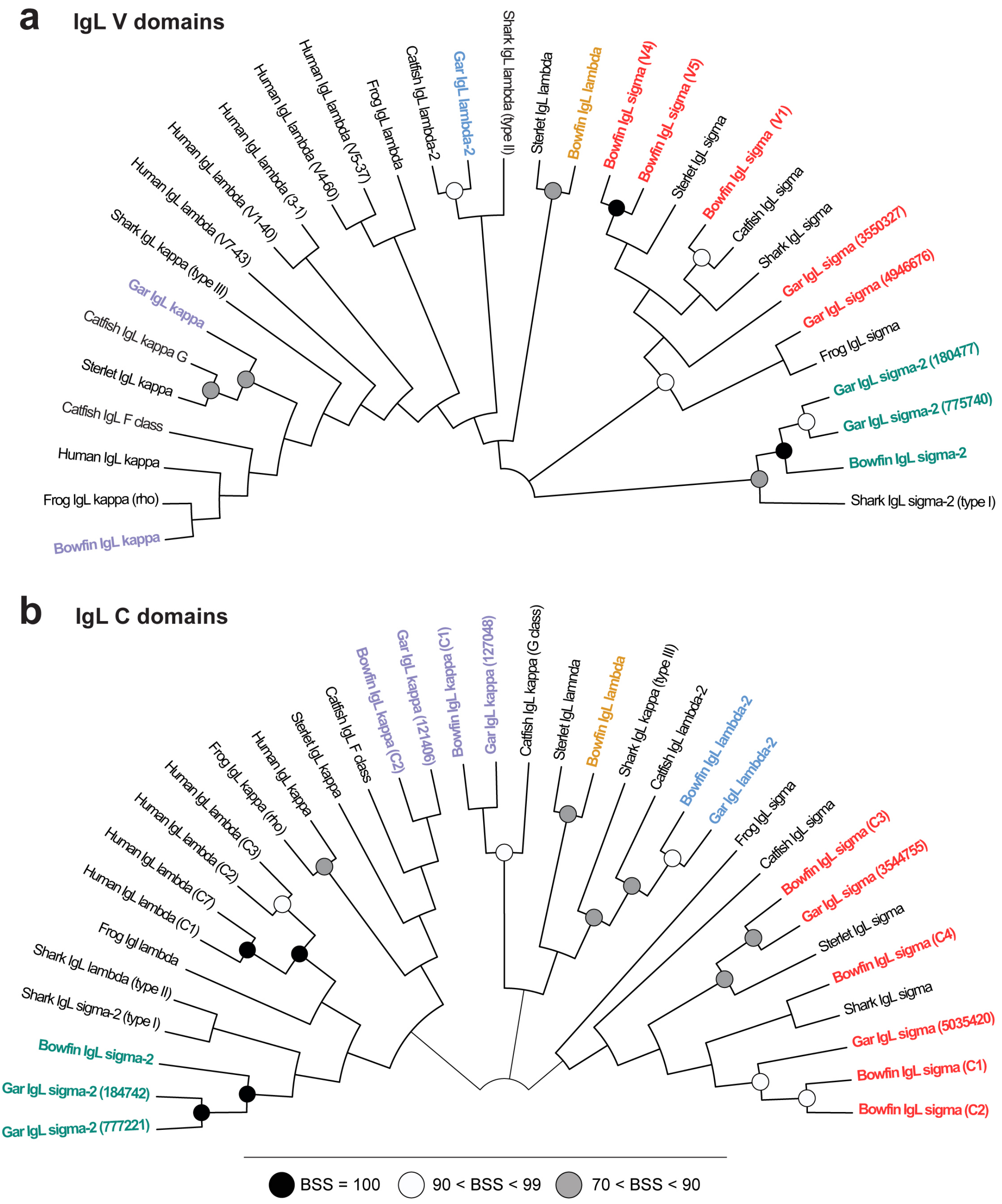

Supplementary Fig. 11. Cladograms depicting the evolutionary relationships among bowfin and diverse IgL sequences. (**a**) IgL variable (V) domains from a range of species. (**b**) IgL constant (C) domains. Sequence and species identifiers are listed in Supplementary Table 13. Following Guselnikov et al. (2018)[58], bowfin and gar sequences representing the five light chain isotypes are indicated by: kappa = purple, lambda = orange, lambda-2 = blue, sigma = red, sigma-2 = green. Note that bowfin IgL lambda and lambda-2 sequences likely reflect pseudogenes. BSS = % bootstrap support (200 replicates). Best fit substitution model: WAG+G+I.

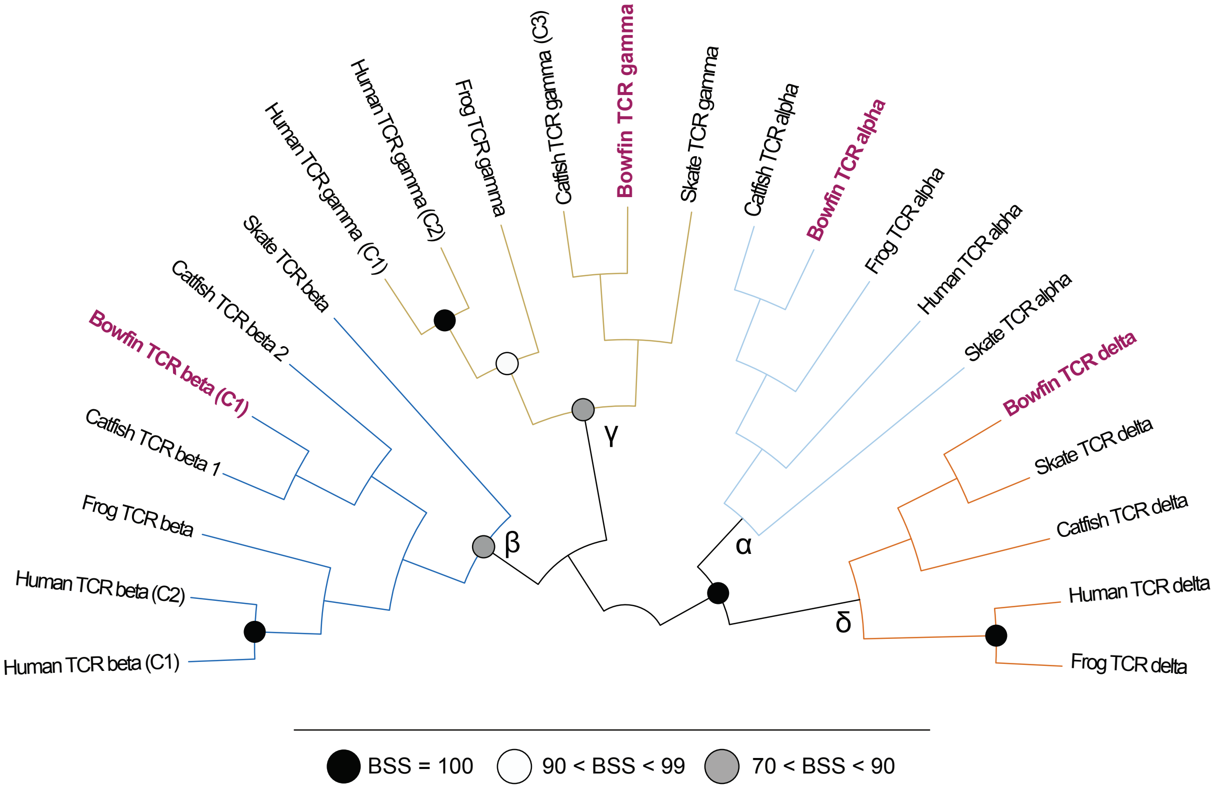

Supplementary Fig. 12. Cladogram depicting the evolutionary relationships among bowfin and diverse T cell receptors (TCRs). TCR constant domains were compared to sequences from other vertebrate lineages. Sequence and species identifiers are listed in Supplementary Table 14. TCRα (alpha), TCRβ (beta), TCRγ (gamma) and TCRδ (delta) are indicated with light blue, dark blue, yellow, and orange branches, respectively. Bowfin sequences are indicated with purple text. BSS = % bootstrap support (200 replicates). Best fit substitution model: LG+G+I.

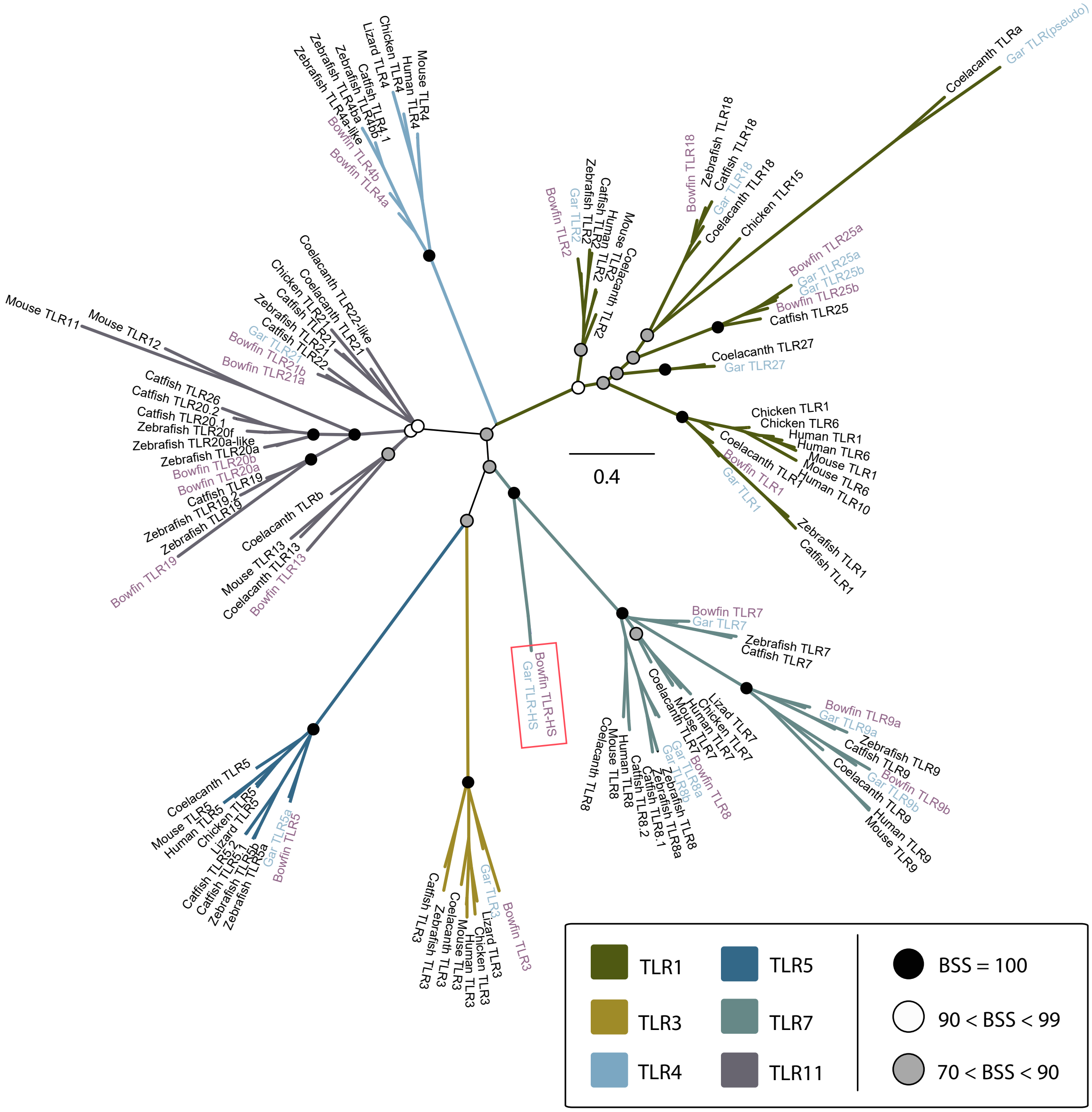

Supplementary Fig. 13. **Evolutionary relationships among vertebrate TLRs.** TIR domains identified in bowfin TLR sequences were compared to TLR sequences from a range of vertebrate species. Bowfin TLRs were annotated based on these relationships. Sequence and species identifiers are listed in Supplementary Table 15. Bowfin and spotted gar have a holostean-specific TLR-HS (red box). Coelacanth sequences were reported previously and include possible pseudogenes ENSLACG00000006376 (TLRa) and ENSLACG00000004773 (TLRb)[129]. Branches are color coded based on the six major families of TLRs. BSS = % bootstrap support.

a Scpp5 protein alignment

```
                 exon2              exon3                  exon4
Gasterosteus     MKLVIFCLCLASTACAAP S--IFHYLPHYAGSRQQVPPSQ VGNPFTAG-QSLPPPGAAGAYSVEL
Danio            MWTSLLCLLLAGAVSAAP LSPFFNYLPHYGSPR------Q GNTGGFQGMPSQPHPAMNAPISMEI
Astyanax         MWSSILCLSFVTMVSSAP ISPFFNYLPHYGNPRP-GPSAQ GTNDFFS---TNGQPAFNAPISMEI
Ictalurus        MWSAVFCFSIISAVSAAP LYSF---LQHYGNPMQSGPSNQ AANDMFS--PLHPHTGMTTPISMEI
Electrophorus    MWSSILCLSFVTAVSAAP MPPFLNYLPHYGNPIPSGPSTQ GNNDFFPPVPTHLQPNINSPISMEI

                 exon5
Gasterosteus     IYPHRVAGGVGGTNAG-Q ----------------------- ------------------
Danio            IFPPRFPANPAGGAAGTS ----------------------- ------------------
Astyanax         VFPPQFQGSPVGGAGAGP ----------------------- ------------------
Ictalurus        LLPPRFPGSAGGQGSG-P GNSMFPGLPSHLQPGVNTPISIE LFHPGFQGTAAGGQGSRT
Electrophorus    VFPPRYPGSPVGAGSG-P ----------------------- ------------------

                 exon6                   exon7       exon8            exon9
Gasterosteus     SF---GFIKYSIPQPPGRQSVEV YYPYDFSQQR- IMTNLPPMTNSPQMPN VFPFEYPPQNIPQQ-IPN
Danio            SFPTQAFIKYSLPKAPGRKSVEI FYPYDFGRA-Q DQP---NVPLIPQLPN IFPFDLMPQTVPQQ-PPV
Astyanax         AFPSQGFIKYSLPKVPGRKSVEI YYPYDFTQQRQ MMPSVPMMPAVPQLPG LVTFDNPPHNVPQQQPPR
Ictalurus        -----GLIKYSIPKAPGRKSIEI YYPYNFAQG-E VLP-----NILPQIPS IFPFNYLPQTPPQQQPPR
Electrophorus    VFPSQAFIKYSLPKAPGRKSVEI YYPYDFSRG-Q ---------------- ILPIDYAPQTVPQQQQPR

                 exon10                                                      exon11
Gasterosteus     IPSFNPNALPSQDP-------MQPLQQDQPIQTSQ ----------------------- MPAKV
Danio            NPPFQDGAPQTQEPQQQTQPEQQQQQQQQQAQTGQ ----------------------- VSTRP
Astyanax         -----ASPPQSNDP-------HPQIMHDQPVQTGQ ----------------------- TPINL
Ictalurus        ----QAAPSQGNDQPFVF---SYPPQSGPQQQTPR AAP-QANDPQQQIQQDPQVPAGQ P----
Electrophorus    ----------------------------------- ATPPQFSDVQQQPQQDQQMQAGQ P----
```

b Scpp1 protein alignment

```
                 exon2              exon3            exon4
Gasterosteus     MMVNIIMFCLMGAVFSNP ISFNAVLESNELDSNS TEN--LSSQSSENGTSAL--L
Danio            MKSALLILCLLGAACANP ILHKVAMEMIQHASNS SESSSISESSDQSNTSEPSEE
Astyanax         MKLTVVILCLLGATAANP ILHKVSMEIIDHASNS T--SSVSESSEESNTSE--HD
Ictalurus        MKLAFVILCLLGAAGANP ILHTDMMET---ASNS SQTSSMSASTEE--TVAIDQD
Electrophorus    MKFAIVILCLLGAASANP ILQTEFIDVMEHASNS SSSISESSKQSD----TSEQD

                 exon5      exon6                    exon7
Gasterosteus     SSEESTSK-Q SQSSESSSESESESTSTESTSEDR KQTEEESNSLLDEKDV
Danio            KSEENVSDSN SSES------------LESESDEP ISKESESHSV---ESL
Astyanax         SSPENTSENI SSES------------DESKSDEQ TSDLSHSHSL---EER
Ictalurus        SSQENTSEDT TSES------------MESNSSEK TLETSQSNSL---EER
Electrophorus    ---------- ------------------------ ITESLKSSS----VEQ

                 exon8                  exon9
Gasterosteus     ADPM--AETTDNSMGSEENIRK SEGAAVSTNDSSESTS---------------------
Danio            IGKSETALTADNTQSSKENIRR GWIYTLKWVQP---NNNIVQPTGQPHENDIQSTSDAS
Astyanax         FGTGEPGMTTDNSQGSQENMRK NWVHLINVKMASKE--DTEEVTDQPDEEDKDTTEDQ-
Ictalurus        FGNGEAGMTVDNSQGSTEIMRK NWIHVFSPQDISSEDNSTSASLALA------------
Electrophorus    FVADVYGITKDNSD-SEENVRE NLLHSVNITILSADDNSISESTENPDTDDSDAEHENS

                 exon9 (continued)
Gasterosteus     --------------------------------------------------SESNETSETS
Danio            QISD---------------------------------------------SSESSESQEKV
Astyanax         -------------------------------------------------TSESSESTEKP
Ictalurus        -SSEISKSMESPEKNSKS-ISSSSESSESTEGQGNNSTSSSSESSESSESSESSESTENP
Electrophorus    ESSESGESNESGKSSESGESSESGESGESDESSESNESGKSSESSESDESGESDESSESG

                 exon9 (continued)
Gasterosteus     ETSDSSD-----------------------------------------------------
Danio            VVN---------------------------------------------------------
Astyanax         TPS---------------------------------------------------------
Ictalurus        EKNSNSSSSESSESSESSESSESQEEKSSSSS----------------------------
Electrophorus    ESNESGKSSESGESSESSESSESGESNESGKSSESGESGKSDESGESSESNESGKSSESG

                 exon9 (continued)
Gasterosteus     ------------------------------------------------------------
Danio            -----------------------------------------------KMQVDNSDDTSES
Astyanax         ------------------------------------------------------SSSSST
Ictalurus        ---------------------------------------ESSESNENQQNASDSSSESKS
Electrophorus    ESGESDESGESSESNESGKSSESGKSDESSESGESGESSESSEDQELDHSSNDTQSDSSS

                 exon9 (continued)
Gasterosteus     -------------------SSDSSTSVEESEASDDALLGQLETKDCVNGT--QSCE--SE
Danio            AEHNGVV--ATEYSNSN--SSSSSSSSESTESKDSGTDSESRSTECVPGDDSQDCE--SE
Astyanax         EDSRAVVVDSSENSHSN---SSSSSSSESHESSHSTESTESQSKECPPGTDSNECD--SD
Ictalurus        VENRST-IDSDENSALKSSNSSSSESHESTETTESTESKQSRSNECQPGADSQDCDSDSD
Electrophorus    MESASS-EESESENDVSDSNGEKSSSSESPEADGIVQSADSHSSECADGD--AECH--SQ

                 exon9 (continued)
Gasterosteus     EYLFQDIGDDAH-YSVDSLMVPDEDEREFSLRR
Danio            ENLPQDIGDDGATDPFNGFLMPDVAEP------
Astyanax         EYQFHDVGDDGATDPFNGFHTTDNAGHEFAFKR
Ictalurus        EYVLQNVGDDGTNDPFDGFHVPDSTEREVTFKR
Electrophorus    DTLEGGVGDDEASEPSNWLDTPVVDL-------
```

Supplementary Fig. 14. Teleost Scpp5 and Scpp1 proteins. Alignment of three-spine stickleback (*Gasterosteus*), zebrafish (*Danio*), Mexican tetra (*Astyanax*), channel catfish (*Ictalurus*), electric eel (*Electrophorus*) Scpp proteins support the presence of (**a**) *scpp5* in the stickleback (groupIX: 8,859,633-8,861,237) and channel catfish genomes (NC_030444.1 14,455,199-14,461,226) and (**b**) of *scpp1* in channel catfish (NC_030418.1: 4,433,039-4,437,245) and electric eel genomes (NC_049545.1: 24,763,716-24,766,384).

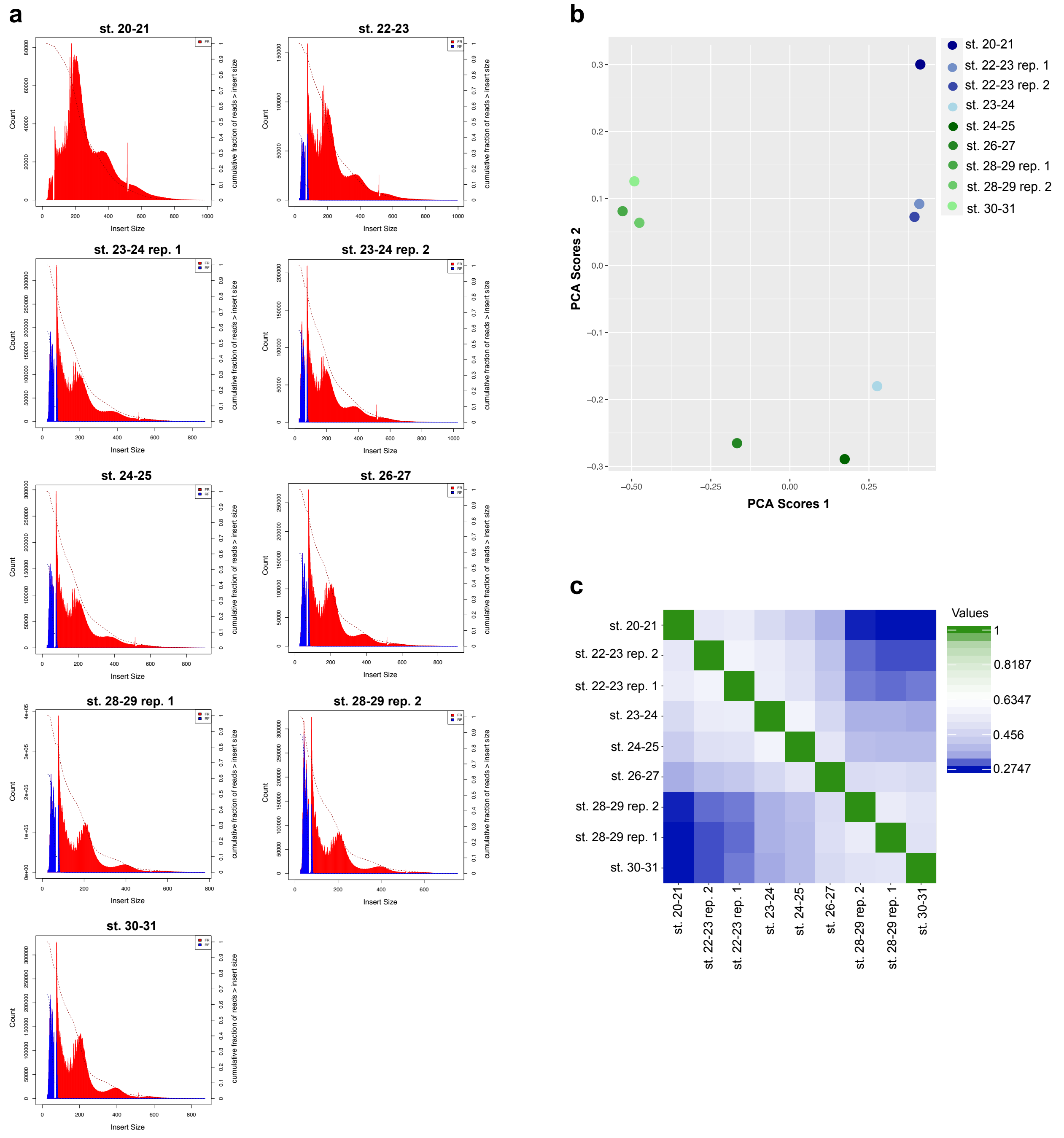

Supplementary Fig. 15. ATAC-Seq sampling through bowfin development. (**a**) Nucleosome periodicity in ATAC-Seq data based on insert sizes of mapped reads. (**b**) PCA plot of pairwise Jaccard distances among OCR profiles of bowfin developmental stages. (**c**) Heatmap of pairwise Jaccard distances among ncOCR profiles of bowfin developmental stages.

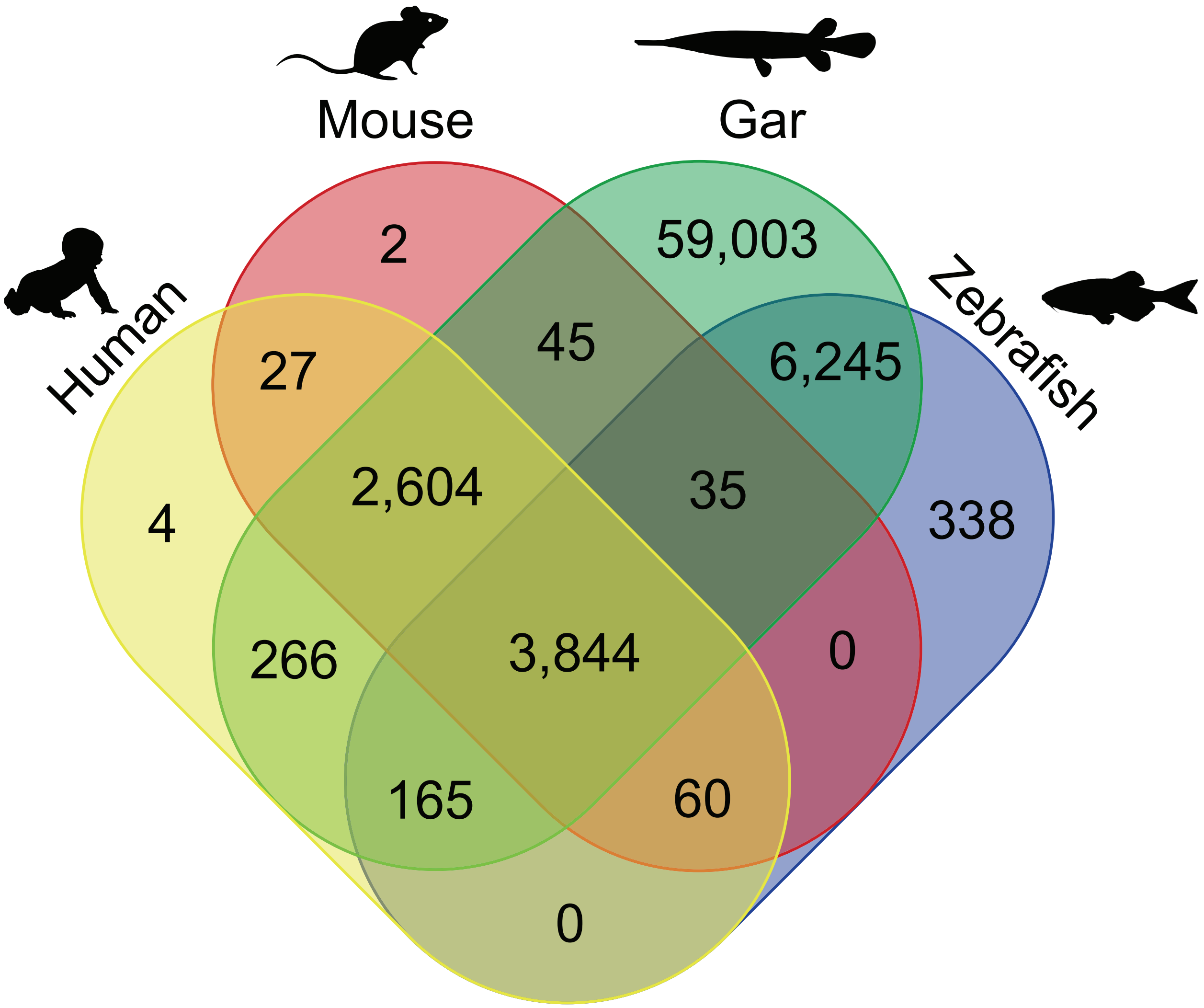

Supplementary Fig. 16. Conservation of bowfin non-coding open chromatin regions. Bowfin ncOCRs in gar, zebrafish, mouse, and human determined from whole genome alignments using Progressive Cactus and halLiftover[86] (see Methods and Supplementary Table 17 for further details). Species silhouettes are from PhyloPic.org.

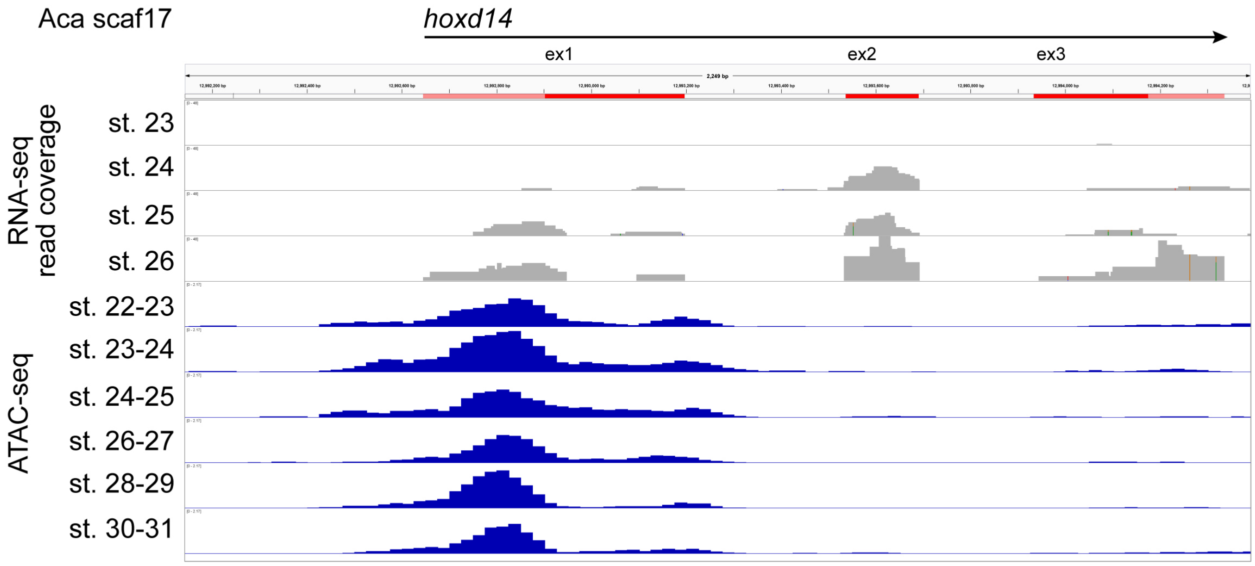

Supplementary Fig. 17. Bowfin *hoxd14* pseudogene. IGV browser view of the *hoxd14* region on pseudochromosome 17 (Aca scaf 17) shows read coverage from fin bud RNA-seq transcriptomes (paired-end samples) and whole embryo ATAC-Seq profiles of different developmental stages[116]. Predicted exons are indicated in red with dark red indicating parts confirmed by fin bud cDNA cloning and sequencing.

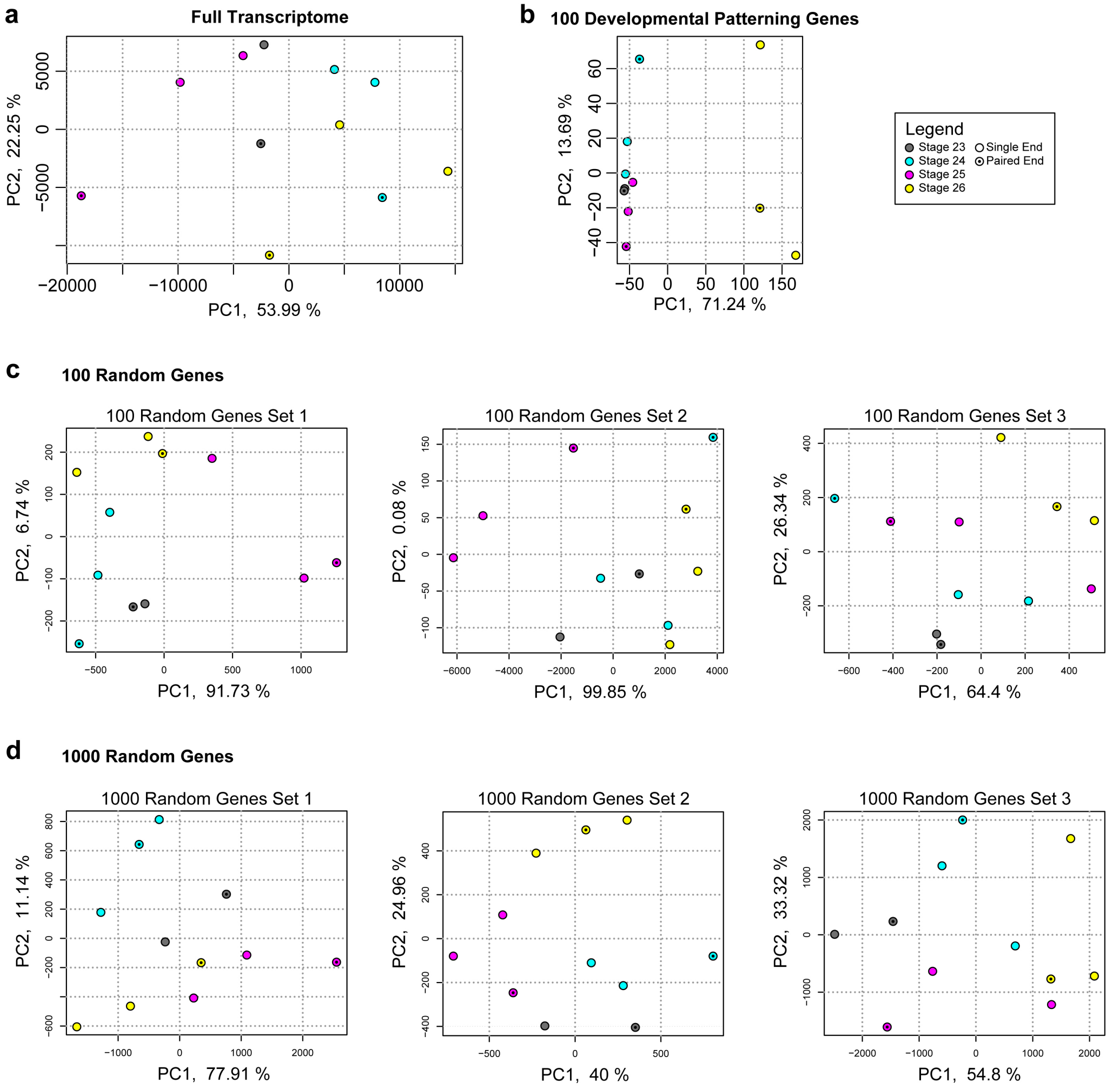

Supplementary Fig. 18. Principle Component Analysis of the fin transcriptome based on the full transcriptome (**a**), 100 developmental patterning genes (**b,** see also Supplementary Table 25), as well as exemplary sets of 100 random genes (**c**) and 1,000 random genes (**d**).

Supplementary Tables

Supplementary Table 1. Assembly statistics for bowfin genome AmiCal1.

	de novo Assembly	Chicago Scaffolding	Chicago + Hi-C Assembly (AmiCal1)
**Sequenced read pairs**	433.5 M	177 M	527 M
**Scaffold N**	64,769	1,846	1,958
**Scaffold N50**	23.5 kb (8,829 scaffolds)	11.7 Mb (22 scaffolds)	41.2 Mb (9 scaffolds)
**Contig N50**	21.1 Kb	21.1 kb	21.1 kb
**Scaffold N90**		1.69 Mb (93 scaffolds)	22.5 Mb (20 scaffolds)
**Assembly Size**	768.2 Mb	775.8 Mb	831.0 Mb
**Longest scaffold**	291.5 kb	27.5 Mb	57.1 Mb
**Chr N**			23 (refs.1,2)
**pseudochromosome N**			23
**% assembly in 23 pseudochr.**			99% (824/831Mb)

References:
1. Ohno et al. (1969) Chromosoma 26:35-40.
2. Majtanova et al. (2017) J Exp Zool B Mol Dev Evol 328:620-628.

Supplementary Table 2. Read mapping statistics. Raw *de novo* genome reads as well as whole embryo and fin transcriptomics RNA-Seq reads were trimmed with Trimmomatic as described in Methods and mapped to the final genome assembly with bowtie2 ("very-sensitive", other parameters set to default).

	Library	Sequencing	Total Reads	Mapped Reads	Unmapped Reads	% Mapped
***de novo* Genome Assembly**	1	Paired	856,560,078	805,312,004	51,248,074	94.02
**Whole Embryo Developmental Transcriptomes**						
Stage 22-23	1	Paired	57,890,682	45,476,494	12,414,188	78.56
Stage 23-24	1	Paired	55,724,154	43,984,090	11,740,064	78.93
Stage 24-25	1	Paired	50,285,408	39,330,342	10,955,066	78.21
Stage 26-27	1	Paired	58,136,568	46,078,733	12,057,835	79.26
Stage 28-29	1	Paired	56,535,344	45,751,948	10,783,396	80.93
Stage 30-31	1	Paired	63,042,972	49,102,203	13,940,769	77.89
**Fin Developmental Transcriptomes**						
Stage 23	1	SE	43,454,251	30,103,826	13,350,425	69.28
Stage 23	2	SE	36,644,157	24,476,766	12,167,391	66.80
Stage 23	3	Paired	140,141,344	75,693,716	64,447,628	54.01
Stage 24	1	SE	52,046,328	34,482,734	17,563,594	66.25
Stage 24	2	SE	49,436,077	32,094,424	17,341,653	64.92
Stage 24	3	Paired	168,309,964	92,386,328	75,923,636	54.89
Stage 25	1	Paired	156,860,268	89,029,014	67,831,254	56.76
Stage 25	2	SE	46,116,597	31,384,683	14,731,914	68.06
Stage 25	3	SE	51,854,039	35,082,258	16,771,781	67.66
Stage 26	1	Paired	179,982,764	100,374,051	79,608,713	55.77
Stage 26	2	SE	47,574,096	31,330,993	16,243,103	65.86
Stage 26	3	SE	46,397,650	27,219,917	19,177,733	58.67

Supplementary Table 3. BUSCO and CEGMA scores for bowfin genome assembly and annotation.

	Genome Assembly (AmiCal1)	MAKER Annotation Transcripts	MAKER Annotation Proteins
**BUSCO Eukaryota odb9** (n: 303)	C:89.1% [S:88.1%, D:1.0%], F:2.0%, M:8.9%	C:91.4% [S:89.1%, D:2.3%], F:6.6%, M:2.0%	C:90.8% [S:88.8%, D:2.0%], F:6.9%, M:2.3%
**BUSCO Metazoa odb9** (n:978)	C:94.0% [S:93.4%, D:0.6%], F:1.1%, M:4.9%	C:93.6% [S:92.5%, D:1.1%], F:4.5%, M:1.9%	C:92.0% [S:91.9%, D:0.1%], F:5.4%, M:2.6%
**BUSCO Vertebrata odb9** (n: 2,586)	C:95.0% [S:94.1%, D:0.9%], F:2.9%, M:2.1%	C:89.1% [S:87.3%, D:1.8%], F:8.5%, M:2.4%	C:88.1% [S:86.6%, D:1.5%], F:8.9%, M:3.0%
**BUSCO CVG** (n:233)	C:97.4% [S:97.0%, D:0.4%], F:1.3%, M:1.3%	C:93.1% [S:91.8%, D:1.3%], F:5.6%, M:1.3%	C:90.1% [S:89.7%, D:0.4%], F:7.3%, M:2.6%
**BUSCO Actinopterygii odb9** (n:4,584)	C:91.9% [S:90.1%, D:1.8%], F:4.3%, M:3.8%	C:83.8% [S:81.1%, D:2.7%], F:9.1%, M:7.1%	C:82.3% [S:79.7%, D:2.6%], F:9.9%, M:7.8%
**CEGMA CEG** (n: 248)	Complete: 98.0%, Complete + Partial: 99.6%	--	--
**CEGMA CVG** (n: 233)	Complete: 96.6%, Complete + Partial: 98.3%	--	--

C: complete, S: singleton, D: duplicated, F: fragmented, M: missing

Supplementary Table 4. Repeat content in bowfin in comparison to spotted gar.

Element name	Bowfin (AmiCal1)			Spotted Gar (LepOcu1)		
	Genome Coverage (bp)	% Genome Coverage	# Copies	Genome Coverage (bp)	% Genome Coverage	# Copies
**all DNA**	**58896956**	**7.087**	**283729**	**40034377**	**4.233**	**182131**
**all LINE**	**32650404**	**3.929**	**122893**	**59115114**	**6.250**	**258483**
**all LTR**	**18887718**	**2.273**	**58259**	**26583911**	**2.811**	**123272**
**all SINE**	**2698499**	**0.325**	**24013**	**29556090**	**3.125**	**171883**
**all Low complexity**	**2183573**	**0.263**	**42965**	**1081106**	**0.114**	**17470**
**all Satellite or simple repeat**	**17206706**	**2.071**	**382157**	**6061576**	**0.641**	**42617**
**all Unknown and misc.**	**51278478**	**6.171**	**255416**	**52850849**	**5.587**	**360694**
**Total**	**183802334**	**22.118**	**1169432**	**215283023**	**22.760**	**1156550**
DNA/Academ	2379	0.000	14	2854	0.000	37
DNA/Blackjack	135799	0.016	615	780	0.000	9
DNA/Crypton	192130	0.023	1663	81043	0.009	719
DNA/Dada	57887	0.007	621	2678	0.000	25
DNA/EnSpm	1501171	0.181	10331	455718	0.048	3722
DNA/Ginger1	42161	0.005	537	7141	0.001	64
DNA/Harbinger	319348	0.038	1939	325325	0.034	3573
DNA/PIF-Harbinger	658249	0.079	2702	272113	0.029	922
DNA/Harbinger (all)	977597	0.118	4641	597438	0.063	4495
DNA/hAT	5599116	0.674	31737	1976269	0.209	11046
DNA/hAT-Ac	3534231	0.425	15119	183725	0.019	664
DNA/hAT (all)	14508164	1.746	71543	8458444	0.894	50019
DNA/hAT-Buster	82903	0.010	143	634172	0.067	2077
DNA/hAT-Charlie	3177061	0.382	16235	3068649	0.324	18248
DNA/hAT-Tip100	1434199	0.173	5601	653446	0.069	2862
DNA/Helitron	634349	0.076	4173	224509	0.024	1101
DNA/IS3EU	69282	0.008	575	69018	0.007	395
DNA/IS4EU	1545	0.000	11	32	0.000	1
DNA/ISL2EU	144576	0.017	1492	32375	0.003	319
DNA/Kolobok	302794	0.036	1539	4431	0.000	33
DNA/Mariner	9254492	1.114	27408	5281056	0.558	15909
DNA/Mariner (all)	32042386	3.856	145570	27815503	2.941	111451
DNA/Maverick	2028821	0.244	3773	40120	0.004	127
DNA/Merlin	43943	0.005	694	75089	0.008	607
DNA/MuDr	200652	0.024	1754	23583	0.002	188
DNA/PiggyBac	324070	0.039	1302	141804	0.015	576
DNA/Polinton	563313	0.068	2836	1055152	0.112	2229
DNA/Sola	649039	0.078	3105	141045	0.015	576
DNA/TcMar (all)	31929994	3.842	145107	26661477	2.819	104790
DNA/TcMar-MER6	7544	0.001	128	1319539	0.140	8686
DNA/TcMar-Pogo	259	0.000	4	224694	0.024	1209
DNA/TcMar-Tc1	31118535	3.745	141278	24498821	2.590	94590
DNA/TcMar-Tigger	378064	0.045	2249	1723991	0.182	7674
DNA/Zator	914	0.000	5	897	0.000	3
DNA/Zisupton	682217	0.082	6322	99632	0.011	635
LINE/CR1	14424539	1.736	53247	27014156	2.856	130318
LINE/Crack	174053	0.021	558	13310	0.001	119
LINE/Daphne	1176	0.000	17	578	0.000	8
LINE/Hero	11926	0.001	32	163465	0.017	434
LINE/Jockey	147	0.000	1	2548	0.000	12
LINE/L1	415886	0.050	2192	328599	0.035	1194
LINE/L2	857499	0.103	2486	869857	0.092	3005
LINE/Nimb	1333	0.000	13	26357	0.003	73
LINE/Penelope	94490	0.011	627	2630510	0.278	9440
LINE/proto	142	0.000	2	0	0.000	0
LINE/R2	6953	0.001	71	11567	0.001	54
LINE/R4	225	0.000	3	4814	0.001	20
LINE/Rex-Babar	67783	0.008	294	7799068	0.825	33197
LINE/Rex1	164118	0.020	628	4488111	0.474	9886
LINE/RTE	824483	0.099	4565	1347219	0.142	4823
LINE/RTE (all)	4186616	0.504	19482	4393992	0.465	19692
LINE/RTE-BovB	15222	0.002	59	811796	0.086	2808
LINE/RTE-X	1163469	0.140	5645	2377062	0.251	12599
LINE/Tx1	1774876	0.214	4980	1024255	0.108	3103
LINE/UnaL2	56414	0.007	91	1197	0.000	7
LINE/Vingi	45250	0.005	203	310591	0.033	1358
LTR/BEL	431888	0.052	1004	1797569	0.190	22144
LTR/Copia	108033	0.013	252	273806	0.029	730
LTR/DIRS	8471791	1.019	25083	259001	0.027	1920
LTR/ERV (all)	1474836	0.177	8822	2760867	0.292	10600
LTR/ERV1	1166885	0.140	6342	2736167	0.289	10261
LTR/Gypsy	7332735	0.882	17056	8364546	0.884	36808
LTR/Gypsy/Gmr1	279	0.000	1	104141	0.011	250
LTR/Ngaro	987717	0.119	5219	13686148	1.447	70156
SINE/5SrRNA	73491	0.009	564	11788688	1.246	131996
SINE/AFC	512	0.000	7	236	0.000	4
SINE/all/rRNA	73728	0.009	567	11856219	1.253	43184
SINE/Deu	152961	0.018	1231	7023144	0.742	42353
SINE/HPA	1197	0.000	24	39924	0.004	478
SINE/MIR	1811610	0.218	15820	3059556	0.323	23491
SINE/rRNA	3202	0.000	34	91430	0.010	834
SINE/tRNA	559995	0.067	5216	1638870	0.173	14532
SINE/V	54117	0.007	624	4809442	0.508	38004

Supplementary Table 5. OrthoFinder results summary.

**Number of genes**	251621
**Number of genes in orthogroups**	234400
**Number of unassigned genes**	17221
**Percentage of genes in orthogroups**	93.2
**Percentage of unassigned genes**	6.8
**Number of orthogroups**	15916
**Number of species-specific orthogroups**	86
**Number of genes in species-specific orthogroups**	579
**Percentage of genes in species-specific orthogroups**	0.2
**Mean orthogroup size**	14.7
**Median orthogroup size**	12
**G50 (assigned genes)**	16
**G50 (all genes)**	15
**O50 (assigned genes)**	3745
**O50 (all genes)**	4306
**Number of orthogroups with all species present**	7532
**Number of single-copy orthogroups**	2079

Supplementary Table 6. OrthoFinder orthogroup statistics for bowfin and 11 other vertebrates.

	Bowfin	Gar	Arowana	Zebrafish	Medaka	Xenopus	Coelacanth	Mouse	Human	Chicken	Anole	Elephant Shark
Number of genes	21948	18341	25402	25902	19699	18442	19569	22585	23070	18346	18595	19722
Number of genes in orthogroups	19047	17887	24632	23758	18002	17620	18840	20670	21799	16518	17431	18196
Number of unassigned genes	2901	454	770	2144	1697	822	729	1915	1271	1828	1164	1526
Percentage of genes in orthogroups	86.8	97.5	97	91.7	91.4	95.5	96.3	91.5	94.5	90	93.7	92.3
Percentage of unassigned genes	13.2	2.5	3	8.3	8.6	4.5	3.7	8.5	5.5	10	6.3	7.7
Number of orthogroups containing species	13309	12637	12713	12751	11372	11356	12296	13237	13345	11485	11745	11962
Percentage of orthogroups containing species	83.6	79.4	79.9	80.1	71.5	71.3	77.3	83.2	83.8	72.2	73.8	75.2
Number of species-specific orthogroups	12	2	3	3	10	8	8	7	4	8	8	13
Number of genes in species-specific orthogroups	38	4	8	8	62	40	66	25	30	76	134	88
Percentage of genes in species-specific orthogroups	0.2	0	0	0	0.3	0.2	0.3	0.1	0.1	0.4	0.7	0.4

Supplementary Table 7. Bowfin sex-biased CDSs and gene annotations.
[see separate .xls file]

Supplementary Table 8. A reference-free k-mer analysis of bowfin sex. Numbers for male and female specific k-mer count are listed for three different filtering conditions to determine a k-mer as sex specific.

Minimal count in heterogametic sex	Maximal count in homogametic sex	Number of male-specific k-mers	Number of female-specific k-mers
25	5	95,928	68,857
15	2	546,314	495,804
15	0	546,314	495,804

Supplementary Table 9. MHC region genes in bowfin, human, and zebrafish.
[see separate .xls file]

Supplementary Table 10. Spotted gar MHC genes.
[see separate .xls file]

Supplementary Table 11. MHC Sequence accession Identifiers.
[see separate .xls file]

Supplementary Table 12. Immunoglobulin heavy chain (IgH) sequence accession identifiers.
[see separate .xls file]

Supplementary Table 13. Immunoglobulin light chain (IgL) sequence accession identifiers.
[see separate .xls file]

Supplementary Table 14. T cell receptor (TCR) sequence accession identifiers.
[see separate .xls file]

Supplementary Table 15. Bowfin Toll-like Receptor (TLRs).
[see separate .xls file]

Supplementary Table 16. Relative SCPP gene expression levels in zebrafish skin at three ages. Fragments Per Kilobase of transcript, per Million mapped reads (FPKM), showing relative abundance of each transcript among all transcripts in the zebrafish skin. FPKM_conf_lo/FPKM_conf_hi represent the lower/upper bound of the 95% confidence intervals of the abundance of each transcript. Relative expression levels of SCPP genes located on chromosomes Dre5 and Dre10 are high (>100 in FPKM) or significant (>10 in FPKM and >0 in FPKM_conf_lo). In contrast, relative expression level of *scpp5* is low (5.6 in the FPKM_conf_lo value only at 5 months of age).

Location		Dre1		Dre10				Dre5			
Gene		scpp5	scpp1	spp1	scpp8	scpp11b	scpp11a	scpp14	scpp13	gsp37	scpp12
5 months	FPKM	7.4	20.9	46.7	8.5	401.4	1340.3	128.0	187.0	16.5	26.9
	FPKM_conf_lo	5.6	18.6	43.6	5.6	381.8	1302.9	119.4	180.2	13.2	23.9
	FPKM_conf_hi	9.2	23.2	49.8	11.5	421.1	1377.8	136.5	193.8	19.7	29.8
24 months	FPKM	0.1	18.9	48.5	6.3	277.2	867.9	104.5	129.5	7.0	16.6
	FPKM_conf_lo	0.0	16.6	45.1	3.7	259.8	836.1	96.3	123.5	4.7	14.1
	FPKM_conf_hi	0.4	21.3	51.8	9.0	294.6	899.8	112.6	135.5	9.2	19.0
42 months	FPKM	0.4	9.4	24.1	14.3	377.4	1255.8	107.1	105.3	12.0	29.9
	FPKM_conf_lo	0.0	7.9	21.9	10.6	359.1	1220.9	99.6	100.3	9.3	26.9
	FPKM_conf_hi	0.8	10.8	26.2	18.0	395.8	1290.7	114.6	110.2	14.7	32.9

Supplementary Table 17. Number of OCRs found for each bowfin developmental stage.

Stage/replicate	#OCRs	Unique OCRs per stage	#ncOCRs	Unique ncOCRs per stage
**20-21**	52971	1406	38834	1321
**22-23 rep. 1 (phylotypic)**	67989	--	51433	--
**22-23 rep. 2 (phylotypic)**	68241	--	52351	--
**22-23 merged reps. (phylotypic)**	80862	4813	63578	4561
**23-24**	82966	3645	63876	3159
**24-25**	81289	2834	63948	2796
**26-27**	90469	3700	71576	3600
**28-29 rep. 1**	114174	--	86650	--
**28-29 rep. 2**	98877	--	76923	--
**28-29 merged reps.**	129837	23219	101314	19256
**30-31**	103336	10607	77912	8365
**all merged across stages**	172276	--	140902 (81.8%)	--
**all merged in 2+ stages**	122055	--	97847 (80.2%)	--
**% all merged in 2+ stages**	70.8%	--	69.4%	--

Supplementary Table 18. Bowfin OCRs in N developmental stages (determined by HOMER).

# Developmental Stage(s)	OCRs	ncOCRs
**1 stage**	50224	43058
**2 stages**	28794	23993
**3 stages**	20259	17477
**4 stages**	14260	12642
**5 stages**	12018	10511
**6 stages**	13496	11598
**7 stages**	33239	21636

Supplementary Table 19. HOMER annotation of OCRs in each developmental stage based on nearest MAKER feature. TSS is transcript start site and TTS is transcript termination site. Log enrichment is based on the expected and observed proportions of peaks in a given feature.

Stage 20-21			
Annotation	Number of peaks	Total size (bp)	Log2 Enrichment
TTS	1437	21851276	0.035
Exon	4037	41758855	0.591
Intron	13365	243154688	-0.224
Intergenic	28612	494594613	-0.15
TSS	5486	23315801	1.874
**Stage 23-23 merged reps. (phylotypic)**			
Annotation	Number of peaks	Total size (bp)	Log2 Enrichment
TTS	2172	21856783	0.021
Exon	6026	41760672	0.559
Intron	20909	243178172	-0.188
Intergenic	45199	494804573	-0.101
TSS	6510	23322407	1.511
**Stage 23-24**			
Annotation	Number of peaks	Total size (bp)	Log2 Enrichment
TTS	2245	21856783	0.031
Exon	7117	41760865	0.762
Intron	20983	243178173	-0.22
Intergenic	45584	494804573	-0.126
TSS	6987	23322407	1.576
**Stage 24-25**			
Annotation	Number of peaks	Total size (bp)	Log2 Enrichment
TTS	2113	21851276	-0.027
Exon	6046	41759939	0.556
Intron	20241	243158426	-0.243
Intergenic	46078	494594613	-0.08
TSS	6754	23316902	1.556
**Stage 26-27**			
Annotation	Number of peaks	Total size (bp)	Log2 Enrichment
TTS	2325	21857884	-0.043
Exon	6994	41762337	0.612
Intron	22989	243183738	-0.213
Intergenic	51082	494805956	-0.086
TSS	7027	23324609	1.459
**Stage 28-29 merged reps.**			
Annotation	Number of peaks	Total size (bp)	Log2 Enrichment
TTS	3668	21857884	0.093
Exon	14083	41762665	1.1
Intron	33593	243183725	-0.187
Intergenic	69686	494805956	-0.159
TSS	8726	23324609	1.25
**Stage 30-31**			
Annotation	Number of peaks	Total size (bp)	Log2 Enrichment
TTS	2793	21857884	0.03
Exon	11392	41762321	1.124
Intron	26298	243183753	-0.211
Intergenic	54994	494805956	-0.172
TSS	7788	23324609	1.415

Supplementary Table 20. Conservation of bowfin OCRs/ncOCRs in other vertebrates. We used halLiftover on a 5-way Progressive Cactus whole genome alignment to determine the presence of bowfin OCR and ncOCRs in four other vertebrate species.

Species	OCRs	OCRs 2+ stages	ncOCRs	ncOCRs 2+ stages
Bowfin	172276	122055	140902	97847
Lifted to Gar	99718	77037	72207	56433
% Lifted to Gar	57.9%	63.1%	51.2%	57.7%
Lifted to Zebrafish	32977	25230	10687	8980
% Lifted to Zebrafish	19.1%	21%	7.6%	9.2%
Lifted to Human	25573	19263	6970	5880
% Lifted to Human	14.8%	15.8%	4.9%	6%
Lifted to Mouse	25124	18921	6617	5594
% Lifted to Mouse	14.6%	15.5%	4.7%	5.7%

Supplementary Table 21. Overlap of bowfin OCRs with gar-centric Conserved Non-coding Elements (CNEs) and bowfin Ultra Conserved Elements (UCEs).

				bowfin OCR/ncOCR overlap							
**Gar-centric CNEs** (Braasch et al. 2016)	Total in gar	Lifted to bowfin	%	OCRs	%	OCRs 2+ stages	%	ncOCRs	%	ncORs 2+ stages	%
**All CNEs**	156087	66182	42.4%	22556	34.1%	19471	29.4%	21127	31.9%	18004	27%
**GCNEs (pan-Gnathostome)**	43765	25704	58.7%	10546	41.0%	9235	35.9%	9979	38.8%	8656	34%
**BCNEs (Bony vertebrate-specific)**	78889	23792	30.2%	7578	31.9%	6576	27.6%	6949	29.2%	5921	25%
**RCNEs (Ray-finned-specific)**	33433	16686	49.9%	4432	26.6%	3669	22.0%	4199	25.2%	3427	21%
**FCNEs ("Fish-specific", lost in tetrapods)**	18497	8384	45.3%	3145	37.5%	2719	32.4%	2935	35.0%	2505	30%

**UCEs** (Faircloth et al. 2013)	Total in bowfin	OCRs	%	OCRs 2+ stages	%	ncOCRs	%	ncORs 2+ stages	%
	364	123	33.8%	111	30.5%	100	27.5%	88	24%

Supplementary Table 22. Number of VISTA enhancers detected in bowfin and zebrafish genomes.

				bowfin OCR/ncOCR overlap							
Species	VISTA enhancers	Lifted to bowfin	%	OCRs	%	OCRs 2+ stages	%	ncOCRs	%	ncORs 2+ stages	%
Human	996	600	60.2%	351	58.5%	319	53.2%	338	56.3%	307	51.2%
		Lifted to zebrafish	%								
		438	44.0%								

				bowfin OCR/ncOCR overlap							
Species	VISTA enhancers	Lifted to bowfin	%	OCRs	%	OCRs 2+ stages	%	ncOCRs	%	ncORs 2+ stages	%
Mouse	634	101	15.9%	78	77.2%	72	71.3%	70	69.3%	63	62.4%
		Lifted to zebrafish	%								
		54	8.5%								

Supplementary Table 23. Location of human VISTA enhancers in bowfin OCRs.
[see separate .xls file]

Supplementary Table 24. Mouse OCRs[89] found in bowfin.

Mouse OCRs		Conservation		bowfin OCR/ncOCR overlap							
**Genetic element type**	**Total in mouse**	**Lifted to bowfin**	**%**	**OCRs**	**%**	**OCRs 2+ stages**	**%**	**ncOCRs**	**%**	**ncORs 2+ stages**	**%**
Exon	20979	7591	36.2%	2369	31.2%	1798	23.7%	248	3.3%	221	2.9%
Intergenic	90217	1435	1.6%	862	60.1%	784	54.6%	836	58.3%	758	52.8%
Intron	135807	8600	6.3%	2581	30.0%	2183	25.4%	1425	16.6%	1280	14.9%
TSS	53668	3671	6.8%	2254	61.4%	2151	58.6%	502	13.7%	473	12.9%
TTS	4516	74	1.6%	25	33.8%	23	31.1%	16	21.6%	13	17.6%
Total OCRs in mouse	305187	21371	7.0%	8091	37.9%	6939	32.5%	3027	14.2%	2745	12.8%
Total ncOCRs in mouse (intergenic + intron)	226024	10035	4.4%	3443	34.3%	2967	29.6%	2261	22.5%	2038	20.3%
**Cell/tissue type**	**Total in mouse**	**Lifted to bowfin**	**%**	**OCRs**	**%**	**OCRs 2+ stages**	**%**	**ncOCRs**	**%**	**ncORs 2+ stages**	**%**
Allantois	793	40	5.0%	12	30.0%	10	25.0%	10	25.0%	9	22.5%
Cardiomyocytes	1519	62	4.1%	35	56.5%	28	45.2%	20	32.3%	18	29.0%
Endothelium	2492	101	4.1%	48	47.5%	42	41.6%	33	32.7%	30	29.7%
Erythroid	1566	90	5.7%	22	24.4%	15	16.7%	9	10.0%	7	7.8%
Extraembryonic Ectoderm	354	16	4.5%	7	43.8%	6	37.5%	3	18.8%	3	18.8%
Forebrain	1550	215	13.9%	166	77.2%	161	74.9%	151	70.2%	147	68.4%
Gut	1284	86	6.7%	47	54.7%	46	53.5%	28	32.6%	28	32.6%
Mid/Hindbrain	1726	206	11.9%	163	79.1%	156	75.7%	151	73.3%	143	69.4%
Mesenchyme	1032	43	4.2%	19	44.2%	17	39.5%	17	39.5%	16	37.2%
Mixed Mesoderm	588	58	9.9%	37	63.8%	35	60.3%	28	48.3%	26	44.8%
Neural Crest	1156	84	7.3%	53	63.1%	52	61.9%	50	59.5%	49	58.3%
Neuro-Mesodermal Progenitor	2107	235	11.2%	152	64.7%	144	61.3%	128	54.5%	121	51.5%
Notochord	149	8	5.4%	5	62.5%	5	62.5%	3	37.5%	3	37.5%
Paraxial Mesoderm	1166	104	8.9%	68	65.4%	65	62.5%	57	54.8%	55	52.9%
Pharyngeal Mesoderm	974	69	7.1%	38	55.1%	37	53.6%	38	55.1%	37	53.6%
Somitic Mesoderm	2029	146	7.2%	89	61.0%	82	56.2%	70	47.9%	65	44.5%
Spinal Cord	2728	353	12.9%	272	77.1%	257	72.8%	244	69.1%	229	64.9%
Surface Ectoderm	1245	91	7.3%	49	53.8%	47	51.6%	32	35.2%	32	35.2%
Ubiquitous	857	108	12.6%	94	87.0%	91	84.3%	22	20.4%	22	20.4%

Supplementary Table 25. Developmental patterning genes used for fin transcriptome PCA.

[see separate .xls file]

Supplementary Table 26. ATAC-Seq profile of putative *fgf8* gene regulatory regions in bowfin.

Region		Location Gar	Location Bowfin	ATAC-Seq peak (Ballard stages)						
**Mouse** (ref. 1)	**Zebrafish** (ref. 2)			20-21	22-23	23-24	24-25	26-27	28-29	30-31
CE39	fgf.dr22	LG5:30947677-30948088	Aca_scaf_19:20039103-20039514	ATAC peak	ATAC peak	ATAC peak	ATAC peak	ATAC peak	ATAC peak	ATAC peak
CE40	fgf.dr14	LG5:30950223-30950719	Aca_scaf_19:20036292-20036744	no peak	no peak	no peak	ATAC peak	no peak	no peak	no peak
	fgf.dr4	LG5:30992807-30992954	Aca_scaf_19:19999288-19999434	no peak	no peak	no peak	no peak	no peak	no peak	no peak
	fgf.dr5	LG5:30994709-30995125	Aca_scaf_19:19996896-19997335	no peak	no peak	no peak	no peak	no peak	no peak	no peak
CE52	fgf.dr6	LG5:31007087-31007580	Aca_scaf_19:19985590-19986107	no peak	no peak	no peak	ATAC peak	ATAC peak	ATAC peak	ATAC peak
	fgf.dr21	LG5:31011442-31011566	Aca_scaf_19:19982750-19983108	no peak	no peak	no peak	ATAC peak	no peak	no peak	no peak
	fgf.dr20	LG5:31031807-31031940	Aca_scaf_19:19964282-19964154	no peak	no peak	no peak	no peak	no peak	no peak	no peak
CE83	fgf.dr7	LG5:31083803-31084013	Aca_scaf_19:19919190-19919363	no peak	no peak	no peak	no peak	no peak	no peak	no peak
CE80	fgf.dr15	LG5:31096149-31096261	Aca_scaf_19:19907658-19907719	no peak	no peak	no peak	no peak	no peak	no peak	no peak
	fgf.dr16	LG5:31099373-31099672	Aca_scaf_19:19904756-19905061	ATAC peak	no peak	no peak	no peak	no peak	no peak	no peak
CE79	fgf.dr1/8	LG5:31101477-31101648	Aca_scaf_19:19902701-19902861	no peak	ATAC peak	ATAC peak	ATAC peak	ATAC peak	no peak	no peak
CE70	fgf.dr2/9	LG5:31115298-31115473	Aca_scaf_19:19893350-19893526	no peak	no peak	no peak	no peak	no peak	no peak	no peak
CE66		LG5:31119662-31119764	Aca_scaf_19:19889478-19889663	no peak	no peak	no peak	no peak	no peak	no peak	no peak
CE64	fgf.dr10	LG5:31124736-31125137	Aca_scaf_19:19885965-19886447	no peak	no peak	no peak	no peak	no peak	no peak	no peak
CE62	fgf.dr3/11	LG5:31132406-31133156	Aca_scaf_19:19878997-19879728	ATAC peak	ATAC peak	ATAC peak	no peak	no peak	no peak	no peak
CE59		LG5:31141354-31141592	Aca_scaf_19:19873851-19874071	no peak	no peak	no peak	ATAC peak	no peak	ATAC peak	ATAC peak
CE60		LG5:31146201-31147373	Aca_scaf_19:19874776-19875263	no peak	no peak	no peak	no peak	no peak	no peak	no peak
	fgf.dr12	LG5:31146831-31147276	Aca_scaf_19:19870268-19870878	no peak	no peak	no peak	no peak	no peak	no peak	no peak
CE58	fgf.dr13	LG5:31153080-31153525	Aca_scaf_19:19867354-19867788	ATAC peak	ATAC peak	ATAC peak	ATAC peak	no peak	ATAC peak	ATAC peak
	fgf.dr18	LG5:31177489-31177812	Aca_scaf_19:19852686-19853018	ATAC peak	ATAC peak	ATAC peak	ATAC peak	ATAC peak	no peak	no peak

ATAC peak
no peak

References:
1. Marinic M, Aktas T, Ruf S, & Spitz F (2013) Dev Cell 24(5):530-542.
2. Komisarczuk AZ, Kawakami K, & Becker TS (2009) Dev Biol 336(2):301-312.

Supplementary References

1 Chapman, J. A. *et al.* Meraculous: de novo genome assembly with short paired-end reads. *PLoS One* **6**, e23501, doi:10.1371/journal.pone.0023501 (2011).
2 Putnam, N. H. *et al.* Chromosome-scale shotgun assembly using an in vitro method for long-range linkage. *Genome Res* **26**, 342-350, doi:10.1101/gr.193474.115 (2016).
3 Hardie, D. C. & Hebert, P. D. N. Genome-size evolution in fishes. *Canadian Journal of Fisheries and Aquatic Sciences* **61**, 1636-1646 (2004).
4 Ohno, S. *et al.* Microchromosomes in holocephalian, chondrostean and holostean fishes. *Chromosoma* **26**, 35-40, doi:10.1007/BF00319498 (1969).
5 Majtanova, Z., Symonova, R., Arias-Rodriguez, L., Sallan, L. & Rab, P. "Holostei versus Halecostomi" Problem: Insight from Cytogenetics of Ancient Nonteleost Actinopterygian Fish, Bowfin Amia calva. *J Exp Zool B Mol Dev Evol* **328**, 620-628, doi:10.1002/jez.b.22720 (2017).
6 Simao, F. A., Waterhouse, R. M., Ioannidis, P., Kriventseva, E. V. & Zdobnov, E. M. BUSCO: assessing genome assembly and annotation completeness with single-copy orthologs. *Bioinformatics* **31**, 3210-3212, doi:10.1093/bioinformatics/btv351 (2015).
7 Parra, G., Bradnam, K. & Korf, I. CEGMA: a pipeline to accurately annotate core genes in eukaryotic genomes. *Bioinformatics* **23**, 1061-1067, doi:10.1093/bioinformatics/btm071 (2007).
8 Braasch, I. *et al.* The spotted gar genome illuminates vertebrate evolution and facilitates human-teleost comparisons. *Nat Genet* **48**, 427-437, doi:10.1038/ng.3526 (2016).
9 Holt, C. & Yandell, M. MAKER2: an annotation pipeline and genome-database management tool for second-generation genome projects. *BMC Bioinformatics* **12**, 491, doi:10.1186/1471-2105-12-491 (2011).
10 Bateman, A. *et al. The Pfam Protein Families Database. http://www.sanger.ac.uk/Software/Pfam/*, <http://www.sanger.ac.uk/Software/Pfam/> (2000).
11 Emms, D. M. & Kelly, S. OrthoFinder: phylogenetic orthology inference for comparative genomics. *Genome Biol* **20**, 238, doi:10.1186/s13059-019-1832-y (2019).
12 Burr, B. M. & Bennett, M. G. in *Freshwater Fishes of North America* Vol. 1 (eds M. L. Warren & M. G. Burr) (John Hopkins Univesity Press, 2014).
13 Dean, R. & Mank, J. E. The role of sex chromosomes in sexual dimorphism: discordance between molecular and phenotypic data. *J Evol Biol* **27**, 1443-1453, doi:10.1111/jeb.12345 (2014).
14 Wen, M. *et al.* Sex chromosome and sex locus characterization in goldfish, Carassius auratus (Linnaeus, 1758). *BMC Genomics* **21**, 552, doi:10.1186/s12864-020-06959-3 (2020).
15 Zhang, A. *et al.* Computational identification of Y-linked markers and genes in the grass carp genome by using a pool-and-sequence method. *Sci Rep* **7**, 8213, doi:10.1038/s41598-017-08476-y (2017).
16 Darolti, I. *et al.* Extreme heterogeneity in sex chromosome differentiation and dosage compensation in livebearers. *Proc Natl Acad Sci U S A* **116**, 19031-19036, doi:10.1073/pnas.1905298116 (2019).
17 Li, S. *et al.* A new approach for comprehensively describing heterogametic sex chromosomes. *DNA Res* **25**, 375-382, doi:10.1093/dnares/dsy010 (2018).
18 Li, H. & Durbin, R. Fast and accurate short read alignment with Burrows-Wheeler transform. *Bioinformatics* **25**, 1754-1760, doi:10.1093/bioinformatics/btp324 (2009).
19 Li, H. *et al.* The Sequence Alignment/Map format and SAMtools. *Bioinformatics* **25**, 2078-2079, doi:10.1093/bioinformatics/btp352 (2009).
20 Marcais, G. & Kingsford, C. A fast, lock-free approach for efficient parallel counting of occurrences of k-mers. *Bioinformatics* **27**, 764-770, doi:10.1093/bioinformatics/btr011 (2011).
21 Morris, J., Darolti, I., Bloch, N. I., Wright, A. E. & Mank, J. E. Shared and Species-Specific Patterns of Nascent Y Chromosome Evolution in Two Guppy Species. *Genes (Basel)* **9**, doi:10.3390/genes9050238 (2018).
22 Moore, E. C. & Roberts, R. B. Polygenic sex determination. *Curr Biol* **23**, R510-512, doi:10.1016/j.cub.2013.04.004 (2013).
23 Baroiller, J. F., D'Cotta, H. & Saillant, E. Environmental effects on fish sex determination and differentiation. *Sex Dev* **3**, 118-135, doi:10.1159/000223077 (2009).

24 Kamiya, T. *et al.* A trans-species missense SNP in Amhr2 is associated with sex determination in the tiger pufferfish, Takifugu rubripes (fugu). *PLoS Genet* **8**, e1002798, doi:10.1371/journal.pgen.1002798 (2012).
25 Feron, R. *et al.* RADSex: A computational workflow to study sex determination using restriction site-associated DNA sequencing data. *Mol Ecol Resour*, doi:10.1111/1755-0998.13360 (2021).
26 Miller, M. A., Pfeiffer, W. & Schwartz, T. Creating the CIPRES Science Gateway for inference of large phylogenetic trees. *Proceedings of the Gateway Computing Environments Workshop (GCE)*, 1-8 (2010).
27 Stamatakis, A. RAxML version 8: a tool for phylogenetic analysis and post-analysis of large phylogenies. *Bioinformatics* **30**, 1312-1313, doi:10.1093/bioinformatics/btu033 (2014).
28 Bouckaert, R. *et al.* BEAST 2.5: An advanced software platform for Bayesian evolutionary analysis. *PLoS Comput Biol* **15**, e1006650, doi:10.1371/journal.pcbi.1006650 (2019).
29 Emms, D. M. & Kelly, S. STAG: Species Tree Inference from All Genes. *bioRxiv*, 267914, doi:10.1101/267914 (2018).
30 Near, T. J. *et al.* Resolution of ray-finned fish phylogeny and timing of diversification. *Proc Natl Acad Sci U S A* **109**, 13698-13703, doi:10.1073/pnas.1206625109 (2012).
31 Betancur, R. R. *et al.* The tree of life and a new classification of bony fishes. *PLoS currents* **5**, doi:10.1371/currents.tol.53ba26640df0ccaee75bb165c8c26288 (2013).
32 Broughton, R. E., Betancur, R. R., Li, C., Arratia, G. & Orti, G. Multi-locus phylogenetic analysis reveals the pattern and tempo of bony fish evolution. *PLoS currents* **5**, doi:10.1371/currents.tol.2ca8041495ffafd0c92756e75247483e (2013).
33 Faircloth, B. C., Sorenson, L., Santini, F. & Alfaro, M. E. A Phylogenomic Perspective on the Radiation of Ray-Finned Fishes Based upon Targeted Sequencing of Ultraconserved Elements (UCEs). *PLoS ONE* **8**, doi:10.1371/journal.pone.0065923 (2013).
34 Hughes, L. C. *et al.* Comprehensive phylogeny of ray-finned fishes (Actinopterygii) based on transcriptomic and genomic data. *Proc Natl Acad Sci U S A* **115**, 6249-6254, doi:10.1073/pnas.1719358115 (2018).
35 Bi, X. *et al.* Tracing the genetic footprints of vertebrate landing in non-teleost ray-finned fishes. *Cell* **184**, 1377-1391 e1314, doi:10.1016/j.cell.2021.01.046 (2021).
36 Irisarri, I. *et al.* Phylotranscriptomic consolidation of the jawed vertebrate timetree. *Nat Ecol Evol* **1**, 1370-1378, doi:10.1038/s41559-017-0240-5 (2017).
37 Hauptmann, G. & Bahram, S. Genetics of the central MHC. *Curr Opin Immunol* **16**, 668-672, doi:10.1016/j.coi.2004.07.001 (2004).
38 Trowsdale, J. The MHC, disease and selection. *Immunol Lett* **137**, 1-8, doi:10.1016/j.imlet.2011.01.002 (2011).
39 Ohta, Y. *et al.* Primitive synteny of vertebrate major histocompatibility complex class I and class II genes. *Proc Natl Acad Sci U S A* **97**, 4712-4717, doi:10.1073/pnas.97.9.4712 (2000).
40 Grimholt, U. MHC and Evolution in Teleosts. *Biology (Basel)* **5**, 6, doi:10.3390/biology5010006 (2016).
41 Yamaguchi, T. & Dijkstra, J. M. Major Histocompatibility Complex (MHC) Genes and Disease Resistance in Fish. *Cells* **8**, 378, doi:10.3390/cells8040378 (2019).
42 Altschul, S. F., Gish, W., Miller, W., Myers, E. W. & Lipman, D. J. Basic local alignment search tool. *Journal of molecular biology* **215**, 403-410, doi:10.1016/S0022-2836(05)80360-2 (1990).
43 Thompson, J. D., Higgins, D. G. & Gibson, T. J. CLUSTAL W: improving the sensitivity of progressive multiple sequence alignment through sequence weighting, position-specific gap penalties and weight matrix choice. *Nucleic Acids Res* **22**, 4673-4680, doi:10.1093/nar/22.22.4673 (1994).
44 Kumar, S., Stecher, G., Li, M., Knyaz, C. & Tamura, K. MEGA X: Molecular Evolutionary Genetics Analysis across Computing Platforms. *Mol Biol Evol* **35**, 1547-1549, doi:10.1093/molbev/msy096 (2018).
45 Grimholt, U. *et al.* A comprehensive analysis of teleost MHC class I sequences. *BMC Evol Biol* **15**, 32, doi:10.1186/s12862-015-0309-1 (2015).
46 Grimholt, U., Tsukamoto, K., Hashimoto, K. & Dijkstra, J. M. Discovery of a Novel MHC Class I Lineage in Teleost Fish which Shows Unprecedented Levels of Ectodomain Deterioration while Possessing an Impressive Cytoplasmic Tail Motif. *Cells* **8**, 1056, doi:10.3390/cells8091056 (2019).

47 Swann, J. B., Holland, S. J., Petersen, M., Pietsch, T. W. & Boehm, T. The immunogenetics of sexual parasitism. *Science* **369**, 1608-1615, doi:10.1126/science.aaz9445 (2020).
48 Dijkstra, J. M., Grimholt, U., Leong, J., Koop, B. F. & Hashimoto, K. Comprehensive analysis of MHC class II genes in teleost fish genomes reveals dispensability of the peptide-loading DM system in a large part of vertebrates. *BMC Evol Biol* **13**, 260, doi:10.1186/1471-2148-13-260 (2013).
49 McConnell, S. C. *et al.* Alternative haplotypes of antigen processing genes in zebrafish diverged early in vertebrate evolution. *Proc Natl Acad Sci U S A* **113**, E5014-5023, doi:10.1073/pnas.1607602113 (2016).
50 Traver, D. & Yoder, J. A. in *The Zebrafish in Biomedical Research* (eds S. Cartner *et al.*) 191–216 (Academic Press, 2020).
51 Dirscherl, H., McConnell, S. C., Yoder, J. A. & de Jong, J. L. The MHC class I genes of zebrafish. *Dev Comp Immunol* **46**, 11-23, doi:10.1016/j.dci.2014.02.018 (2014).
52 Flajnik, M. F. A cold-blooded view of adaptive immunity. *Nat Rev Immunol* **18**, 438-453, doi:10.1038/s41577-018-0003-9 (2018).
53 Flajnik, M. F. & Kasahara, M. Origin and evolution of the adaptive immune system: genetic events and selective pressures. *Nat Rev Genet* **11**, 47-59, doi:10.1038/nrg2703 (2010).
54 Mirete-Bachiller, S., Olivieri, D. N. & Gambon-Deza, F. Immunoglobulin T genes in Actinopterygii. *Fish Shellfish Immunol* **108**, 86-93, doi:10.1016/j.fsi.2020.11.027 (2021).
55 Piazzon, M. C. *et al.* Differential Modulation of IgT and IgM upon Parasitic, Bacterial, Viral, and Dietary Challenges in a Perciform Fish. *Front Immunol* **7**, 637, doi:10.3389/fimmu.2016.00637 (2016).
56 Salinas, I. The Mucosal Immune System of Teleost Fish. *Biology (Basel)* **4**, 525-539, doi:10.3390/biology4030525 (2015).
57 Zhang, Y. A. *et al.* IgT, a primitive immunoglobulin class specialized in mucosal immunity. *Nat Immunol* **11**, 827-835, doi:10.1038/ni.1913 (2010).
58 Guselnikov, S. V. *et al.* Diversity of Immunoglobulin Light Chain Genes in Non-Teleost Ray-Finned Fish Uncovers IgL Subdivision into Five Ancient Isotypes. *Front Immunol* **9**, 1079, doi:10.3389/fimmu.2018.01079 (2018).
59 Rast, J. P. *et al.* alpha, beta, gamma, and delta T cell antigen receptor genes arose early in vertebrate phylogeny. *Immunity* **6**, 1-11, doi:10.1016/s1074-7613(00)80237-x (1997).
60 Aoki, T., Hikima, J., Hwang, S. D. & Jung, T. S. Innate immunity of finfish: primordial conservation and function of viral RNA sensors in teleosts. *Fish Shellfish Immunol* **35**, 1689-1702, doi:10.1016/j.fsi.2013.02.005 (2013).
61 Fitzgerald, K. A. & Kagan, J. C. Toll-like Receptors and the Control of Immunity. *Cell* **180**, 1044-1066, doi:10.1016/j.cell.2020.02.041 (2020).
62 Quiniou, S. M., Boudinot, P. & Bengten, E. Comprehensive survey and genomic characterization of Toll-like receptors (TLRs) in channel catfish, Ictalurus punctatus: identification of novel fish TLRs. *Immunogenetics* **65**, 511-530, doi:10.1007/s00251-013-0694-9 (2013).
63 Wcisel, D. J., Ota, T., Litman, G. W. & Yoder, J. A. Spotted Gar and the Evolution of Innate Immune Receptors. *J Exp Zool B Mol Dev Evol* **328**, 666-684, doi:10.1002/jez.b.22738 (2017).
64 Sievers, F. & Higgins, D. G. Clustal Omega. *Current Protocols in Bioinformatics* **48**, 3.13.11-13.13.16, doi:10.1002/0471250953.bi0313s48 (2014).
65 Pasquier, J. *et al.* Gene evolution and gene expression after whole genome duplication in fish: the PhyloFish database. *BMC Genomics* **17**, 368, doi:10.1186/s12864-016-2709-z (2016).
66 Minh, B. Q. *et al.* IQ-TREE 2: New Models and Efficient Methods for Phylogenetic Inference in the Genomic Era. *Mol Biol Evol* **37**, 1530-1534, doi:10.1093/molbev/msaa015 (2020).
67 Minh, B. Q., Nguyen, M. A. & von Haeseler, A. Ultrafast approximation for phylogenetic bootstrap. *Mol Biol Evol* **30**, 1188-1195, doi:10.1093/molbev/mst024 (2013).
68 Hoang, D. T. *et al.* MPBoot: fast phylogenetic maximum parsimony tree inference and bootstrap approximation. *BMC Evol Biol* **18**, 11, doi:10.1186/s12862-018-1131-3 (2018).
69 Finn, R. D., Clements, J. & Eddy, S. R. HMMER web server: interactive sequence similarity searching. *Nucleic Acids Res* **39**, W29-37, doi:10.1093/nar/gkr367 (2011).
70 Kawasaki, K. *et al.* SCPP Genes and Their Relatives in Gar: Rapid Expansion of Mineralization Genes in Osteichthyans. *J Exp Zool B Mol Dev Evol* **328**, 645-665, doi:10.1002/jez.b.22755 (2017).

71 Kawasaki, K. & Weiss, K. M. Mineralized tissue and vertebrate evolution: the secretory calcium-binding phosphoprotein gene cluster. *Proc Natl Acad Sci U S A* **100**, 4060-4065, doi:10.1073/pnas.0638023100 (2003).
72 Afgan, E. *et al.* The Galaxy platform for accessible, reproducible and collaborative biomedical analyses: 2016 update. *Nucleic Acids Res* **44**, W3-W10, doi:10.1093/nar/gkw343 (2016).
73 Mansfeld, J. *et al.* Branched-chain amino acid catabolism is a conserved regulator of physiological ageing. *Nat Commun* **6**, 10043, doi:10.1038/ncomms10043 (2015).
74 Bolger, A. M., Lohse, M. & Usadel, B. Trimmomatic: a flexible trimmer for Illumina sequence data. *Bioinformatics* **30**, 2114-2120, doi:10.1093/bioinformatics/btu170 (2014).
75 Dobin, A. *et al.* STAR: ultrafast universal RNA-seq aligner. *Bioinformatics* **29**, 15-21, doi:10.1093/bioinformatics/bts635 (2013).
76 Kapustin, Y., Souvorov, A., Tatusova, T. & Lipman, D. Splign: algorithms for computing spliced alignments with identification of paralogs. *Biol Direct* **3**, 20, doi:10.1186/1745-6150-3-20 (2008).
77 Trapnell, C. *et al.* Transcript assembly and quantification by RNA-Seq reveals unannotated transcripts and isoform switching during cell differentiation. *Nat Biotechnol* **28**, 511-515, doi:10.1038/nbt.1621 (2010).
78 Liu, Z. *et al.* The channel catfish genome sequence provides insights into the evolution of scale formation in teleosts. *Nat Commun* **7**, 11757, doi:10.1038/ncomms11757 (2016).
79 Kawasaki, K. *et al.* Coevolution of enamel, ganoin, enameloid, and their matrix SCPP genes in osteichthyans. *iScience* **24**, 102023, doi:10.1016/j.isci.2020.102023 (2021).
80 Cheng, P. *et al.* The American paddlefish genome provides novel insights into chromosomal evolution and bone mineralization in early vertebrates. *Mol Biol Evol*, doi:10.1093/molbev/msaa326 (2020).
81 Grande, L. & Bemis, W. E. Osteology and Phylogenetic Relationships of Fossil and Recent Paddlefishes (Polyodontidae) with Comments on the Interrelationships of Acipenseriformes. *Journal of Vertebrate Paleontology* **11**, 1-121, doi:10.1080/02724634.1991.10011424 (1991).
82 Findeis, E. K. Osteology and phylogenetic interrelationships of sturgeons (Acipenseridae). *Environmental Biology of Fishes* **48**, 73-126, doi:10.1023/A:1007372832213 (1997).
83 Miyabe, K. *et al.* GSP-37, a novel goldfish scale matrix protein: identification, localization and functional analysis. *Faraday Discussions* **159**, 463-481, doi:10.1039/C2FD20051A (2012).
84 Buenrostro, J. D., Wu, B., Chang, H. Y. & Greenleaf, W. J. ATAC-seq: A Method for Assaying Chromatin Accessibility Genome-Wide. *Curr Protoc Mol Biol* **109**, 21 29 21-21 29 29, doi:10.1002/0471142727.mb2129s109 (2015).
85 Heinz, S. *et al.* Simple Combinations of Lineage-Determining Transcription Factors Prime cis-Regulatory Elements Required for Macrophage and B Cell Identities. *Molecular Cell* **38**, 576-589, doi:10.1016/j.molcel.2010.05.004 (2010).
86 Armstrong, J. *et al.* Progressive Cactus is a multiple-genome aligner for the thousand-genome era. *Nature* **587**, 246-251, doi:10.1038/s41586-020-2871-y (2020).
87 Quinlan, A. R. & Hall, I. M. BEDTools: A flexible suite of utilities for comparing genomic features. *Bioinformatics* **26**, 841-842, doi:10.1093/bioinformatics/btq033 (2010).
88 Visel, A., Minovitsky, S., Dubchak, I. & Pennacchio, L. A. VISTA Enhancer Browser - A database of tissue-specific human enhancers. *Nucleic Acids Research* **35**, doi:10.1093/nar/gkl822 (2007).
89 Pijuan-Sala, B. *et al.* Single-cell chromatin accessibility maps reveal regulatory programs driving early mouse organogenesis. *Nature Cell Biology* **22**, 487-497, doi:10.1038/s41556-020-0489-9 (2020).
90 Babarinde, I. A. & Saitou, N. The Dynamics, Causes, and Impacts of Mammalian Evolutionary Rates Revealed by the Analyses of Capybara Draft Genome Sequences. *Genome Biol Evol* **12**, 1444-1458, doi:10.1093/gbe/evaa157 (2020).
91 Graham, J. B. *Air-Breathing Fishes*. (Academic Press, 1997).
92 Cass, A. N., Servetnick, M. D. & McCune, A. R. Expression of a lung developmental cassette in the adult and developing zebrafish swimbladder. *Evol Dev* **15**, 119-132, doi:10.1111/ede.12022 (2013).
93 Liem, K. F. Form and function of lungs: the evolution of air breathing mechanisms. *American Zoologist* **28**, 739-759 (1988).
94 Perry, S. F., Wilson, R. J., Straus, C., Harris, M. B. & Remmers, J. E. Which came first, the lung or the breath? *Comp Biochem Physiol A Mol Integr Physiol* **129**, 37-47 (2001).

95 Zheng, W. *et al.* Comparative transcriptome analyses indicate molecular homology of zebrafish swimbladder and mammalian lung. *PLoS One* **6**, e24019, doi:10.1371/journal.pone.0024019 (2011).
96 Meyer, A. *et al.* Giant lungfish genome elucidates the conquest of land by vertebrates. *Nature* **590**, 284-289, doi:10.1038/s41586-021-03198-8 (2021).
97 Wang, K. *et al.* African lungfish genome sheds light on the vertebrate water-to-land transition. *Cell* **184**, 1362-1376 e1318, doi:10.1016/j.cell.2021.01.047 (2021).
98 Funk, E., Lencer, E. & McCune, A. Dorsoventral inversion of the air-filled organ (lungs, gas bladder) in vertebrates: RNAsequencing of laser capture microdissected embryonic tissue. *J Exp Zool B Mol Dev Evol*, doi:10.1002/jez.b.22998 (2020).
99 Funk, E. C., Breen, C., Sanketi, B. D., Kurpios, N. & McCune, A. Changes in Nkx2.1, Sox2, Bmp4 and Bmp16 expression underlying the lung-to-gas bladder evolutionary transition in ray-finned fishes. *Evolution and Development* **in press** (2020).
100 Funk, E. C., Birol, E. B. & McCune, A. R. Does the bowfin gas bladder represent an intermediate stage during the lung-to-gas bladder evolutionary transition? *J Morphol* **282**, 600-611, doi:10.1002/jmor.21330 (2021).
101 Tatsumi, N. *et al.* Molecular developmental mechanism in polypterid fish provides insight into the origin of vertebrate lungs. *Sci Rep* **6**, 30580, doi:10.1038/srep30580 (2016).
102 Menke, D. B., Guenther, C. & Kingsley, D. M. Dual hindlimb control elements in the Tbx4 gene and region-specific control of bone size in vertebrate limbs. *Development* **135**, 2543-2553, doi:10.1242/dev.017384 (2008).
103 Nikaido, M. *et al.* Coelacanth genomes reveal signatures for evolutionary transition from water to land. *Genome Res* **23**, 1740-1748, doi:10.1101/gr.158105.113 (2013).
104 Zhang, W. *et al.* Spatial-temporal targeting of lung-specific mesenchyme by a Tbx4 enhancer. *BMC Biol* **11**, 111, doi:10.1186/1741-7007-11-111 (2013).
105 Brito, P. M., Meunier, F. J., Clement, G. & Geffard-Kuriyama, D. The Histological Structure of the Calcified Lung of the Fossil Coelacanth Axelrodichthys Araripensis (Actinistia: Mawsoniidae). *Palaeontology* **53**, 1281-1290, doi:Doi 10.1111/J.1475-4983.2010.01015.X (2010).
106 Hara, Y. *et al.* Shark genomes provide insights into elasmobranch evolution and the origin of vertebrates. *Nat Ecol Evol* **2**, 1761-1771, doi:10.1038/s41559-018-0673-5 (2018).
107 Onimaru, K. The evolutionary origin of developmental enhancers in vertebrates: Insights from non-model species. *Dev Growth Differ* **62**, 326-333, doi:10.1111/dgd.12662 (2020).
108 Cao, J. *et al.* A human cell atlas of fetal gene expression. *Science* **370**, doi:10.1126/science.aba7721 (2020).
109 Domcke, S. *et al.* A human cell atlas of fetal chromatin accessibility. *Science* **370**, doi:10.1126/science.aba7612 (2020).
110 Frazer, K. A., Pachter, L., Poliakov, A., Rubin, E. M. & Dubchak, I. VISTA: computational tools for comparative genomics. *Nucleic Acids Res* **32**, W273-279, doi:10.1093/nar/gkh458 (2004).
111 Brudno, M. *et al.* Glocal alignment: finding rearrangements during alignment. *Bioinformatics* **19 Suppl 1**, i54-62, doi:10.1093/bioinformatics/btg1005 (2003).
112 Stanke, M. & Waack, S. Gene prediction with a hidden Markov model and a new intron submodel. *Bioinformatics* **19 Suppl 2**, ii215-225, doi:10.1093/bioinformatics/btg1080 (2003).
113 Bian, C. *et al.* The Asian arowana (Scleropages formosus) genome provides new insights into the evolution of an early lineage of teleosts. *Sci Rep* **6**, 24501, doi:10.1038/srep24501 (2016).
114 Martin, K. J. & Holland, P. W. H. Diversification of Hox Gene Clusters in Osteoglossomorph Fish in Comparison to Other Teleosts and the Spotted Gar Outgroup. *J Exp Zool B Mol Dev Evol* **328**, 638-644, doi:10.1002/jez.b.22726 (2017).
115 Long, W. L. & Ballard, W. W. Normal embryonic stages of the longnose gar, Lepisosteus osseus. *BMC developmental biology* **1**, 6 (2001).
116 Ballard, W. W. Stages and rates of normal development in the holostean fish, Amia calva. *Journal of Experimental Zoology* **238**, 337-354, doi:10.1002/jez.1402380308 (1986).
117 Gehrke, A. R. & Shubin, N. H. Cis-regulatory programs in the development and evolution of vertebrate paired appendages. *Semin Cell Dev Biol* **57**, 31-39, doi:10.1016/j.semcdb.2016.01.015 (2016).

118 Hornblad, A., Bastide, S., Langenfeld, K., Langa, F. & Spitz, F. Dissection of the Fgf8 regulatory landscape by in vivo CRISPR-editing reveals extensive intra- and inter-enhancer redundancy. *Nat Commun* **12**, 439, doi:10.1038/s41467-020-20714-y (2021).
119 Komisarczuk, A. Z., Kawakami, K. & Becker, T. S. Cis-regulation and chromosomal rearrangement of the fgf8 locus after the teleost/tetrapod split. *Dev Biol* **336**, 301-312, doi:10.1016/j.ydbio.2009.09.029 (2009).
120 Marinic, M., Aktas, T., Ruf, S. & Spitz, F. An integrated holo-enhancer unit defines tissue and gene specificity of the Fgf8 regulatory landscape. *Dev Cell* **24**, 530-542, doi:10.1016/j.devcel.2013.01.025 (2013).
121 Niswander, L. & Martin, G. R. Fgf-4 expression during gastrulation, myogenesis, limb and tooth development in the mouse. *Development* **114**, 755-768 (1992).
122 Colvin, J. S., Feldman, B., Nadeau, J. H., Goldfarb, M. & Ornitz, D. M. Genomic organization and embryonic expression of the mouse fibroblast growth factor 9 gene. *Dev Dyn* **216**, 72-88, doi:10.1002/(SICI)1097-0177(199909)216:1<72::AID-DVDY9>3.0.CO;2-9 (1999).
123 Sun, X. *et al.* Conditional inactivation of Fgf4 reveals complexity of signalling during limb bud development. *Nat Genet* **25**, 83-86, doi:10.1038/75644 (2000).
124 Yonei-Tamura, S. *et al.* FGF7 and FGF10 directly induce the apical ectodermal ridge in chick embryos. *Dev Biol* **211**, 133-143, doi:10.1006/dbio.1999.9290 (1999).
125 Nakamura, T. *et al.* Molecular mechanisms underlying the exceptional adaptations of batoid fins. *Proc Natl Acad Sci U S A* **112**, 15940-15945, doi:10.1073/pnas.1521818112 (2015).
126 Fischer, S., Draper, B. W. & Neumann, C. J. The zebrafish fgf24 mutant identifies an additional level of Fgf signaling involved in vertebrate forelimb initiation. *Development* **130**, 3515-3524, doi:10.1242/dev.00537 (2003).
127 Amemiya, C. T. *et al.* The African coelacanth genome provides insights into tetrapod evolution. *Nature* **496**, 311-316, doi:10.1038/nature12027 (2013).
128 Oulion, S., Bertrand, S. & Escriva, H. Evolution of the FGF Gene Family. *Int J Evol Biol* **2012**, 298147, doi:10.1155/2012/298147 (2012).
129 Boudinot, P. *et al.* A tetrapod-like repertoire of innate immune receptors and effectors for coelacanths. *J Exp Zool B Mol Dev Evol* **322**, 415-437, doi:10.1002/jez.b.22559 (2014).
