# Supplementary material for: The bowfin genome illuminates the developmental evolution of ray-finned fishes
Source: Nat Genet. 2021 Aug 30;53(9):1373–84. doi: 10.1038/s41588-021-00914-y (PMC8423624; doi:10.1038/s41588-021-00914-y)
Supplement: Supplementary file 2 — Reporting Summary [file 41588_2021_914_MOESM2_ESM.pdf]

nature research

Corresponding author(s): Ingo Braasch

Last updated by author(s): Jun 19, 2021

Reporting Summary

Nature Research wishes to improve the reproducibility of the work that we publish. This form provides structure for consistency and transparency in reporting. For further information on Nature Research policies, see our Editorial Policies and the Editorial Policy Checklist.

Statistics

For all statistical analyses, confirm that the following items are present in the figure legend, table legend, main text, or Methods section.

n/a	Confirmed	
☐	☒	The exact sample size (n) for each experimental group/condition, given as a discrete number and unit of measurement
☐	☒	A statement on whether measurements were taken from distinct samples or whether the same sample was measured repeatedly
☐	☒	The statistical test(s) used AND whether they are one- or two-sided<br>*Only common tests should be described solely by name; describe more complex techniques in the Methods section.*
☐	☒	A description of all covariates tested
☐	☒	A description of any assumptions or corrections, such as tests of normality and adjustment for multiple comparisons
☒	☐	A full description of the statistical parameters including central tendency (e.g. means) or other basic estimates (e.g. regression coefficient) AND variation (e.g. standard deviation) or associated estimates of uncertainty (e.g. confidence intervals)
☐	☒	For null hypothesis testing, the test statistic (e.g. F, t, r) with confidence intervals, effect sizes, degrees of freedom and P value noted<br>*Give P values as exact values whenever suitable.*
☐	☒	For Bayesian analysis, information on the choice of priors and Markov chain Monte Carlo settings
☒	☐	For hierarchical and complex designs, identification of the appropriate level for tests and full reporting of outcomes
☒	☐	Estimates of effect sizes (e.g. Cohen's d, Pearson's r), indicating how they were calculated

Our web collection on statistics for biologists contains articles on many of the points above.

Software and code

Policy information about availability of computer code

Data collection	Software was only used for data analyses.
Data analysis	Custom scripts and workflows for the Pool-Seq sex determination analysis are available at https://github.com/RomainFeron/paper-sexdetermination-bowfin, for the gene order phylogeny at https://github.com/DyogenIBENS/BowfinGOPhylogeny. Other software was used as described in detail in the Methods section. ATAC-Seq reads mapping to the mitochondrial genome were removed with removeChrom.py [available from https://github.com/harvardinformatics/ATAC-seq/blob/master/atacseq/removeChrom.py]. Other standard bioinformatic softwares were used and cited in the manuscript such as: Augustus v2.6.1, BEAST2 v2.6.3, BEDtools 2.27.1, BLAST, Bowtie v2.3.4.1, BUSCO v5, BWA v0.7.1718, CEGMA, CLC Genomics Workbench, ClustalW, Cufflinks, deepTools v3.5.1, Ensembl Compara, Exonerate v2.2.0, Galaxy, Geneious 9.1.8, HiRise, HMMER v3, HOMER v4.11, HiCExplorer v3.6, IGV v2.4.16, IQ-TREE, M-Coffee, MACS2 v2.2.6, MAKER v2.31, MEGAX, Meraculus, mVISTA, Orthofinder v1.1.3, Phylip, PicardTools v2.18.1, Progressive Cactus v.1.2.3, RAxML HPC-HYBRID-AVX , RepeatMasker v4.0.5, RepeatModeler v1.0.8, SAMtools v1.11, SNAP, SortmeRNA, TreeBeST, Trimmomatic v0.38, and Trinity v2.8.5.

For manuscripts utilizing custom algorithms or software that are central to the research but not yet described in published literature, software must be made available to editors and reviewers. We strongly encourage code deposition in a community repository (e.g. GitHub). See the Nature Research guidelines for submitting code & software for further information.

Data

Policy information about availability of data

All manuscripts must include a data availability statement. This statement should provide the following information, where applicable:

- Accession codes, unique identifiers, or web links for publicly available datasets
- A list of figures that have associated raw data
- A description of any restrictions on data availability

The bowfin reference genome assembly (AmiCal1) is available at GenBank under the accession number PESF00000000, raw reads at the Sequence Read Archive under accession numbers SRR14766073-SRR14766075. Transcriptomic and ATAC-Seq reads are available under accession numbers SRP281665 and SRP252716; assembled transcripts are available under accession number GIOP00000000. The MAKER gene annotation is available at https://github.com/AndrewWT/AmiaGenomics. Data for synteny analyses and the gene order phylogeny are available at https://github.com/DyogenIBENS/BowfinGOPhylogeny.

Field-specific reporting

Please select the one below that is the best fit for your research. If you are not sure, read the appropriate sections before making your selection.

☐ Life sciences ☐ Behavioural & social sciences ☒ Ecological, evolutionary & environmental sciences

For a reference copy of the document with all sections, see nature.com/documents/nr-reporting-summary-flat.pdf

Ecological, evolutionary & environmental sciences study design

All studies must disclose on these points even when the disclosure is negative.

Study description	Analyses consisted of standard bioinformatic pipelines. Detailed methods, and parameters and softwares used are described in the manuscript. When possible replicates were used to increase statistical power, and all data was confirmed to be of high quality before analyses.
Research sample	Both bowfin (Amia calva) and spotted gar (Lepisosteus oculatus) are not threatened. Samples were chosen from habitats where they are abundant and accessible. Samples were collected with the necessary permits and represent the Louisiana (adult bowfin, embryonic spotted gar) and Oneida Lake, NY (embryonic/larval bowfin) populations as described in the manuscript. Adult bowfin are sexually dimorphic and sex was determined after sacrifice upon dissection of gonads. Bowfin and gar developmental staging was performed by comparison of their morphology to that described in developmental staging literature.
Sampling strategy	Sampling procedures and sample sizes for individual experiments are provided in the Methods section. To obtain sufficient material, pooling of embryos and tissues was necessary for developmental ATAC-Seq and RNA-Seq experiments in a stage-specific, empirically determined manner as detailed in the Methods section. When possible, replicates were sequenced for developmental genetics. Data quality was assessed and determined to be of high quality before and after analyses as described in the manuscript.
Data collection	All data was collected in the laboratories of the authors or downloaded from repositories like NCBI Genbank, Ensembl, and the VISTA enhancer browser as well as the literature as described in the manuscript. Sequencing instruments used for the generation sequence data include Illumina HiSeq X Ten, Illumina NextSeq v2.5, and Illumina NovaSeq 6000 as detailed in the Methods section.
Timing and spatial scale	All developmental gene expression and chromatin analyses were performed on the same population of bowfin in Oneida Lake NY, Apr-Mar 2016 and Apr-Mar 2018 during the natural spawning season. Sample collection was stopped when the last desired developmental state of a sample was reached. All adult bowfin were collected around Thibodaux, Louisiana in Feb 2015, Apr 2017, and May 2018. Gar embryos were collected from a laboratory spawn in Apr 2018 as described in the manuscript.
Data exclusions	No data collected were excluded from analyses.
Reproducibility	A single reference genome assembly from a single adult male bowfin was generated. A single reference immune transcriptome was generated from a single adult female bowfin. Pool-Seq was performed with 30 males and 30 females. Developmental ATAC-Seq experiments were performed in 1-2 replicates per stage, developmental RNA-Seq experiments in 1-3 replicates per stage as detailed in the Methods section. RNA in situ hybridization was performed with 10 replicates per gene and per species as detailed in the Methods section. Photos of embryos are representative of at least 5 individuals per developmental stage. See Methods and figure legends for further details.
Randomization	Random samples were taken from pools of healthy adults and embryonic/larval clutches. To remove batch effects in sequence data, developmental ATAC and RNA reads were pooled into a single Illumina lane and normalized for comparisons to remove any technical differences in read depth.
Blinding	No blinding was necessary as all data were used for the genome assembly and sequence data generation and analyses thereof.

Did the study involve field work? ☒ Yes ☐ No

Field work, collection and transport

Field conditions	Animals were collected during spring March-May in both Lousiana and New York, when temperatures were warming prior to extensive annual habitat flooding. Bowfin and gar spawn during this time.
Location	Atchafalaya Basin (29°48'45.4"N 91°13'14.9"W) and Bayou Chevreuil (29°54'50.6"N 90°47'56.9"W) in Louisiana, and Oneida Lake, New York (43°12'36.6"N 75°55'18.2"W). Sampling was performed in water depths of 0.5-2m.
Access & import/export	All animals were sampled in accordance with permits from Nicholls State University (#IA046/IA053), Michigan State University (#10/16-179-00), and Cornell University (#2006-0013).
Disturbance	Disturbance was minimized by excluding by-catch with non-lethal electroshocking and capture with hand nets.

Reporting for specific materials, systems and methods

We require information from authors about some types of materials, experimental systems and methods used in many studies. Here, indicate whether each material, system or method listed is relevant to your study. If you are not sure if a list item applies to your research, read the appropriate section before selecting a response.

Materials & experimental systems

n/a	Involved in the study
☒	☐ Antibodies
☒	☐ Eukaryotic cell lines
☒	☐ Palaeontology and archaeology
☐	☒ Animals and other organisms
☒	☐ Human research participants
☒	☐ Clinical data
☒	☐ Dual use research of concern

Methods

n/a	Involved in the study
☒	☐ ChIP-seq
☒	☐ Flow cytometry
☒	☐ MRI-based neuroimaging

Animals and other organisms

Policy information about studies involving animals; ARRIVE guidelines recommended for reporting animal research

Laboratory animals	Embryos and larvae of Amia calva (bowfin) and Lepisosteus oculatus (spotted gar) were used as laboratory animals. Lab animals were humanely euthanized according to Institutional Animal Care and Use Committee (IACUC) protocols from Nicholls State University (#IA046/IA053), Michigan State University (#10/16-179-00), and Cornell University (#2006-0013).
Wild animals	Adult bowfin were captured in the Atchafalaya Basin and Bayou Chevreuil, Louisiana by electrofishing and transported to the laboratory in bayou water. DNA for the genome sample was taken from a sacrificed single adult male. Immune tissues (gill, spleen, intestine) were dissected from a sacrificed single adult female bowfin. For Pool-Seq analyses, fin clips were taken from 30 adult male and 30 adult female bowfins that were euthanized to allow for gonadal inspection by dissection. All animals were handled and euthanized according to Institutional Animal Care and Use Committee (IACUC) protocols from Nicholls State University (#IA046/IA053), Michigan State University (#10/16-179-00), and Cornell University (#2006-0013).
Field-collected samples	Bowfin embryos were collected from nests in Oneida Lake, New York. Eggs attached to nest material were collected with hand nets and transported to the laboratory in lake water with methylene blue added to abate fungal growth. In the laboratory, eggs were separated from nesting material by hand and placed in fresh lake water. Eggs and embryos were raised in static containers of lake water (16-18 degree Celsius) with a 12h dark/12h light photoperiod and moved to fresh water every other day. Embryos and larvae were sampled at the relevant stages following the bowfin staging series by Ballard (1986). Adult bowfin were captured in the Atchafalaya Basin and Bayou Chevreuil, Louisiana. At the end of experiments, all animals were euthanized following the approved IACUC protocols from Nicholls State University (#IA046/IA053), Michigan State University (#10/16-179-00), and Cornell University (#2006-0013).
Ethics oversight	Bowfin work was approved under Institutional Animal Care and Use Committee (IACUC) protocols from Nicholls State University (#IA046/IA053), Michigan State University (#10/16-179-00), and Cornell University (#2006-0013). Bowfins for genome sequencing, sex determination analysis, and immune transcriptomics were sampled in the Atchafalaya Basin and Bayou Chevreuil, Louisiana (USA). Bowfin embryos and larvae for developmental and fin bud transcriptomics, ATAC-Seq, and RNA in situ hybridization were collected from nests in Oneida Lake (New York, USA), then raised in the laboratory as described until sampling at the desired developmental stages. Spotted gar embryos were obtained, raised, and fixed as described and approved by the Nicholls State University (#IA053) and the Michigan State University (#10/16-179-00) IACUC.

Note that full information on the approval of the study protocol must also be provided in the manuscript.
