# Supplementary material for: The bowfin genome illuminates the developmental evolution of ray-finned fishes
Source: Nat Genet. 2021 Aug 30;53(9):1373–84. doi: 10.1038/s41588-021-00914-y (PMC8423624; doi:10.1038/s41588-021-00914-y)
Supplement: Supplementary file 5 — Bowfin SCPP gene predictions. [file 41588_2021_914_MOESM5_ESM.pdf]

Supplementary Data 1. Bowfin Scpp gene predictions.

The initiation codon, the termination codon, and the poly(A) signal are shown in red, and the nucleotides at the beginning of exons are highlighted in yellow. The nucleotide sequences for mepe, dmp1, enam, scpplpq20 are incomplete.

```
>mepe: 5′ end & 3′ end missing
GTTAAAACATGAAGTCAGCTGTCCTTTTCCTGTGTTTTCTGAGTGCAGCTTCAGCCCTGATGAAATTTCACCATCCTTACAGATCCCCTGGAGACTA
CATTGATGATAGAGGG
>ibsp
TGACTTCAGATTTTCCTTAACTTTTTCTGTTCATATTATTTTTATTTGTTTTAAGCGCATTTCCAGTCAGTTTCTTGTACAATGAGGATTACTGTGT
TGCTCATTTGTTTCTTGGCGGTAACGCTTGCTTTTCCTACACAAAGAAAACGGGTAACTCGCTCTGATAGCTCCGAGGAGAGAGTGGAAAATGGCGA
GTCATCAGACAAGAATGAGGATAAATCTGATGATAGTGACTCCAGCGATGAAGGAGACAATGGAGGAGAAGGGAAGGGAAAAGACGATGATGGAGAA
GGGGACAATGGAAAGGAGGGGGATGGAGAGGAATGGAATGGGGAGAAGGGCAATGGAGAAGAGGACAATGGGGAGGAGGATGATGAGGAGGAGGACA
ATGGAGAGGAAGGGAATGGAGGAGAGGACAATGGGGAGGAGGACGATGAGGAGGAGGACAATGGAGAGGAAGGGAATGGGGAGGAGGATAATGGGGA
AGaagggaaggggGAGGAGGACAATGGAGAGGAAGAGAATGGAGAAGGTGACAAAGGTGAAGAAGGCAATGGAGAGGAAGGGGTTGGACAAGAAGGA
AATGGAGAGGAAGGGATGGGAGAAGAAGGAAATGGAGAGGAAGGGATGGGAGCAGAGGGAAATGGAGAGGAAGGGATGGGAGCAGAAGGAAATGGAG
AGGAAGGGATGGGAGAAGAAGGGAATGGAGAAGACACAGAAGCAGGAAATGGAAACAGTGAAGGAGAACCTGGTGAGATAAAGAGTAGTCTGAAGGA
AAGGGGAGATGAGGATGGCGAGCCTGAACAAAATGGGGAAGAGGAAGACGTGAACAGCGGTGACCATGGTTATGAGGAAACTACTGGAGTGGATGGG
GAGCAAATAGAAAACGGGGAATCCGGGGCTGATGGAGAGGAGGGAAACGGGGAGGAGGTGGACAAAAGTTTTAGAAGTGTGGATAACGAAGATGGGG
AGAACGGAGAGGACACTGACAGAGAGGGTTCCAAGGGTGGAAGGGGGGAGGATGAAGGAGGAGAAGAGGGAGAAGGAGATGAGGACGAAGGAGGAGA
GGAGGGAGAAGAAGATGAGGATGAAGGAGGAGATGAGGGAGAAGATGATGAGGATGAAGAGGGAGAAGAAGATGAGGATGAAGGAGAAGAAGAGGGA
GAAGAAGATGAGGATGAAGGAGGAGAAGAGGGTGAAGAAGATGAGGATGAAGGAGGAGAAGAGGGAAAAGAAGAGGGTGAAGGAGGAGAAGAGGGCA
ACAGAGAAGAAGGGGGAGAAGACAATAAAGGAGAGGAGGGAGAGGAGGACCACAAAGAAGTAGGAGAGGGCACTGGGGAAGAAGAACAAGAAGGGGA
AAAAGCAGACAAAGAAGAAGGCAATGTAGTAACAGATGCGGAAGACAGTGAAACGAAACGTGCAGACTCTGGCAATGGAAGAGGTTACAGACAGTAT
AACAATGTTGAAGATCACAAGACACCTGGCAAGGAAAGCAACCTTCACAACAAAGGGTCTGAGGGAGAGGGACACGAGACAGAAACTAGAGGCCGCA
GAATTGACAGCAAGCGCAACGCAACAGGAGATTACGACAACAAGAACAATGGGGAACAGGAATATTACAGCAACAGAGCGCACGAGAACGCCATCAC
AGACAATTCTGCATGAGAAGAACACAGCTCCCAGAACATGGTGATTACAAAGAGGTGACGGATTAGCTACACTTTTTGGTAATGAAGATGCTGATGG
CAAAACAATGATTCAGTCAGTATGATTTGGACAGCCTTGAAATAGTGTGAGGAAAATGCTGGTTGTTTTCATCTGGAATCACCATGATGAAAAATAA
AATGCTGCTTGTGATTGGAGAACAAATACCTGATTATCACATGCTTTGCTTCAGCAAAACTAATGCTTGAAACTCCTTTATGTCTCTTGTTCTTTAT
AAAAGTCCCATTCCACCTTGCACTTCATAACACTTACATCAGTAGCTAAATTTGGAAAATGTCTCTCTAAGCAATATTATGAGACCACAATTGCTGA
CAAAGACTGATACAATGTGTTTTCAAAGTATATATACAATGTATTAATTAATATAACGTATATGTCATTGTAGTTATGTGATCTACACATACTAGAA
ACATATTTTTATTGTTAACATTTTGTTTGATATTCTGTTTTTATGTCATGTCACTCCCATTTGTTACAAATAAATAAATAAATCACGATTTAAGTCC
CAG
>scpp1
GTTCAAATTTGGACATTGTCTGACTTCAAGTATTTTACGAAGGAAATTGTGTTCGACCAGAGTGACAGCAAGTCAACACCACCTTGCTTTCTTGATA
GTGTTTGGATCTAAATAGCTGAGGAAGAATAAACCTTTAATGATGATTGCTACAGTATTTGTTGTGTGCCTGGTGACAGCTGTCTTGGCTAACCCTA
TCTTACAACATAATAACATGGAAGGCAATGACCCTGAAGTCGACTCGGCAGCTAACAGCACTTCCATTGAAATGTATATGTCAGGAAACAAGACCTA
CACATACAGTTCATCTGCACAGCAGAGTTCGGATGAAAGTGACACATCAGAAGACAAGAGCTCCATGGAGAATACGTCGGAAGATACGTCGTCTGAA
GAAAGCAGCTCCATGGAGGACACGAGTTCTGAGGAAGATGACACCTCTGAAGACAGGACTTCAGAAGAAGACAGTTCCTTGGAGACATCTGAATCCA
CATCCGACAGCACATCGGAAGACAAGACTTCTGAAGAGGGACACCACAACCCCAGAGACAAGACATCTGAAGAAATGGATGACACTTCAGAAAGCAG
GAGCGACTCGGCTGAAAATGGGAACACCAGAGACGACAGTCGAGACAGAGAGCAGGCTGTCAGACGGCACCGATTTCCAATTTTTAAAATCAACCTG
AAGAGCGATGAAGAAAGTGCAGCCAGTGAACCTGATGAAGACAAAACCAGCCACGAGGACACAGCAGAGGAGAAGGAGGAGGAGGAAGAGGAGGAGG
AGGAGGAAGGGGCCGATGAAAACGAAGGTGAGGAATCCAACAGCACAGAGAGTGATGGAAACGCAAGCAACGACGGGCACAGGAGAGGCAGGAAGTG
CGAGGAGAAAGAGAGTCACGACTGCGAGGAGGACAGCGAGGATTACTCTTTCGAAGACATTGGAGACGACACCCACGAGCTCAATGACAATGTGCTG
CTGTTGGGTGAGGAACAGAACGCACTGTTCGCGTGGTGAATGTCACAACGTTAACCCTTAGGATCGTAAAACCAGTATCTGTTGTACTGACATTATT
CCATGATCAGGGAAAATCACTGGGGTTTTGAAGTCTTTAAACAGCTGTGCCACTCAGGATGTGCGATCAAGCTGACCAGATTTTTTTTGGTCACACT
TCACAATAATGGCAGCAAATACCTACTGAATTATTGTATGAACACCGCAGGAGTGATGCCTGAATATCCAGTGCACTTCCTCTGAGCTCCAATGTGG
ATCATAACAGTTAAGTTTAAAGTGAGGGTCAGGATTAAGGACAGAGTTTCTAAATGAGGGTCTGGTTTAAGACTTAAACATGTGATCAACATCAGAG
CTCAAAGAAAGGGCAAGCAATATTCAAGAGTAAGGCCTGTGGTGTTCATATATTAATTAACTAGGACTTGGCAGCCTTTATTGTAAATTGTTAACAA
AGAAGAATAAAAACAGGAGGAAAGGGTTTCTATACAGTTTGAATTTCTCTTCAACTGTGCAGTCACCCCAATATCCCAAAATCCCAATGTTATTAGA
ACACAATCAATTCTGCTTTCAAATCATCGCATTTGCTTTCTTTAGTAACTCCACTCACATAACATTTTCTTTTGGCACACATCCTGGGCTCAGTGTA
CAAACAACATTGGCATGCTTTCTGAAGTTTGTCCAAACCCTAGTGAATGATTTTATTATCTTATTATTTTCAAGGATGTATTCTTGTTTTTTCTATG
AGTATATTATTTTTGACATCTGTTTGAAGGTATAACCTTTCATGTCAGCACATAACCTCCTCCCCTCAAAATTGACTGAAGTTGTTAATATAGGTTT
GTAACCATTCTGATTGTTTATCTACTGCCAAGACATTGGAGCAGTTAAGTGTATCTTTCCTATTTCAAGTCCAACCTTTTTCTAGTGTCTTTATGTA
TTTATTTATTTTCCTCGATGATAAAAAAGGAAATTAAAATTATATCTATAGAAATCTTTTAAAGCATGAAATGGTATCCAGCCAATTGCATTCATGA
CTGAATCACTTCCCAATGAGGTAAAAGTCACTTGACTGTGTGATTTTACCTGATTAAATACAACTAATAAACACGGGGTCAATATAACTTTTGCATC
TTATTAAATTCTCCATTTTGTATATGAATGGGTAAAACTGGGGGAAAGGATTTTATTTATTTTAATCAGTTCAATCCTCATTTTCATTTTTCTTTAT
GTACCTTTGTCTCCTCTGTCTTTTTATATTCCTAAATATTTATTTTGGTAAATATCACTTTATGCAAAGTTGTTTTTATATTTATTAAAG
>dmp1: a unsequenced gap shown by NNNNNNNNNNNNNN
AAGTATAGCCCCAAAGAAGATCCTAAATCCCTGAAGTCTGAGCAGGAATTCATTACAGTAGCAATCCAGTGAGACAGCAGTATTTCTGCAGTGAAGG
AGATTGAATGACAACATGAAGACTGTGATATTACTTATTTGCCTCCTGGCTCTTGCATCTACTTTACCTACGTATCGTATCAAGCGCACAGCTCTGC
AAAGCAGCTCAGAAGAAGAACAGGAGGTAGACGACTCTTCTGAAGAAAGCGAAGAGAGCCTTGAACAGGATAATTCAGAGGAAGATGCAGTGCATGC
AGCTGCCAATGCAGAAGAGACCGGCAGTGATGTTGCTGTTGAGCTGACGGTGGACAATGGAGGGGATAATGGCAATACTGCTAATGAAGGCCAGGCA
CCGTATGGGACTGGGGCTGACACCAATGGTGATGGGGAAACTGAAAATGGAAAAGACAGTGTGCAGGCTCCTATAGAAAATGGCAGCACACCCACTA
GTGCCAATGGAGGTGCAGAGACAGCAGGAGGAGCCAGCACTGACAATGAAGGAACAGAACAATCCAGTACAGGGGGTGAAGCTGATCCTAGTGGCAT
CACTGGATCGAACGCAAATGGCAACCTTGAAAAGGCAGACCTTGCAGATCTTACAAGTAGCAGTGCTGTCCAATCAAATGGTGACCCAATAGGCAAT
GGAGACAACACTGGAGAAAGTGGACAAGCTGGCAAAGCTCCTGAGGGTATCCATGTAGATGATCAGCAAAATAACATAAATCATGTGGTGGAAGCTG
```

CTGTGGAAAGTGACGCTACAGATCTGGGCAACTCTGTACTCGTTGGAGACTATGAGGTCAGTGGGACAGAGAACAGAGACGAAGATGAATTGGGGAG
CACTGAAAACAATGAAGTCGCAGGAGACGACTATGAATTTGATGATGCAGGAATGCAAGGTGATGATCCAATGGTTTCCCGTGGAGATGAGAGTGGG
AAtacacaaaatgATCAGTCTAGTTCTAGCTCTAGTAGTGAGGAAGGTAAAAGCAGTGATTCCAGTTCTAGCTCTAGCAGTGAAGAAGGTAAAAGCA
GTGACTCCAGTTCTAGTNNNNNNNNNNNNNNNTAGCTCCAGTTCTAGCAGTGAGGAAGGTAAATGTAGTGACTCCAGTTCTAGCTCTAGTAACGAGGA
AGGTAAAAGCAGTGACTCCAGTTCTAGCAGTGAAGAAGGTAAAAGCAGTGACTCCAGTTCTAGCTCTAGCAGTGAAGAAGGTAAAAGCAGTAATTCG
AGTTCTAGCTCTAGCAGTGAAGAAGGTAAAAATGGTGACTCCAGTTCTAGCTCTAGTAGTGAGGAAGGTAAAGGCAGTGACTCCAGTTCTAGCAGTG
AAGGTAAAGGCAGTGACTCCAGTTCTAGCTCTAGTAGCGAGGAAGGTAAAGGCAGTGACTCCAGTTCTAGCAGTGAAGAAGGCAAAAGCAGTGACTC
CAGTTCTAGCAGTGAAGAAGGTAAAAACGGTGACTCCAGTTCTAGCTCTAGTAGCGAGGAAGGTAAAAGCAGTGACTCCAGTTCTAGCAGTGAAGAA
GGTAAAAGCAGTGACTCCAGTTCTAGCTCTAGCAGTGAAGAAGGTAAAAGCAGTGACTCCAGTTCTAGCAGTGAAGAAGGTAAAGGCAGTGACTCCA
GTTCTAGCTCTAGCAGTGAAGAAGGTAAAAGCAGTGACTCCAGTTCTAGCAGTGAAGAAGGTAAAGGCAGTGACTCCAGTTCTAGCTCTAGCAGTGA
AGAAGGTAAAAGCAGTGACTCCAGTTCTAGCAGTGAAGAAGGTAAAGGCAGTGACTCCAGTTCTAGCTCTAGCAGTGAAGAAGGTAAAAGCAGTGAC
TCCAGTTCTAGCAGTAAAGAAGGTAAAGGCAGTGACTCCAGTTCTAGCTCTAGCAGTGAAGAAGGTAAAAGCAATAATTCCAGTTCTAGTTCTAGCA
GTGAAGAAAGCAAAGGCAGTGATTCCAGTTCTAGCTCTAGTAGTGAGGAAGGTAAAAGCAGTGACTCCAGTTCTAGCTCTAGCAGTGAAGAAGGTAA
AAGCAGTGACTCCAGTTCTAGCAGTGAAGAAGGTAAAAGCAGTGACTCCAGTTCTGAAGAAGATAAAAGCAGTGACTCCCATTCCAACAATGAAGAT
GGTGATGAGAACACTGAACCTAATGCCAGTTACACAATTATTGAACACAATATCTCAAGCTCTCGAAAAGAGCCACTGAAACATGTTTTCCAAGACA
GCAGTAGTGAAAGTGATAACAGCAATGATAGTGCTGATACGAGTGAGTAAACAAAGTGAACTCAAAACAGCGTTTAACTTTTTCTTGGTTTTAGATA
ATTTATTTTTTCCAGTTATCAGTATTATCAACATCCGTGTACTTTTACAAAAAACTTACAAGATGGAAGGAGTGCTTACATAAAAGAGGAGTTTTGA
ATGCTCAGAAATATTGTGAGGAAACAGTGAAACTTGAATAGATGCGTTTGCATAGTATATATGCTTCAAAGCAAAAATAATATACAGGAGCTGCTTT
CCTACCAGGATTTTGTTTTAAATAGATTCATTTAATTTTCTAGATGACACACATAGATCTATATAAAAACCTGTGGTCCTTGTCAACATCAAAATAA
AGTCAAGTGATGATACGCAAATAAATAAA
>scpp9
GAACATTCTCACTTTCTTTCCACAGGTCATTGGTGAATTAAGAGAAGTATACAAAATGAAGATTATTTGTTTATTTTTTTTCATCTGCCAAACCATC
ATATGTCTGGTGGATGCCGGGCCACTGTCTGCAAGTGTAGAGCTTCTGGGGTTAAATGGTGCTGTTGTGGCAAATGGAGTAAACCCAGATCTCCTGA
ATGGAGCCTTGCTTGGGCAGCAGCAAATAGCACAGCTTCTGGGGTTAAATGGTGCTGTTGTGGCAAATGGAGTAAACCCAGATCTCCTGAATGGAGC
CTTGCTTGGGCAGCAGCAAATAGCACAGATGGTGTCAGGGCTCCAGCCCTTCGTTCTACAGCAGCCAGGCATTGGTCAGGTGGGTCTACAGCAGGCT
CAATTACTTGCTGTAGGACAGCAGCAAGCTGGCACCCCTCAGCTACAAGGACAACTGATACAGCTGCCCAATGGGGGTTTTGTGTTTTTATTACCAC
AAGGGAATGGGGCTGTAGCAGCGGGGGCAGCAGGAGCTGGACAGCAATTACAAATACTGACCCCTGGGCAGCAGGCACAGATCCTTCCAGTTGTGGG
AGGAAACTGCATCCAGCAACCAGCGGCTGCGATTGAATTAGCAGCAGCTGCACAGCCTGTGGGTAATGGAGCCGTCAGAGTCAAGCATTCAGCACCT
GGAAACCCAATGTCAAGCTTAGAAGCAGAAGAAACTGCACCACTTACAAATGAACGCCCCACAGTGTCACTTGCAGAAGCCCTAAGGATTCCTTTAT
TTTCCTCCAAAATGGATCTCGAAGTTGAAGAGGGCTCAGACGATGACCTTGCTGTTACTGGGTCAATGGATCCTGCTGCTGCTGCTACTGCTACCCC
GTTGCCCCTGTATTCACAAACAACTGACCCACCTACAAACTCAAAGATACTCCAGGAGCTGCCATGATGTTCATCTGAACTTAAGGAGCAGTGGAGT
TAACAGTGTATCTCAGTATACATCAATGTTCCACTAATCAGTAGATATAGATATAAAACTGTATTGGTTATTAAGAACACCTAAGATAGATATGATA
TGAAAACAACTTGTTTAAAATTGCCTAGTGTAATTTTACCATGTATTAAACTGCTAAATAAAGAATCCTAACCTAACAAGTTA
>scpp9l2
CAAAACATTAAACATCTTCTCTTGCAATCAGTGAGTGAAACAACGGATACGTCTCAAAAAGAGTTTTTCTAAGCAGGATTCAACCTGAAAAATGAAG
CTTGCACTGTTTTTAGTCTTCTTCTGTGGCTCAGCAGCCATCTCTCATGTTGCAGCAGCGAAAATAACCCTGCTTACACCCCTGGGAAATGGATTGA
ATCCAGGCCTATTGAATGGGGCAGGTTTAGCTGGACAGCCACAGCTTTTACAGAAAGTAAAATTGGTCGCAGGCCTTAATCCAGGGCTGCTTGGAGG
TGGACTGAATCCAGCGCTGCTTGGAGGTGGACTGAATCCAGCGCTGCTTGGAGGCGGACTTAATCCAGATTTATTGAATGGAGCAGGTTTAATTGCA
CAACCTCAGATCCTGCAGAAAGTAAAATTGGTCGCAGGCCTAAATCCAGGGCTGCTTGGAGGTGGACTGAATCCAGGGCTGCTTGGAGGTGGACTGA
ATCAAGGGCAGTTTGGAGGTGGACTGAATCCAGTCTATTTGAATGGAGCCGGTTTAATTGCACAACCTCAGATCCTACAGGGACTGCCTTACATTAT
TTTGCCACCTCAAGCAAATGCTGCAGCTGcccagccatggGCAAACAATCTCCCACAGCCAGCTATGGCAACTCAGGCCAATACTGGAAATGTCAAC
CAGCCACAAGTGGCAGCTACACAGACCCCCCAAATTCCTCTCACACGGTATAAGCGCTTCATCCCTGGAATGGGCCCTCAAGTCTGGAGTGGTGCAG
CAAATCAGATGCAACCAAGTGCACCGCAGCCCACCGCCACTGCAGGACAGCGAGGGGACTACCCAAGTACACCGGCTTCTAATGCAGTCAATCAACC
AAATCAAATCTAATTCATGGAAGGCTCCATAGATTAATCTAATTCTGCTTAGTTTGCAAATTAATTCTACTTTGGGCTGGTCCAACATGCCAAGATT
CGCACTGGTCTTTGAGTATCCAGTGGTATTTTAGGTGGGTAGTCCTATACCAGTGACAGTAGCGGTCTGTGAAATCAGCTGTTTATGTGCTATTGCA
TTCTACCTTTCTTTAATGTCACATTATATCACTGTGGTATTCATTGTATTCTCTGTTTTGGATGAGCTTGAACATTAGTATGTAGTTGCTACCCTAT
AATAAGGGCTATAACCATTACATTGAAGTGGTACGATTATATTCAGGATTTTCTCAAGCAACAGGCAATTACATCATGAACGTTTATGTTTTGCTTT
GTTTTGTTTTCTGTAAAAGTTGTTGCTAACAAATAAAATCTATGGAAAACTGAAAA
>scpp9l1
TTCTTGTCTAGCAATCGTGTATGACACACCTGATACATCCAACTGCATCGAAAAAAAGTCAAGATTCAGACTGAAGAAATGAAGCTTGCACTGTTTT
TAGTTTTCTTCTGTGGCTCAGCGGCCATCTCTCATGCTGGAGCAGGGAAAATAACCCTGCTCGCACCCCTGGGAAATGGACTGAATCCAGGCTTATT
GAATGGCGCAGGTTTAGCTGGACAGCCACAGGTTGTACAGTTGGCTCCTGGAGGCCTAAGGTTCTTACTGCCACAGCCAGGTGTCCCGGCTCAGCAG
GGTGTGGCAGCTCAGCAGGGCGTGGCAGTCTCCCCTGAAGTCGCGCAACTGCTGCAACAGCTGGTGGGAGCTCTGGCCAACGGGGGAAACGTCAATC
AGCAGCAAGTGGGAGCTCCACAGCCTGGCTTAGTTCCTATCATTCGAATCAAGCGCTCAGTCCCAGAAATGGCCAATGAAGACTTGACTGGAGCAGC
AACTCAGATTCCCCCAAATACCCCAGAGCCCAATGTTGCTTCAGACAAGCTTGCAGATCTGCCGAAGACTGATCAAACAGTCTAACTAAAAGAAGAG
TCCAAGGTAAAACATATGGCAGTCCAATATTTCTGGCAGACTGGAAAACTCCAGGAAACGTATTTAATCATTTATGAACCTTTAGATGCTACAGTAA
CTGCCTGAACTAAAACAAACATAAAAAAGCTATGAAGAAAAAGAAATACAGTAATTATTCTGTTAAAATATTAAGCTTGGCAAACTGAAAATGTGCT
GTATTTTAATGTACACCTGCTACTCTCAATATCAATAAACATTAATGTATTGT
>scpplpq3
ATTCAGGAAGACCTTCAGGTTCCAGCACCAACATTCACTGAAGCAACTCCAATAACAACTACAATGATGAACACTGTCGTTCTGCTAGCCTGTTTAA
CTGGAACAAATCTTGCTTTACCTCTCTTAGGATTGACTGCAAGTAACAGCTTGGAGATTTTGGGATTTGGTGGTCTTGGCCTCGGTATGGCGCAATC
TCAGGTCCTACCCTTTGGTCAACAGCTTGTGTCCCAGCTGTCTCAGATtggcagcccaaGACAAGTGTGGCTCCCTTCTCAGGGGGCTTTACCTGCC
CAGCAGCCCCAGAACCCTCAGATGCTGTTTCCTGCACAGGGCCCAGTCAACCCTTCTCTATTACTTCCTGCACAGGGCCCCATCAACCCTAATCTAT
TATTCCCGGCCCAGGGGTCAGGGCAGCCCATGATATTCTTCCAGTCACAGATGCCTCCCTCCGTATTTTACCCCCAAGGAAACCTACCCTCTCAGGT
CGTGCTGTCTCCCCCAGTGATACTCTCCTCCCAGACCAACCCTGGGCAGATGCCTCGGGATCAGGGACCTCAGGCTCCAATCCAGCCCCAGGCTCCA
GTGGACCCCCAGGGGCCTCCAAACACACAGCAGCCTCAGGTCCACAACCAGTTTCTTCCATTCAACCCCATCTTCCAACAACAGGCACAGCAGGACT
ATCCATACTACTACTATCAGTACCTCCAACAGCAGAATCAACCACCCAACCCGACACAGACCCCCGACCTGGGCACTGCAGAGTCAGCAGCCACTCA
CGCGCTGCCTGAACCCCTGCAGAAAACTCCCCTGGAACAGACAGAAGTTGCCGATGTTATCAGCCTCATGGAGCCATAACATTTATTTTTGAAGGAG
AGAACTACAAGGCAAAACGTGTTGAATGCTGAGAGGACAAGAAAACATCAACTTGCTTGGCATGAATGCTCTCCATGCAAAGAAGCTGCAGCTCCAA
GATCTATTACTAATTGTTTCATTATACATCTATGTCTGCTGCTATATTTGTAATTATTGTAATTGCTTAACAGCTGCAGTTCGCTGCAATTTATATC

AAAGCAGCCCTTTGTGAAGCTTCAGCATCTTAAAAACCTTTTATAGAAAGGTGTTCTTTTGAATATGTATGTATGTATGTTCTGTCTTGGTATTGAC
ATTGTATTCACAGCAGAGTGAAATCAATAAAGTTTAATTGTAGTAGCAGTAAA
>odam
GCTTTTGCGAAGATTTGAGTCTGGAACCTACGTACAAAGGGCGTGGGGAAATGCAGACTGTTGGAGTTTTCGTGTGCCTTGTGAGCGCATGTCTGGC
CATACCTGTATACCGTCAGCAAGTTGGGTTATCGACAAGTTACAGTCATGAGTTTCTGCATTTTAATGGGATGACATACAGAGGAGCTGGAATTGGT
CCAGGGCAGGCAGGACCATTCATCCCTCCCTTCGTGTTCCCGCAGCAGCCTGATGTAGGATTGCCATCCCAGCTGCCTCTTAACCCCTCAGTCACAG
TTCAGGAACAGGGCCCTCTGCCCCCTCATGTTCTCCTGCCTACTCATGTGTTTGTACCCTCCCAGGGCAACCTGCCCCCTCAGGTGCAATTCCCTCC
CAGCCAGCAGGACTTGATGCCCCTCAACCCCCAACTGCCCTCCGGTCCACAGCTGCCCCTCCCTCCACAGGACCCCAACGTGCCCCAGCAGCCCCAG
AATCCCAGCCAGATGGGCCCACAGTACTTTCCTTCAGTGGCATTCCCACAGCAGCCAGGACAGGGAATACCTTACTACTTCACATACGGTTATGCAC
AGCAAATGCCCCCATCAGTGATCCAGCCAACCCAAAGCACAGCTCAGCAGAACCTGGCACAGGCAACACCATCGCCCCAGCTGCCACCACAGGAGAC
GCTAGGCCAGGGGACACCTGCACCAGACACAGGGCTCGCAACAGCTGCTCCTGCACAAAACAGAGGGGATACCAAGACCGGCAACGAGGAGGGTCTT
CCAGGCTTTTCATTTCTCTTTGAGCCATAGGATCCAAACTGATCTGATCCAAAAGTCAGAAAACGACTCCTGGGATTAAATGGTCGATTAAATCACT
GATTCTTGAAAAAAGGCCTTGAAGACATGACAGCGTTGAGAGGCTACCCCACTGTCACATACTTTTGTTATGGATGTTATTTTGCATCCACATTTTT
TTTTTCACTTAAAATATTACATTAACTTTAGTGTCTTCTCCATGTGTAACATTTTTAAATGTTAAATACATTTTTTAAACAAAA
>scpp3aa
TATTGCCACATTACACTTGTGTGTATTATTTCAAAATCATTTATCTTCCTTAATTAGGGGGGTTTTCTGCCTGAAAGGTATTTAAATTGGACCTGGT
CAGTTTCATTCTGCAGGAGCTACTTGTGTGCTGGATCTACCTTCAGCAGTAAACAGACATCTTTAAACCATGAAGACAGTTGTGTTTTTGGTTTGCT
TCATCGCACTCAATGCTGCTATTCCAGATGAAGAGGATTTTCCTCTCCGCCTCGATCGTTCCATCTCCTCCAGTGAAGAGGGAATGCAGGCGTTCGA
GATTCTCACGTATTTAATTCGACTGTTGGCAGCAGGAACACGAACCCCAGCAACAACAACCGCAGCCACAACAACTGTGCCCACAACAACTGTGCCC
ACAACAACTGTGCCCACAACAACTGTGCCCACAACAAATGGAACGCTTTAACCAGCTGCATCGGTCTCACTAACTGTCTGCAATGCATCCTTTATGG
ACTTAAATAATTAATCTTATCAATAAATATGACAATTTACAA
>scpp3ba: borders between exons 3 and 4 not determined
CTTTAAATAGGAGCCCTTCAGAAAGCGTTTCATCCAGCCTGACACAGGGTATCATTCCACTCAGACACTTTCTCCTCCAGTGTTGAGAAGCCTCCAA
GCTAAGCATGAAGACTGTGTTTCTTGTCATGTGTCTCATAGCACTGAGCTTTGCTTTCCCGATTGAAGATGAACTCGAACGCATAGCACGGTCAGCT
GAACAAGCTGCTCCGAGTCCCCTTTTCGCTGGATTTGACGTCAACAAGCTTATTGAGATTATTAAAGCACTGCTGCCTCTGCTCACAATGGGATGAT
AAACTGGAAACTCTTGTGTCTTCCAGATTCCCAGCATGAGCTCAAAACTGCTCCTTTAGGCTACAGCAGCTTCCACTAACCCCGGCCTGCTTCCTGT
TCACAGCTGTACATCTCAATCTGATCTTTAACCAAAGCTTAAAAGCATTTAACACACAATCATGTTTGTTCTTTTCTGTGTATATTGTAACATTGTA
CACTAATTGTTTGTATGTGCATGTATATAACATGTCAAATAAACATGTCAAAG
>scpp3ca
CTCACACAAAACAACAATATTTACATTATTTAGTTAATGCGATACGATTGGCCATTGGCTTTCTGAAATTCCTTTAAATAGGACCATGTTAGAATAT
GTTTAAACCAGCCTGACACAGGGAATCATTCGACTCGAACTATTTCTCCTCCTGCATTGAACACCGTCCAAGACAATCATGAAGACTGTGTTTCTCG
TCCTGTGCCTCATAGCACTGAGCATTGCTCTCCCGGTTGATGAAGAACTGGAGCGTGTGGCACGGTCCTCTGAAAAGACGGCTGCAACCTCTCCTGT
GGCTGGATTGGACATCCAGATGGTTTTGAATCTTATTAAACAACTTATGCCAGTACTAGGATTGGGAAAATAAAATGGAAAAACATGTGACTTCAGG
ATTTCTAACACAATCTCCAAACCTGCTCAATCTCCTGCAGCATGTTCCCCACACCCACAGAACATCTTTTCATCTTTCTCTAGGCTGTATGATTTAA
TCTCAGCTTTAAACACAACATGTACTATTTTAAAGGGCTACATTTTAAATTAAATCTTTGTTCTCTTTCCTGTTTATGTTACAACACAGTAGACTGA
CTGCTTTCAATGTAGACATCTAATAAACTTGTTAAAG
>scpplpq4-like
CTCGCCGCAGAAATGCATGTGAAGTTGCAGCTGTTATCTCTATAACCTATAAAACAAAAAGCTGTGCTGGTCTTTAGAGTAGAACTCATAGGCTAAT
GTTCTCTGTCACTCAGATTTTACACTGTGTTGTTTTTTAGGTGTTGCAGATTATTCAGCAGATGCACAATGAGTGCTGCCTTGACTGTATTATTTCT
TTCCGCGATTTTTCCTGGAGCTAATGGGCAGCAGCAGCAGCAGTTCAGAAAGTGCTGAACCACAACTTTTTCCTCAAGCTGTTAAATTTTCCCTTTTGG
CAATTTTTGCACATACCTCCTCCCCCAACAGCGCCTTCTCCTCCTCAGTCACCACTCCTACAACTCCCACTACCCCCCCAAGAGGAGATGGACCCT
GACGTCACAGAAGCGGATTTGGAGATTTGCTCTGGACTGCAGATTCAAATTTACTGCAATACATTCGCAATGAAATCCCCAATATTGCCCTCCCGTT
ACAATACAATAAGTAGACGTTTGTTCATTTGCCTGCATTAGTATAGCTTCCTTATTGTTTTTTGTTCGCACCTGCACTCAACCATTACCAGTGTACC
TTCGTTTTATCAACTTGTATTTACTTAAATATATCTACCGCTTATTTGTATAACCATATATAATATAATATAACCATAACTGGTAGTAGATTAATAT
ACTTTGTATGGCACTTTTTAAATAAAATAAGTAGCCCATCAAACTACCGTGAAATAAA
>ambn
TCTCAATTCCTGTGTCCTAAAGCAAGTATTTTGAAGTCGTATTGGAGAATAAGGTTTCCAAAATGAAAGCCGTAATTATACTAATGTGCCTCATTGG
ATTGACACTTAGCATGCCTCTTTCTGCGTATCGGCAGTTGCAGCAACAGATGTTACAGCAGATTTATCATCAACAACAGCCGCAGCAAACAGGGACA
CCGAGGATGTTAAGTCTGAGCCAAGAGGCACTACAAGAAATGGCACTTTACAGAAGATTAACACAACAGCAATCAGCGGTGTTTCCTCAAATGCACG
GTCCCATGGGTGTGCAGCCACAGCTACCTCAGACTCCCATAGGCCAGACCCCGTTGCAGGGACCTCCTCAGCAGGGACCTCCTCAGGGTCAGATGCC
CTCTCAGTCTTCTCCACAGGAGCTTCCCATGTTCAAGCTTGTAGACTTCCAGCCTGGCTTCCAGCAGCAGCAGCAGACATACCAGGGGCAACAGTAT
GATTATGAAAATCCCTTCCTGTTGCATCATGTTCCTGCCCAGCAGACACCAGTTGTTGCCAATGCTGGGGGTGTTCCAGCCATGCCCCAGGCCACAG
CTGCCCCTGGGCAGCAGGGGGAGCAAGCTGGGCAGCAGAGACAAATCGTCCTGGCCCCCCAACCGGGAATTCAGGTCCTGCAGCCCCACCCCCAGGG
GCCACAGCCAGGCACTCAGGGACAGCAGCCAACACTGTCATCACAACTGCCCCCGATCTTCCATACTTTTGGAATTGTCCCTATCATGCCCCAGCAA
GCCCAGGAAGGGCAGCAGTTCCCTGGGTATGGCATGTTCTTCAACGCTCAGTGCTCCCCGCAGCCGGCGCCGCCTGCTCTGCCAGGGCAGCCTCAAC
AGCAAGAGATCCTTTACCTGCAGATTCAACCCGCACAGACAGCTATGGGGGGTGTTGGACTGGGAATTGTAAGCTCTGAAGAATTACAGGCAGCTGG
GGGCATGGCTGGCTTTGGTGGATATATCCCTATACTTGGTGGAGCAGGGTATGCAGGCTTTGCAGGAGGCGTGGCAGGTACAGAGGGGATCATACCC
GGCGGTCCCCAGACAGGAGTGCAGACTGCCGGAGTCATACCTATTGGCCAGGGGGGCAGTCCTGTTGTTCCAGACGTTCTCCCCTCAGGCCAGGGGA
CCCTGACACCCACTCTGCGGAGCACCATATCAGTCCAGGGAAACCAGCCTGCCCCTCAATCCAGAAACCCCAACACTGGGACCACACCAGCTGGGGC
CCGCAGTGTGACAGCTCAGAACCCACCTGCAGGGTCTGAAAATCCCACACAAAGGCCTCCCGTGAACAACGCTGCCACCCAGAGAGCAGTTGTCAAA
AATAAACAAGTGCCTGAAATGGATGACTACAAAGCATCCAACCTATTTCCTTGAGAATGTGACCCAATTGCGCCAAGTGCTACCCATGAAGTTTCAC
ATATCACATGACTAGTCGCCTATATCTGTGTCCAAAATGTATGTTTTTATATATTTTGAAATAACATATTTAACTACCACTGTATCCTTAAGTTGTT
TTTACATTTATGTTAACCTGTGGTATTTTACAGTATGTATTTTTTATGCATGGGATATAACATATTTAGAAAACATACATATATATATAtatatata
taTATATATATATTGAAATAAAGTATTGCTATG
>scpp5
ATTTCCTGAAGCGTCCTCAAAGGAATATAGTACAAAAATGAAGTCTGCTCTCTTGTTCATGTGCTTATGTGGCACCACATTTGCAGCCCCGGCTTTA
TATCAATACCTCCCCCATGTAGAAACACCATGGCAGCCTGTTGAACAGCAGGGACAACGTCAACAGATCAATCCTTATGCTCCACCACTACAGATTC
CCCAACAGCAAGGCCCAGCCAAAGCGGCCAGCTATGAGATTATATTTCCTTTAGGAATGACTCCGCAATCAGGAGCAACTCAAGCATACATCAAACA
GAAGATCCCTCAGGGCCCTGGAAGACAGAGTTTGGAAATTATGTACCCAATTACTCTTGGACAGCAGCAACAGATGTACCCGTATGGAAATGCACCA
ACACAAACTGCGTCTCAAACAGTGCATCACCAACAGCCAATTGTGTACCCTACCTTTGGAAACATGCCACCTCAGTCAAACAACAATATGTTCCCAT
ATCTTGGAATGGCACCAGCCCCGCAGAACCCTTTTGTAAGGCCACAGTCAGTCCCTTACCTTGTCCCCCAGCCTGTCCCTCAGGAACCAATCCAACC

AGCCCAACCTGTCCAACCCATCCAACCTATCCAGCCCGTCCAACCTATCCAGCCCGTCCAACCCATCCAACCCATCCAACCCATCCAACCTGTCCAA
TCAGTTCAGTCGGTGAACCCTGCCTTCCCAAGTCAGAACCCCCCTTTGGAAAAGAGGGATTGACTTGATGCTGTCATTTTGAATTGAAGCTGGTCTT
CGAACTGAGGGGGCATGTGAAAAGTCACTGTTCCCCAATGTGTCCTTTTGCTCTTAATTTTCATTCAGGTAGATGGAGAAGAATATTCTTAACGTGT
AGCAATGCATGCTGGATCCGCTCTTCAGAATTGAGCTCAATATATAAAAGAAGATGGGACCGATCCTTTAGAAGTGGGATGACAGCAGGAAAATAGT
CTTTGTAGAGCTAGCTGATTAACGTGTCCTCTCTAATGCTGATGTGATGCTTCTCTTTGGTGTTATGTATGTCAGCCATGGTATTAAGTTTCTTTTG
TTTTGTCAATGCTGTGACAGACTATTTCATGAAGTATATCAGATGTCAGATGTGAAAATGAAACTGTGCAGCTGTTTTAAACAAACAAAAGAACAAA
ATCCTGTACTGCTGTTTGAAATAAAATATGCTAATCTTAGAAATAAAGTTTTAAACCATCACAAAAA
>enam: 5’ end missing, some exons missing (NNNNNNNNNNNN)
TTCAGACCATCTTATGGCTCCATCCCTGAGACCAGACACCAGCCACCACGTGAAGAGTTTGGACGCTATGGAAATATCCCAGAGACGACACCGCAGC
AGCCTCACCCTGAGCTCAATCCTTCATTCGCTGAACCGCAGCCAAGACCACTCCAGCCAGAGAAACCCACTCAAGATNNNNNNNNNNNNGTCCCTGC
CTTTGCACTTGATCAGCTGCAAAGACAACAGAATTATCCTAGACTTGTGTATGTGCATCTTCCAGCAATGAGTGAGCCACATCATGGCGGTTATATG
CCTTTCGGGGGGTACGGGGGCTTAGGTCGACAAGGGTCCCTGTCCGAAGAAATAAATGAGATAGAGGCAGAACCACCAGCTAAGCCCAAAGCCGTGG
CGACACAGACCCCCAGCACGGAAGGCGCGGCCCCCAACTTTGGAGGAACAGCCACCAAGACGGAGGGAGCAAACACCAACACCATTAGTGCCCCGGT
CAATGGAGTGGGCACGGGTTTGGGCGTGGATGGATCCCCAAGTGGTCAGGAAGGCTCCCCTACCAGCCCTGAAGGAGCTCCCACAGGGGCGGAAGGA
AAGACCCCAGCTGTAGGCCACGATCCCACAGGTAGAGGAGACACAGTGCCCAGCCCAGAGGTCAGTTCCCCCAGAGAGATTGGCCATTCCCCAGCTC
AGGGACCCCATCCAGTTTCCCCCAACAACATGGATCCCTTCACAGGGGGGGTGTGGCCGGCCGGCATACGAAAGGTCACCCCTTCTCCTGGAGCGAG
CACCACCCAGGGCTTCGGGGGAGCAGCAGGTGTGGGAGACTCCGCCAGCACGGTCAACTCCCAGCCCCTGCCGGACAACCAGGTCCTCCAAACACCC
TTCTCCCCTTCAGACGGGGTGAACGACAACAACCCTTCCGCAGCACAGGATACTCCCTGTGACCAGCCGGGGAATAGTGGCCAGCTGGAGAGTGCTG
CTCCTTCCGCAGACAGCACGGCTATGCCAGGCTACGGAGACAGGATTGTGTTAACCACTGCCGGTGCCATACATGCCGGAGAAAATCGCGAGCCCTG
CGCTACACACGACCGCATGCCTCACGCAGAGGAGAGTCTTCCTTACTACTGATGAAGAAGGGGGAGAGCTTGCTGTGTTTTTAAGATAAAACCCAAT
ACATTTATATTCCCCCATGAAGATTCATGAAGTTAGTCTGTAATTGATACCAATTAGATATGATATCCATACAATGAGATTAACTGTTGAGCAAGTT
TGTATTTTTACATTTTTCACTTTTGTAATGTTTTCTTTATTGTCGTTGTTGTTGTTGTTGTTCTTTTCTACCATACCTGGACAGAATATGTTTCTTG
CATCACCGCTTTTATTTTTTTAATGATGTCAGACACAGCAATTATTATTATAATAATAATATTCTATTTGATTATATTTTTGTCCCAAATGCATTGT
AGTTGTTCCGATTTAATAAATATGTAAAACAAAAAAATACGTCTATTGTTTTTTTCTTTCTTTCTTTCCATGCAAACCACACAAATATTTAATAATT
TTGCCTGTGTGTGATGTGATATGTGATCCATGTTTTTATGACGTTATTTATGATCATTCTGGGATAGTAAGTAGCAAATCATTGCCACTGATTAGGA
AGATATGAGGCATAGCCAGGTTCTTGAAGATACAGGCATTTTCACACATGTGTAATTTATATTTAGAGAGTAGTGTTACCTCTGTATAAGTAAATTT
CTTAATAATTAAGCTTGTTTTTTTCTTAATAAAAGATTAATAAA
>scpplpq8
CAGACAGTGAGGACACGGACAAGAGCCTGCGGGGCCGAACACACTATCTTCTCACGTTATTTCACTGGGACTTTGCACACAAGTAACTGTGCTCTGC
ATCCAGGGGGATTTTAATTCAAACACAAAATGAAGAAACTTATTTTCTTCACCTGCCTTTTGACGGTTACCTTTTCTCATCCAGTCTTAAACAAGTG
GCAACACAACAGACTTGTCAAGAGAGACCATTCTGCCAGTCATGAGGTGGAATATCTTTACCAGCTTTATCTCAGCAGAATACACAGCCAACCACAA
GGATTACGCCCTCAGCCTATTTACAGATACATACCACAACAGATCGGGCAGGCAGTGAAAATCTATTGTGGATATATACAACTACACCCACGAGTGA
TTATCCCAGTTCCCACTGTTGACCCTTCAATACCCATGGTGACCCCCAGACTTCCAGCCCCAATAGCAACCGCAAGGCCAATGCAAAACACAACACC
ATTACCACCCCCTGGTCTCAACACAAGACCTGCAGCCACCACTGCCAGCATAAGAGGAGACATCTGAGCACATCTGGATCAGAATGGCATTTATTAA
CTATTTGTTTAAAAATATCTCTTCCAATTTTGTTGGCTATGAAAATTTTTTAAAGGTTTTTTGAAAACAAAGGGAAATGTATATTTATTTTCTATGA
CATTTATAACTGTGTTATTTATTTGTATAGTGCATTCATTACATGTTTGTTAAATTCATTCAAACGCTGTACAATGTCTACTGTCTATGGTATATAG
AAATATTAGTACAACAAAATAAAAATATTTTTTGTCCTTGCAGTTCATTCAAGATCTTAATGAAGATGTGAATTTGATGAAAATGTGTTTGGCAACT
GCAAATTAAATTCTATGGTGTG
>spp1
GAGAAATTGAACATTTTCTGGCACACACAAAAAGTTTTACAAAGTTTTGTTGTTTTGTTCAGTGACTGTGTAACCTTTCAAGTTCTGAAACAAGTGA
CTGCATTTACCTTCCAAGAAGAAGTTGAGGTGAAATTCTGGAAAATGAAAACTTCAGTGGTATTACTTCTTCTCCTGGGCACAGCCTATTGTCTGCC
TATGCGTCGCTCCTCTGGTAAACTGAAAGTGGACAGCTCTGAAGAAGAGCAGGGCTTCAACAGAAACCGAGGCCAAAAACAACTGAAGGAACAACCA
GCACTACAGGCAGTGCCTCCACAGGGTCTTGCAGTTGAATCAGATGAGAGTTCTGATAGCTCTAAGGAGCCAGAGGCAGCATCTAATGAGAGCACAG
ACAGCACTGACAGTATTGACAGCTCCATCACTGAGGAGGATAATGACACAGAAGAAGCAGATGACAGTAATGAGTCTGACTCAACAGAGGCCACAAC
TGCTGAAATTGTAACACCAGAGCCTCCAACTGAGCTGACCATTCCACCAATCATCGACACAGGCAGGGGCGACAGCCTTGGATACTATGAAGATTCC
TACAAGTCTATGAAGTTCCTCAAGAGTAACAAAATAGAACCCACTCTTCACAAGATCTATGTGTCTAAAGTGGAAGAAAGCCTGAATCTGGTCAGTA
AGAATCTGGCAGGGAAGAAACAAGTGAAGTCTCCTTCCAACAGCAAGGATGAAAATGCCATTGACAAGGACCTGAAAGTATTTAAGGCCGTACAAAC
AAGTGATGATTTGGACGAGGACACCAGCACTCCAGAGGTCGAAAGCCAAGGCCTGGACTCTGCCAATGCAGAAGCACCCCAAGTCCGCCAAGCGGTC
ATTCCACAAAGCAGCGATCAGAGCACAGACAGCACCGCCAGCCAGGGAGATGAGAGCGCCGAGGGGACCGACAGCACCAGCGACGAGACCGACAGCA
GCCAGCAAAGCAGTGAGGAGTCGACAGCATCTCCGGCGGACGACAGCAACAGCGATCAGACCTCCGAGAGCTCAGAGAGTGCTTCTGTGGAGGCCAC
CACCAACGCAGACAATAACCCTGTGCTGGTCAAATAATATTCTAACACATCGACATTGATGGAAGCCAATGTTTAACACGGTGCACCTTTGTGAAAT
GCTCCCTTACATTTCATCATTGTAGCAGGTTACTATGAAAAAGGCCCCACTGGCCCATTTACTGCAATCCTCATTACAAATCAGTCCTGTACCGGGC
ATACAGTAGTAATGGATATTTTGTTTGTTGCACAATAAATAACGAAATGTTGCTATTTTTTACAATTTTGTATTGCATTTTGTATCATGATTTCTAA
TTAAATAAAAATGTGTTTTTTTTTTCTTAAAA
>scpplpq16
GGCTAGAAGCAATTGACCTGCGCTTATTATCAACAGACCTACAGAGCTTCTAAGTACAGTAGAAATCAACTCCCAGAAGACTTGCTAAAAGTTTGCA
TAAAAAAAAGATATCTTTTCCAACATGAAACTTTTGGTGTTTGCTTTACTTGTTTGTGTTGCAGTGTGCAGTGTTCCTGCATTTGCAAACAGGCCAC
CAGAACGACATGGTCCCAGAAGAGAACCATTCCCTAACCATCCTAAATTCCCTTCCCATCCTAAACCCCCCTGCAAGGGAAAATCTACAACTCCTCC
AACCACAAGTGGACCTGCAACCAGCACTTCAACAATCACTACTGAGGCCAGTTCCACAACACCTTCAGTTACTAATGCCACTACACCTGCACCTGCC
AACACTACAGTCAGCAGAGGGGATATCACAGCAGGGAATTGAGGGAAATGAAATTAATAATGCTCTTTCCGACCTGCAAAAACTGGCTGCCTAAAGC
CGATTCTGCCTTCCTTTTCTTTCTAATGAATTTTCAGAATAACACAGGACAGAAATATATATTCATAATGGGGAAAAAATTAATAGTTTACACCAGT
GAGGGACAAAAATGCAGTTTTTATGCCTAGTGCTAGCATTTATATGACCAAAAATGGATTTCTCATTGTCATAATGATGCCTGACTTTAAATTCATA
TTATCATTTTTTGTTCAAGGTTAGTTGTAGCTCATATCTCAGCCATTTCCTTTGATGTGTTTACTTCTATTTTTTTTGTCCACACTGACTTATATTT
CATCAGGGTATTCTTATCACATAGGAATTCTTACAAAATTCCTATAGCACTTTTCAGTGGATGTACAGAGTGAGCCATTCAATGTTTTTCTAAAACC
AACTTGTGTATGTCAGCAATGCATTGCCTTGGTCTCTTTCTTAATTAAACAAATCAATACTTGCTATGTGATGAGCATGTATGTGACCATAATTACT
TTTGGGCAGCTGTAACTTTCAGAACTCATAATTAATAAGAAAGGCCATTGGCATTGGCAGGTGTCCCTCATTTTGAGTCCTTCTGATGTAACGCGTT
ATTTGCAATATTTACTAATGATTAATTATTAACCTTTCATGCTATATGTACTCATCTAATAAACATCTTTCAGCATTA
>scpplpq14-like
ATAACTTTTTATTTCTATAGGACACATCACTCCTGCATCAACAACACAACACCTGCATCTTCTGGATAAGAAACTGATTCAAAAATGAAGATTTTTC
AGATCATGGTTTTCATTTTCCTTTGTGTGGCAGCAACAACTTCACTTCCAAGAAAGAAAGCCAGTTCAGAGACAGCATCATCAGAAGAGAAGTCGTC
CTCTGAAGAGATCTTGTTGAAGCCAACCTTAGTTGTGCCAAAGACCAAAGTTGTGCCAGTCACCGCTGTTCCTTCCAAGACCACAGTCCAGCCAGTG

ACCAAAGTCCATCCAGTGACCACAGTCCATCCAGTGACCACAGTCCAGCCAGTGACCACAGTCCAGCCAGTGACCACAGTCCAGCCCATCGGCACTG
TTAGCCAAAATACCACAATATGAAATGTTGTGCCAACCACTAACAGGAATGTTGTCACAAAGAAATGTCTGACCACAAGCCAAACCTCTACTGACAA
CAGAAGGAATGGAAAATTGAGTTGAACAAATTAGTTAATTTTAAATTCTATTAGTGTCTTTGTGTGCTAGTCTGTAACTGCATGTACTTTCTCATTA
ATCTGCTTGATAATAAAACAAAATAAAAAATCAATAAATGAACTAATAGCCTGTGTGAGATATACATCTATGTTGATATTGGCACTATTGAAAGCAA
ATGCTATTTCACCTTGAAAGTGTATCAAATGCATATAGTTTAGAATGAAGCACATATAACAATAGTATCTGTTTAGAACTGGCAATATTAAATACAT
TTACAAAGTTTAATATCTCGTATATGAAGTATATTAATATGTTAATAAATTAATGTTATACTATAAAGCTATAAACTAGAAAATCTATCAATTAATA
ACAACATTAATAAGTTGGTTATCATTTCCTATATTCACCAGTTTGTAAACTATATATATATATATATATATAATATTTATATTTCAGTCATGACTATGG
CAATGCTTAAGAAGTAGACACAACAAATCAGGTCTGAAATCTGCAAATTGCAGAGGAATGGAGAACAGTAGAATCCAGTTAACAAAAATTACAGATC
AGTCATAATGTGTGGAAGTGGTGAACTGAAGTATTTTGGGATTGTGTTTTGGGACATGTATTTCTCAATCTTTGTTTAATCCAGTCCTTGAAAACAA
TGGTGATATCCCAAGGGAAATCCAAGGGTGGATAAGTTACATATTTTAGGGCTTTTTCATTGATGTATCCCATCCCTGACATTCCATGTAAATAAGC
CCACAAATTACATTGCCCAACCCTGACTACACCATTTGGGGCTAATCTAAATGTGTGATTGTTGAATTTAAA
>scpplpq14: 3’ end missing
TGGGAATCAAATTTGGATATCACTTTATTATACCATACTGGTTGAGCACCAACAATAAGAAACAAACAAACAAACAAACAAACAAAAATGGCGATTC
TGAAGACCATGTTTTTAATTTACCTTTGTGTGGCAGCAACAAATTCATTTCCAAGCAAGTTCCCGAGACCATCACATACAGACAGTTCAGAGTCAGA
GTCGTCTGAAGAGAGTTCCTCTTCTGAGTCCTCATCCTCTTCCGAAGAGATGTTCCGAAGGACAAGCACCACAGTTCAGCCAGAAACCACCACCAAT
GCTGCCACCAGCTGTTTCACAGAGGAAATTCCAACTGAAATCCCAACCACAGTCAACCGAGGAGATGCTTATCGGAGTTAAAGAAACTGTAATAAGA
AATTGATTAGTTGTTCATTTTAAACTATTTTCTTTCTGTGCTAGACCTTAAATTGATGTAGTTTCTCATTATTGATATGTAAAATGGTTCCTGGTTA
CACAAGAGAATGGAAAATGGTTAGTCAGCAAAACCTAGAGTCCTTCATGGCGTAGTGTGTTACCAATTGTTTTCTTGGTGACTATGGTCCCA
>scpplpq20: exon 1 missing
ATACTCCTTGCAAAATGAAGGAAATTATTCTGTTCACCTGCTTCCTAGGACTCACACTTCAAGCGCCTACGCCTCAGGAACAAACCGCGGGCCCACC
CAGTATAGAAATTCTTCTTCCATATGGATTCCAAGGCCAGGCATTTGGGACGCCACAGCACGCAGCACACATTCTCCAGCAAAATGGACTGCCAGCA
CACGCCAGCATAGAATTTCTGGTGCCGTATGGAGGCCCCCACCATCAGTCTGGTCTCCCCTCGCAGATGGGACAGATGTTTCCCAACCAAGGGTACA
TCAAACAGAAGAAGCTGCAGGCGCCCGGAGCAGCGAGCAACGAGCACAATAATCCCGATCCTGCTGCTGAACAGACACCCCAACAAGACCCAGCTGG
GCCTGCTAGCATTGAAATCCTCATGCCTTATGGCTTCACGGGGCAGCAGTTCGGGATCCCAAATACCCAGAACCCCTCTCAGCAGATGCCTCAGCAC
CCGGGTCATGTGAGCATCGAAATGCTGTACCCCTATGCGTTCCCAGGACAGGCATACGGATACCCTCAGCAGCATGGGATGCAGAATGATGCCCCCG
CCCCCCCGGCCGCCGCCGCCGCCGCCGCCGCCGCCCCCCCGATGGCGCCGGGAGCCGTGGGCCACGCCAGTGTGGAGATTTTGTATGGATACGGATT
CCCGAACCAACAACAAGGACTCCATCCATCAATTCCTTCTCAAGGATTTTTGAAACGCAAGATTCCCCAGCCGGCCGGAGCGCGCAGTCTGGAAGTG
CTGTACCCGTTTTCATACAGACCAGAGAACCCGATGTTCCCGTTCGCTAACACAGCCCCCGCGACGCCGGAGCAGCTCAATTCTCAGAGCCCGAACG
TGAGGAAGCAATCTTCTCATCTGCGGCAGGAGGTTCCGCCTCCAGCCGAACTCTTCCCGAAGCAGCACCACCAGGTTATCCCCACTGTAATTCCCGG
TGACAGGATGCAGCAGGAGCCCGAGCAAGCGGTTCTGCCAAGTGCCAAAACCCCTGTTGAGCAGGAGGATTAATTCAAAGTGATCTTCGCTGGAGTG
ATGACATCCTGGCAATGAATCCCTCCTTTCTTACATCAGGCTGCATCTTCAGTTAATATATATATATATATATTAGACGTCCAATTTGAATTAGCGG
TTCAGCTGAAACTTTTACAATGTGTTCTGTTCACAAACCCCAGGTGTGAGTCACGCACTACTGATTTTAAATCATATTGACATTTGTTACATTTGTC
TTCCCAAACAGAAGAAATGATAAAAACACAGACTCACATTTTGCTGACAATAAGTAATCACAAACAACAGTACAGTCCTAGGTTTTATATATTATAA
TATTATATATTCACTGCACATATGATGTCATTACTCTCTGCTTTCTATGGTATTGATTACTTGCTCCAAATGTCTATGTAATCCAAGTCCAGAAGTG
AATGGATATATTTTAGAAAGCTTTTATAGATTCTTTGTTGTTTTAAGAAACTGATTTTGTGAAAAACATGACATTTGGCAACCAGTTCTTTTGGGAC
ACATCTGTGTTTAATTATAGAGCTTCGTTATATCAAACAGTCGGGGAAAATTAAATACGAATTATTATGTTGTTTCTTTATCTGTATGTCACCATTT
GGAAGAACATTTTGTTATGTCTGCTGCTTGAGATTCTAATCTTTTAAACAAATGATCTATTTAATGTAGAAATAAATATTCCCTTAAAACACTACCA
CTGGCTTTTTATTTCATCTGGTGCTTGTTATAC
