# Supplementary material for: The bowfin genome illuminates the developmental evolution of ray-finned fishes
Source: Nat Genet. 2021 Aug 30;53(9):1373–84. doi: 10.1038/s41588-021-00914-y (PMC8423624; doi:10.1038/s41588-021-00914-y)
Supplement: Supplementary file 6 — Bowfin Hox gene transcripts. [file 41588_2021_914_MOESM6_ESM.pdf]

Supplementary Data 2. Bowfin hox gene transcripts.

>Aca_hoxA01_scaf10
ATGAGCTCCTTCCTAGATTACCCTATGATCAGTGGAGAAGCCGGGTCCTGCTCTTCCAGGGCTTTTCACCCCG
AGCATGGAATTACAACTTTCCAGTCGTGCGCTGTCACCACTAATAATTGCGCCAGTGATGATCGCTTCATGAT
CAGCAGGGGTTCACAGGGTGGCATCCCTCACCACCCTCACCAACCTGCCGCAGCCTACCAGCCTCACAACACT
CTGGGCATCCCCTACGCCAGTCACCCCAGCTGCGGCACCAGTTATGGCGCGCAGAGTTTCTGTGCAAGTTACA
ACCATTACGCACTGAACCAAGATATGGACTCGAGTGCGGGGTATCCTCAGTGCGCCCCAACAGTTTACTCTGG
GAACATCTCGTCCTCTGTGGTTCAGCATCACCAGGGGTACAGCGGAGCATCACTGGGGGCACTTCAATACCCA
AATGCTGCTTACGGGCACGAACAGCCGAACCTGTCATTTGCAGGCTGCACTAACCCCTTATCTCCATTGTGTG
CCACTCACCAAGAATCCTGCTGTTCTCCTTTATCCGAAGCATCCCCCCCAGCACAGACATTTGACTGGATGAA
AGTCAAAAGAAATCCGCCCAAAACTGGCAAAGTTGGCGAATATGGCTTCGCGGGTCAGCCCAACACAGTGAGG
ACCAATTTTACAACCAAGCAGCTGACCGAGCTGGAGAAGGAGTTTCACTTCAACAAGTACTTGACGAGAGCCA
GGCGTGTGGAAATAGCTGCCGCCTTGCAACTCAATGAGACACAGGTAAAGATATGGTTCCAGAACCGGAGAAT
GAAGCAGAAGAAACGGGAGAAAGAGGGGCTGTTACCCAGTTCCCCCTCGACCCCGACAGAAAGTGACGAGAAA
GCCGAAGAGGCGTCAGACAAGTCCCTCTCCGCCCCTTCAACACCTTCTCCCGCCTCGTCTGCAGCCTCCACAG
TAGAAACATCTACCTCCACGTAA
>Aca_hoxA02_scaf10
ATGAATTACGAATTTGAGCGAGAGACTGGTTTTATCAATAGTCAGCCGTCGCTTGCTGAGTGCCTGACATCTT
TTCCCCCTGTCGGTGATACATTTCAAAGTTCATCAATCAAGAGCTCGACGCTTTCACACCCGACACTGATTCC
TCCTCCTTTTGAGCAGACCATTCCCAGCCTAAACCCAGGCAGCCATCCTCGCCACAGCCGCCCAAAACAGAGC
CCCAATGGCAGCAGCCCCTTGCCGGCCGCTTCCCTTCCCCCGGAGTACCCCTGGATGAAGGAGAAAAAAGCTT
CCAAGAAAAATCACCTGCCGACTTCTTCAGCCACAGCTACAGCAACCCCGGGACCGGTCTGCTTCCCCCCGAA
AGACTCGCCCGAGATCCCTGATGGCGCTGGCGGGGGATCTCGCAGGTTGAGGACCGCATACACCAACACTCAG
CTCCTGGAGCTGGAGAAGGAATTTCATTTCAACAAGTATCTGTGCCGACCAAGAAGGGTGGAGATTGCAGCTT
TGCTGGATTTGACTGAGAGGCAAGTGAAAGTGTGGTTTCAGAACAGGAGAATGAAACACAAGAGGCAAACGCA
GTGCAAAGAGAACCATAATGGCGAGGGGAAGTTTAAGACTCTGGAGGACGGTGGGCAAAGCGAGGAGGAGAAG
TCCCTCTTCGAGCAAGCCCTCAACAATGTCTCCGGAGCTCTCCTGGAAAGGGAGGGTTACACATTTCAACAAA
ATTCCCTGACTGCACAACAGGCTCACAATATGCACAATGGAGATGTCCAAAGTTTCACTGTTTCGCCTTTAAG
CAGCAATGACAAAAATGTGAAACATCTCCAGAACCCGTCCCCCACTGTTCAGAACTGCTTGTCAACAATGGCC
CAGAACTGCGCAGCTGGCCTGAACAATGACAGTCCCGAGGCCCTGGACGTCTCCTCTTTGCAGGATTTTAACG
TTTTCTCATCAGATTCCTGCCTACATCTCTCAGATGCTGTTTCCCCCAGTTTGCCAGAATCCCTCGACAGCCC
TGTGGACATTTCTACGGACAGTTTTGATTTTTTCTCGGACACTCTCACAACCATCGACCTCCAGCATCTGAAC
TATTAA
>Aca_hoxA03_scaf10
ATGCAAAAAGCGACCTACTACGACAGCTCTGCGATATACAGTGGCTACCCCTATCAAGGCGCAAATGGTTTCG
GTTATAATGCCAATCAGCAGCAATATCCGCCCTCATCTCATGTGGAAAGTGACTACCATCGACCTGCCTGCTC
CCTGCAGTCACCTAGCGCCACCGTTCCCCACCAGAAGCCCCATGACATCGCAGAGAGCTGCATGCGGAACAGT
GCTGTCCAGAGCAGCCAGCCCCCGGTTATCCCCGACAGCCACCAACAACCGCCAAGCCAGGCCCCGGCAGCTG
CGCAGCCCCCCCCGCCGCCCGTCTCTCCACCCCAAAACACCAGCACCAGCTCCAGCCAGTCCAACACAAGCAA
GACCCCAGCGCACAGCTCTCCCAATGCGGCCACCAGCAAGCAGATTTTCCCCTGGATGAAGGAGTCCCGCCAG
AACTCCAAGCAGAAAAACAATAGTTCCAGTTCAGTGGAGAGCTGTCCCGGGGAGAAGAGCCCCCCCGGCTCCG
CCGCGTCCAAGCGAGCCCGCACCGCCTACACCAGCGCGCAGCTGGTGGAGCTGGAGAAGGAGTTTCACTTCAA
CCGCTACCTTTGCCGGCCGCGGAGGGTGGAAATGGCCAATCTGCTCAACCTCACGGAGAGACAGATCAAAATC
TGGTTTCAGAACCGCCGGATGAAGTACAAAAAGGATCAAAAGGGCAAGGGGATGATGCCTTCCCCCGGAGGAC
AGTCCCCGAGGAGCCCCGTCCCCCCGGGGTCTTCAGCAGGAGGATACCTGAACTCTATGCATTCGCTGGTGAA
CAGTGTGCCGTATGAACCCCAGTCCCCCACGTCATACAATAAGCCTCATCAAAATTCATACCCTCTCTCCACG
TCATATCCGGCGCCTCTAAATAATTGCCCGCCTCCCCAAAAGAGATACGCCGGGACTGCCGCCGTTACGCCTG
AATACGACACGCACCCCCTTCAAGGCAATGGCAGCTATGGGACTTCCCATCTGCAGGGGAGCCCGGTGTATGT
GGGAGGCAGCTACATAGATCCCATTGCCAATTCCGGGCCCTCCCTTTTTGGCTTGACACACCTCCCTCACCCA
TCATCAGCCAACATGGACTACAATGGCGCGGGCAACATGGCCAGCAACCACCACCATGGACCTTGCGACCCAC
ACCCCACATACACAGATCTGACTCCTCACCACTCGTCTCAGGGAAGAATTCAGGAAGCGCCCAAGTTGACGCA
TCTGTAA
>Aca_hoxA04_scaf10

ATGGCCATGAGTTCGTATTTGATCAACTCCAACTACATCGAACCTGCTTTCCCGTCTTGCGAGGAATACTCGC
AGACCAGCTACATACCCACCCCCTCTCCTGAATATTATGACCGGCCGAGAGATCCTGGATTCCCACATCGAGA
GGCGCTCTTCCAGCGGTCAAACTACAGGGACCAGAGCTACGACTACAACAACACTGTCCATCCTAGTGGGCAG
GAGGACTTTCCCCAGCGAGGACATGATCCAGCCCAGCCCGTCCTGCAGACCCACATTGCGCGGCGGAACCAGG
ACTGTGAAAGTGTGGCAGTGACCACAGACAGCAGCCTGCCTGAGAAATCCCCAGCTGGCCAGAAGGCTAAAGA
GCCCGTGGTTTACCCCTGGATGAAGAAAGTGCATGTTAACAGCGTAAATAGCACGTATAGTGGAGGAGAGCCA
AAGAGATCCAGGACTGCCTACACCCGACAACAGGTCCTCGAACTCGAGAAGGAGTTTCATTTTAATCGATACC
TGACGAGACGTCGACGTGTCGAGATAGCGCACACCCTGTGCTTGTCTGAACGGCAGGTCAAGATTTGGTTTCA
GAACAGGAGGATGAAGTGGAAGAAAGATCACAAACTGCCCAACACTAAGACACGCTCCACCAACTCGTCACCT
TCAAGTCAGCAGTCAAAGGCACCATCTCAGATCCCTCAGTCAGTTGCACCAGTTCCAGCTGTTTCGAATCTAT
AG
>Aca_hoxA05_scaf10
ATGAGCTCTTATTTTGTCAACTCATTCTGCGGTCGCTATCCTAATAGCCCGGACTACCCGTTGCATAATTATG
GAGATCACAGCTCGGTGAACGAGCAATACAGGGATTCTGCGACCATGCATGCCAGCAGGTACGGCTACGGCTA
CAATGGGATGGATCTTAGTGTCGGGCGCTCGACCTCGAACCACTTTGGTGCTGGTGAAAGAGCTGCCAGCTAC
ACATCTGGTGCAACAGCAGCCGCTGCAGAGCCCAGGTACAACCCACCTGCAACCGCGGCTCACTCGCCTCCAC
CTGACCCTCTACCTTGCTCCTCTGTTGCCAGTTCGCCTGTCCGCGAAACTCACCGCGCTGGTAAAAACTCCTT
AGCCAGTCCCACTACCTCTTCATCCAGTTCAAACAACAGCAGCTCGCTCTTGAGCAGGGACGCTGTGGGGAAG
GCGCCTGCCGCTGAGGATGACCACCCGGCCAGCAGCGCCCAGACAAGTTCACAAAACGGCCAAAACGCCACCG
AAACCGCACAGCCCCAGATATACCCCTGGATGCGAAAGCTGCACATAAGTCACGACAGCATGGCAGGACCTGA
AGGGAAGAGAGCCAGAACAGCCTATACACGATATCAGACTCTGGAGCTGGAGAAAGAGTTCCACTTCAATAGA
TACCTGACCCGAAGGAGGAGGATTGAAATCGCACATGCCTTGTGCCTCTCGGAGAGACAGATTAAGATCTGGT
TCCAAAACAGGAGAATGAAATGGAAAAAAGATAATAAACTGAAAAGCATGAGCATGGCAGCAGCGGGAGGCGC
GTATCGTCCCTAA
>Aca_hoxA06_scaf10
ATGAGTTCGTTTTTTGTGAACCCCGTGTTCCCCGCGTCCTTGCCCAGTGGTCAGGAGTCCTTCCTCGGACAGA
TGCCATTGTATACATCTGGCTATGATGCTCTAAGGCACTTTCCAGCTTCCTATGGCACGTCTGCGATACAGGA
CAAGACTTATCCATCACCGTGCTTTTATCAGCAATCCAATACTGTGCTTAATGCAAGGGTACCCTATGAGTCC
GGGCCGTCCTGTTACCCTCCGGAGAAGGACCCAGCAAACCCCTACAGCACCGGCAGTGGCAAGCAGAAGGCAT
GCAACCAGTATGGACAACTTAACGTCAACCAAGAGTTTAAATCCGACTGTGCTCAAAACAAAATCTTCTTTAA
TGAGTCCCCGGACCGGAAGTTCCCTTCCTCCGTTTATCCATGGATGCAACGCATGAACTCTTGCAATGGGTCG
TTTTATGGAACACATGGGAGGCGAGGGCGCCAGACTTATTCCAGATACCAAACTCTGGAGCTCGAAAAGGAGT
TTCATTATAACAGATACTTGACCAGACGTCGCCGGATAGAAATTGCCAACGGCCTTTGTCTAACGGAACGACA
GATTAAAATATGGTTTCAAAATCGGCGCATGAAATGGAAAAAGGAGAACAAGATTTTAGGCACCACACAGGAA
AATAGCCAAGAAGAAGAGAAAGAAAAAGCTGCGAAATCATAG
>Aca_hoxA07_scaf10
ATGAGTTCTTCTTATTATGTGAATAGTCTTTTTAGCAAATATGCGGCGGGGACTTCCCTGTTTCCCAATGTAG
ACCAGTCCTCCTGCTCTTTCGCACCCGGCGCTCAGAGAGCCGCATATGGACCGGGGACAGCCGCCTTCTCCTC
GTCTCTACCCGGGCTCTACAGCGTCAATAATGCCATCTACCAGAACCACTCGGTGTTCACCTCCGGGTACAAC
CTGGGCTCGGACGCCTACAACCTGCCCTGCAACTCGTTTGACCAGAACATGCCCGTCCATTGCAACGACCTGG
CCAAAGGCAACTGCGACAAAACGGACCCCCTGAGCATCCACTCTCAGGCTGATAGCAATCTCCGGATGTACCC
CTGGATGAGAAGTTCAGGTGCTGACAGAAAGCGGGGCAGACAGACCTACACCCGGTACCAGACCCTGGAGCTG
GAGAAGGAGTTTCATTTCAACCGCTATCTGACTCGCCGGCGGCGCATCGAGATCGCCCATGCCCTGTGCCTCA
CCGAGAGGCAGATCAAAATCTGGTTTCAGAACCGCAGGATGAAGTGGAAGAAAGAGCACAAGGAAGACAGCTC
CATCTCCAGCACAGCCCCCAAATCCACAGCGGAGGAGAAGGAAGAGGAGGACGAGGAGGAGGAAGACTGA
>Aca_hoxA09_scaf10
ATGTCGGCTTCAGGGACTCTCAGTAATTATTATGTGGACTCTCTTATTATCCACGAGAGTGAGGATCCTTTAC
AGCCCAGATATCCCACTGGTCCAGGGGTTCAACAAGCAAGACAGCCAGCTTTGCCCGAGCACACCGAGCTGAC
CCCATGCAGTTTCCAGACGAAATCCTCTGTGTTTGGCACATCCTGGAGCCCCGTGCATGCTCAGCCTCCCAGC
AGTGTGGCCTCGGTCTACCATCCGTATGCGCATCACCAAGGGCCAGCTGGGGAGTCCGATGGAAGGTACTTGA
GGTCTTGGTTACTGGAGCCCATGTCTGGATCCCTGCCTTTCCCCGGATTACCATCGAACAGGCAGTACGGTGT
CAAACCCGAGCCTCTGAGCCGGAGAGGCGACTGCGCCACGTTTGAGACGCACACCCTGGTTCTGTCCGATTTT
GCCACTGGATCCTCCCCGGCAGAGAAGGAGAAGCATCCCAGCGAAGTAGCCTTTTCCGACGGGAATGGAGAGA
ATGTGTCAGCCGAGGAGAAATCAGAAGTTGATGCTAATAATCCTTCTTCAAACTGGCTTCATGCGAGGTCGAC

AAGAAAAAAACGCTGCCCTTACACTAAGCACCAGACATTAGAGTTGGAAAAGGAGTTTTTGTTTAACATGTAC
CTCACCAGAGACCGTAGATACGAAGTGGCAAGACTCCTAAATCTCACCGAAAGACAGGTCAAAATTTGGTTTC
AGAACAGGAGGATGAAGATGAAGAAAATCAATAAAGATCGGCCGAAGGATGATTAA
>Aca_hoxA10_scaf10
ATGTCATGCTCAGACAGCCCGGCTGGAAACTCATTTCTAGTAGACTCCTTGATCAGTGCCAGCCGAGCCGAAA
GCGGTGGCGCCTACTATCAGGGCAGTGGCGTTTACTTGCCACCAGCTTCTGAGCTGCCTTACGGACTACCCAA
TTGTGGATTTTTTCCAGGATTAAGTAAACGGAGCGAGGCGAACTCCCAGAATATGGTTCCCACCTCCAGCCCG
TACATGCCCGGGATGGAGGTATGGCTGGATCCACCGAGATCATGCCGCATGGACCCACCTGCGAATCAGCAGG
TAGCGCCCTGTTCCTTCTCGCCGAGCATTAAGGAGGAGAGCGCCTATTGCCTTTACGAGTCGGAGAAGTGTCC
AAAAGGGTCAACGGCAGAAGACCTGTCCTATTCAAGGCTAACATCTGGCTCCTGCGCCGTCAGTGAGGGCGGC
ACGGTTCCTGTACCTGGTTACTTCCGCCTCTCTCAGACTTATGCAACTTCAAAGGTGTATCACGGTGTTCAGT
CGAGTTCGTCTCCGTTTGTCCTGCAACCGCCGGTCCGTTTCGAGACGCCGCCGTCGTCCGCTGCCTCTACAGA
GACTGGCAGGAGAGGCAGTGAGGAGGCAGCCCCCAGCACTTTACCGTGCGTCCCCCAAAGAGAGGAGGAGAAC
AGGGTCTCTTCGGCTGCAGAGGACTCCTCTCCAGACCCCACAGAGAATGGCAAAACAAGTCCCGACAAAGCGT
CCAAAGGAGATACGAAGAATGAAAACACAGCAAACTGGCTGACAGCAAAAAGCGGAAGGAAGAAACGCTGTCC
CTACACCAAGCACCAGACTTTGGAGCTGGAGAAGGAGTTCCTGTTCAACATGTACCTGACTCGAGAGCGGCGC
CTAGAGATCAGTCGCAGTGTCCACCTGACGGACAGACAGGTCAAGATCTGGTTCCAGAACCGCAGGATGAAAC
TGAAGAAGATGAGTCGAGAGAACAGGATCCGGGAACTGACTGCGAATTTCAGCTTTTCGTGA
>Aca_hoxA11_scaf10
ATGATGGATTTTGATGAGCGGGTTTCTGTGGGCTCTAACATGTACTTACCCAGTTGTACGTACTACGTCTCCG
GTGCTGATTTCTCCAGCCTCCCTTCCTTTTTACCCCAGACCCCGTCTTCTCGCCCCATGACGTACTCCTATTC
ATCTAACCTGCCCCAGGTCCAGCCTGTGAGAGAAGTCACCTTCAGGGACTATGCCATTGATGCATCCAGTAAG
TGGCATCACAGAAGCAATCTCTCCCATTGCTATTCGGCAGAGGAGATCATGCACAGGGACTGCCTGCCTGCCC
CTACCACCATGGGGGAAATGTTCGCGAAAAACAACTCCACTGTCTACCACTCGAGCTCCAACTCCACGTCCAA
TTTCTACAGCAGCGTGGGGAGGAACGGTGTGCTGCCCCAAGCCTTTGACCAATTTTTCGAGACGGCGTACGGG
AACACAGAAAATCCGCCGTCCGACTACTCTGGGGACAAAAATGCCAACAAACTGCCTCCTGCCGCCGCCGTGC
CGGGGTCTGATCCATGCAGGGAGACAGAGGCGAAGGAGCGCAGGGAAGAGAGCAGCAGCCCCGGGTCCTCTTC
CGGCAACAATGAGGAGAAATCCAGCAGCACCAGTGGCCAGCGTACTCGTAAAAAGAGATGCCCCTACACAAAA
TACCAGATCCGAGAATTGGAGAGAGAGTTTTTTTTCAGTGTCTATATTAACAAAGAGAAGCGCCTTCAGCTGT
CAAGGATGCTCAACCTCACTGACCGGCAGGTTAAAATCTGGTTTCAAAATAGAAGAATGAAAGAAAAGAAATT
GAACAGAGATCGGTTACAATATTACACAACCAATCCTTTGCTTTAA
>Aca_hoxA13_scaf10
ATGACAGCGTCATTGCTTCTCCATCCCCGCTGGATTGACCCGGTGATGTTCCTCTACGACAACGGCTTAGATG
AAGTGAACAAGAACATGGAAGGCTTTGCAGGAGGCAACTTTGCTGCGAATCAGTGTCGGAATCTGATGGCCCA
TCCTGCATCCCTGGCTCCCAGCACTGCCTACACGTCCAGTGAGGTGCCAGTGTCGGGCATGGCCGAGCCTGTC
AAACAGTGCAGCCCCTGTTCTGCAGCCCAGAGCTCCTCCAGTGCGTCTCTGCCCTATGGATATTTTGGCAGTG
GCTACTACCCCTGTAGGATGACCCATCACAGCAGCATTAAGTCCTGCGCGCAGCCTGCCTCCTATGCAGAGAA
GTACATGGACACGTCGGGCTCAGGCGAAGACTTCACGTCCAGAGCAAAGGAATTTGCGTTTTATCAAGGCTAT
GCTGCTGGCCCCTATCAGCCCGTGCCCAGCTACTTGGACGTGCCAGTAGTCCCTGCTATTAGTGGCCCCGGGG
AACCCAGGCATGAACCACTGTTGCCCATGGAGAGTTACCAGCCGTGGGCCATTACCAATGGGTGGAATGGTCA
AGTTTACTGCTCGAAGGAGCAGAGCCAGCCGACACATCTGTGGAAATCAACCATTCAAGATGTCGTCTCACAC
CCAGGGGATGGCAACTCTTTTCGACGTGGGAGAAAGAAACGGGTGCCCTATACAAAGGTGCAGCTTAAAGAAC
TGGAGAGGGAATACGCTACTAATAAATTCATTACAAAAGACAAGCGAAGGAGGATATCCGCTCAGACAAACCT
GTCCGAAAGACAAGTCACTATCTGGTTCCAGAACCGGAGAGTCAAGGAGAAGAAAGTCGTGAATAAACTGAAG
AGCAGCAGTTAG
>Aca_hoxB01_scaf2
ATGGACAATACCAAAATGAACTCTTTCCTAGAGTATACAATCTGTAACCGTGGGACGAGCGCCTACTCACCCA
AGACAGGCTTCCACCACTTGGATCAGGGAATCGCTGCGCCTTTCCCCCCCAGCTCAGGTACAACAAGTGACAG
CTTCAACACTGACGGACGCTTTTTCGTTGGAGGCAGCGCTTCCGCCGCCCCGGCGCAGCATCAGCACCAAACC
CCGACCTACCCTCACCATCACCCGCACCCCCATCACACCAACATGGGGATCCCTTACGCCAGCACGGGGGCCA
CAGCCTATGGCTCCCAGACGTGCTCGAACCCGGACTATGGGCACCAGTATTACCTCAACCAGGAGCAGGAAAA
CGTTTACTTCCAGTCCTCAGGTTACTCCGCGCCCAGTATGGGGCCCAACCTCGGCTCTCTGGCTGGTGGCTAT
TGCGGGGCCCCCGGAGCTGTGGCTGCCAGCCAGTACCCGCACACAGCCTGTCCGGGTCAGGAGCAGCAGCAGC
AGCAGCAGGGCTATCTGCACAGCTCCTTCTCCAGCCTGTCTCCCACCCAGACGGGGGAGTCAGACCTGAGCTG

CCAGGCAGAGCAGAATTCGTCGGGTCAGACGTTCGACTGGATGAAAGTCAAGAGGAACCCCCCCAAAACAGCC
AAAGTAGCTGAGTACGGTCTTCAGGGCCAGCAGAACACCATTCGGACCAACTTCACCACCAAGCAGCTGACGG
AGCTGGAGAAGGAGTTCCACTTCAACAAGTACCTGACGCGGGCCCGGAGGGTGGAGATCGCCGCCGCGCTGGA
GCTCAACGAGACGCAGGTGAAGATCTGGTTCCAGAACCGGAGGATGAAGCAGAAGAAGCGGGAGAAGGAGGGC
TTCGCGCCCGCCGCAGGCCGGGGCTCCTCCAAGGACCTCGGGGAACACTCGGACAACTCCAACTCGACGTCAC
CGGACGCGTCTCCAAGCTCGGCAACCTCTTAA
>Aca_hoxB02_scaf2
ATGAATTTTGAATTTGAGAGGGAGATTGGGTTCATAAACAGCCAGCCTTCACTTGCAGAGTGCCTGACGTCTT
TTCCCGCTGTCCTGGAGTCATTTCAAACTTCATCAATCAAGGAGTCGACATTAATTCCTCCTCCTTTTGAGCA
CACGATCCCCAGCCTGAATCCCTGCACTACCAGCCAGCCGCGACCGAGAACCCAAAAAAGAGCTGCCAATGGC
CTCCTGCTCCGGACCCCGGCACAGCAGAGCTCGGCTCCGCCAGCGGCTGCCGGCCCTCTGGCTGCCGAGTTCC
CCTGGATGAAGGAGAAGAAATCCTCGAAGAAGACCCAGCAGCCGGGGAATGCCACCGCCACCACCTCCTCCCC
CTCTCCCTCGTCCTCCAGCTTCGTAGCAGCGGGTCTGGACTCTCCAGCAGAAGCGCAGAACGGGCTGGACAGC
ACCGGCGGGTCTCGGAGGTTGAGGACAGCCTACACCAACACACAGCTCCTGGAGCTGGAGAAAGAATTCCACT
TCAACAAATACCTCTGTCGGCCTCGCCGTGTGGAAATTGCTGCCTTGTTGGATCTAACGGAAAGACAGGTCAA
AGTGTGGTTCCAGAACAGGAGAATGAAGCACAAACGGCAAACCACGCACCACAAGGACGGCCAGGAAGGGGAC
CCGGGTGGGTTCGACTCCATGGAAGGCACCGAGGCGGCCTCCCCGTTTGCTAGCCAGCCCCTGGAGGCGTCGG
GGTCTGCCGCCTCCGAGAGCGAAACCTGTAACTCCGCTTCGTCCTACCCGAACAGCAATGACAATACGCAGCC
CGCCTCCGAGGAAGCACCAACAGCACGGCAGGAGCCGGCATCTGCCCCCGACAAAGCCGTCAGATCTCCCGTG
TACCCACCGCCCACTACAGATCACCTCCCAACAATGGGCCCGGCCTGCGCATCTGCCCTGGATAATTCGTTCT
CAGAGCCGCAGGACCCTGCCACTCTGTCCGATTTGAATTTCTTTTCTGCAGACTCCTGCCTACAGATTTCAGA
CGCTTTGTCGCCCAGTTTACAAAGCTCCCTGGATAGTCCTGTCGACTTTTCCGAGGAGGATTTCGACTTCTTT
ACGAGCACACTTTGTACTATAGATTTACAGCATTTGCAATTTTAA
>Aca_hoxB03_scaf2
ATGCAGAAAACTACCTACTACGACAACTCGACACTTTTCGGAGGCTACTCGTACCAAGGGGCCAACGGCTTCG
GCTATGATGCCCCCCAGCAGGCCTACCAGCCCTCGACCCACCTGGAGAGCGACTACCAGAGATCGGCGTGCTC
GCTGCAGTCCCTGGGCAATAGCACCTCCCAACACGCCAAAACGAAAGACCTCAACGGGAGCTGTATGCGCCCC
AGCCTACCACCTGAGCATCACCAAGCCCCTCCCGTCTCCCCTCCCCAGAACCCCGCCAGCACCACCAACAGCA
GCTCGGCGCAGCAGCCGGGCAGCAGCAGCAGCAGCAGCAGCAGCAGCAGTAAAACAGCCCCCTCCAAGTCCTC
TTCTCTGCCCCCCAACCCCAACCTCACCAAGCAGATTTTCCCCTGGATGAAAGAGTCGAGACAGACCTCGAAA
CAGAAAAACAGCTCCCCCAGTACAGCGGAGAGCTGCAGCGGCGAGAAAAGCCCCCCGGGTTCTGCTGCTTCCA
AGAGAGCGCGCACGGCCTACACCAGCGCGCAGCTGGTGGAGCTGGAGAAGGAGTTTCACTTCAACCGGTACCT
GTGCCGGCCGCGCCGAGTGGAGATGGCCAACCTGCTCAACCTCAGCGAGAGGCAGATCAAGATTTGGTTCCAG
AACCGGAGAATGAAGTACAAAAAGGATCAAAAATCGAAAGGGATGGGCTCCTCCTCGGGCGGGCCCTCTCCCA
CCGGCAGTCCGCCCCTCCCCATGCAGTCCTCGGCCGGTTTTATGAACTCCATGCACTCAATGACTAGTAACTA
CGACGCGCCCTCCCCGCCCTCCTTCAACAAGCCTCATCAGAACGCCTATGCCATGTCCACGGCCTACCAAAAC
CCCATGAAAAGCTGCCCCTCGCAACAGAAGTATGCCAACACGGCGCCAGAATACGACCCCCACGGGCTACAGG
GTAACGGTGGCAGCTATGGGACTCCCAATCTCCAGGGCAGCCCCGTGTATGTCGGAGGGAACTATGTGGATTC
GATGCCCGCCTCGGGCCCGTCTCTGTACGGACTCAACCACCTGCCGCATCACCAGGCTAACAACATGGACTAC
AACGGTGCCACGCAGATGACGTCTAGTCAGCACCACGGACCTTGTGACCCCCACCCGACATACACAGACCTTT
CAGCGCACCATCCTTCTCAGGGTAGAATCCAGGAAGCGCCCAAACTAACTCATCTGTAG
>Aca_hoxB04_scaf2
ATGGCCATGAGTTCCTTTTTGATCAACTCCAACTATGTGGATCCGAAGTTTCCACCCTGCGAGGAATATTCTC
AGAACGACTACCTACCCAGCCACTCTCCGGATTACTACAGTGCACAGAGGCGAGAGCCTGCGTTTCAGCATGA
GGCGATGTACCACCAGCGGTCGGGCTGTACCGACCCTGCTTACTCTTCCTGCCACGGTCCCGGGCAGCCCGCA
GTGGTCATGTCCCCCCGAGGTCACGTCCTTTCACAGCCCGGGCTCCCGAACCCCCTGCCCGAGCCGAACCACC
ACTGCGACTCCGCCACCCCGAGTCCCCCTCCTGCTTGCGGCCAGAACTCCGTGAACCAAAGCACTTCTTCGTC
CAGTTCATGCAAGGAGCCCGTAGTTTACCCCTGGATGAAGAAAGTCCACGTAAACATCGTGAGCCCAAATTAC
ACCGGGGGGGAACCCAAGCGCTCCCGGACAGCTTACACCAGGCAGCAGGTCCTGGAGCTGGAGAAGGAGTTTC
ACTACAACCGTTATCTCACCCGGCGGCGCAGAGTGGAGATCGCACACACCCTGTGCCTGTCCGAGCGCCAAAT
CAAAATATGGTTTCAGAACCGACGGATGAAGTGGAAAAAAGACCACAAGCTACCCAACACCAAAATTAGGTCC
TCTAATACATCCACCACAAACTCGACCGCCAGCCAGGCGCTCGGCAGTTCTCAGAACCGGGCGACTGGACCTC
CGCCCAGCCTATAG
>Aca_hoxB05_scaf2

ATGAGCTCTTACTTTGTAAACTCGTTCTCAGGGCGATACCCAAATGGCCCCGACTATCAGTTACTAAATTATG
GAACTAGCAGTAGCGCTATGAACGGTTCGTACAGGGATTCCGGCACCATGCACTCGGGCTCATACGGCTACAA
CTACAATGGGATGGACCTAAGCGTCAATCGCTCAACCTCCTCCGGCCACTTTGGGGCTATTGGTGATAACTCC
CGGGGTTTTCAGTCTCCGGCCCCGGAGTCCCGGTTCAGGCAGCCGTCTAGCTGCTCTCTATCGTCTCCCGAGT
CCCTGCCCTGTTCCAACAGCGAAACTCTCGGACCAAAAAGCTCATCGCCCCCTTCCGATCAGAGCGCCAGCAC
CGGCAGCAGTAACCTCGCCGCCAACAGCAGCACACATTTCACAGAACTAGACGAGACCAGCGCTTCTTCCGAG
ACGGAGGAAAGCACCCACAGAGCCAGCAACCCCATTCCCCGGACACAGCAGCAGCAGCAGCAGCAGCAGCAGC
AGGAGGCGACTACAACCTCCACCACGGCAGCGGCCGATGGACAAGCACCACAGATATTCCCTTGGATGCGGAA
GCTTCACATTAGCCATGATATGACTGGACCAGATGGGAAAAGGGCCCGAACAGCGTACACCCGTTACCAGACC
TTAGAGCTGGAGAAAGAGTTTCATTTCAATAGATATCTGACCCGGAGGAGGAGGATAGAGATAGCCCATGCGC
TCTGCCTGTCGGAACGGCAGATAAAGATCTGGTTCCAGAACCGAAGAATGAAGTGGAAGAAGGATAATAAACT
CAAAAGCATGAGTCTGGCCACAGCGGGTAGCGCTTTTCAACCCTAA
>Aca_hoxB06_scaf2
ATGAGTTCCTATTTTGTAAACTCTACTTTCCCCGTGACTCTGCCCGGCGGACAGGAGTCTTTCTTGGGACAGA
TTCCGTTATATTCCTCCGGATACACGGATCCTTTAAGGCACTACCCCGGCGCAGCCTATGGATCTGCAGGCGT
TCCAGACAAGGCTTACCCATCTACTTATTATCAGCAGGCTAACGGTGCCTACGGCCGGGCCAACACAGCCGGA
GCCTGTGACTACGCAGCGGCCAGTTTTTACCGAGAGAAGGACCCGGCGTGCGCGCTGTCCAGCCTGGAGGAGC
ACTCTCTCGGGCTCAGCCAGGAGCAGCGCAAGACAGACTGCTCGGGGCAGAATAAAAACATATTCGCTGAAAG
TGACGACCAGAAGACATCCGCTCCCGTTTACCCCTGGATGCAGCGCATGAACTCCTGCAACGGGTCGGTCTTC
GGCAGCACCGGCCGCAGGGGCCGGCAGACCTACACACGCTACCAGACCCTGGAGCTGGAGAAGGAGTTCCACT
TTAACAGGTATCTGACCCGGCGACGTCGCATCGAGATTGCGCACGCACTGTGCCTGACGGAACGACAGATTAA
GATTTGGTTCCAGAACCGCAGGATGAAGTGGAAAAAAGAGAACAAACTGATCAACTCTTCACAAACCAGCGGA
GAAGAGGAGGAAGAAAAGAGGACAGAATAA
>Aca_hoxB07_scaf2
ATGAGTTCATTGTATTATGCGAATGCTTTATTTTCCAAATATCAAGCTGCGAGTTCGGTTTTCCCAACCGGGG
TATTTCCCGAGCAAACTTCTTGCGCTTTTGCGTCCAATTCCCAGCGAGCCAGCGGCTATGTCTCGGGGTCCGG
TGGCACTGCCTTCTCCAGCTCAATGCCGGGTCTCTACAGCAGCGGCGGCAGCATGCACCCCCCAAACCAGAGC
GTGTACTCCGCCAGCTATGGCCTCAACGCGGGCTCCTTCAACATGCACTGCTCCCCCTTTGACCAGAACCTGC
CCGTGATGTGCGCCGGGGACCCAGCCAAGCAGACCTGCAGCAGCAAAGCGGAGCAGCGGGACTGTGAGCAGCA
GAATGAAGCCAACTTCCGCATCTACCCTTGGATGAGAAGTACAGGTACAGATAGGAAGAGGGGACGACAGACC
TACACCCGCTATCAGACTCTCGAGCTGGAGAAGGAGTTTCACTTCAATCGCTACCTGACCAGGCGGCGCAGGA
TCGAGATCGCACACGCTCTGTGCCTCACGGAACGACAGATTAAAATCTGGTTTCAGAACCGGAGGATGAAATG
GAAGAAAGAAAACAAAACCACCGACCAGAGCCCGTCCACTGAGGACAAACCCGAAGCAGAGGACGAGGAGGAG
GAAGAGTAA
>Aca_hoxB08_scaf2
ATGAGCTCATATTTTGTCAACTCTCTTTTCTCCAAATACAAAAGTGGCGATTCTCTACGTCCCAATTATTACG
AGTGTGGGTTTGCACAAGACCTCGGAAGTAGACCCACTGTTGTTTATGGACCTGGTACCGGTGGAACTTTTCA
ACACCCATCTCAGATTCAGGAGTTTTACCACCACGGCGCTTCCACCCTCTCCACGACGCCTTACCAACAGAAT
CCCTGCGCCGTGACCTGCCACGGAGAGCCGGGCAATTTCTATGGATACGACGCTCTGCAAAGGCAAACGCTTT
TCGGGACTCAAGATACAGATCTAGTCCAGTACAGCGACTGCAAGCTGGCAGCGAACAGCATTGGGGAGGAGAC
AGAAAGCACAGAGCAGAGCCCCTCTCCCACACAACTCTTCCCATGGATGAGACCCCAAGTAGCTGCCGGACGG
AGGCGAGGCCGGCAGACCTACAGTCGCTACCAGACCCTGGAGCTGGAGAAGGAGTTTCTGTTTAATCCCTACC
TGACGCGCAAGCGGCGGATCGAGGTATCGCACGCCTTGGGACTGACCGAGCGGCAGGTCAAAATCTGGTTTCA
GAACAGAAGGATGAAGTGGAAAAAGGAGAACAACAAAGACAAATTTCCCAGCAGCAAATCGGAGCAGGAAGAA
ATAGAGAAACAGAAGAAAGAGAAGGACCAAGGCGAGGTGGGGGAGACGGCGGAGGAAGACGGTGACAAAGAGA
AACAAATGTAG
>Aca_hoxB09_scaf2
ATGTCCATTTCTGGAACTCTTAGCAGTTACTATGTTGATTCCATCATAAGTCACGAAAGCGAAGATCCGACTT
CAGCCAAGTTCTCCAATGTACAGTATTCAAGTTCCAGGCAGCCCGGAGGACACAGTGAGCATCCCGAATTTCC
TTCCTGCAGTTTCCAGCCTAAACCTCCTGTTTTCGGCGCGTCCTGGAGTGCTTTCAATCCCCACTCGGCTAAT
GGGTTACCCGCGGTCTACCATCCCTACATCCCGCCTCAGGCTGCCCCCTCCTCGGACACTAGGTACCTGCGCA
CTTGGCTCGATTGTGCCCCGAGAACTGAGCCCCTGGCCGGACAGGGGCAGGTCAAACTAGAGCCACTGCTGGG
ACACCTCGGCGAGCCCCCAAAAACCGGGGGACAGGAGTACAGTTTGGAGCCTTCCTCGGCCAGGGAGATTAAT
TCCAATCAAGGCTCCGGCTTCGAGGACAATAAGGATATTTGTGAAGGCAGCGAGGACAAAGAGGGTCCAGATC

AAACTAACCCCTCTGCCAACTGGTTGCACGCCCGCTCCTCCAGGAAGAAGAGGTGCCCGTACACCAAATACCA
AACCCTGGAGCTCGAGAAGGAGTTCCTCTTCAATATGTACCTCACCAGGGACCGGCGGCACGAAGTGGCCCGC
CTGCTGAACTTGACTGAGAGACAAGTGAAAATCTGGTTCCAGAACCGCAGGATGAAAATGAAGAAAATGAACA
AGGAGCAGCCTAAGGATTAG
>Aca_hoxB10_scaf2
ATGTCATGCTCTGAAAGATCCGTTTTCACTGCTTTTTCTGTTAACTCTTTAATAAGCGGCGACCATAAAGAGC
CCCCCTGCACCACCTATCAACTCGACAGTTTATCTCAGGACTGCCGAGCCCCAAATGACTGGGGATCTGTGCC
AAGTCCAGTCATACCCGGCGAAATGAACCAACACCAGCACCAACACCAGCACCAGCAGTCGGCAATGGCTTTG
CTTCCGCACCAGTTTCACCCGGGCTTGGTAAACTGGGGAGATCACTCCAGCCCCGGCCGGGTGCAGCAGGTCA
GCGCTTGTCCCTATAGCGGCTCAAACGGAAAAGATGATTCCTCGTACTTCTATCTGGACTCGGATAAGCATTC
AAAACCACTGCCAGACGTTTCCGCCTTCACCAGACTCGTGTCCGAGATGGGCTCTATGAGCAGCGCCGCAGGA
CACATCCAGGGCTACTTCCGTCCAGAGCAGTCCTACACTGGGCTCAAGCTGCTCGAGTACAGCGCCCAGGACC
TGTCCCCAGTCAGCCACCCCATCCCGACCCACTCAGATACACAGCCGCTGTACAACCTGAGCTTCCCCTCGCA
GCACTGCACAGACACAGATAGAAAACACGGGGGCGATGACCTAGCAGCAGTCTGCAACTCTGTCTGTCCACCT
CAGAAAGTCAACGGCAGCAGCAGCAGCACCAGCAGCAACAACAACAACAACAACAACAACAACAAAAACAAGG
AAGCTGTGGCCCCAGGTTTACTGCAAGAAAAGCCCCTCTCAGAATTTCCCGAGGGCGATAAAACTCCCAGACA
GGACGCGCAAGAAGGCTCAAAAGGTGAGAATGCAGTAAGCGGCTGGCTGACAGCGAAAGCCGGAAGAAAAAAG
CGGTGCCCTTATTCCAAACACCAAACCCTGGAACTGGAGAAGGAGTTCCTCTTCAATATGTACCTGACTCGAG
AGCGCCGCCTGGAGATCAGCCGTAGCGTCAACCTCACGGACCGCCAGGTCAAAATCTGGTTCCAGAACCGGCG
GATGAAACTGAAAAAAATGACCCGGGAGCACAGGACGAGAGACGCAACTGCGCATTTCTCAATTGGACGCTGA
>Aca_hoxB13_scaf2
ATGACCACCTCCCTGGTCCTTAATCCGCGCTGGGTGGACTCGGTAATGTTTGTATACGAAAACAACTTGGATG
AACTTAACAAGAACATGGAGGGTCTGGTGGGTGCCGGCAATTTCGCAGCCAACCAGTGCAGGAATCTGATGGC
CCATTCCGCCCTGGGAGGCCACCCTTCCTCTCTCGTACACGGCTCCGGCTACTCCGCAGTCGATGTGACCGGG
GCTGGATCCGTAGAAGCGGGAAAGCAATGCGCTCCGTGCCCGGGGGTTCCCCAGGGGTCTTCCGCTGCTCCTC
TGCCATATGGGTATTTCGGCAACGGCTACTACTCCTGCAGAATGGGCAGAGGTGCACTGAAGTCTTGCACCCA
ACCCAGCGCCCTCTCCAGTTATTCCGCAGAGAAATACATGGACACCCCTGTGGCGTCCGAAGAGTACCCTAGC
AGGGCCAAGGAATTCGCCTTCTACCACGGCTATGCCAGTCCCTACCAACCCATGGCCAGCTACCTGGACGTTT
CTGTGGTGCAGACAATCAGTGGGGCCGGGGAGCCCCGGCATGAGACGCTGCTGCCAATGGACAGCTATCAGCC
GTGGGCACTGACCAACGGATGGGGCAGTCAGATGTACTGTTCCAAAGACCAGAGCCAGACTGGACATCTTTGG
AAATCTGCTTTAGCAGACGTAGTGGCCCACCAGCAAGACGGGAGCTCCTTCCGCCGCGGTAGAAAGAAAAGGA
TACCTTACACCAAGGCGCAGCTAAAGGAACTGGAAAAAGAATATGCCGCCAACAAATTCATCACCAAAGACAA
GAGGAGAAAGATTTCTGCAGTAACCAACCTTTCGGAAAGACAGATTACTATATGGTTTCAAAACCGGAGGGTT
AAAGAAAAGAAATTCGTGGCAAAAGTCAAACCCAGCGCTCCTTAA
>Aca_hoxC01_scaf6
ATGAACGCGTACCCAGAGATAGTGTGCGGAGCGGAGCCCGGCGCTCCCTACGCGGGGGGATACCCGGAGGCCC
GGCTTCAGCATCTGGACCAGAGACCTTATTCGAAAGTGAGCTGTGGCGGAGAAGGACGTTACGTGGTGGGCAC
GGATTTCGCCTTGCATCGCCGGGTCTCACCTTTCCCCTGCAGCGGCGCCCTCAACCCCCCTCCGGCTCCCACT
CCCACTCCATCTGCCCCTCAGCATCCCGGGTTCTCCGGCACGAACACTTTCCACGCCGGCAGTCTTCACGTCA
CTTTCAGTCCCGGGACACCTGCTAGAGCCTCCTTCACCCACAGCCAGGGATACCCCTCCCAGCCGGCCTACCT
CCACCATGCCCACCCGCACTCCTGCACAAACTCCCAGGACCTGGATTACACCGGGACTGGGTATCCCGTCAGC
GCCGACCCTCCGGTGACCTTTTCCGTGCTCCACCCCCGGCTTGGGACGCTGGATCATCCCTACCAGTCGTGCG
CTCAGGAAGGGTCGAGGTTACCTCTTCCTGAATGCCCATCGGAGGACGGAGACAACCCCCGGCTGGACACCGG
CTACAGCAGCAAAACATTCGACTGGATGAAAGTGAAGCGAAACCCTCCCAGGATTGCGAAGCCAGTGGCCGCA
GCCTGTGCGGTGAACTACCTCGGTCTAGGCCTCGGTCCAGACGGCGCTGACCCCCCTGGCAGTCTACACGGCA
ACGGGGCGCCGAGGACCAACTTCAGCACCAAGCAGCTGACCGAGCTGGAGAAGGAGTTTCACTTCAACAAGTA
CCTGACGCGGGCCCGGCGGGTGGAGATCGCCACGGCCCTGCAGCTCAACGAGACGCAGGTGAAAATCTGGTTC
CAGAACCGGCGGATGAAGCAGAAGAAAAGGGAGCGGGAGGGCCTGCTGCTCGGGACCCTGGCCTCCTCCGGCG
GCGGCGGCGGCGGCAGCAGCCAGGAGGACTCACCCTCGGACAAGTCTGACAATGGCTCCTCGCCCGCCCCTTC
TCCGCCGCCACATCCAGTCCTACCTCCACCCCAGCCGCTCCCTCCCAACACAATCACTCGCGCCCTTTCGCCC
TCGTTGCAAAGCAAGGGCTGTGATAATGCCCCCGGCACCAGCCCCGACTCCTTGTGA
>Aca_hoxC03_scaf6
ATGCAAAAGACGCTTTATTACGAAAATGCAGGCCCCTTTTTGGGTTACCCTTGCCAAGAACCCGCCGGATTGG
GATTCGAGCAGCTGTACCCAACGTCCTCAGAATACCAGTCTCTCACCTGCTACCTGCAAGACGACACCTCCCC

GTGTGACCCGAGCAGCGACCCCGAGCACAATGTGGGCTCCAGCGGCTGCCACATCCCCGAGCTGCCGGAGCCC
CCACTCCGACACCCCCCTCTCACCCCCTCCCCAGGCGCCAGCTCCACTTCGACCCACAGCAGTCCCTGTAAAA
GCGCCTCGGACCCGCTCGACTGCAGCCCCGGAGCAGAGAAAGCAACCGCTAACGGCAGGACTAGCACGGGCTC
TGCCTTCAAGAGCAACTCACCAAAACAAATCTTTCCGTGGATGAAGGAGTCGCGCCAGAGCTCCAAACAGAAG
CACAACAATTTCATCACACCTGGAGAGTCAGGCAGCAGTGGGGAGTGCAGTCCCCCTGGAGGCGGAGCTGTGG
GCGGGGCTTCAAAGCGCGCCCGCACGGCTTACACCAACGCCCAGCTGGTGGAGCTGGAGAAGGAGTTCCACTT
CAACCGCTACCTGTGCCGGCCGCGGCGGCTGGAGATGGCCGCCCTGCTGCAGCTGTCTGAGCGCCAGATCAAG
ATCTGGTTCCAGAACCGCCGCATGAAGTACAAGAAGGACCACAAGGGCCGGGCTGGGCCGGGCTCCCCGCTTG
GAGGGGGCTCTCCCAGCCGCAGTCCACCTCTAGGAGCACCATACTCGGGGGAGCTGGGCTACGAGACCCCCCT
CTCCCATCCCTACGCCAAAACCCCCGGCCCCATGGTCTACGGATTGGCTGCTTATTCCGCCTCGCCCTATGAC
TGCCCCCCCCCGCTACAGCTGCCGCCTCAGAAGAGGTACGGCGACCCCGCCCCCCTGCCACAGGATTACGACC
AGGTTGACAACAGCTACCCCAGCCCTGGATACGTGGGGGCCAGCTTTGGAGAACCCCCCCCTTCCCACCCAAT
CCCACTCTTCAACCTCCAGCACCTCTCCTCATCCTCTGCCTCCTCTTCCTCCTCTACCTCCTCCTCCTCCTCC
TCCTCCTCTGCCAGCATGGACTACAGCTGCGTGGCCCCCAGCCCCCCCAAGCACTCGCTGGGCCCCTGTGACC
CCCACCTGCCCTACAGCAACCTGGGCTCGCACTGCACGCCTCAGGGCTCCGCTCAGGTACCCCCCACACTCAC
TCACCTGTGA
>Aca_hoxC04_scaf6
ATGATCATGAGCTCGTATTTGATGGACTCTAACTACATCGATCCGAAATTTCCTCCATGCGAGGAATATTCGC
AAAATAGCTACATCCCCGACCACAGCCCCGAATATTACAGCCGAGCCAGGGACACTACCAGCTACCAGCATCA
CCACCAGGACTTGTACCCTCCGCGAGCGAGCTACCAGGACCGCCAGTTTAACTGTGCGAGCATCCCGGAGCCT
GACACTGTGCGAGGACATGGGCTCCCCCACCCCGGGCACCTGCTGGCGGGGAAGGCGCAACCAGCTCCCTGTG
AGCCCCCGCCACTGCCCACGTCCCCCTCTACCCCGCCGGCTGTGCCTTCCGCCTGCACCCAAGCCACACCGGA
GCATCCGAACAACACAACCTCCTCCAAGCAGCCCGTAGTATACCCCTGGATGAAGAAAATCCACGTCAGTACC
GTGAACCCCAATTACAATGGAGCGGAACCCAAACGATCCCGGACTGCATACACCAGACAGCAGGTTTTAGAAC
TGGAGAAAGAGTTTCACTATAACCGCTACCTTACCCGGCGAAGACGCATTGAAATCGCTCACTCCTTGGTTCT
CTCCGAACGGCAGATCAAAATTTGGTTTCAGAACCGGAGAATGAAGTGGAAAAAAGACCACAGGCTTCCCAAC
ACCAAAGTCCGATCGTCGGCTTCCTCGGGCTCCTCTTCCGGCTCAACTACAACCTCAACCCCCGGCGCTGTGG
CATCGGTTGCCGCCGCCACCACCGCTTCCAGTACGGTATCAGCAAGCGAAGAACTCTCCCGGGTACCCAATAC
TGACCGAGGCGAGGATATTACAAGGTTATAA
>Aca_hoxC05_scaf6
ATGAGTTCGTACGTCGCCAACTCGTTTTACAAGCAAACCCATGAGACTCCTGCTTACACTATGCACGGCTATG
GGAACTATGGATCTGTCTCTGAGCTGCACCCGTCCAGGTACTGTTACGGAGGGCTGGACCTCAGCGTCTCCTT
CCCTTCGCCGGCCTCTTCCAACTCCCTCAACCGTGGGGAGATGAGCGCTAGCCTGCGCGGCAGCCCGGACTCG
CAGCCACCGCCGCCTCCTCCTCCTCCACCGCCGCCGCCACCGCCGCCGCCTTGCTCCGCCGTGGGCTCCCCGA
GAAGGGCTCTGAGCGCCGGCGGCCAGGGCTCCCTCTCCGGCGGGCTGTTCTGTCAGAAAGCCGAGGGGGACAT
GGATCTTAACGACAAGCCCGGCAGTAACAGCAGAGCCGGCGAGATCAAAGTGGAAGCAGTCCAGCCGGTAAGA
CAGGCAACCCATGCGACCCAGCCGCAGTCAGACCTCCACCAGCCCCAGATATACCCCTGGATGACCAAGCTTC
ACATAAGCCACGATTCAGACGGGAAGAGGTCCCGGACCAGCTACACCCGCTATCAGACTCTGGAGCTGGAGAA
AGAGTTTCACTTCAACCGCTATCTGACCCGCCGCAGGCGCATCGAGATCGCCAACAGCCTTTGTCTGAACGAG
AGGCAGATCAAAATCTGGTTTCAGAACCGGCGCATGAAGTGGAAGAAGGATTCGAAGCTTAAAGCGAAGGAGA
CCGTTTAG
>Aca_hoxC06_scaf6
ATGAATTCGTATTTCGCAAACCCGTCGCTCTCCTGCCACTTAACCGGTGGACAAGACGTTTTACCCAACGTGG
CCCTAAATTCAACTACCTATGACCCCGTTAGGCACTTCTCGTCGTACGGCGCCGCCGTGGCTCAGAATCGGAT
TTACTCTTCTCCCTTCTATTCGCCCCAAGACAACGTCGTGTTTGGGTCGAGCCGAGGACCGTACGAGTATGGA
TCTAACGTGTTTTACCAAGACAAGGATGTGCTGCCCAGCTGCAGGCAATCCAGCATGGGACAGAGCGCACAGA
GCACCCTCGCCCAGGACTATAGCACGGAGCAAAGCAGGACGGGCACGCAGGAGCAGAAAGGCAGCATTCAGAT
TTACCCCTGGATGCAGCGAATGAACTCTCACAGTGGAGTGGGGTACGGATCTGACAGACGGAGAGGGCGCCAG
ATTTACTCTCGGTACCAAACTCTAGAACTCGAGAAGGAATTCCACTTCAATCGCTACTTGACGCGACGCAGAC
GGATCGAGATCGCGAACGCTCTGTGTCTCACAGAGCGCCAGATCAAAATCTGGTTCCAGAACCGGCGGATGAA
GTGGAAAAAGGAGAGCAACCTGACGTCCACCATCGCGGGGGGTGGGTCGGCAGCGGGATCCCAGGAGGAGGAA
AAAGAAGGGATCGAAGAGGAGGCAGAAGACGAGAAGAAAAAAGAATGA
>Aca_hoxC08_scaf6

ATGAGCTCCTATTTCGTGAACCCTCTTTTCTCCAAATACAAAGCCGGCGAGTCTCTGGAACCAACTTACTACG
ACTGCCGGTTCCCTCAGAGCGTCAGCCGGAGCCACGCGTTGGTTTACGGTCCCGGAGCGGCGCCCACCGGCTT
CCAGCACCCGTCCCACCACGTCCAGGACTTCTTCCACCACGGCACCTCCGGCATCTCCAACCCCGGATACCAG
CAAAGCCCCTGCGCCCTGGCTTGCCACGGGGACGCCACCAAATTCTACGGATACGAAGCCCTTCCGAGGCAGT
CGCTGTATGGCGCACAGCAAGAGCCCAGCGTAGCGCAATATGCAGACTGTAAATCGACTAACGGCGCTAACCC
AGGAGACGGACAAGCCCACTTAAATCAAACTTCGTCTTCTAGTCTCATGTTTCCCTGGATGAGACCACACGCT
CCGGGGAGACGTAGCGGCAGGCAGACTTACAGCCGGTATCAGACCCTGGAGCTGGAGAAGGAGTTTCTCTTCA
ACCCTTACCTGACACGGAAGCGCAGAATCGAGGTGTCCCACGCCCTCGGACTGACGGAGCGGCAGGTGAAGAT
CTGGTTTCAGAACCGGCGGATGAAGTGGAAAAAAGAGAACAATAAAGACAAGTTCCCGGGCCAGAGAGGGGAG
ACCGAGGCCGAGGAGGAAGGCAACGAGGACGGAGAGGCTGAAGAGAAGGAAACGGAAGAGAAAGAGGAGAGCA
AGGAGTGA
>Aca_hoxC09_scaf6
ATGTCGACCACGGGTCCCATAAGCAACTATTATGTGGATTCATTGATAAACCATGAAAGCGAAGAAGTCTTGG
CGGCTGCTCGCTTCTCGGCTCCGGGATCTCTCCCCTCGGGTTCCCGTCCGGCAGGCTTGGTACCAGAGTGTAC
CGATTTCCCCTCTTGCAGCTTTGCCCCAAAACCTCCCATCTTCACGACTTCCTGGGCCCCGGTGCAGTCCCAG
TCCTCAGTGGTGTATCACCCCTACACGCATCAAACCCACTTAGGGACAGACTCCAGGTACGTGCGGTCTTGGC
TGGAGCCTATCTCAGGCGCCGTGTCCTTCACCGGCTTCCCGGCCAACAGCAGACACTACGGGCTGAAACCCGA
CGCGTTCCCGGAGCGCAGGGTCGGGGATTGCCTGCCTCCCAACGCACGAAACTACTCGGATTATCTCTACGGG
TCTTCGGCTGACATTCGCGACAGGACGGCGCAAAACATCCCCTCTCCGGAGTCCGAGGTTTTAGCTTCCAGCA
AACACAAAGAGGAGAAGCCAGAATTAGACCCGAACAATCCGGTTGCTAACTGGATCCACGCCCGCTCCACGAG
AAAGAAGCGGTGCCCTTATACCAAATACCAGACTCTCGAACTGGAGAAGGAGTTCTTATTCAATATGTATCTT
ACCAGGGACCGGCGCTACGAGGTGGCCCGCGTCCTCAACCTCACGGAACGCCAAGTCAAAATCTGGTTCCAGA
ACCGGAGGATGAAAATGAAGAAAATGAATAAAGAGAAGACCGACAGCAACGAACAATAA
>Aca_hoxC10_scaf6
ATGTCATGTCCCAACAACGTGGCGGCGGGTTCCTTTCTCATGGATTCTCTCATGGGCTCCAACGCGTACCGAG
GTGAGGGCTACACGGCCAGCCCGGGGATTTACATGCAAACGGGCGCTGAGTATGGCTGTGCAGTGATGAGAAA
CTGTGGGATTGTACCATCCCCTCTCTCCAAAAGAGACGAGATCAGCCCGGCCAACCTGCCGCTCAGCGCCTAC
CACCACCCGTCTTACCTGCCGCAGCTGGACACCTGGGGGGCAGACCCTAAAAGTTCCTGCAGGATCGAGCAAC
CTGTTGCCAGACCTTTGCCCTCCTGCTCCTTCCCAGCCGCCAATGTCAAAGAGGAGGCGATGTGCTGCATGTA
CAGAGCAGACATCAGCGGGGCGAAGGAGGCTGCCGAACCGACCACCTACACCCGACTTGGGGCGGGGAGCAGC
CAGCCCGAGCGCGCCGTGCCAGTGCCGGGCTACTTCCGAGTCGCCCAGGGTTACCCGGGGGAGAAGGCGCAAG
ACGCGGACGAGTTCGGCCCCGGTTTCGCCTCGGTGGTGAGCAGTTTGACCCCTCCAGTTGACACCAGCGGGAC
GCCTCCACTGCCCACCGCCGAATCCAAGCCAGAACAGCAGACAGACACCGCCGAGATCAAGGACGAGGACCCC
ACCGCGGCGAAGAGATTATCCGAGCTGGACAGGGAGAGCTACCCCAAGACAGACAGCAGTACAGACGCCTCGG
ACACCGAAGTCAAAGAGGACTTGAAAGCCGACAAGGCCACAGGAAGCTGGCTGACAGCGAAGAGCGGAAGAAA
GAAGAGATGTCCTTACACAAAGCACCAGACTCTCGAACTGGAGAAGGAGTTCTTGTTTAACATGTATCTGACT
CGTGAGCGCCGCCTGGAGATCAGCAGGAGCATCAACCTCACCGACAGACAAGTGAAGATCTGGTTTCAGAACC
GGAGGATGAAACTGAAGAAACTCAATCGAGAGAGCCGCGTCCGGGAGATCACTTCGACCTACAACTTCACCTG
A
>Aca_hoxC11_scaf6
ATGTTTAACTCGGTCAATCTGGGCAACTTCTGCTCCCAGACTCGCAAAGACAGGACTTCCGACTTTGGGGACA
GAGCGGCTTGTGCCTCCAACCTCTACCTCCCCAGCTGCACCTATTACGTCCCCGAGTTTTCCGCGGTCTCCTC
GTTTCTGCCACAGGCCCCGTCTCGACAGATCACCTACCCGTATTCCACAAACCTGACTCAAGTGCAGCCGGTC
CGGGAGGTTTCCTACGGCTTGGACCCTTCCAGCAAGTGGCATCACCGAAGCAACTATGCGTCCTGCTATTCAG
GAGAGGATCTGGTGCATAGGGATTGCCTCCCGCCCTCCACCATGACCGAAATGCTCATGAAAAACGAGAGCGT
GTACAGCCACCACCACCACCACCACCACCCCGGCACCAACCACCCTTCCTCGGGCTTCTACTCCAGCGTGGGG
AAGAACAACGTCCTCCCGCAGGGTTTCGATCGCTTTTTCGAGGGCGCCTACTGCAGCGCCGACAGCCAGCCCG
AGAACTGTTTACAAAAGAGCGAGGCGAGCAAGCTGGAGCCGGAGACGCAGGCTAGCGAGCTCCCCGGCGCCGC
AGACCCGGAAAAGGACCCGGAGGATGAGGAGGAAAACACAAACTCGGGCTCATGCACCTCTTCCTCAGGCACG
AAGGACGGCAGCGGCAGCAAGAACAACCACTCGAGTACCCCTCGCACACGGAAGAAGAGGTGTCCCTATTCCA
AGTTTCAGATCCGAGAACTGGAGCGGGAGTTCTTTTTTAACGTTTACATCAACAAGGAGAAGCGGCTCCAGTT
GTCCAGGATGCTGAACCTCACCGACCGACAGGTCAAGATCTGGTTCCAGAACCGGAGAATGAAAGAAAAGAAA
TTGAGCCGAGACCGCTTGCAGTACTTCTCAGGCAACCCTTTATTATGA
>Aca_hoxC12_scaf6

ATGGGCGAGCATAATCTTCTTAATCCCGGGTTTGTGGGACCTTTGGTAAACATCCACACGGGAGACACCTTCT
ATTTCCCTAATTTTAGAGCATCCGGGGGACAGCTGGCGGGTCTACCGTCTCTCTCCTATCCGAGAAGGGACAA
TGTTTGCTCCCTCCCCTGGAACCCCTCGGAGCCGTGCAATGGATACCCTCAATCCTACCTTAGCAGCCCCATG
TCCATTAACCCTTCCTTCAACCGTGGCTGCGATATAACCCGGCCGGAGGAAGGCAAATGTTACTACAGCAACA
GTGGTGGCGGTGGCGGCGGCGTCAGGGAGTCTTGCTCGGAGAATGGGAGCCTTAAAAGAGAGGACAGGGTGAG
AGACAGTACTTCCATAGCGTCCGAACACGGGCTGCACAACGGTATGGGCAGCGGCGGCGGTACCTTCTCCAAA
TATGACTATGGAGTCGAGCCGCTGACCCAAGACCCGCCGTCCTGTCAGTCTCTCGAATCCGACTCCAGCTCCT
CGCTGCTCAACGAGGGAGGCAAGACCCCGGCCAGCGACCCCCAGACACTGGCATCGCCGGGAAACCACGCAGC
CAGCATGGCCACCGGTGGGGGCGCCCCGTGGTACCCGATGCACACCCGGACGCGCAAGAAACGTAAACCCTAC
TCCAAGCTGCAGCTCGCCGAGCTGGAAGGGGAGTTCATGCTCAACGAGTTCATCACACGGCAGCGGCGCAGGG
AACTGTCCGACCGGCTCAACCTCAGCGACCAGCAGGTGAAGATCTGGTTCCAGAACCGGAGGATGAAGAAGAA
AAGACTGCTGCTGAGGGAGCAAGCGCTGTCCTTCTTTTAG
>Aca_hoxC13_scaf6
ATGACGACTTCGCTGGTTCTGCATCCACGCTGGGCGGACACCTTGATGTACGTGTATGAAAAAAGCCCGAATG
AAAATAACCAGAATAAAAGCCCAGCTATGGAGGGACTGAGCGGGAATTGCCCCGCGAGCCATTGCAGGGATTT
GATCTCGCACCCAGCTCTGGGGCGGCATTCCGGCAGCATAGCGACCCACCAGGGCTCCGTGTACTCGGATATT
TCCTCGCCAGACGCCGGCAGACAGTGCCCCGCTCCCCAAACTTCATCTAGCGCGACCCTGGGCTACGGCTACC
CCTTTGGAAGCCCATATTACGGGTGCAGGTTGTCTCACTCGCACAACGTGAACTTGCAGCAGAAGCCCTGCTC
CTACCACCCGGCAGAGAAATATGCTGAGCCCAGCAGCGCGCTACCCTCGGAAGAACTGTCCAGCAGGGCCAAA
GAATTCGCCTTTTACCCCAGTTTTGCCAGCTCTTACCAGGCTGTCCCGGGCTATTTAGACGTGTCAGTGGTTC
CCAGTATCAGTGCGCACCCGGAACCGCGGCACGATGCCTTGATTCCCATGGAAGGGTATCAACATTGGGCGCT
GTCTAATGGCTGGGACGGGCAGGTGTACTGTTCCAAGGAGCAAACACAGTCAACTCACCTCTGGAAGTCCCCG
TTCCCAGATGTGGTACCCTTGCAGCCGGAGGTGAGCAGTTACCGCCGCGGCCGGAAGAAGCGGGTGCCCTACA
CCAAGATCCAGCTGAAGGAGTTGGAGAAAGAGTACGCGGCCAGCAAGTTCATTACCAAAGACAAGCGACGGCG
CATCTCGGCCACCACCAACCTCTCCGAGCGCCAGGTCACCATCTGGTTCCAGAACCGCCGGGTCAAAGAAAAG
AAGTTCGTCAGCAAATCGAAGAACAGCACGCACATGCACGCCACTTGA
>Aca_hoxD01_scaf17
ATGAATACGTACTTTGACTACTTTTCCAGCGGAGACGTGTTAGCTTTGCCTGCAAAATGTTGCCATGCTGAGC
ACAGGGCTGTCCCTCTGCAGTACTCAGTGACTGGCGACACTATGGGGAGACTGCCAATAGGTATCAATGCCCT
GGATCATCCTTCAAACACCCGGGCAGTTTCTCCTGGGATGCATCCTCCAAGTACATTGGAAGTACCTTACGAG
AACAGCACGAACAGCGCCGAGTTAAACTATGTGGCCCAAGGCATAGGTTATGATTTACCGTACGGATGTCCTA
ACGACACAGAAGACAGCGGGGCACATGTCCAGTACATCACATCTGTATACCCGGGACATGGATCGTATCCACT
CAATCACGGAGAGCCCTCTTTCAACGATCTTGGCGAGGAAGACCAATACAAGCACAACAACAAGACATTGCGT
GGGTTCTACTCGGGAAGTTACCCCAACCTTTCATCTTCTCAAGTGAGTTACCCAAAGTCCACACCAATGCAAG
AAGAGACCCAAAACACTGCAAATACCTTCGAGTGGATGAAGATCAAACGAAATAATCCCAAAACCAGTAAAGC
ACTCGAGTATGGGATTCCGACCTCAGCTGCAACAGCTCGAACAAATTTCACCACCAAACAACTCACCGAGCTT
GAGAAGGAATTTCATTTCAATAAATATCTAACCAGGTCAAGGAGGGTGGAGATAGCCAATACGCTGCAGCTAA
ACGAGACCCAGGTGAAAATCTGGTTTCAGAACAGAAGAATGAAGCAAAAAAAGAGAGAGAGGGAAGGCCTTGC
CACTGTGCCTCCGCCTCGCGTTTCCAAAAACTCGGACACGTCTTCTTCAGAAAACAATTCTCCATCCTCGTCT
CCATCGTGTTCCCCACAAACATCTGTATGTTAG
>Aca_hoxD02_scaf17
ATGAGCAAAGTGTTTGAGACAGAGGTTGGATTTATTAACAGTGAGCCATCCTTAGCGGAGTGCCTTACATCTC
TTCCTCCTCTTGGCGAGACATTTCAAAATTCATCAATCAAGAACTCGTTGCTTTCAGATTCGACACTGATTCC
TCCTCCTTGTGAACAAGGCTTTAGCATCCTGAACTCTGGAAACACATTTCAAAATGAGAGGAGCCAAACTTCG
AAAGATATTGACAGCGCAGCCTTAGGCAGCAAACCTCTGGAATACCCATGGATGAAGGAGAAGAAAAATAAAG
GGAAACACGCTGCTATACCGGGTACCTCTTCCTCTGGATCTTCAAATGAAGATAACGAAGACTTTCAGGATAC
CCTCGGTGGTAGTCCTCTTCACAGAAGACTCCGCACAACATACACTAATACTCAGCTATTGGAATTAGAGAAG
GAGTTTCATTATAACAGGTACCTTTGCAGACCAAGGAGAATAGAAATTGCATCTCTTCTAGATTTAACTGAAA
GACAAGTCAAAGTCTGGTTTCAAAACAGACGAATGAAGCATAAAAGACAATCCCGGTTCAAACAAGGGAAAAT
ACTTGATTCCAAAGGCATTGTAAGCAGTGGTTGTGTCAACAGTGTCTTTTATAGTGGGCAATCACAGACAATC
CTGGACAAGGTTGCTTTAGAGGAGACAGATTTTGAGCAAACCGAAAAGGATGGACCACACCAAGAAAAGATAC
TGATGGAACTGTCTTCGTTTTCTGGAAACCAAAGTGATGACGAGTTTCTCGTTTCCAGCGACAAAAGTTGCTC
TTTGGCAAACTTGAAGACCTTGTCATTTGCTGTGGACAGTTTCAATATACTTTCTTTGGAGGATTTAGATACA

ATTTCAATCGACCTTTTCACAGCAGAACCCTCATTTTCATTAACTGAAACCGACACAACCAATAACGATGTAG
ATGCGTTTAATATTTTAATTGAAAATATTTGTACAACAGATTTCCAACAGCTGCAATTTTAA
>Aca_hoxD03_scaf17
ATGCAGAAAGCAGCATACTATGACAATACAGGGTTGTTTGGAGGCTATTCATACCCTAAAACTGATTCATATA
GTTACAGCTCCACTCACCAGCCGTACCCAGCATCTAATATTGAGAACGATTTTCAGGGTCCAGTTTGTACTAT
ACAAACACCAACTATTAGACCCCCCAGTCATAAAACTACTGACATGAATGGCAGCTGTATGCGAACAAGTAAC
AATCAAGGTAGCAGCCAACCAGCCAGCATCAGTGATCAACCACAAGCACCTCCTTTGCCAGCTTCTTCACCGA
ACTCCAACAGCACTCCAGCCCAGAAAAAAAAGTCTGCTTCTAATACTTCTAATTCTGCCACTCCGGTTCTTAC
AAAGCAAATATTCCCATGGATGAAGGAGTCCCGGCAGAACTCAAAGCAGAAAAACAACTGCACAACTCCAGGT
GACACGTGTGATGACAAGAGCCCACCAGGTCCAGCCTCGAAGAGAGTCCGCACAGCCTACACCAGCGCCCAAC
TGGTCGAGCTGGAGAAGGAGTTCCACTTCAACCGGTACCTCTGCCGTCCCCGCAGAGTGGAGATGGCCAACCT
ATTGAATCTGACAGAGCGCCAAATAAAGATATGGTTTCAAAACCGGAGGATGAAGTACAAAAAGGATCAAAAA
GCCAAGGGGATAATGCACTCTCCAGTAGGACATTCCCCCGACAGAAGCCCTCCTTTAAGTGGCCCGAACCATA
TCGGATATTCCAGTCAGCTTCCCAATGTAAACAGTCTCAACTACGACGCTCCCTCGCCCACGTCGTTTGCTAA
GCCACAGCAAAACATGTACGGCTTGGCTGCGTACACGGCGCCATTAGGTGGCTGCATACCCCAGCAGAAAAGG
TACCCGGGGACAGAATACGAACACCACAGCATGCAAGGCAATGGTGGCTTTACCAATGCTAATTTACAAGGCA
GCCCCGTGTATGTTGGAGGGAATTTCGTTGATTCCATGCCAGCGTCGGGTCCCATGTTCAACCTCGGCCATCT
CCCCCATCCTTCATCTGCCAGCGTGGACTACAGCTGCGCCGCTCAGATCCCGGGCAACCATCACCATGGACCT
TGTGACCCCCATCCCACATACACAGATCTAACTTCTCACCACACATCTCAGGGACGGATTCAGGAAGCACCTA
AACTAACGCATCTGTAG
>Aca_hoxD04_scaf17
ATGGCCATGAGTTCGTATATGGTGAACTCCAAGTATGTGGATCCCAAATTTCCTCCTTGTGAGGAATACTCGC
AGAACAGCTATATACCTGACCAGGGCCCGGGGTATTACAGCCCACCGCAGGACCCTGATTTTCAGCATCCAGG
CATCTATGCTCGGTCAAACTACACCGAGCAGCCTTTCAGCTGTAGCACTGAGCCGGGCTCGACAGTGCAGCCG
CGGGGTCATGTGCAGGAGCACCCCGGCCAGCAGAGCCACTTCCCCGCCCCGGCTGAGCAGTGCCCACCGGTCC
AACTGGCCGGCCCTCGGTCCTGCGGCCAGCAGCCAAACACCAAGACCCAAAACGGGATACAAGCCAAGCAACC
AGCAATAGTTTATCCCTGGATGAAGAAAGTCCACGTTACAACGGTAAATCCAGATTTCACCGGAGGGGAACCC
AAACGATCAAGGACAGCGTATACAAGGCAGCAGGTTTTGGAACTGGAGAAAGAATTTCATTTTAACAGGTATC
TGACCAGGCGGCGTCGTATTGAAATTGCGCACACTCTGTGTCTCTCTGAGCGACAGATCAAAATCTGGTTTCA
GAACAGGAGGATGAAATGGAAAAAAGACCACAAACTTCCCAACACGAAGGGCAGGTCGTCGTCTGCCTCTAGT
CAGCATTTACAGACTGTGCAAAAGGACAACCAGACTGAGATCACAACTTTATAA
>Aca_hoxD08_scaf17
ATGAGCTCTTATTTTGTAAATCCATTTTATTCCAAATACAAGCCGGGGGAAACCATCGTCCCAGCGTACTACG
ACTCTCCCTTTGCACAAGATGTGAGCAGTAGACATGCGATGGTGTATGGCAGCAGTGCCAGTTTCCAGCACCC
AGCACCAGTCCAGGACTTTTACCATCATGGGAGCGCAAGTTATCAGCCGGATCCGTGTGGGATTGCGTGTCAT
AGCGACCCCTCCAAGTTTTACGGGTACGATAACTTACAGAGACAGCAGCTCTTTACGACCCAGCCAGAGGCCG
AGCTGCTACAATACCCCGACTGTAAATCGTTCGCTGGTAATACTGGCGAGGGACCAGAGCACTTAAATCAGGA
CTCGTCTCCTTCTCAGATGTTTCCCTGGATGAGACCTCAAGCTCCTGGCCGCAGAAGAGGAAGACAGACCTAT
AGTCGGTTCCAGACTCTGGAGCTGGAGAAAGAGTTTCTTTTTAACCCTTATCTGACCAGAAAGCGACGCATCG
AGGTGTCCCATGCGCTGGCACTGACGGAGAGACAGGTCAAAATCTGGTTTCAAAACAGGAGGATGAAATGGAA
AAAGGAAAACAACAAAGACAAATTCCCGGCGTCACGTCCAGAGGAAGAAGCAGAAATTAAAATGGAAGCAGAG
AGCTTGGAGCAAGAAAGGGAGGGGGACGACTCGGATTGA
>Aca_hoxD09_scaf17
ATGTCCAGCAGTGGCACACTTAGTAACTATTATGTGGATTCAATAATAGGACACGAAGCAGAGGACGTGTATG
GAGCCCGCTTTGTTCAGGCAGGACACAGTACGACCTCCAGGCCATCAGGTGTGGGAGATAACGCGGATTTTTC
CTCCTGCAGCTTTGCACCCAAGTCCGCCGTCTTCTCCGCGTCTTGGTCCCCGGTGCACCCTCAGTCCACCGCC
GCAGTGTCCGGGATTTACCACCCCTATGTGCACCAGTCCCACCTTGCGGCCACCGACAACAGATACGTCCGAT
CCTGGATAGACCCCCTCTCCAACTCGGTCTCTTTTTCCGGCTTTCACCCCAACAGTAGGCATTTCGGGACAAA
GCCGGAAAGTTTGCCCCCCAAAAGGACTGAGTGCGCTTCTTTTGACGCACAGACTCCCAACCTCCCCGACTTC
AGCGGCGGGACATTTCCAGAGAGCAAGGAGAAAGCCACCAAGGAGCCGGTGGGCGAGGATGTCTCCAGTCCCA
GCGAGCCCATGGAGGAGAAGCAACAGCAACTTGACCCAAGCAACCCGGCAGCAAACTGGATCCATGCACGTTC
AACGAGGAAGAAACGGTGCCCCTACACCAAATATCAGACACTAGAACTGGAGAAAGAGTTTCTTTATAACATG
TACCTCACCAGAGACCGCCGATACGAAGTGGCTAGGATTTTGAATTTAACCGAAAGGCAGGTAAAAATATGGT
TTCAGAACAGAAGGATGAAAATGAAAAAGATGAACCGAGAGAAAGGCAGTAGGAGCACCAATGACTAA

>Aca_hoxD10_scaf17
ATGTCCTTTCCCAACAGCTCTCCCGCTGCCAGTACCTTTTTAGTAGACTCCTTGATCAGTGCTTGCAGGACTG
ACAGTTTCTATTCCAGCAGCAATATGTACATGCCATCCAGCACAGAAATGGGAAGTTATGGGATGCAAACCTG
TGGACTTCTGCCTTCCCTCGGCAAAAGAGGAGAAGTTAGCCACCAAAATATGGGCATGAATGTCCATTCGTAT
ATACCTCAGATAGATAACTGGGCAGATCCGAGCAGATCGTGCAGAATAGACCAACCAGCCACCCAGCCGATGC
TCACTTGCACTTTTCCATCCAACATAAAAGAAGAAAGTAACTGCTGCATGTATTCCGACAAGAGAGCAAAGGT
CAACTCTGCCGACATGCCCGCTTATCCGAGCCTGATTCCCGAACCGTGCCCGGTGGAGAGTCCCGAAATCCCC
GTCCCGGGATATTTCAGACTGAGCCAGACTTATGCATCTGGGAAAAATCAAGAGTTCAGTCATGCATCTGAAA
CGAGTTCAAACACAATGCTGCAGTTGAGCAGAGTCCATCCCAAATCCCTTGCTGAGGTGGAAAAGAAAATAAT
TGAAAATCCGAGTAACCGAGAGCCTGCCAAGACCCCCAGTCCTGTAGAAAGCCCAGAGCCAAAGTCCAGTTCG
CAGGAGGACAAGCATGGCTCTACTGAAGCGTCTGCCTCCAGCCCAGACGTGCCAGACAAGGACGCTAAAGATA
GTAAAAGCGACGCCCCAACAAGCAATTGGTTAACTGCAAAAAGTGGCAGGAAAAAAAGATGCCCTTACACAAA
GCACCAGACACTGGAATTAGAGAAGGAGTTTTTATTCAACATGTACCTGACTCGAGAGCGCCGCCTAGAGATC
AGCAAGAGCGTCAACCTCACCGACAGACAGGTCAAGATATGGTTTCAAAACCGCAGGATGAAGCTGAAGAAGA
TGAGCCGGGAGAACCGCATCCGTGAACTCACCTCCAACCTCACCTTCTCCTGA
>Aca_hoxD11_scaf17
ATGACGGAGTGTGATGATCGCAGCAACTGTGCGTCAAATATGTATCTGCCCAGCTGTACATATTATGTCTCAG
CACCCGATTTCTCGTCGGTGTCATCGTTTTTACCTCAAACTACTTCGTGCCAAATCAACTTTCCTTATTCTTC
CAATATAACACAAGTCCAACCTGTGCGGGAAGTGGCTTTCAGGGACTATGGTCTGGATCATCCAAGCAAATGG
CACTACAGAGGCAATTACGCTTCCTATTATTCACCAGACGAGATCATGCACAGGGATCTGATCCAGTCATCGA
GCAGAACGGAAATGATCTTCAAAAATGACTACCACCACGGCAGCTCAAATCCGTCATGCAATTTATTCACGAA
TGTGGGCAGGAATGGCATTCTTCCGCAAGGATTCGACCAGTTCTTTGAGACTGGGAACGGCAATGCAGAAAAG
TCCAACTCTGAGCACATGCGGGAGAAGGCAGAGTCCAGTTTCCCCGCAGATGCTGCCTGTGACAAACTGTCTC
CAGACACTTCAGACCAGGAGAAACAAACGCCAACTGAAAACACATTGGAGGATTCCCCTTCATCCCACTGCGC
CGAGGAGAAGCGCACCGGCTCAAGTTCCTCGAAGTCAAGAAAAAAGAGATGCCCCTATTCCAAATTTCAGATT
CGAGAACTGGAACGGGAGTTCTTTTTCAACGTGTACATAAACAAGGAGAAGCGCTTGCAGCTGTCCAGGATGT
TAAACCTGACAGATCGACAAGTGAAAATCTGGTTCCAGAATCGAAGAATGAAAGAGAAAAAGCTGAACAGGGA
TCGGCTTCAGTATTTCACCGGGAATCCCTTATTCTGA
>Aca_hoxD12_scaf17
ATGTGTGAGCGCAATCTCCTCAATTCCGGCTATGTTGGTTCTCTGTTGAATTTCCCCCCTCCGGACTCCTTTT
ATTTCCCCAATCTGCGGGGCAATGGGGCGCAGCTTGCCGGGCTCCCCCCGATCTCTTACGGCCGGAGAGAGGT
GTGCTCGCTCCCGTGGACTTCCCCAAGTTCGTGCGCATCTCCGCCACAGAGCCGCGCCTTTAGCGGGTACCCT
GCACCCGTCCCGGGCAGCTCAGTGCCTGCAAGTGCCAGTGCTCACAACCACAGCAAGGGACCACTGGAAGAAG
CCACTAAATACTACTTCCAGGACGCCAACCTCAAACCCGAGGAACGGTGTCGACCAGCGCCATCTTTTGCCGG
GGATCACGGAGATACCAGTCCTGCCAGCTCCACCAAATATGAGTTCCCTAACCTGGACAGAGAGTCGCACAGC
TCGCCTGCCCATGCTGAACTGAATAGCAGTAGCCAAGCAGTCACCGAAGGCATCAAGCAATCTGTCAACTTAA
ATATAAGCATGCAGCCATCATCCAGCCATGCCTGTAGCAGAGCATCCCTATCTGATGGCTTGCCTTGGTGTCC
TTCACAAATGAGGTCCAGAAAGAAACGGAAGCCATACACCAAACAACAGATCGCTGAACTGGAGAGCGCATTT
CTGATGCACGAATTCATAAACAGACAGAAGAGGAAGGAGCTGTCGGACAGGCTGGACTTGAGCGACCAGCAAG
TCAAAATCTGGTTTCAAAACCGACGCATGAAAAAGAAAAGACTAATGATGCGGGAGCATGCTCTCTCCATGTA
CTAG
>Aca_hoxD13_scaf17
ATGGAGGGTTTGGGAGGGGATATCCCCGCAGCCCAGTGCAGGAGTTTTTTTCCTACTGCTTTTGGGGCTCATG
CCAGCCGAACTGCACCAGGGTCACCAGTCTATCCCATTGCAGACAGAGCAACCTCCCTCGGCCCAGATGCAAT
CAAACCATACACAGCTCTTCCATGCTCTGCAGCCTCCGCAAACACGTCCATGGGCTGTGGATGTCATTTTGGG
AACTCTTACTATGGTTGCAAAATCCCACACAGTACCGGATTCCAGCCGAACGCGATGGAACAAACTGCACATT
CTTCTCTGGGTGGACATTCAGTGGATACATATATGGATATTTCAGGTCGAACAAGTGGTAATGTCCACTGTAA
CGAGATGTCCGGAAGAGCAAAAGAGTTTACTGTTTACCAAGGATATACAGGTTCCTATACACGGATTCCGGGC
TATATCGATGTACCTGTAGTACCTAGAACTGCTACTGGTGACCCGAGGCATGAGGCTGTTAGATCAATCGAGG
GCTACCAACCTTGGACACTGTCAAATAGCTGGAACAGCCAACTTTACTGTACCAAAGAACAAACACACAACTC
TCACATCTGGAAGTCGTCATTTACAGGTGAGATCATGCTAAACCAGCCCGAGATGTGTGCTTACCGGCGGGGG
AGGAAGAAGAGGGTACCTTACACCAAGCTGCAACTCAAGGAGCTTGAACGAGAATACAACAGGAACTCATTTA
TTACCAAAGAGAAAAGGCGTCGGATAGCGACGACAACCAACCTCTCTGAAAGACAAGTCACTATTTGGTTTCA
AAATCGCAGGGTTAAAGACAAGAAAATCGTATCAAAAATTAAAGACGTTGAAAAATATTAG
