# Supplementary material for: The bowfin genome illuminates the developmental evolution of ray-finned fishes
Source: Nat Genet. 2021 Aug 30;53(9):1373–84. doi: 10.1038/s41588-021-00914-y (PMC8423624; doi:10.1038/s41588-021-00914-y)
Supplement: Supplementary file 3 — Peer Review Information [file 41588_2021_914_MOESM3_ESM.pdf]

natureresearch

Peer Review Information

Journal: Nature Genetics
Manuscript Title: The bowfin genome illuminates the developmental evolution of ray-finned fishes
Corresponding author name(s): Dr Ingo Braasch

Reviewer Comments & Decisions:

Decision Letter, initial version:

18th Nov 2020

Dear Ingo,

Your Article, "The genome of the bowfin (Amia calva) illuminates the developmental evolution of ray-finned fishes" has now been seen by 3 referees. You will see from their comments below that while they find your work of interest, some important points are raised.

Reviewer #1 thinks this is great work and only has minor comments.
Reviewer #2 has a longer list of suggestions but also has a favorable opinion, saying that the work is well done overall and that bowfin is an important missing link in the field of fish genomics.
Reviewer #3 is equally positive about this work since it provides a high-quality reference genome for an important species/group and also since the findings on the monophyly of holostean fishes are interesting. Their comments are thoughtful and constructive. Of note, please make sure that the complete dataset is fully accessible.

We are interested in the possibility of publishing your study in Nature Genetics, but would like to consider your response to these concerns in the form of a revised manuscript before we make a final decision on publication.

We therefore invite you to revise your manuscript taking into account all reviewer comments. Please highlight all changes in the manuscript text file. At this stage we will need you to upload a copy of the manuscript in MS Word .docx or similar editable format.

We are committed to providing a fair and constructive peer-review process. Do not hesitate to contact us if there are specific requests from the reviewers that you believe are technically impossible or unlikely to yield a meaningful outcome.

When revising your manuscript:

*1) Include a “Response to referees” document detailing, point-by-point, how you addressed each referee comment. If no action was taken to address a point, you must provide a compelling argument. This response will be sent back to the referees along with the revised manuscript.

*2) If you have not done so already please begin to revise your manuscript so that it conforms to our Article format instructions, available
here.
Refer also to any guidelines provided in this letter.

*3) Include a revised version of any required Reporting Summary:
https://www.nature.com/documents/nr-reporting-summary.pdf
It will be available to referees (and, potentially, statisticians) to aid in their evaluation if the manuscript goes back for peer review.
A revised checklist is essential for re-review of the paper.

Please be aware of our guidelines on digital image standards.

Please use the link below to submit your revised manuscript and related files:

[REDACTED]

Note: This URL links to your confidential home page and associated information about manuscripts you may have submitted, or that you are reviewing for us. If you wish to forward this email to co-authors, please delete the link to your homepage.

Please do not hesitate to contact me if you have any questions or would like to discuss these revisions further.

Nature Genetics is committed to improving transparency in authorship. As part of our efforts in this direction, we are now requesting that all authors identified as ‘corresponding author’ on published papers create and link their Open Researcher and Contributor Identifier (ORCID) with their account on the Manuscript Tracking System (MTS), prior to acceptance. ORCID helps the scientific community achieve unambiguous attribution of all scholarly contributions. You can create and link your ORCID from the home page of the MTS by clicking on ‘Modify my Springer Nature account’. For more information please visit please visit www.springernature.com/orcid.

We look forward to seeing the revised manuscript and thank you for the opportunity to review your work.

Sincerely,

Tiago

Tiago Faial, PhD
Senior Editor
Nature Genetics
https://orcid.org/0000-0003-0864-1200

Reviewers' Comments:

Reviewer #1:
Remarks to the Author:
NG-A56077 entitled "The genome of the bowfin (Amia calva) illuminates the developmental evolution of ray-finned fishes" describes the genome sequencing and the evolutionary and developmental analysis of bowfin, Amia calva. I think this paper will be a milestone for the genome analysis of ray-finned fish. The findings described in this manuscript are the last missing stone for the effects or impacts of the teleost-specific whole-genome duplication in ray-finned fish evolution. The manuscript is presented clearly and all data presented here are very interesting and impressive for readers of the evolutionary and developmental biology field. Analyses of the chromatin accessibility by ATAC-seq presented this manuscript are more attractive. This is new data and has different dimensional analysis comparing with the spotted gar (Lepisosteus oculatus) paper previously described by the same author. I have no major comments on this manuscript but some minor comments or suggestions.

Minor Comments
1. One important question on the ray-finned fish genome is long conserved synteny among ray-finned fish genomes. I think there are two possible explanations of this conserved synteny. One possible explanation is an effect of high fecundity (egg number) in the ray-finned fish. Most individuals with inter-chromosomal rearrangements or fission will mate with individuals that have normal chromosomes. F1 fish and descendants have deleterious effects with chromosomal trisomy or monosomy and individuals with arranged chromosomes will disappear from the population. Another possible explanation is long distance enhancers within the same chromosome. Enhancers and promoters cannot interact each other after chromosome fission and then disappear from the population. Authors did Hi-C analysis in this manuscript. I ask for analysis of the long-distance enhancers for the conserved synteny comparing with Hi-C data of mammalian genomes.
2. Authors identified 163,771 open chromatin regions(OCRs) in bowfin genome and described that "Orthologs of 61% of human enhancers (600/989) were found in bowfin and over half (52%; 314/600) overlap with bowfin ncOCRs. In contrast, orthology of only 45% (449/989) of human enhancers could be established in the zebrafish genome (Supplementary Table 18), illustrating the usefulness of the slowly evolving and 'unduplicated' bowfin genome to connect accessible, non-coding, regulatory regions from human to fish.". This is interesting but I am also interested in sequence conservation of ncOCRs among ray-finned fish. 51 fish genomes from the Ensembl Compara database are available. I ask for an analysis of conserved ncOCRs among bowfin, ray-finned fish, and human or mammals' genomes. The ncOCRs specific to fish genomes will "shed light on the evolution and development of ray-finned fishes" specific phenotypes.

Reviewer #2:
Remarks to the Author:
The authors reported a chromosome-level bowfin (Amia calva) genome assembly with 23 chromosomes and shed light on the evolution and development of ray-finned fishes and bony vertebrates in general. This is an interesting and valuable manuscript that generated high quality genome and comparative analyses for bowfin, which is a unique and important model in many studies across ray-finned fishes and bony vertebrates. Despite the considerable efforts made on generating and analyzing the data, still some details are missing and should be reported. Here, I provide suggestions on further improvement of their analyses and presentation of the results.

Major comments:
1. As an important species in the evolution and development of vertebrates, the genome assembly is of vital importance. As described in the manuscript, the bowfin genome assembly is of high quality in genome accuracy and integrity. However, there is no evidence on it. For example, some basic figures, like diagram of chromosome interactions, short-read mapping ratio, transcript mapping ratio, and the short-read depth results, are missing and should be specifically described and added.
2. The authors introduced a lot about the phylogenetic relationship, which is in fact one of the most fascinating point for bowfin research, but only one phylogenetic tree was constructed with few proofs. I recommend a detailed reconstruction of the phylogenetic relationships, including but not limited to the construction of gene tree and species tree, with the non-coding regions. Besides, except for NJ, more models, including Bayes and ML should be used to support this conclusion.
3. The evolution rate of key nodes in the evolutionary history is important, thus I recommend the analyses of bowfin evolution rate comparing to its relative species (such as cavefish, channel catfish, zebrafish, spotted gar, etc.).
4. In the “Sex determination in bowfin” section, the analysis was achieved by implementing Pool-Seq analyses leading to a conclusion of “sex determination in bowfin is either polyfactorial, too small to be detected using Pool-Seq, and/or based on environmental effects”. As a complement, a k-mer based reference-free approach searching was also provided. Here, a detailed description of the candidate sex-determining genes or regulate elements related to these sex-specific k-mer sequences should be listed.
5. According to the channel catfish genome paper, only several exons of scpp1 and scpp5 are retained whereas others including scpp8 are lost (Liu, Z., Liu, S., Yao, J. et al. The channel catfish genome sequence provides insights into the evolution of scale formation in teleosts. Nat Commun 7, 11757 (2016). https://doi.org/10.1038/ncomms11757). However, on the contrary, this study showed a different result. In the main text, they described only scpp8 is retained, but in the supplementary Fig. 11, both scpp1 and scpp5 could be detected. Please implement a thorough examine and make a modification for this misleading description.
6. “For each developmental stage, two replicate libraries were sequenced with 75 bp single-end reads, and a third was sequenced with 150 bp paired-end reads on an Illumina NextSeq machine” and “One whole embryo per stage was dissociated into a cell solution with 0.125% collagenase (Sigma C9891) at 37°C until completely dissociated, then strained (100uM filter) and counted on an improved Neubauer chamber”. The authors used the RNA-seq data from different batches, which could easily affect the accuracy of the transcriptome results. The accuracy of the analysis result needs to be evaluated more carefully. Besides, at least three biological replicates should be used in the ATAC-seq analysis.
7. The authors have made great efforts to analyze the biological characteristics of bowfin in many

aspects, but they are not well-organized in the introduction section, which makes the result section of the whole paper looks very isolated and scattered. It would be useful if the text would have a logical framework in introduction and result sections.

Minor comments:
1. In the “Bowfin genome assembly and annotation” section, the repeats content of bowfin (~22%) is very close to that of spotted gar (~21%). Although the overall proportion is very close (Supplementary Table 2), the differences of SINE, Satellite/Simple-repeat, DNA content is significant, which suggest the insertion of some repeats may happen after the divergence between them. The insertion time of these repeats in spotted gar and bowfin should be further analyzed.
2. In the “Unraveling the holostean immunogenome” part, “Class I and class II genes are tightly linked on one chromosome in cartilaginous fishes and tetrapods. In contrast, teleost class I and class II genes are not linked, and class III genes are scattered throughout teleost genomes... Bowfin, in contrast, has a cluster on superscaffold 14 that contains the majority of class I, II, and III genes”. This may lead to a misunderstanding that superscaffold (chromosome) 14 in bowfin is a fused chromosome comparing to the teleost, but in Fig. 1c, it didn’t show a merge event. The description of this part should be corrected, and I recommend a synteny analyses between bowfin Chr14 and zebrafish genome. What’s more, the MHC class in cartilaginous fish should be added in Fig. 2.
3. Lost and retained genes cannot be well distinguished in Fig. 3. I recommend using dotted lines or different colors to distinguish.
4. In Fig. 5, the Hox cluster organization in bowfin and spotted gar genomes are exactly the same, including the genes in each cluster and transcriptional direction. Since the two species have a very close phylogenetic relationship, is there any variation in their amino acid sequence comparing to other species?
5. Some writing mistakes are recommended to polish across the paper, such as “Fig.” and “Figure”, “HiC” and “Hi-C”, “kbp” and “kb”, and “mbp” and “mb”.
6. An adult male was sampled and used for genome sequencing, and the word “male” appears many times. Whether the author has successfully assembled the sex chromosome needs to be clarified.
7. Except for the scaffold N50, the size of contig N50 is also a very important metrics to evaluate the quality of genome assembly. It is recommended to supply this value to the readers.
8. The authors claim to generate a chromosome-level genome assembly of bowfin, but using the word “superscaffold” instead of “chromosome” in the text. The authors should explain the reason and supply the result of the Hi-C interactions.
9. The authors got a very good CEGMA and BUSCO results of the genome assembly. However, the BUSCO analysis for the protein-coding genes was missed.
10. The authors described that both teleost and bowfin lack micro-chromosomes. More evidences and analyses should be provided to get a more reliable conclusion. If not, the authors should be careful to make this conclusion.
11. “We leveraged 3,223 marker genes present in all genomes”. Please clarify how these genes were filtered from the total coding-genes?
12. “The total number of bowfin SCPP genes (22) is considerably smaller than that of gar, which has the largest known SCPP gene repertoire among vertebrates (38). All 22 bowfin SCPP genes have a gar ortholog”. This result suggests 16 SCPP genes were not existed in bowfin genome. The authors should employ more analysis, such as the alignment of raw sequencing data of bowfin, to further examine or prove this conclusion.
13. The definition of “Conserved Non-coding elements (CNEs)” and “Ultra Conserved Elements (UCEs)” should be explained clearly in the main text.
14. “Members of this paralogy group are absent from tetrapods and teleosts, but present in lamprey,

cartilaginous fishes, paddlefish, sturgeon, and gar.” If so, that’s very interesting.
15. In Method section, could the authors please elaborate on the detailed parameters used in each software?
16. “which provided 89.9x physical coverage of the genome (1-50kb pairs)”. Does the word “genome”, represents the evaluated genome size or the assembled genome size? Please clarify.
17. “For each library, chromatin was fixed in place with formaldehyde in the nucleus and then extracted”. More detailed information, such as concentration and time, needs to be provided. This is just an example, please check and revise the similar problems in the Method section carefully.
18. “A file containing nucleotide counts for each genomic position was generated with the PSASS pileup command v3.0.1b [47310.5281/zenodo.3702337] and used to compute FST between males and females…”. I think it would be helpful to explain the “FST” here, so that more readers can easily understand it.
19. “For each developmental stage, two replicate libraries were sequenced with 75 bp single-end reads, and a third was sequenced with 150 bp paired-end reads on an Illumina NextSeq machine”. The PCA analysis should be performed in this study.
20. The term “Dre” should be removed from the Fig. 2.

Reviewer #3:
Remarks to the Author:
The bowfin is an iconic textbook example in comparative anatomy for its prototypical fish body plan and key phylogenetic position. Both gar and bowfin are indispensable outgroup to investigate teleost evolution, which shed light on the evolution and development of ray-finned fishes and bony vertebrates. The genome of the gar has been sequenced and published several years ago. In this manuscript, the authors report a chromosome-level high-quality genome assembly of bowfin genome, providing another reference genome at the basal position for evolution and development studies in teleosts and tetrapods. The authors investigated the bowfin’s phylogenetic position and chromosomal evolution based on the genome information, explored several key gene families, gene regulatory regions, and developmental processes in bowfin genome.

In my view, this is a very significant new paper, first because it reports a high-quality chromosome-level reference genome of the evolutionary important bowfin; second, because it provides significant evidences to support the monophyly of holostean fishes, clarifying the long-standing, controversial debate; third, because it promotes the wider exploration of the gene organizations and functional roles of several gene families related to development and evolution. This paper will interest a wide variety of scientists with an interest in evolutionary mechanisms. I have comments and suggestion on some specific paragraphs, as follows:

The data set of bowfin offers valuable insights into genome evolution in holostean fishes and the genomic basis of phenotypes in ray-finned fishes, which indeed will greatly benefit researchers in this field. While accession numbers the author provided couldn’t be retrieved. I recommend the authors to release all related genome data. The manuscript reported a high-quality genome assembly of bowfin and the BUSCO assessment using eukaryotic BUSCOs (303 single-copy orthologs) showed 100% complete. It would be good if the authors could try vertebrate BUSCOs, which include more than three thousand BUSCOs.

The authors report the results for identifying sex determination region or loci in bowfin genome using

Pool-seq approach with high-quality reference genome of bowfin. They stated that no genomic region exhibiting sex differentiation was observed. As shown in Supplementary Fig.3, however, the sequence depth was clearly different between female-pool and male-pool in multiple chromosomes, including Chr 12, 18 and 22, and many female specific SNPs can be also observed in Chr 4, 8, 9 and 11. Based on the results, I suggest the authors to check these regions and the harbored genes, and perform more comparisons with the sex determination genes or mechanisms in teleost species. Besides, I also suggest to genotype the 30 males and 30 females individually by using whole genome resequencing (or Restriction-site associated DNA sequencing), and conducting a genome wide association test to identify the sex associated regions, which would generate more comprehensive result for sexual determination regions or loci detection. In Supplementary Note 3, the author discussed that "the male reference genome represents the homogametic sex". In Supplementary Table 5, however, the authors suggested that male possesses heterogametic sex in bowfin. It seems that a controversial result was observed between reference-based and reference-free k-mer analysis. Please clarify.

The manuscript mentions the sexual dimorphism of bowfin, but only provides male photo in Figure 1. I suggest to add both female and male photo for demonstrating sexual dimorphism of bowfin. The developmental stage of these 30 males and females should also be reported.

Bowfin represents a once large taxonomic group that is now mostly extinct. It would be interesting to calculate the historical population size, and find out the reasons for the significantly reduced population size, such as geological events.

I find the section to test the Holostei vs. Halecostomi scenarios very interesting. Focusing on the micro-chromosomes in gars, but absent in teleosts and bowfin, the authors identified orthologous chromosomes related to micro-chromosome fusions between bowfin and gar, bowfin and medaka, and found out the convergent evolution of bowfin and teleosts. Combing with the gene gain and loss and some other analysis, evidence efficiently supports the monophyly of holostean fishes. Thus settled the long-debated phylogenetic relationship with gar. My concern is that the gar genome includes 29 LGs, and the closely related bowfin has 23 LGs, while only 3 two-to-one chromosome triplets between gar and bowfin were identified based on orthologous genes. Could the authors try a syntenic analysis based on genome sequence using a circos plot or some others to better reflect the chromosome fusion and genome organization between bowfin and gar?

SCPP gene family encodes proteins involved in biomineralization and was the research hotspot of fish genomics and evolutionary developmental biology in recent years. In this manuscript, 22 SCPP genes were identified from two large genomic clusters in bowfin, which arranged similarly to gar. A reduced gene cluster on superscaffold 9 in bowfin genome was identified by the syntenic analysis with gar, which was helpful to the evolutionary developmental biology study of scale in ray-finned fishes. Expression evidences in gar and zebrafish, as the authors cited in the manuscript, suggested that the SCPP genes in this cluster are potentially involved in scale formation. I suggest to perform similar experiment and collect SCPP gene expression evidences in bowfin, which is vital to support the inference. The manuscript suggests that enam and ambn genes silence their expression during scale development but not in dental enamel formation. Could the authors provide gene in situ hybridization or expression evidence to support it?

A total of 43 bona fide Hox cluster genes were identified with the same repertoire as gar. Of which, Hoxd14 was identified as a pseudogene in the bowfin genome. High expression of hoxd14 was detected in the tail bud and vent at stage 24 and in the posterior pectoral fin mesenchyme at stage

26, which was also supported by reliable gene expression evidences in paddlefish, shark and lamprey. However, gene expression evidences of hoxd14 in gar were not mentioned in the manuscript. Considering the monophyletic relationship of bowfin and gar, transcriptional evidence of hoxd14 in gar is important for better understanding the function and evolution of hoxd14 in Holostei .

Both bowfin and gar are bimodal breathers that have the capacity to breathe both from water and air. They have a gas bladder that serves to maintain buoyancy, and also allows them to breathe air. It would be interesting to identify the genes or gene families associated with bimodal breathers.

Author Rebuttal to Initial comments

Response to Reviewers

We would like to thank all three reviewers for the supportive evaluation of our work and their helpful comments and thoughtful suggestions – all of which have significantly improved this study. Please find our response to comments below, addressing each individual point in detail.

In addition to these changes, we have incorporated a recent analysis of scaffolded genome data from bowfin from a recent published work on ray-finned fishes[1]. As our bowfin data is far more extensive with a chromosome-level genome assembly, our work extends, corrects, and clarifies many of the points made in the analysis by Bi et al. (2021)[1], and we have cited them in the text where appropriate.Our extensive developmental analyses performed here are able to extend simple, and sometimes errant, analyses of presence of genetic loci with changes in their activity in relation to character change. We feel that this extensive work truly highlights the elegance of understanding holostean fishes to detail the genetic changes underlying character change and robustness of developmental pathways to generate diverse forms.

Response to individual reviewers' comments:

Reviewer #1:

Remarks to the Author:

NG-A56077 entitled "The genome of the bowfin (Amia calva) illuminates the developmental evolution of ray-finned fishes" describes the genome sequencing and the evolutionary and developmental analysis of bowfin, Amia calva. I think this paper will be a milestone for the genome analysis of ray-finned fish. The findings described in this manuscript are the last missing stone for the effects or impacts of the teleost-specific whole-genome duplication in ray-finned fish evolution. The manuscript is presented clearly and all data presented here are very interesting and impressive for readers of the evolutionary and developmental biology field. Analyses of the chromatin accessibility by ATAC-seq presented this manuscript are more attractive. This is new data and has different dimensional analysis comparing with the spotted gar (Lepisosteus oculatus) paper previously described by the same author. I have no major comments on this manuscript but some minor comments or suggestions.

We thank the reviewer for the supportive evaluation of our study and the constructive feedback. Supporting the reviewer's highlights of our approach, we also would like to highlight that recent articles on non-teleost fish genomes[1,2] did not include any developmental analyses or chromatin accessibility (ATAC-Seq) or other detailed gene regulatory investigations similar to ours here. We agree that this increased depth of analysis makes our analysis unique and broadly impactful.

Minor Comments

1. One important question on the ray-finned fish genome is long conserved synteny among ray-finned fish genomes. I think there are two possible explanations of this conserved synteny. One possible explanation is an effect of high fecundity (egg number) in the ray-finned fish. Most individuals with inter-chromosomal rearrangements or fission will mate with individuals that have normal chromosomes. F1 fish and descendants have deleterious effects with chromosomal trisomy or monosomy and individuals with arranged chromosomes will disappear from the population.

This is an interesting hypothesis postulating that high fecundity prevents the fixation of deleterious chromosomal rearrangements. This hypothesis certainly needs careful further investigation in the future and will require chromosome-level genome assemblies from non-teleost ray-finned fishes, well-annotated for both coding and non-coding genetic elements, such as presented here.

Another possible explanation is long distance enhancers within the same chromosome. Enhancers and promoters cannot interact each other after chromosome fission and then disappear from the population. Authors did Hi-C analysis in this manuscript. I ask for analysis of the long-distance enhancers for the conserved synteny comparing with Hi-C data of mammalian genomes.

We thank the reviewer for this important suggestion. Here, we have added the Hi-C contact maps from blood of the single bowfin individual from which the genome was assembled. The main initial motivation of Hi-C here was to use it for scaffolding the genome assembly and we succeeded in generating 23 chromosome pseudomolecules/pseudochromosomes (using the NCBI terminology), matching the n=23 chromosomes of the bowfin karyotype.
To address this reviewer's point (and a similar point raised by Reviewer 2), we have now included the genome wide Hi-C contact maps in the new Supplementary Fig. 2d as well as in the new Extended Data Figure 1b-d.

Using the Hi-C contact information, we exemplarily target in on a topologically associating domain (TAD) on bowfin pseudochromosome 15 that contains the hemoglobin gene cluster region (Extended Data Fig. 1 c,d). Within this TAD, our ATAC-Seq data furthermore identify a well-known hemoglobin gene enhancer[3] as an intronic open chromatin region within the neighboring *nprl3* gene (Extended Data Fig. 1e). The TAD shows conserved synteny (Extended Data Fig. 1f) with the tetrapod hemoglobin region TADs[4,5], supporting the high quality of our data. We agree that more extensive, in-depth investigations of Hi-C data from bowfin and gar from multiple developmental stage and diverse adult tissues in comparison to other vertebrates – and integrating chromatin accessibility data as presented in our example – will be an important future research avenue. While we are working towards this goal by investigating a broader panel of Hi-C samples for both holosteans species and teleosts, such extended efforts are beyond the scope of the present study.

2. Authors identified 163,771 open chromatin regions (OCRs) in bowfin genome and described that "Orthologs of 61% of human enhancers (600/989) were found in bowfin and over half (52%; 314/600) overlap with bowfin ncOCRs. In contrast, orthology of only 45% (449/989) of human enhancers could be established in the zebrafish genome (Supplementary Table 18), illustrating the usefulness of the slowly evolving and 'unduplicated' bowfin genome to connect accessible, non-coding, regulatory regions from human to fish.". This is interesting but I am also interested in sequence conservation of ncOCRs among ray-finned fish. 51 fish genomes from the Ensembl Compara database are available. I ask for an analysis of conserved ncOCRs among bowfin, ray-finned fish, and human or mammals' genomes. The ncOCRs specific to fish genomes will "shed light on the evolution and development of ray-finned fishes" specific phenotypes.

We thank the reviewer for this important suggestion. In fact, we are currently working on a more extensive atlas of ncOCRs throughout holostean development by surveying multiple developmental stages and adult tissues of spotted gar in comparison to the bowfin data here as well to data from teleost fish (zebrafish, medaka). This will reach a depth of analysis that is currently not possible to achieve with the bowfin system alone, given its more difficult husbandry

and limited sample availability compared to gar. Such large-scale analyses have been set back by the COVID pandemic lockdown and the unavailability of sampling opportunities [see also Nature 580, 19 (2020) doi: https://doi.org/10.1038/d41586-020-00964-y] and thus are beyond the scope of the current study but will be included in a complete study on its own.

Whole genome alignments (WGAs) for 50+ species are furthermore a computationally highly challenging task, even for genome centers dedicated to such efforts. Nevertheless, we are working towards an extensive WGA data set to be integrated with the above mentioned, extended follow-up study of holostean ncOCRs. Although 50+ ray-finned species are available through Ensembl, bowfin as well as some other key fish species (bichir, sturgeons) have not been integrated yet. It is important to note that numerous fish genomes in Ensembl are from the Vertebrate Genome Project (VGP) and their Embargo Data Use Policy (https://genome10k.soe.ucsc.edu/data-use-policies/) prevents the use of genome-wide data from the species before publication.

However, to address the reviewer's point here, we now provide an extended analysis of the sequence conservation of OCRs and ncOCRs among our focal species. To this end, we have generated of a 5-species Progressive Cactus WGA that includes bowfin, gar, zebrafish, human, and mouse (Cactus runtime >1 month for completion with these 5 species). This 5-way WGA is a more comprehensive approach to establish orthology among holosteans, teleosts, and tetrapods than the BLAST approach we used in the previous version. The WGA results obtained here are nevertheless highly consistent with the previous BLAST-based results and conclusion remain the same. Given this, we chose to include the WGA results only in the revised manuscript. We now present conservation data for the bowfin OCRs/ncOCRs in the other species in a new Supplementary Table 20 and new Supplementary Fig. 15. Additionally, we have updated all our analyses for the overlap of OCRs/ncOCRs with known genetic elements from the literature (bowfin UCEs, gar-centric CNEs, human and mouse VISTA enhancer, mouse singe nuclei OCRs) on orthologies established with the WGAs (revised Supplementary Tables 21-24).

To further address the reviewer's request to identify ray-finned specific ncOCR elements, we connect our bowfin ncOCR data to more fine-grained conservation information from our previously generated gar CNEs[6], which allows us to parse them into ray-finned specific (RCNEs), bony vertebrate specific (BCNEs), and pan-gnathostome elements (GCNEs) as defined in Braasch et al. (2016)[6] (see revised Supplementary Table 21). We find that CNEs conserved from the gnathostome ancestor more often intersect a bowfin ncOCRs than those that emerged later in the bony vertebrate or ray-finned evolution. Furthermore, we also identify "fish-specific" elements (FCNEs) from Braasch et al. (2016)[6] that are specifically lost in tetrapods (see revised Supplementary Table 21) and that have the potential to inform the water-to-land transition at the gene regulatory level in future studies.

We comment on all these new approaches, data, and results in succinct detail in the revised main text and in the revised Supplementary Note 8.

Reviewer #2:

Remarks to the Author:

The authors reported a chromosome-level bowfin (Amia calva) genome assembly with 23 chromosomes and shed light on the evolution and development of ray-finned fishes and bony vertebrates in general. This is an interesting and valuable manuscript that generated high quality genome and comparative analyses for bowfin, which is a unique and important model in many studies across ray-finned fishes and bony vertebrates. Despite the considerable efforts made on generating and analyzing the data, still some details are missing and should be reported. Here, I provide suggestions on further improvement of their analyses and presentation of the results.

We thank the reviewer for recognizing the value of comparative studies on organisms like bowfin with key phylogenetic positions poised to inform vertebrate genomic and developmental evolution. We appreciate the attention paid to providing important suggestions that we think have further strengthened our study.

Major comments:

1. As an important species in the evolution and development of vertebrates, the genome assembly is of vital importance. As described in the manuscript, the bowfin genome assembly is of high quality in genome accuracy and integrity. However, there is no evidence on it. For example, some basic figures, like diagram of chromosome interactions, short-read mapping ratio, transcript mapping ratio, and the short-read depth results, are missing and should be specifically described and added.

We agree that the genome assembly is of utmost importance and is a critical foundation for all subsequent analyses. A high-quality genome assembly at the chromosome level is paramount to achieve the chromosome rearrangement and gene order analyses performed here and are essential for identifying *cis*-regulatory loci in proximity to their target genes.

To address the reviewer's request for additional quality measures of our genome assembly, we have added genome-wide Hi-C contact maps illustrating the higher interaction frequency within our chromosome pseudomolecules than between them (new Supplementary Fig. 2d, Extended Data Fig. 1b). More detailed information on the final genome assembly as well as on the intermediate *de novo* and Chicago-based assemblies is provided in the revised Supplementary Table 1. The new Supplementary Figs. 1-2 detail our scaffolding steps with Chicago and Hi-C. We further report high read mapping statistics for genome assembly (Supplementary Table 2) as well as RNA-seq experiments (Supplementary Table 2 and Supplementary Note 6.1).

2. The authors introduced a lot about the phylogenetic relationship, which is in fact one of the most fascinating point for bowfin research, but only one phylogenetic tree was constructed with few proofs. I recommend a detailed reconstruction of the phylogenetic relationships, including but not limited to the construction of gene tree and species tree, with the non-coding regions. Besides, except for NJ, more models, including Bayes and ML should be used to support this conclusion.

Thank you for this important suggestion and we fully agree that it is critical to have high statistical confidence in the monophyly of holosteans due to our subsequent interpretation of evolutionary changes over vertebrate evolution.

First, we would like to highlight that a large body of literature already exists that favors the Holostei scenario of neopterygian relationships, based on sequence data, i.e., phylogenomic studies using various nuclear genomic data sets (coding and non-coding) and a variety of approaches for phylogenetic inference. A number of exemplary studies are cited in the article, including Near et al. (2012)[7], Betancur et al. (2013)[8], Broughton et al. (2013)[9], Braasch et al. (2016)[6], Irisarri et al. (2017)[10], Hughes et al. (2018)[11], and Bi et al. (2021)[1].

To address the reviewer's request for additional phylogenetic analyses at the sequence level (adding to the studies from the literature summarized above), we constructed Maximum Likelihood and Bayesian phylogenies with the 2,079 1:1 orthologs obtained from Orthofinder for 12 vertebrate species (see new Supplementary Note 4). Both show 100% (bootstrap and posterior probability) support for holostean monophyly (new Supplementary Fig. 6a), in agreement with the aforementioned published phylogenomic studies, the OrthoFinder species tree generate with STAG (new Supplementary Fig. 6b) as well as with our phylogenetic inference using gene adjacencies as discussed below.

It is important to note that here <u>we present an additional, highly novel approach to reconstruct the phylogenetic relationships of neopterygians by using data from gene order rearrangement (gene adjacency)</u> in the genomes of bony vertebrates. To the best of our knowledge, such an approach has been rarely used in the literature and thus our work here has highest potential to serve as a template that can be applied to many other controversial phylogenetic relationships in the future, now that chromosome-level genome assemblies are becoming increasingly more common. In the revised version, we expand our phylogenetic inference from the distance matrix generated from the gene adjacencies. The matrix has been added as new Supplementary Fig. 5). All methods we used (NJ, FastME, UPGMA) were successful in reconstructing phylogenies strongly supporting holostean monophyly (100% bootstrap support for all three methods; see revised main Fig. 1e).

Overall, we are extremely confident that our analyses here at the sequence, gene order, and genome structure levels finally puts the century-old, controversial debate about neopteryigan relationships, the so-called 'gar-*Amia*-teleost problem'[12], to rest.

3. The evolution rate of key nodes in the evolutionary history is important, thus I recommend the analyses of bowfin evolution rate comparing to its relative species (such as cavefish, channel catfish, zebrafish, spotted gar, etc.).

We would like to emphasize that the molecular evolutionary rate of holosteans (both gar and bowfin) in comparison to teleosts and other vertebrate lineages has already been extensively analyzed by ourselves in the gar genome paper (which included bowfin reference transcriptome data)[6] as well as by others using our bowfin and gar data[13,14] that subsequently confirmed the results of the gar genome article. We now make references to these published studies more obvious in the main text.

Here, we have added a completely novel way of analyzing rates of genomic evolution by analyzing the change in gene adjacencies (see Methods and new Supplementary Note 5). In the previous article version, we only compared gar and bowfin though (and found no significant difference in their rates). To address the reviewer's important point, we have now also added

comparisons of bowfin to zebrafish, medaka, and arowana to cover comparisons to three main branches of teleost diversity: ostariophysians, percomorphs, and osteoglossomorphs, respectively (new Supplementary Note 5). All three comparisons reveal a significantly lower rate in bowfin compared to the three main teleost lineages, confirming the slow rate of holostean genome evolution also at the level of gene order arrangements.

4. In the "Sex determination in bowfin" section, the analysis was achieved by implementing Pool-Seq analyses leading to a conclusion of "sex determination in bowfin is either polyfactorial, too small to be detected using Pool-Seq, and/or based on environmental effects". As a complement, a k-mer based reference-free approach searching was also provided. Here, a detailed description of the candidate sex-determining genes or regulate elements related to these sex-specific k-mer sequences should be listed.

Using a k-mer approach to list all potential candidate sex-determining genes would be very difficult given the small size of these sequences (31 bp), the high number of sex-biased k-mers, and the lack of annotation of these k-mer sequences. But we agree that an analysis focusing on genes and their coding sequences (CDS) instead of the whole genome is important. As such, we have extracted from the Pool-seq analysis results of all annotated genes with a sex-biased pattern = (gene regions with a mean corrected male depth > 10 reads OR a mean corrected female depth > 10 reads) AND [(with a difference between male sex-specific SNPs and female sex-specific SNPs > 5) OR (a male/female mean corrected depth >= 1.5 OR <= 0.66)].

Using this approach, we identified 104 genes (see new Supplementary Table 7) exhibiting a sex-biased pattern (either sex-biased coverage or sex-biased SNPs). These 104 genes are scattered among 22 of the 23 bowfin chromosomes and on 3 unplaced scaffolds (Aca scaf 31, Aca scaf 55 and Aca scaf 68), without any special biologically meaningful enrichment (i.e., more than a single gene in a given genomic region with the same bias) in a restricted region that could be seen as a potential sex determining region on a sex chromosome. Their gene annotations (see new Supplementary Table 7) did not reveal any ortholog of a known vertebrate sex determination gene.

This absence of a restricted genomic region enriched in sex signal for CDS and the lack of any candidate as a putative master sex determining gene constitute additional pieces of evidence (in addition to the Pool-seq and k-mer whole genome analyses) that bowfin sex determination is more complex than a strict and simple monofactorial system with a well differentiated sex locus.

These additional results have been integrated in the Supplementary Note 3.

5. According to the channel catfish genome paper, only several exons of scpp1 and scpp5 are retained whereas others including scpp8 are lost (Liu, Z., Liu, S., Yao, J. et al. The channel catfish genome sequence provides insights into the evolution of scale formation in teleosts. Nat Commun 7, 11757 (2016). https://doi.org/10.1038/ncomms11757). However, on the contrary, this study showed a different result. In the main text, they described only scpp8 is retained, but in the supplementary Fig. 11, both scpp1 and scpp5 could be detected. Please implement a thorough examine and make a modification for this misleading description.

Liu et al. (2016)[15] stated that in the genome of the scaleless channel catfish, *scpp8* is absent and only parts of *scpp1* and *scpp5* were identified. It was further stated that *scpp5* is absent in scaleless three-spine stickleback, while *scpp1* is absent in the scaleless electric eel. Based on these observations, the study concluded that the loss of *scpp1* or *scpp5*, or both would cause the scaleless phenotype. By contrast, our reanalysis of the channel catfish genome revealed the

presence of apparently functional (complete ORF and RNA-seq data) *scpp1*, *scpp5*, and *scpp8*, as we shown in main Figure 3. Moreover, we identified stickleback *scpp5* and electric eel *scpp1*. We show amino acid sequences encoded by channel catfish *scpp1* and *scpp5*, stickleback *scpp5*, and electric eel *scpp1* in revised Supplementary Figure 13, which has been updated to include electric eel *scpp1* and *scpp5*. Our analysis thus suggests that neither *scpp1* nor *scpp5* is relevant for the scaleless phenotype in teleosts. We clearly state these results in the revised Supplementary Note 7.

In response to the reviewer's comment, we have furthermore updated the text, incorporating information of a recent study by one of our authors[16] highlighting the involvement of *scpp5* in ganoin formation in gar.

In addition, information from a recent analysis of the SCPP gene family in the scale-reduced sterlet sturgeon and paddlefish[2] has been integrated into the revised main text and Supplementary Note 7, which further strengthens the importance of SCPP genes on gar LG2 and bowfin 9 for scale formation. However, scale composition in acipenseriforms is not well characterized, with some conflicting information regarding the presence of ganoin in the literature depending on species (as discussed in Supplementary Note 7). Our own analyses of sterlet SCPP genes (the paddlefish genome assembly is not yet publicly available) reveal complex differences between several available genome versions and the published analysis (K. Kawasaki, unpublished data). Thus, while the published acipenseriform data as cited in Supplementary Note 7 are in line with our overall conclusions, a more detailed comparison of neopterygian and acipenseriform SCPP genes will require additional investigation of scale formation in acipenseriforms and important improvements to their genome assemblies in the future.

6a. The authors used the RNA-seq data from different batches, which could easily affect the accuracy of the transcriptome results. The accuracy of the analysis result needs to be evaluated more carefully.

We agree it is important to examine the data/results for batch effects. Thus, we examined this with a PCA analysis for the RNA-Seq data in the new Supplementary Figure 17. As further discussed in revised Supplementary Note 11, we illustrate the clustering of transcriptomes when examining the gene expression of 100 core limb development genes (Supplementary Figure 17b) to visualize potential variation. We furthermore provide RNA-seq read mapping statistics in new Supplementary Table 2 and Supplementary Note 6.1, showing the relative consistency of mapping rate across the different RNA-Seq libraries.

6b. Besides, at least three biological replicates should be used in the ATAC-seq analysis.

We appreciate the reviewer's dedication to increasing power of statistical analyses by suggesting more replicates. The requested replicates are standard for established laboratory models like mouse or zebrafish but extremely difficult to get by with a 'non-model' species' like the bowfin. We would like to point out that we conduct all developmental genetic analyses in bowfin on wild-caught embryos. This is because, although bowfin is an invaluable missing piece of the puzzle to understand vertebrate evolution, this non-model species thus far cannot be spawned in captivity. We and others studying bowfin must rely on eggs collected from wild nests guarded by an aggressive, protective male that enters flooded backwaters during a rainy spring. We have obtained these highly precious bowfin embryonic samples prior to 2019. Because of unfavorably dry weather conditions in 2019 (male bowfin make nests in shallow

backwaters/flooded regions) and COVID research restrictions since spring 2020, no new eggs were collected in the past two years; unfortunately, COVID restrictions are still ongoing.

Despite the lack of opportunities for additional systematic sampling and the impracticalities for a controlled laboratory breeding program, we have been able to add additional samples and replicates to our bowfin ATAC-Seq series from previously fixed samples. For the revised study, we sequenced an earlier bowfin developmental stage (st. 21-22) prior to the conserved phylotypic stage as well as additional replicates for two other developmental stages (phylotypic st. 22-23 and st. 28-29), representing early embryonic and later larval development, respectively. This brings our total number of sequenced libraries to 9. The present work represents not only the only ATAC-Seq study conducted on non-teleost fishes, but – to the best of our knowledge – also the only chromatin accessibility study conducted on wild-caught, non-model fish embryos.

All our ATAC-Seq libraries are of high quality and reflect expected nucleosome periodicity (revised Supplementary Fig. 14a). We also show with a PCA and heatmap of pairwise jaccard distances that obtained OCRs/ncOCRs are most similar to their replicate library or adjacent developmental stage (Supplementary Fig. 14b,c). Additionally, by sequencing 9 ATAC-Seq libraries through development, libraries across developmental stages can also be considered as internal controls when identifying conserved non-coding regulatory loci. For example, we find 21,636 ncOCRs conserved across all seven developmental stages (revised Supplementary Table 18).

As a note, we are very careful to not overinterpret our OCR data. For all our conclusions regarding their potential as gene-regulatory elements, we are relying on their overlap with other data sets from the literature in support of regulatory function such as sequence conservation to other species (Supplementary Fig. 15, Supplementary Table 20), overlap with previously established UCE and CNEs (Supplementary Table 21) and functional validation data from the literature such as the VISTA Enhancer elements (Supplementary Tables 22-23), other described enhancers from the literature (Fig. 4, Extended Data Figs. 1e, 4), and mouse developmental OCRs (Supplementary Table 24).

As an additional measure for the stringency of all these comparisons of bowfin chromatin accessibility to known genetic elements, we now also provide information on the subset of overlap with OCRs/ncOCRs in 2 or more bowfin developmental stages (2+ stages; see Supplementary Tables 17, 20-22, 24). Overall, we show that the vast majority, around 70% of OCRs and of ncOCRs, overlap in 2+ developmental stages (Supplementary Table 17). Our criteria of overlap to a conserved element require coverage of at least 33% in length (see Methods), which is a stringent approach to avoid small ‘fringe overlap’ simply by chance.

For all these reasons, and especially because many ncOCRs are orthologous with known, experimentally verified enhancers active in a variety of other species (human, mouse, zebrafish) as well as across organs and tissues (heart, brain, lung, limb, etc.), we are highly confident that our ATAC-Seq data presented here strongly support the conclusions drawn in the manuscript.

7. The authors have made great efforts to analyze the biological characteristics of bowfin in many aspects, but they are not well-organized in the introduction section, which makes the result section of the whole paper looks very isolated and scattered. It would be useful if the text would have a logical framework in introduction and result sections.

We thank the reviewer for pointing to this need for organizational clarification of the introduction. We have addressed this with edits to the substantial revision and reorganization of the introduction to provide the reader with a better set up of the results of the study.

Minor comments:

1. In the "Bowfin genome assembly and annotation" section, the repeats content of bowfin (~22%) is very close to that of spotted gar (~21%). Although the overall proportion is very close (Supplementary Table 2), the differences of SINE, Satellite/Simple-repeat, DNA content is significant, which suggest the insertion of some repeats may happen after the divergence between them. The insertion time of these repeats in spotted gar and bowfin should be further analyzed.

We thank the reviewer for suggesting this additional analysis. We have now added this to the manuscript, and indeed it revealed some striking differences between bowfin and spotted gar repeats. To make the holostean repeat content as comparable as possible, we first reannotated repeats in the spotted gar genome with the exact same methods and repeat libraries used for bowfin (instead of just relying on our previously published data[6]). This generated a highly similar repeat content result for spotted gar to our previous analysis and serves as an internal control for the appropriateness of our methods for bowfin.

Next, as suggested, we generated Kimura substitution level-based repeat landscapes for both species to show the timing of repeat activities in both genomes (new Supplementary Fig.3). Both holostean genomes evolve at similar rates (see above) and Kimura distance are thus directly comparable across the two species. While we found bursts of transposable element (TE) activity at Kimura distances 25 and 7-8 in spotted gar (as we have described previously[6]), we do not see similar peaks in the bowfin genome. The bowfin does not show any 'older' peak of TE activity, but a more pronounced and more 'recent' signal at Kimura distance 4 that is not seen in spotted gar. Not only do we not see an overlap by Kimura distance of the single burst in bowfin and the two bursts in spotted gar, but these peaks are also based on amplifications of different TE classes. We therefore conclude that despite their overall similar total repeat content, the two genomes have undergone quite different repeat activity trajectories. Neither of the two genomes shows a very strong recent TE activity at Kimura distance 0-1 as seen in mammals and teleosts[6].

These new results are now briefly mentioned in the main text and presented in detail in the revised Supplementary Note 2.2, the new Supplementary Fig. 3, and the revised Supplementary Table 4.

2a. In the "Unraveling the holostean immunogenome" part, "Class I and class II genes are tightly linked on one chromosome in cartilaginous fishes and tetrapods. In contrast, teleost class I and class II genes are not linked, and class III genes are scattered throughout teleost genomes... Bowfin, in contrast, has a cluster on superscaffold 14 that contains the majority of class I, II, and III genes". This may lead to a misunderstanding that superscaffold (chromosome) 14 in bowfin is a fused chromosome comparing to the teleost, but in Fig. 1c, it didn't show a merge event. The description of this part should be corrected, and I recommend a synteny analyses between bowfin Chr14 and zebrafish genome.

Thank you for pointing out this potential misunderstanding. To clarify, we now say in the main text:

“Bowfin, in contrast, has a cluster on pseudochromosome 14 that contains the majority of class I, II, and III genes (Fig. 2, Supplementary Figs. 7,8, Supplementary Tables 9-11, Supplementary Note 6), and that is not the result of a recent chromosome fusion (Fig. 1c, Supplementary Fig. 7).”

In addition, we have added a new Supplementary Figure 4 showing the synteny comparison between the bowfin MHC cluster on Aca scaf14 and the zebrafish genome. We now refer to this figure in the main and supplemental texts when addressing the synteny of bowfin and zebrafish/teleost MHC regions.

2b. What’s more, the MHC class in cartilaginous fish should be added in Fig. 2.

Regarding cartilaginous fish MHC regions, the linkage of MHC class I, MHC class II, TAP1, TAP2, LMP2 (PSMB9), and LMP7 (PSMB8) genes as cited in the text was in fact detected by segregation analyses[17,18], but not by data analyses of cartilaginous fish draft genomes.

Our preliminary analyses of published cartilaginous fish draft genomes (*Callorhinchus milii*, *Rhincodon typus*, *Carcharodon carcharias*, *Scyliorhinus torazame*, and *Chiloscyllium punctatum*) so far failed to confirm the linkage of MHC class I and class II genes due to fragmentation of genomic region near MHC class I and class II genes; relatively small scaffolds contain MHC class I, class II, or class III genes in these draft genomes. Examination of linkage of MHC class I, class II and class III genes thus is currently not possible. The only exception was a scaffold derived from the *Amblyraja radiata* genome, which contains MHC class I and class II genes as well as some MHC class III genes.

Even though these are very interesting data to be analyzed further that confirm previous segregation analyses cited in our text, the data are from the Vertebrate Genomes Project (https://vgp.github.io/). Their Embargo Data Use Policy (https://genome10k.soe.ucsc.edu/data-use-policies/) restricts us to analyze and use freely their data before their first presentation of a genome-wide analysis of the data. Currently, then, we are not able to add the requested cartilaginous fish data to main Fig. 2.

3. Lost and retained genes cannot be well distinguished in Fig. 3. I recommend using dotted lines or different colors to distinguish.

Figure 3 shows the presence of SCPP genes in five neopterygians species and only shows retained, but not any lost genes. Dashed lines indicate genes orthologies among species and we realize that these lines were difficult to see in the previous PDF version. We have improved the visibility/contrast for these dashed orthology lines in the revised version.

In Fig. 5, the Hox cluster organization in bowfin and spotted gar genomes are exactly the same, including the genes in each cluster and transcriptional direction. Since the two species have a very close phylogenetic relationship, is there any variation in their amino acid sequence comparing to other species?

Besides the presence of the extensively discussed *hoxd14* pseudogene in both holosteans species, we did not find any striking differences at the level of gene structures compared to other ray-finned species. We took a detailed look into the gene structure of every Hox gene identified and confirmed:

- Start codon
- Stop codon
- One intron and two coding sequences
- Homeodomain sequence, including three alpha helixes always located in the second coding sequence
- Canonical splicing sites (assessed by visual sequence inspection)

We have added this information to Supplementary Note 10.2. It is worth noting that, even though bowfin and gars are more closely related to each other than to any other extant species, the current estimate of their divergence with holosteans is still >250 million years ago, making this level of conservation of their Hox clusters even more striking.
It is further important to point out that, in contrast to the recently published analysis of the fragmentary draft genome assemblies of bowfin and alligator gar (Fig. S2 in ref.[1]) which proposed loss of *hoxC5* and *hoxC11* in bowfin and loss of *hoxB4*, *hoxD9*, and *hoxD12* in alligator gar, we find these genes to be present in both holostean lineages (main Fig. 5).

5. Some writing mistakes are recommended to polish across the paper, such as "Fig." and "Figure", "HiC" and "Hi-C", "kbp" and "kb", and "mbp" and "mb".

We have fixed these inconsistencies.

6. An adult male was sampled and used for genome sequencing, and the word "male" appears many times. Whether the author has successfully assembled the sex chromosome needs to be clarified.

Genome assemblies with accurately assembled sex chromosomes are very rare, even in species in which sex loci on sex chromosomes have been characterized. This is mainly due to technical limitations as only recent approaches[19] have allowed to produce haplotype-phased and chromosome-size diploid assemblies. Combined with some additional data (Pool-Seq, RAD-Seq or genetic mapping of the sex locus), these haplotype-phased and chromosome-size diploid assemblies can provide successfully assembled sex loci and/or potentially sex chromosomes (i.e., both the X and the Y chromosome sequences or the Z and the W for simple monofactorial systems). Having sequenced a phenotypic male (it would be the same if we had sequenced a phenotypic female) genome does not necessary imply that sex chromosomes have been successfully assembled. The indication that we sequenced a phenotypic male individual is important, even if we did not find any sex locus in bowfin, and despite the fact that published karyotype investigations[20,21] did not support the existence of heteromorphic sex chromosomes in bowfin.

We understand that without the characterization of the bowfin sex determination system the words "male genome" or "male assembly" probably bring an unnecessary precision. We thus revised all these "male" (and "female") references throughout the main and supplementary texts to clearly indicate that we mean the phenotypic male or female conditions.

7. Except for the scaffold N50, the size of contig N50 is also a very important metrics to evaluate the quality of genome assembly. It is recommended to supply this value to the readers.

This information has been added to Supplementary Table 1.

8. The authors claim to generate a chromosome-level genome assembly of bowfin, but using the word "superscaffold" instead of "chromosome" in the text. The authors should explain the reason and supply the result of the Hi-C interactions.

Following this recommendation, we have revised this terminology and got rid of the term "superscaffold". Instead, we now follow the NCBI nomenclature and use the term "chromosome pseudomolecule" or "pseudochromosome" for short. The only instances in which we continue to use the term "scaffold" is when we are addressing assembly regions that are not part of the 23 pseudochromosome (i.e., unplaced scaffolds).

We now also added the Hi-C interactions; please see the new Supplementary Figure 2 and the new Extended Data Figure 1 as well as the response to Reviewer 1 (comment 1) above.

9. The authors got a very good CEGMA and BUSCO results of the genome assembly. However, the BUSCO analysis for the protein-coding genes was missed.

A similar point has also been raised by Reviewer 3 (comment 2.). We now provide a more extended BUSCO analysis for both the genome assembly and the gene annotations in Supplementary Table 3.

10. The authors described that both teleost and bowfin lack micro-chromosomes. More evidences and analyses should be provided to get a more reliable conclusion. If not, the authors should be careful to make this conclusion.

With regard to bowfin, the two studies that have performed karyotype analyses of *Amia calva*, Ohno et al. (1969)[21] and the much more recent, detailed investigation by Matjanova et al. (2017)[20] show that there are no micro-chromosomes in this monotypic species.

With regard to the absence of micro-chromosomes in teleosts, we are not aware of any evidence for true micro-chromosomes in teleosts in the literature and also after consultation with several other experts in the field. B chromosomes have been reported for teleosts and are sometimes called micro-chromosomes in the literature. However, such B chromosomes are supernumerary, non-essential, extra chromosomes that are often are not stably inherited in a Mendelian fashion and/or present in all individuals of a population (e.g.[22,23]) and thus do not qualify for true micro-chromosomes. Of course, since only a minor fraction of the 30,000+ living teleost species have been investigated karyotypically, we cannot exclude the possibility that true micro-chromosomes exist in some teleosts. Interestingly, mounting evidence by us and others[6,24,25] suggests that the ancestral bony vertebrate micro-chromosomes retained in gar and chicken have been absorbed into larger chromosomes in teleosts by pre-TGD fusions as described here.

To make our point clearer, we now say in the main text:

"While gar and chicken retained micro-chromosomes from the bony vertebrate ancestor[7,45,46], karyotype analyses provide no evidence for micro-chromosomes in bowfin[29,37]. To the best of our knowledge, true micro-chromosomes have also not been discovered in teleosts."

11. "We leveraged 3,223 marker genes present in all genomes". Please clarify how these genes were filtered from the total coding-genes?

For clarification, we have changed this key information in the main article (p.7) to:

"… leveraged all 3,223 marker genes that are present in exactly one copy in non-teleost genomes and in one or two copies in the TGD-derived teleost genomes."

The exact breakdown is that 1,527 are in single copy in all species, 1,614 are single-copy in non-teleosts and 1 or 2 in teleosts, and 82 are single-copy in non-teleosts and exactly 2 in all teleosts. This breakdown is provided in the Methods section (p.24).

12. "The total number of bowfin SCPP genes (22) is considerably smaller than that of gar, which has the largest known SCPP gene repertoire among vertebrates (38). All 22 bowfin SCPP genes have a gar ortholog". This result suggests 16 SCPP genes were not existed in bowfin genome. The authors should employ more analysis, such as the alignment of raw sequencing data of bowfin, to further examine or prove this conclusion.

In the genomic sequences of various actinopterygian species, many SCPP genes are overlooked by automatic annotation, partly because sequences of SCPP genes in phylogenetically closely related species are unavailable, and partly because many SCPP genes encode low complexity amino acid sequences that contain long repetitive sequences (and are recognized as different genes that encode similar repetitive sequences or are not recognized as a protein-coding exon).

Our analysis includes blast searches using all known and well-annotated gar SCPP genes as queries against the bowfin genome sequence, including unassembled small contigs, as the database. This analysis effectively detects bowfin SCPP genes because of the comparatively close phylogenetic relationship between gar and bowfin. However, it is still possible that some SCPP genes were missed in this blast search or that some SCPP genes were secondarily lost either in the gar genome or in the bowfin genome. To address this question, we also searched for bowfin SCPP genes in all genomic regions that are syntenic to gar SCPP gene clusters (within bowfin pseudochromosomes 9, 12, and 22) using intron-spanning transcripts obtained from RNA-seq analysis and investigated individual exons to confirm characteristic exon-intron borders of SCPP genes as described previously by us[26] (entirely untranslated exon 1, all phase-0 introns, borders between the signal peptide and the mature protein encoded near the border between exon 2 and exon). In addition, for these genomic regions, we also run the exon-prediction software GENSCAN (http://hollywood.mit.edu/GENSCAN.html) to detect exons directly from the genomic sequence. Although we cannot exclude the possibility that some exons/genes were still missed by our meticulous analysis, it is very unlikely that a large number of bowfin SCPP genes have been overlooked.

We have revised our Supplemental Note 7 to include a more detailed description of our approach and now explicitly mention the GENSCAN exon prediction step.

13. The definition of "Conserved Non-coding elements (CNEs)" and "Ultra Conserved Elements (UCEs)" should be explained clearly in the main text.

We now provide definitions for both terms based on the original definitions from the cited studies[6,27]. Since the definitions are rather complex and difficult to put in a few words, we chose to add them to the Methods section, and we refer to these detailed definitions in the Results sections as well as in Supplemental Note 8.

14. "Members of this paralogy group are absent from tetrapods and teleosts, but present in lamprey, cartilaginous fishes, paddlefish, sturgeon, and gar." If so, that's very interesting.

It is indeed striking to find the presence of *hoxd14* broadly among non-teleost fishes. In holosteans, both spotted gar and bowfin have retained a *hoxd14* pseudogene. Notably, the *hox* gene analysis in a recent article on non-teleost ray-finned fishes[1] missed the *hoxd14* gene for both bowfin and alligator gar which might be explained by the fragmentary nature of their genome assemblies.

Here, despite its predicted pseudogene nature, we find that bowfin *hoxd14* is expressed in a pattern expected for a posterior-most *hox* gene.

Addressing a similar point raised by Reviewer 3 (comment 7.), we now report our attempts to clone *hoxd14* from gar cDNA with a similar strategy that led us to successfully clone bowfin *hoxd14*. Our inability to amplify *hoxd14* from gar is consistent with the previously reported absence *hoxd14* transcripts in gar RNA-Seq data[6], so that there is currently no evidence for expression of this gene in gar. With the data at hand, we cannot tell whether the pseudogenization of *hoxd14* predated the divergence of gar and bowfin or whether the holosteans lineages convergently pseudogenized this gene. Knock-out studies in bowfin would be necessary to further investigate the possibility of any functional role of *hoxd14*, but CRISPR genome editing is – at least currently – not possible in bowfin due to its inaccessible reproductive biology (see above).

15. In Method section, could the authors please elaborate on the detailed parameters used in each software?

The important parameter details have now been added across the Methods section and we also indicate where default parameters were used.

16. "which provided 89.9x physical coverage of the genome (1-50kb pairs)". Does the word "genome", represents the evaluated genome size or the assembled genome size? Please clarify.

We have clarified this in the text and now say:

"…which provided 89.9x physical coverage of the kmer-based estimated genome size of 0.91 Gb (1-50 kb pairs)."

17. "For each library, chromatin was fixed in place with formaldehyde in the nucleus and then extracted". More detailed information, such as concentration and time, needs to be provided. This is just an example, please check and revise the similar problems in the Method section carefully.

We have added the requested information to the Methods section and now say:

"A Chicago library was prepared as described. Briefly, ~500ng of HMW gDNA (mean fragment length = 50 kb) was reconstituted into chromatin in vitro and fixed in 1% formaldehyde for 15min at room temperature."

“A Hi-C library was prepared as described. For each library, chromatin was fixed in place in the nucleus in 1% formaldehyde for 10 minutes at room temperature and then extracted.”

18. “A file containing nucleotide counts for each genomic position was generated with the PSASS pileup command v3.0.1b [47310.5281/zenodo.3702337] and used to compute FST between males and females…”. I think it would be helpful to explain the “FST” here, so that more readers can easily understand it.

We have added the explanation and now say in the Methods:

“A file containing nucleotide counts for each genomic position was generated with the PSASS pileup command v3.1.0 [10.5281/zenodo.4442702] and used to compute the Fixation index (F_{ST}), a measure of genetic differentiation between populations, here between males and females”

19. “For each developmental stage, two replicate libraries were sequenced with 75 bp single-end reads, and a third was sequenced with 150 bp paired-end reads on an Illumina NextSeq machine”. The PCA analysis should be performed in this study.

We now provide a PCA analysis for the RNA-Seq data in the new Supplementary Figure 17 and further explanation in revised Supplementary Note 8.

20. The term “Dre” should be removed from the Fig. 2.

This has been removed and species icons have been added to this and other figures.

Reviewer #3:

Remarks to the Author:

The bowfin is an iconic textbook example in comparative anatomy for its prototypical fish body plan and key phylogenetic position. Both gar and bowfin are indispensable outgroup to investigate teleost evolution, which shed light on the evolution and development of ray-finned fishes and bony vertebrates. The genome of the gar has been sequenced and published several years ago. In this manuscript, the authors report a chromosome-level high-quality genome assembly of bowfin genome, providing another reference genome at the basal position for evolution and development studies in teleosts and tetrapods. The authors investigated the bowfin's phylogenetic position and chromosomal evolution based on the genome information, explored several key gene families, gene regulatory regions, and developmental processes in bowfin genome.

In my view, this is a very significant new paper, first because it reports a high-quality chromosome-level reference genome of the evolutionary important bowfin; second, because it provides significant evidences to support the monophyly of holostean fishes, clarifying the long-standing, controversial debate; third, because it promotes the wider exploration of the gene organizations and functional roles of several gene families related to development and evolution. This paper will interest a wide variety of scientists with an interest in evolutionary mechanisms.

We thank the reviewer for their very helpful comments and suggestions and agree that genomic explorations of non-model species are absolutely critical for testing evolutionary hypotheses.

I have comments and suggestion on some specific paragraphs, as follows:

1. The data set of bowfin offers valuable insights into genome evolution in holostean fishes and the genomic basis of phenotypes in ray-finned fishes, which indeed will greatly benefit researchers in this field. While accession numbers the author provided couldn't be retrieved. I recommend the authors to release all related genome data.

Our bowfin genome assembly has been released into GenBank (accession number PESF00000000) on March 27, 2021.
Please further use this reviewer link to the developmental genetics Bioproject on NCBI:
https://dataview.ncbi.nlm.nih.gov/object/PRJNA659268?reviewer=cj054l1a7gtcikjqi9vni4pfjf
Immune transcriptome data are available under NCBI accessions SRR11303972 (SRA) and GIOP00000000 (TSA).
The full genome data will be publicly released immediately upon publication, following the guidelines provided by *Nature Genetics*.

2. The manuscript reported a high-quality genome assembly of bowfin and the BUSCO assessment using eukaryotic BUSCOs (303 single-copy orthologs) showed 100% complete. It would be good if the authors could try vertebrate BUSCOs, which include more than three thousand BUSCOs.

This point has also been raised by Reviewer 2 (minor comment 9.). We now provide a more extended BUSCO analyses for both the genome assembly and the gene annotations in Supplementary Table 3.

3a. The authors report the results for identifying sex determination region or loci in bowfin genome using Pool-seq approach with high-quality reference genome of bowfin. They stated that no genomic region exhibiting sex differentiation was observed. As shown in Supplementary Fig.3, however, the sequence depth was clearly different between female-pool and male-pool in multiple chromosomes, including Chr 12, 18 and 22, and many female specific SNPs can be also observed in Chr 4, 8, 9 and 11.

It is true that the Male/Female depth ratio (in Supplementary Fig. 4d) and the sex specific SNPs (Supplementary Fig. 4e,f) show variations in some chromosomes and that without more explanations this could be seen to be significant. However, most of these variations are limited to very small regions scattered all over the genome, which does not fit with the expectation of a monofactorial genetic sex determining system. The possibility of more complicated sex determination systems cannot be ruled out with the current data we produced, and these possibilities were already discussed in the manuscript.

However, in the revised Supplementary Note 3 we now provide more explanation on why we think that these results are not sufficient to characterize a sex determination system.

3b. Based on the results, I suggest the authors to check these regions and the harbored genes, and perform more comparisons with the sex determination genes or mechanisms in teleost species.

This has been done with a specific search for sex-biased CDS (see answer to Reviewer 2, main point 4., and new Supplemental Table 7) and we did not find any biologically meaningful signal in CDS, which supports our hypothesis that bowfin sex determination is more complex than a strict and simple monofactorial system with a well differentiated sex locus.

3c. Besides, I also suggest to genotype the 30 males and 30 females individually by using whole genome resequencing (or Restriction-site associated DNA sequencing), and conducting a genome wide association test to identify the sex associated regions, which would generate more comprehensive result for sexual determination regions or loci detection.

As the sex phenotypes of the 30 males and 30 females used for Pool-Sequencing have been recorded with 100% confidence (macroscopic examination of the gonads; this has been made clearer in the methods sections of main and supplemental texts), we can completely exclude that some outliers (erroneously phenotyped individuals) would have prevented the identification of a well-differentiated sex determining region (SDR) in bowfin.

Our pooled sequencing results support that bowfin sex determination is not as simple as a strict monofactorial system with a well differentiated sex locus. The other possibilities are then: 1) a polyfactorial sex determination system, 2) a complete or partial environmental sex determination system (ESD), or 3) an extremely small SDR. A GWAS analysis using Restriction-site associated DNA sequencing will very likely not be resolutive enough to resolve such complex cases. A GWAS with the re-sequencing of whole genomes of many males and many females would likely provide a higher resolution and may provide additional information, but it would require more than 30 males and 30 females to capture a polygenic signal or a very small SDR and therefore be highly complex and very costly to implement. As a comparison, the experimental design that enabled the characterization of the small sex locus in the pufferfish *Takifugu rubripes*[28] was performed using a linkage analysis that included several genetic panels with a total of 1,445 individuals.

We have now modified our supplemental text to suggest this complexity.

3d. In Supplementary Note 3, the author discussed that "the male reference genome represents the homogametic sex". In Supplementary Table 5, however, the authors suggested that male possesses heterogametic sex in bowfin. It seems that a controversial result was observed between reference-based and reference-free k-mer analysis. Please clarify.

We apologize for our unclear phrasing, but this sentence was not stated to mean that "the male reference genome represents the homogametic sex". Instead, the complete version in our previous text was: "It is possible that the male reference genome represents the homogametic sex". We agree that this sentence could be misunderstood, and we thus rephrased the Supplemental Note 4 to better explain our initial idea and now state:

"Without any clear indication of a bowfin sex determination system, we cannot rule out the hypothesis that our bowfin reference assembly from a phenotypic male represents the homogametic sex, and thus would not allow the detection of potential female-specific regions."

3e. The manuscript mentions the sexual dimorphism of bowfin, but only provides male photo in Figure 1. I suggest to add both female and male photo for demonstrating sexual dimorphism of bowfin.

As requested, we now provide pictures of a representative adult bowfin male and female from the Louisiana population in Supplementary Fig. 4a,b, showing the sexual dimorphism of bowfin.

3f. The developmental stage of these 30 males and females should also be reported.

The precise developmental stage (i.e., age) of the 30 phenotypic males and females used for the Pool-sequencing approach was not recorded, but all these animals were mature adult bowfin with functional gonads. To be clear, their phenotypic sex was determined by macroscopical examination of the gonads (not just the pigmentation sexual dimorphism), leaving no doubt about their sex phenotype. This is now clearly mentioned in the texts (Results, Methods, and Supplementary Note 4).

4. Bowfin represents a once large taxonomic group that is now mostly extinct. It would be interesting to calculate the historical population size, and find out the reasons for the significantly reduced population size, such as geological events.

This is a very interesting idea, and we already allude to the importance of a detailed population genomic investigation of bowfin at the end of the Discussion where we point to studies suggesting that multiple cryptic bowfin species exist. In fact, by the 1870s up to 13 nominal species of bowfin had been described, which were later synonymized into the monotypic *Amia calva* by Jordan and Evermann (1896) without further analysis[29]. Very limited mitochondrial data by others that have not been formally published[30] suggest genetic divergence among bowfin populations across their geographic range that could support the existence of multiple bowfin species, especially when considering the slow rate of molecular evolution in holosteans[29].

The unrecognized diversity of bowfin is thus likely very complex, yet a detailed population genomic analysis as suggested by the reviewer will not be possible without increased sampling across the range of bowfin. A good representation of population level variation will be necessary

to get at this using standard population genetic approaches, but such samples are currently not at hand.

An alternative approach with the data we have also falls into the same problem. First, we would need extra data to estimate the divergence dates. There are a few possible approaches here, including a compilation of fossil data coupled with an implementation of a fossilized birth-death model to get at the crown of living bowfins (assuming the most extreme divergences represent cryptic species, which they likely do). However, this will be difficult to do without first obtaining convincing data that we are indeed dealing with multiple evolutionary distinct lineages, which brings us back to square one of a range-wide species delimitation project.

We agree that with more sampling, a detailed population genetic investigation of the genus *Amia*, guided by our reference genome, will be a great follow-up project. That said, with the samples and data hand, this is currently not feasible and well outside of the scope of the project to do properly.

5. I find the section to test the Holostei vs. Halecostomi scenarios very interesting. Focusing on the micro-chromosomes in gars, but absent in teleosts and bowfin, the authors identified orthologous chromosomes related to micro-chromosome fusions between bowfin and gar, bowfin and medaka, and found out the convergent evolution of bowfin and teleosts. Combing with the gene gain and loss and some other analysis, evidence efficiently supports the monophyly of holostean fishes. Thus settled the long-debated phylogenetic relationship with gar. My concern is that the gar genome includes 29 LGs, and the closely related bowfin has 23 LGs, while only 3 two-to-one chromosome triplets between gar and bowfin were identified based on orthologous genes. Could the authors try a syntenic analysis based on genome sequence using a circos plot or some others to better reflect the chromosome fusion and genome organization between bowfin and gar?

We thank the reviewer for this suggestion. We think that Oxford grid-type species comparisons are the most effective way to present the requested synteny data. We now present Oxford grids of bowfin vs. gar, vs. chicken, and vs. medaka in the new Extended Data Figure 2, which also includes the items of our previous supplementary figure on the comparison of holosteans genomes vs. chicken.

It is important to point out that – as can be inferred from these figures – that chromosomal rearrangements beyond the microchromosome fusions highlighted in main Figure 1 have occurred since the divergence of bowfin and gar, explaining the difference in chromosome number. The point of Figure 1 is to show that the microchromosome fusion in bowfin are completely different from those in teleosts (as shown by the medaka example) and thus do not provide any evidence for the Halecostomi scenario despite similarities of teleosts and bowfin at the gross karyotypic level.

6a. SCPP gene family encodes proteins involved in biomineralization and was the research hotspot of fish genomics and evolutionary developmental biology in recent years. In this manuscript, 22 SCPP genes were identified from two large genomic clusters in bowfin, which arranged similarly to gar. A reduced gene cluster on superscaffold 9 in bowfin genome was identified by the syntenic analysis with gar, which was helpful to the evolutionary developmental biology study of scale in ray-finned fishes. Expression evidences in gar and zebrafish, as the authors cited in the manuscript, suggested that the SCPP genes in this cluster are potentially involved in scale formation. I suggest to perform similar experiment and collect SCPP gene expression evidences in bowfin, which is vital to support the inference. The manuscript suggests

that enam and ambn genes silence their expression during scale development but not in dental enamel formation. Could the authors provide gene in situ hybridization or expression evidence to support it?

Unfortunately, such experiments are currently not possible due to the lack of scale and tooth material from the critical developmental timepoints. Scale development does not start until considerably later than the last described developmental stage 31[31] (length ~1.8cm, see main Fig. 4a). According to the literature, scale development starts at about 2.5-3cm length[32,33] and our collective team has so far not been able to grow up bowfin to this stage (we suspect this could be due to the missing paternal care). Due to weather conditions in 2019 and the ongoing COVID lockdown and restrictions since 2020, we have not been able to obtain any bowfin eggs during the past two years for additional growth attempts. Obtaining stages of the right developmental time is sheer luck, because these fish are not spawning in captivity and we rely on eggs that have been collected from wild bowfin nests. The father guards the nest and the hatched larvae. With rising water level, these so-called larval “bowfin balls” are extremely rarely seen and once the larvae disperse, they are almost impossible to find. We have not been able yet to collect larval fish of these necessary timepoints over several years of trying. Furthermore, expression of SCPP genes may not be detected in adult scales and in adult jaws. Regardless of the developmental ages, it is very challenging to obtain the skin that overlies growing scales and the jaw that contains developing teeth for expression analyses, particularly at this point given the ongoing restrictions on field work. Thus, we will have to rely on future, post-pandemic sampling attempts to be able to properly perform the suggested experiments for a follow-up study.

7. A total of 43 bona fide Hox cluster genes were identified with the same repertoire as gar. Of which, Hoxd14 was identified as a pseudogene in the bowfin genome. High expression of hoxd14 was detected in the tail bud and vent at stage 24 and in the posterior pectoral fin mesenchyme at stage 26, which was also supported by reliable gene expression evidences in paddlefish, shark and lamprey. However, gene expression evidences of hoxd14 in gar were not mentioned in the manuscript. Considering the monophyletic relationship of bowfin and gar, transcriptional evidence of hoxd14 in gar is important for better understanding the function and evolution of hoxd14 in Holostei.

We thank the reviewer for bringing up this important point and for giving us the opportunity to present our ‘negative’, but essential data. We now have added a section in Supplementary Note 11 that describes our unsuccessful attempts to clone gar *hoxd14* transcripts from cDNA, using a similar strategy to the one that successfully cloned bowfin *hoxd14*. Failing to clone gar *hoxd14* transcripts is consistent with our previous report that there is no evidence for *hoxd14* expression in our published gar RNA-Seq panel[6]. Although this makes gar *hoxd14* expression very unlikely, of course we cannot say for certain that it is not transcribed at all.

8. Both bowfin and gar are bimodal breathers that have the capacity to breathe both from water and air. They have a gas bladder that serves to maintain buoyancy, and also allows them to breathe air. It would be interesting to identify the genes or gene families associated with bimodal breathers.

The reviewer raises a very interesting aspect of bowfin biology and of our study. First, we want to point out that members of our author team (E. Funk, A. McCune) have already investigated and recently published on the development of the bowfin gas bladder in depth. Their studies explored genetic mechanisms and RNA-Seq-based gene expression patterns responsible for differences in dorsal gas bladder development in bowfin vs. ventral aquatic lung development in

bichir and ventral terrestrial lungs[34-36]. We do not intend to unnecessarily duplicate these studies and instead now more clearly highlight this important previous work in the manuscript.

In addition, other recent studies[1,37,38] have shown transcriptional similarities between bimodal gas bladders and tetrapod lungs in adult tissue. Our focus here takes a more developmental approach to vertebrate air-filled organ evolution and sheds new light on the deep homology of lungs and gas bladders at the gene regulatory level.

Previous studies by us and others[1,6,39-41] identified conservation of the *tbx4* intronic 'lung enhancer' element between tetrapods and non-teleost fishes, but a connection to teleost *tbx4* has remained elusive.

Our study here is the first to show that the *tbx4* lung enhancer region is accessible (open chromatin) during the critical stages of development gas bladder development in any ray-finned fish (Fig. 4f). In the revised version, by reanalyzing data from single cell expression and chromatin accessibility atlases[42,43], we now also show activity of this enhancer in specific cell type populations in the lung of the developing human fetus (new Extended Data Fig. 4a,b and revised Supplementary Note 9).

Importantly, using the 'holostean bridge' principle, our study is the first to show that the *tbx4* lung enhancer region is in fact conserved in teleost fishes (Fig. 4g, Extended Data Fig. 4c). In addition, we also show that a previously described putative teleost swim bladder enhancer region[44] is conserved in holosteans and located in accessible chromatin during bowfin development (Fig. 4g, Extended Data Fig. 4c).

All these new lines of evidence support lung-gas bladder homology across bony vertebrates. To further highlight this important aspect of bowfin biology, we have expanded the discussion of our *tbx4* enhancer results in the main text, now in its own dedicated new subsection '*Deep homology of vertebrate air-filled organs at the gene regulatory level*' (p.13). We expanded the related Supplementary Note 9 and elevated the 'holostean bridge' connection of *tbx4* enhancer conservation across bony vertebrates to Extended Data Figure status (Extended Data Fig. 4).

References:

1 Bi, X. *et al.* Tracing the genetic footprints of vertebrate landing in non-teleost ray-finned fishes. *Cell* **184**, 1377-1391 e1314, doi:10.1016/j.cell.2021.01.046 (2021).

2 Cheng, P. *et al.* The American paddlefish genome provides novel insights into chromosomal evolution and bone mineralization in early vertebrates. *Mol Biol Evol*, doi:10.1093/molbev/msaa326 (2020).

3 Vernimmen, D. Uncovering enhancer functions using the alpha-globin locus. *PLoS Genet* **10**, e1004668, doi:10.1371/journal.pgen.1004668 (2014).

4 Huang, P. *et al.* Comparative analysis of three-dimensional chromosomal architecture identifies a novel fetal hemoglobin regulatory element. *Genes Dev* **31**, 1704-1713, doi:10.1101/gad.303461.117 (2017).

5 Ulianov, S. V. *et al.* Activation of the alpha-globin gene expression correlates with dramatic upregulation of nearby non-globin genes and changes in local and large-scale chromatin spatial structure. *Epigenetics Chromatin* **10**, 35, doi:10.1186/s13072-017-0142-4 (2017).

6 Braasch, I. *et al.* The spotted gar genome illuminates vertebrate evolution and facilitates human-teleost comparisons. *Nat Genet* **48**, 427-437, doi:10.1038/ng.3526 (2016).

7 Near, T. J. *et al.* Resolution of ray-finned fish phylogeny and timing of diversification. *Proc Natl Acad Sci U S A* **109**, 13698-13703, doi:10.1073/pnas.1206625109 (2012).

8 Betancur, R. R. *et al.* The tree of life and a new classification of bony fishes. *PLoS currents* **5**, doi:10.1371/currents.tol.53ba26640df0ccaee75bb165c8c26288 (2013).

9 Broughton, R. E., Betancur, R. R., Li, C., Arratia, G. & Orti, G. Multi-locus phylogenetic analysis reveals the pattern and tempo of bony fish evolution. *PLoS currents* **5**, doi:10.1371/currents.tol.2ca8041495ffafd0c92756e75247483e (2013).

10 Irisarri, I. *et al.* Phylotranscriptomic consolidation of the jawed vertebrate timetree. *Nat Ecol Evol* **1**, 1370-1378, doi:10.1038/s41559-017-0240-5 (2017).

11 Hughes, L. C. *et al.* Comprehensive phylogeny of ray-finned fishes (Actinopterygii) based on transcriptomic and genomic data. *Proc Natl Acad Sci U S A* **115**, 6249-6254, doi:10.1073/pnas.1719358115 (2018).

12 Grande, L. An Empirical Synthetic Pattern Study of Gars (Lepisosteiformes) and Closely Related Species, Based Mostly on Skeletal Anatomy. The Resurrection of Holostei. *Copeia*, 1-863 (2010).

13 Takezaki, N. Global Rate Variation in Bony Vertebrates. *Genome Biol Evol* **10**, 1803-1815, doi:10.1093/gbe/evy125 (2018).

14 Takezaki, N. & Nishihara, H. Support for Lungfish as the Closest Relative of Tetrapods by Using Slowly Evolving Ray-Finned Fish as the Outgroup. *Genome Biol Evol* **9**, 93-101, doi:10.1093/gbe/evw288 (2017).

15 Liu, Z. *et al.* The channel catfish genome sequence provides insights into the evolution of scale formation in teleosts. *Nat Commun* **7**, 11757, doi:10.1038/ncomms11757 (2016).

16 Kawasaki, K. *et al.* Coevolution of enamel, ganoin, enameloid, and their matrix SCPP genes in osteichthyans. *iScience* **24**, 102023, doi:10.1016/j.isci.2020.102023 (2021).

17 Ohta, Y. *et al.* Primitive synteny of vertebrate major histocompatibility complex class I and class II genes. *Proc Natl Acad Sci U S A* **97**, 4712-4717, doi:10.1073/pnas.97.9.4712 (2000).

18 Ohta, Y., McKinney, E. C., Criscitiello, M. F. & Flajnik, M. F. Proteasome, Transporter Associated with Antigen Processing, and Class I Genes in the Nurse Shark Ginglymostoma cirratum: Evidence for a Stable Class I Region and MHC Haplotype Lineages. *The Journal of Immunology* **168**, 771, doi:10.4049/jimmunol.168.2.771 (2002).

19 Garg, S. *et al.* Chromosome-scale, haplotype-resolved assembly of human genomes. *Nat Biotechnol* **39**, 309-312, doi:10.1038/s41587-020-0711-0 (2021).

20 Majtanova, Z., Symonova, R., Arias-Rodriguez, L., Sallan, L. & Rab, P. "Holostei versus Halecostomi" Problem: Insight from Cytogenetics of Ancient Nonteleost Actinopterygian Fish, Bowfin Amia calva. *J Exp Zool B Mol Dev Evol* **328**, 620-628, doi:10.1002/jez.b.22720 (2017).

21 Ohno, S. *et al.* Microchromosomes in holocephalian, chondrostean and holostean fishes. *Chromosoma* **26**, 35-40, doi:10.1007/BF00319498 (1969).

22 Valente, G. T. *et al.* Origin and evolution of B chromosomes in the cichlid fish Astatotilapia latifasciata based on integrated genomic analyses. *Mol Biol Evol* **31**, 2061-2072, doi:10.1093/molbev/msu148 (2014).

23 Vicari, M. R. *et al.* New insights on the origin of B chromosomes in Astyanax scabripinnis obtained by chromosome painting and FISH. *Genetica* **139**, 1073-1081, doi:10.1007/s10709-011-9611-z (2011).
24 Sacerdot, C., Louis, A., Bon, C., Berthelot, C. & Roest Crollius, H. Chromosome evolution at the origin of the ancestral vertebrate genome. *Genome Biol* **19**, 166, doi:10.1186/s13059-018-1559-1 (2018).
25 Simakov, O. *et al.* Deeply conserved synteny resolves early events in vertebrate evolution. *Nat Ecol Evol* **4**, 820-830, doi:10.1038/s41559-020-1156-z (2020).
26 Kawasaki, K. & Weiss, K. M. Mineralized tissue and vertebrate evolution: the secretory calcium-binding phosphoprotein gene cluster. *Proc Natl Acad Sci U S A* **100**, 4060-4065, doi:10.1073/pnas.0638023100 (2003).
27 Faircloth, B. C., Sorenson, L., Santini, F. & Alfaro, M. E. A Phylogenomic Perspective on the Radiation of Ray-Finned Fishes Based upon Targeted Sequencing of Ultraconserved Elements (UCEs). *PLoS ONE* **8**, doi:10.1371/journal.pone.0065923 (2013).
28 Kamiya, T. *et al.* A trans-species missense SNP in Amhr2 is associated with sex determination in the tiger pufferfish, Takifugu rubripes (fugu). *PLoS Genet* **8**, e1002798, doi:10.1371/journal.pgen.1002798 (2012).
29 Sinopoli, D. *Morphological Variation of Bowfin (Amiidae: Amia calva Linnaeus 1766) Populations from the Mississippi River Basin: Taxonomic and Conservation Implications.* Master of Science thesis, State University of New York, (2019).
30 Clark, M. J. *Population Genetics of Bowfins (Amiidae, Amia spp.) Across the Laurentian Great Lakes and the Carolinas* Bachelor of Science thesis, State University of New York, (2015).
31 Ballard, W. W. Stages and rates of normal development in the holostean fish, Amia calva. *Journal of Experimental Zoology* **238**, 337-354, doi:10.1002/jez.1402380308 (1986).
32 Grande, L. & Bemis, W. E. A Comprehensive Phylogenetic Study of Amiid Fishes (Amiidae) Based on Comparative Skeletal Anatomy. an Empirical Search for Interconnected Patterns of Natural History. *Journal of Vertebrate Paleontology* **18**, 1-696, doi:10.1080/02724634.1998.10011114 (1998).
33 Burr, B. M. & Bennett, M. G. in *Freshwater Fishes of North America* Vol. 1 (eds M. L. Warren & M. G. Burr) (John Hopkins Univesity Press, 2014).
34 Funk, E., Lencer, E. & McCune, A. Dorsoventral inversion of the air-filled organ (lungs, gas bladder) in vertebrates: RNAsequencing of laser capture microdissected embryonic tissue. *J Exp Zool B Mol Dev Evol*, doi:10.1002/jez.b.22998 (2020).
35 Funk, E. C., Birol, E. B. & McCune, A. R. Does the bowfin gas bladder represent an intermediate stage during the lung-to-gas bladder evolutionary transition? *J Morphol* **282**, 600-611, doi:10.1002/jmor.21330 (2021).
36 Funk, E. C., Breen, C., Sanketi, B. D., Kurpios, N. & McCune, A. Changes in Nkx2.1, Sox2, Bmp4 and Bmp16 expression underlying the lung-to-gas bladder evolutionary transition in ray-finned fishes. *Evolution and Development* **in press** (2020).
37 Meyer, A. *et al.* Giant lungfish genome elucidates the conquest of land by vertebrates. *Nature* **590**, 284-289, doi:10.1038/s41586-021-03198-8 (2021).
38 Wang, K. *et al.* African lungfish genome sheds light on the vertebrate water-to-land transition. *Cell* **184**, 1362-1376 e1318, doi:10.1016/j.cell.2021.01.047 (2021).
39 Hara, Y. *et al.* Shark genomes provide insights into elasmobranch evolution and the origin of vertebrates. *Nat Ecol Evol* **2**, 1761-1771, doi:10.1038/s41559-018-0673-5 (2018).
40 Onimaru, K. The evolutionary origin of developmental enhancers in vertebrates: Insights from non-model species. *Dev Growth Differ* **62**, 326-333, doi:10.1111/dgd.12662 (2020).
41 Tatsumi, N. *et al.* Molecular developmental mechanism in polypterid fish provides insight into the origin of vertebrate lungs. *Sci Rep* **6**, 30580, doi:10.1038/srep30580 (2016).
42 Cao, J. *et al.* A human cell atlas of fetal gene expression. *Science* **370**, doi:10.1126/science.aba7721 (2020).
43 Domcke, S. *et al.* A human cell atlas of fetal chromatin accessibility. *Science* **370**, doi:10.1126/science.aba7612 (2020).
44 Nikaido, M. *et al.* Coelacanth genomes reveal signatures for evolutionary transition from water to land. *Genome Res* **23**, 1740-1748, doi:10.1101/gr.158105.113 (2013).

Decision Letter, first revision:

22nd Apr 2021

Dear Ingo,

Your Article, "The genome of the bowfin (Amia calva) illuminates the developmental evolution of ray-finned fishes" has now been seen by the 3 original referees. You will see from their comments below that while they find your work improved, some important points are raised by reviewers #2 and #3. We are interested in the possibility of publishing your study in Nature Genetics, but would like to consider your response to these concerns in the form of a revised manuscript before we make a final decision on publication.

We therefore invite you to revise your manuscript taking into account all reviewer comments. Please highlight all changes in the manuscript text file. At this stage we will need you to upload a copy of the manuscript in MS Word .docx or similar editable format.

We are committed to providing a fair and constructive peer-review process. Do not hesitate to contact me if there are specific requests from the reviewers that you believe are technically impossible or unlikely to yield a meaningful outcome.

When revising your manuscript:

*1) Include a “Response to referees” document detailing, point-by-point, how you addressed each referee comment. If no action was taken to address a point, you must provide a compelling argument. This response may be sent back to the referees along with the revised manuscript.

*2) If you have not done so already please begin to revise your manuscript so that it conforms to our Article format instructions, available
here.
Refer also to any guidelines provided in this letter.

*3) Include a revised version of any required Reporting Summary:
https://www.nature.com/documents/nr-reporting-summary.pdf
It will be available to referees (and, potentially, statisticians) to aid in their evaluation if the manuscript goes back for peer review.
A revised checklist is essential for re-review of the paper.

Please be aware of our guidelines on digital image standards.

Please use the link below to submit your revised manuscript and related files:

[REDACTED]

Note: This URL links to your confidential home page and associated information about manuscripts you may have submitted, or that you are reviewing for us. If you wish to forward this email to co-authors, please delete the link to your homepage.

Please do not hesitate to contact me if you have any questions or would like to discuss these revisions further.

Nature Genetics is committed to improving transparency in authorship. As part of our efforts in this direction, we are now requesting that all authors identified as 'corresponding author' on published papers create and link their Open Researcher and Contributor Identifier (ORCID) with their account on the Manuscript Tracking System (MTS), prior to acceptance. ORCID helps the scientific community achieve unambiguous attribution of all scholarly contributions. You can create and link your ORCID from the home page of the MTS by clicking on 'Modify my Springer Nature account'. For more information please visit please visit www.springernature.com/orcid.

We look forward to seeing the revised manuscript and thank you for the opportunity to review your work.

Sincerely,

Tiago

Tiago Faial, PhD
Senior Editor
Nature Genetics
https://orcid.org/0000-0003-0864-1200

Reviewers' Comments:

Reviewer #1:
Remarks to the Author:
I think the authors did a good job.
The authors clearly addressed my comments and also described the additional topics on the gene regulation networks of vertebrate air-filled organs.
I have no more comments on this manuscript and it is acceptable for publication.

Reviewer #2:
Remarks to the Author:
The authors have solved most of the questions or concerns I have raised, but one major comment has

yet to be solved.

In the analysis of batch effects, I agree with the authors on the existence of noise in the full data. However, in general, many protein-coding genes have multiple functions. Therefore, the authors should: 1) provide the list of the 100 limb development genes to the readers to make the result more repeatable; 2) clarify the criteria and definition of limb development genes; 3) use groups of random gene list to evaluate batch effects, and further check the result and conclusion.

Reviewer #3:
Remarks to the Author:
As the nodal organisms of tetrapod and ray-finned fish evolution, the genomic resources of bowfin and gar were indispensable in the developmental evolution of ray-finned fishes. In this analysis, the authors reported the first chromosome-level bowfin genome assembly with 23 chromosomes, investigated the monophyly of holostean fish, focused on the sex determination, immunogenome, SCPP gene family, Hox gene cluster, and fin development, which were important research in teleost evolution. The authors added several necessary explanations and discussions, and significantly improved the manuscript.
In the meantime of the authors' first revision, Bi et al. published chromosome-level bichir genome, as well as scaffold-level genomes of bowfin, paddlefish and alligator gar, and then elucidated the phylogeny and comparative genomics between Holostei and Teleostei (Bi et al. (2021); doi: 10.1016/j.cell.2021.01.046). Despite the manuscript reporting the first high quality chromosome-level reference genome of bowfin, the novelty about "monophyly of holostean fishes" based on genome-scale information is compromised by Bi's paper. The comparison of genomic collinearity between bichir and bowfin is necessary to understand genome organization and gene order differences in Holostei in the next revision.
I was disappointed about the studies of sex determination. The current results still cannot clearly explain the mechanism of sex determination in bowfin. I suggest to remove this section from the manuscript.
For the scale formation section, it's unfortunate that the bowfin cannot grow up to the stage of scale formation in the laboratory. This undoubtedly weakens the reliability of this part of inference to a certain extent.

Author Rebuttal, first revision:

Response to Reviewers

We would like to thank again all three reviewers for their helpful comments and thoughtful suggestions that have significantly improved this study. Below we address each of the remaining individual points in detail.

Reviewer #1:

I think the authors did a good job.
The authors clearly addressed my comments and also described the additional topics on the gene regulation networks of vertebrate air-filled organs.
I have no more comments on this manuscript and it is acceptable for publication.

We thank the reviewer for the supportive evaluation and the prior constructive feedback that has improved our study.

Reviewer #2:

The authors have solved most of the questions or concerns I have raised, but one major comment has yet to be solved.

We thank the reviewer for the highly valuable feedback and supportive evaluation of the prior revision of our study. We address the remaining comment in full detail below.

In the analysis of batch effects, I agree with the authors on the existence of noise in the full data. However, in general, many protein-coding genes have multiple functions. Therefore, the authors should: 1) provide the list of the 100 limb development genes to the readers to make the result more repeatable; 2) clarify the criteria and definition of limb development genes; 3) use groups of random gene list to evaluate batch effects, and further check the result and conclusion.

We fully agree with the reviewer that protein coding genes can be highly pleiotropic, especially developmental toolkit genes. Thus, referring to any gene simply as a "limb gene" in shorthand can be misleading. The set of 100 genes was selected a priori as a list of candidate genes to examine development in the context of the bowfin fin, based on genes with known roles in fin and limb patterning and their implication in the fin-to-limb transition. As many of these genes have roles beyond the fin and limb, we now refer to this list more generally as "100 Developmental Patterning Genes."

To further address the reviewer's requests, we have taken the following steps:

1) We now provide the list of 100 Developmental Patterning Genes as new Supplementary Table 25, including the RPKM values for each gene and each sequencing library.

2) As clarified in the revised Supplementary Note 11.2, this list was manually curated based on extensive analysis of the fin and limb development literature. Criteria for inclusion in this list are genes and gene families that (1) are known to affect fin and limb patterning, and/or (2) have been implicated in the fin-to-limb transition. These include genes that have roles in the proximal-distal patterning, anterior-posterior patterning, appendage bud initiation, outgrowth, and the apical ectodermal ridge to apical ectodermal fold transition in fins and limbs. Importantly, as the GO terms related to fin and limb development are not well curated and omit critical patterning genes, we used this list to manually curate a more specific gene set. GO terms 'Fin Development' (GO:0033333) and 'Limb Development' (GO:0060173) for instance both lack all Meis gene family members, Ptch2, Twist2, and Grem2, among others.

3) Following the reviewer's suggestion to use random subsamples to assess batch effects in our data, we have run PCAs on sets of 100 and 1,000 randomly selected genes and show exemplary sets in the new Supplementary Fig. 18 c, d. These random subsamples reflect the noise seen in the full data set as they fail to group samples by sequencing approach or developmental stage.

As stated in the revised Supplementary Note 11.2, we thus conclude that insights from the fin bud transcriptome data set are most valid when restricted to developmental patterning genes. Batch effects due to sequencing approach may exist for individual genes, but this effect is overshadowed by the general noise in the data set.

Reviewer #3:

As the nodal organisms of tetrapod and ray-finned fish evolution, the genomic resources of bowfin and gar were indispensable in the developmental evolution of ray-finned fishes. In this analysis, the authors reported the first chromosome-level bowfin genome assembly with 23 chromosomes, investigated the monophyly of holostean fish, focused on the sex determination, immunogenome, SCPP gene family, Hox gene cluster, and fin development, which were important research in teleost evolution. The authors added several necessary explanations and discussions, and significantly improved the manuscript.

We are encouraged by the reviewer's enthusiasm for the changes made based on the prior round of review.

1. In the meantime of the authors' first revision, Bi et al. published chromosome-level bichir genome, as well as scaffold-level genomes of bowfin, paddlefish and alligator gar, and then elucidated the phylogeny and comparative genomics between Holostei and Teleostei (Bi et al. (2021); doi: 10.1016/j.cell.2021.01.046). Despite the manuscript reporting the first high quality chromosome-level reference genome of bowfin, the novelty about "monophyly of holostean fishes" based on genome-scale information is compromised by Bi's paper.

We respectfully disagree with the notion that the work of Bi et al. (2021)[1], although impressive, reduces the impact of our findings. If anything, the comparisons between their findings and ours reinforces the conclusions and quality of the work we present on the bowfin.

Importantly, Bi et al. (2021) performed only *sequenced-based* phylogenomic analysis of ray-finned fish relationships in agreement with the already existing large body of literature that favors the Holostei scenario of neopterygian relationships, also based on sequence data. We cite Bi et al. (2021) along with other published sequenced-based phylogenomic investigations. Bi et al. (2021), however, do not discuss the important controversy over neopterygian relationships or holostean monophyly at all, nor does their study focus on the comparison of holosteans and teleosts (it focuses almost entirely on the comparison of ray-finned fish vs. lobe-finned fish and tetrapods).

We want to point out again that our study presents a highly novel approach to reconstruct the phylogenetic relationships of neopterygians that uses data from *gene order rearrangements* (gene adjacency) in the genomes of bony vertebrates. Such an approach has not been covered by Bi et al. (2021) or any other previous study. Our work here thus not only puts the 'gar-*Amia*-teleost problem' to rest; it also offers a novel and unique approach leveraging the phylogenetic signal in gene orders from chromosome-level genome assemblies that can be applied to many other controversial phylogenetic relationships in the future.

2. The comparison of genomic collinearity between bichir and bowfin is necessary to understand genome organization and gene order differences in Holostei in the next revision.

We have now done specific comparisons between the holostean and bichir genomes. In a new Supplementary Fig. 5 we now include the genome comparisons of bowfin vs. bichir and gar vs. bichir. These comparisons show that bowfin and bichir independently evolved derived states of genome organization, fusing micro-chromosomes and macro-chromosomes in lineage-specific ways so that both genomes no longer contain micro-chromosomes. Supplementary Fig. 5 shows that the fusions involving ancestral micro-chromosomes are different in bichir from those in bowfin as well as from those in teleosts (shown Fig. 1).

This analysis further strengthens our conclusion that bowfin pseudochromosome 13 and gar LG6 are derived from a holostean-specific chromosome fusion, further supporting holostean monophyly.

In the main text, we now make two explicit references to the bichir genome comparison and say:

> "These fusions are also not shared with micro-chromosome fusions in the derived genome of bichir (Supplementary Fig. 5), representing the most basally diverging extant ray-finned fish lineage (Bi et al. 2021). We conclude that bowfin, teleosts, and bichir independently fused different sets of micro-chromosomes. The karyotypic similarities of bowfin and teleosts are the result of convergent evolution rather than a common origin and thus do not support the Halecostomi scenario."

"… in support of holostean monophyly, chicken chromosomes 13 and 23 correspond to a fusion chromosome in both bowfin and gar (Extended Data Fig. 2f), a rearrangement neither found in teleosts nor in bichir (Supplementary Fig. 5)."

3. I was disappointed about the studies of sex determination. The current results still cannot clearly explain the mechanism of sex determination in bowfin. I suggest to remove this section from the manuscript.

Given our data, we can exclude that bowfin has a simple monofactorial sex determination system with well-differentiated sex chromosomes. While it may seem 'disappointing' to not find a clear-cut sex determining system, such observation is commonplace in fishes such as in the zebrafish where, even in this well-studied system, the mechanisms of sex determination are not yet elucidated.

While our analysis does not offer a definite explanation for sex determination in bowfin, we maintain that our results provide highly valuable biological information that should be reported. Our fine-grained Pool-Seq results are in line with previous karyotype studies in bowfin[2,3] that did not find any obviously visible sex chromosomes either. Clear-cut sex determination systems, however, should not be expected to be the norm in vertebrates, especially in fishes (e.g.[4,5]). In contrast, our findings show that bowfin sex determination is very likely polyfactorial and/or environmentally influenced, as it is the case for many fish species (e.g.[6]), or monofactorial with a very small differentiation of sex chromosomes as previously shown in some pufferfish species[7]. Importantly, we previously found a very similar situation for spotted gar[5], suggesting that all holosteans may share an absence of sex chromosome differentiation at the molecular level. Our finding of complex holostean sex determination systems will guide future studies that investigate the mechanistic details.

To address the reviewer's concern, we reduced the text devoted to sex determination in the main article. Instead of featuring this part in its own Results subsection, we condensed our results into three sentences as last paragraph of the previous subsection on the general genome and assembly characterization (which we hence renamed with the more inclusive title "The bowfin genome"). All detailed information has now been moved to Supplementary Note 3. In addition, to keep all information on this part in one place, we moved the detailed methods description for the sex determination analysis to Supplementary Note 3 as well. We think this nicely responds to the concern while maintaining access and presentation of the data.

4. For the scale formation section, it's unfortunate that the bowfin cannot grow up to the stage of scale formation in the laboratory. This undoubtedly weakens the reliability of this part of inference to a certain extent.

We agree that additional functional investigation of scale formation in bowfin would be ideal. However, it should be noted that our investigation here is the most detailed analysis of SCPP

gene repertoires among ray-finned fish to date. Importantly, our investigation uniquely generates testable hypotheses about the function of specific SCPP genes with respect to scale formation that we will address in future studies should the necessary bowfin samples become available.

To address the reviewer's point and to highlight the novel hypotheses based on our comparative analysis, we have rephrased the last sentence of the scale subsection in the main text and now say:

> "We thus hypothesize that reduced biomineralization of the bowfin scale is attributed to both changes in gene regulation and the loss of specific SCPP genes."

In addition, we added the following, concluding sentence to Supplementary Note 7:

> "In summary, our comparative analysis of holostean SCPP genes reveals the gene cluster on gar LG2 and bowfin pseudochromosome 9 as a putative hot-spot for scale phenotype variation across ray-finned fishes and generates testable hypotheses for future functional investigations."

All things considered, we feel that the presentation and discussion of this work nicely highlights key questions in ray-finned biology as it relates to vertebrate evolution. The vignettes detail important aspects of unique biology that the bowfin presents as well as areas in which inclusion of bowfin genomic data resolves comparison across holostean and bony vertebrates more broadly. The fundamental findings we made here on the genetic basis of holostean development and evolution shows the importance of this high-quality genome and its application in understanding the unique biology of this group of fishes.

References:

1 Bi, X. *et al.* Tracing the genetic footprints of vertebrate landing in non-teleost ray-finned fishes. *Cell* **184**, 1377-1391 e1314, doi:10.1016/j.cell.2021.01.046 (2021).

2 Ohno, S. *et al.* Microchromosomes in holocephalian, chondrostean and holostean fishes. *Chromosoma* **26**, 35-40, doi:10.1007/BF00319498 (1969).

3 Majtanova, Z., Symonova, R., Arias-Rodriguez, L., Sallan, L. & Rab, P. "Holostei versus Halecostomi" Problem: Insight from Cytogenetics of Ancient Nonteleost Actinopterygian Fish, Bowfin Amia calva. *J Exp Zool B Mol Dev Evol* **328**, 620-628, doi:10.1002/jez.b.22720 (2017).

4 Bachtrog, D. *et al.* Sex determination: why so many ways of doing it? *PLoS Biol* **12**, e1001899, doi:10.1371/journal.pbio.1001899 (2014).

5 Feron, R. *et al.* RADSex: A computational workflow to study sex determination using restriction site-associated DNA sequencing data. *Mol Ecol Resour*, doi:10.1111/1755-0998.13360 (2021).

6 Guiguen, Y., Fostier, A. & Herpin, A. in *Sex Control in Aquaculture* (eds H.-P. Wang, F. Piferrer, S.-L. Chen, & Z.-G. Shen) 35-63 (2018).

7 Kamiya, T. *et al.* A trans-species missense SNP in Amhr2 is associated with sex determination in the tiger pufferfish, Takifugu rubripes (fugu). *PLoS Genet* **8**, e1002798, doi:10.1371/journal.pgen.1002798 (2012).

Decision Letter, second revision:

Our ref: NG-A56077R1

20th May 2021

Dear Ingo,

Thank you for submitting your revised manuscript "The genome of the bowfin (Amia calva) illuminates the developmental evolution of ray-finned fishes" (NG-A56077R1). My colleagues and I find that the paper has improved in revision, and therefore we'll be happy in principle to publish it in Nature Genetics, pending minor revisions to comply with our editorial and formatting guidelines.

We are now performing detailed checks on your paper and will send you a checklist detailing our editorial and formatting requirements in about a week. Please do not upload the final materials and make any revisions until you receive this additional information from us.

Thank you again for your interest in Nature Genetics. Please do not hesitate to contact me if you have any questions.

Congratulations!

Sincerely,

Tiago

Tiago Faial, PhD
Senior Editor
Nature Genetics
https://orcid.org/0000-0003-0864-1200

Final Decision Letter:

In reply please quote: NG-A56077R2 Braasch

13th Jul 2021

Dear Ingo,

I am delighted to say that your manuscript "The bowfin genome illuminates the developmental evolution of ray-finned fishes" has been accepted for publication in an upcoming issue of Nature

Genetics.

Prior to setting your manuscript, we may make minor changes to enhance the lucidity of the text and with reference to our house style. We therefore ask that you examine the proofs most carefully to ensure that we have not inadvertently altered the sense of your text in any way.

Once your manuscript is typeset and you have completed the appropriate grant of rights, you will receive a link to your electronic proof via email with a request to make any corrections within 48 hours. If, when you receive your proof, you cannot meet this deadline, please inform us at rjsproduction@springernature.com immediately.

Your paper will be published online after we receive your corrections and will appear in print in the next available issue. You can find out your date of online publication by contacting the Nature Press Office (press@nature.com) after sending your e-proof corrections. Now is the time to inform your Public Relations or Press Office about your paper, as they might be interested in promoting its publication. This will allow them time to prepare an accurate and satisfactory press release. Include your manuscript tracking number (NG-A56077R2) and the name of the journal, which they will need when they contact our Press Office.

Before your paper is published online, we shall be distributing a press release to news organizations worldwide, which may very well include details of your work. We are happy for your institution or funding agency to prepare its own press release, but it must mention the embargo date and Nature Genetics. Our Press Office may contact you closer to the time of publication, but if you or your Press Office have any enquiries in the meantime, please contact press@nature.com.

Acceptance is conditional on the data in the manuscript not being published elsewhere, or announced in the print or electronic media, until the embargo/publication date. These restrictions are not intended to deter you from presenting your data at academic meetings and conferences, but any enquiries from the media about papers not yet scheduled for publication should be referred to us.

Please note that <i>Nature Genetics</i> is a Transformative Journal (TJ). Authors may publish their research with us through the traditional subscription access route or make their paper immediately open access through payment of an article-processing charge (APC). Authors will not be required to make a final decision about access to their article until it has been accepted. Find out more about Transformative Journals

<B>Authors may need to take specific actions to achieve compliance with funder and institutional open access mandates. For submissions from January 2021, if your research is supported by a funder that requires immediate open access (e.g. according to Plan S principles) then you should select the gold OA route, and we will direct you to the compliant route where possible. For authors selecting the subscription publication route our standard licensing terms will need to be accepted, including our self-archiving policies. Those standard licensing terms will supersede any other terms that the author or any third party may assert apply to any version of the manuscript.

Please note that Nature Research offers an immediate open access option only for papers that were first submitted after 1 January, 2021.

You will not receive your proofs until the publishing agreement has been received through our system.

If you have any questions about our publishing options, costs, Open Access requirements, or our legal forms, please contact ASJournals@springernature.com

If you have posted a preprint on any preprint server, please ensure that the preprint details are updated with a publication reference, including the DOI and a URL to the published version of the article on the journal website.

To assist our authors in disseminating their research to the broader community, our SharedIt initiative provides you with a unique shareable link that will allow anyone (with or without a subscription) to read the published article. Recipients of the link with a subscription will also be able to download and print the PDF.

As soon as your article is published, you will receive an automated email with your shareable link.

You can now use a single sign-on for all your accounts, view the status of all your manuscript submissions and reviews, access usage statistics for your published articles and download a record of your refereeing activity for the Nature journals.

An online order form for reprints of your paper is available at https://www.nature.com/reprints/author-reprints.html. Please let your coauthors and your institutions' public affairs office know that they are also welcome to order reprints by this method.

Sincerely,

Tiago

Tiago Faial, PhD
Senior Editor
Nature Genetics
https://orcid.org/0000-0003-0864-1200
